# Complexity of Guanine Quadruplex Unfolding Pathways Revealed by Atomistic Pulling Simulations

**DOI:** 10.1021/acs.jcim.3c00171

**Published:** 2023-07-17

**Authors:** Petr Stadlbauer, Vojtěch Mlýnský, Miroslav Krepl, Jiří Šponer

**Affiliations:** Institute of Biophysics of the Czech Academy of Sciences, Královopolská 135, Brno 612 00, Czech Republic

## Abstract

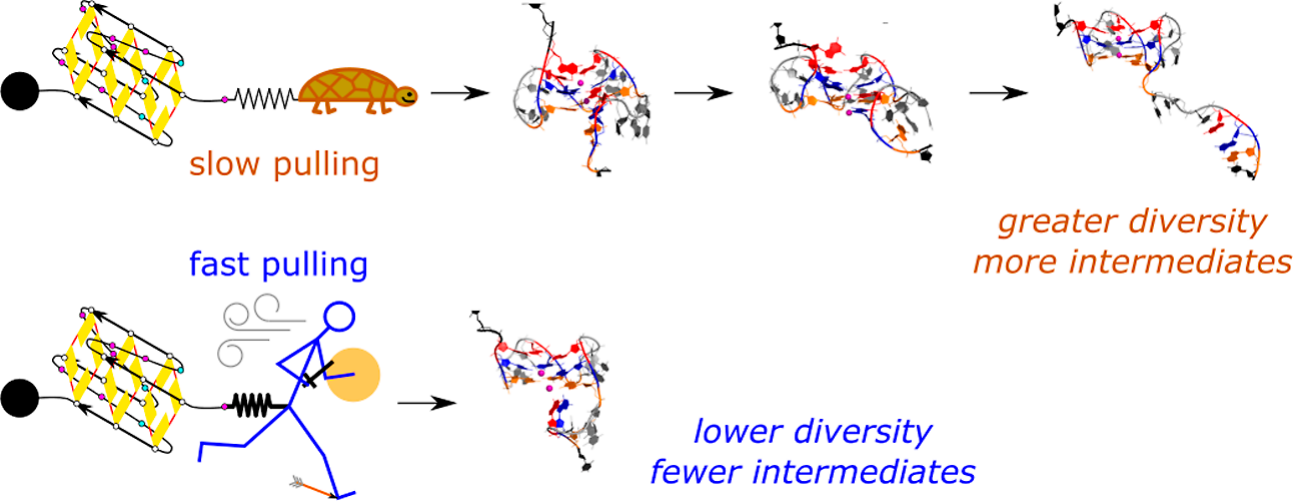

Guanine quadruplexes (GQs) are non-canonical nucleic acid structures involved in many biological processes. GQs formed in single-stranded regions often need to be unwound by cellular machinery, so their mechanochemical properties are important. Here, we performed steered molecular dynamics simulations of human telomeric GQs to study their unfolding. We examined four pulling regimes, including a very slow setup with pulling velocity and force load accessible to high-speed atomic force microscopy. We identified multiple factors affecting the unfolding mechanism, i.e.,: (i) the more the direction of force was perpendicular to the GQ channel axis (determined by GQ topology), the more the base unzipping mechanism happened, (ii) the more parallel the direction of force was, GQ opening and cross-like GQs were more likely to occur, (iii) strand slippage mechanism was possible for GQs with an all-*anti* pattern in a strand, and (iv) slower pulling velocity led to richer structural dynamics with sampling of more intermediates and partial refolding events. We also identified that a GQ may eventually unfold after a force drop under forces smaller than those that the GQ withstood before the drop. Finally, we found out that different unfolding intermediates could have very similar chain end-to-end distances, which reveals some limitations of structural interpretations of single-molecule spectroscopic data.

## Introduction

Guanine quadruplexes (GQs) are a common non-canonical form of nucleic acids. They are formed by sequences rich in guanine (G), which are widespread—hundreds of thousands of known and putative sites have been identified in the human genome.^1,2^ Notably, GQs are abundant in gene regulatory parts and in the telomeric region, where they play roles in maintaining cell function and genome integrity.^3−7^ Mutations leading to over- or under-formation of G4s often lead to diseases, such as cancer^8−12^ or neuropathologies.^13−15^

**Figure 1:**
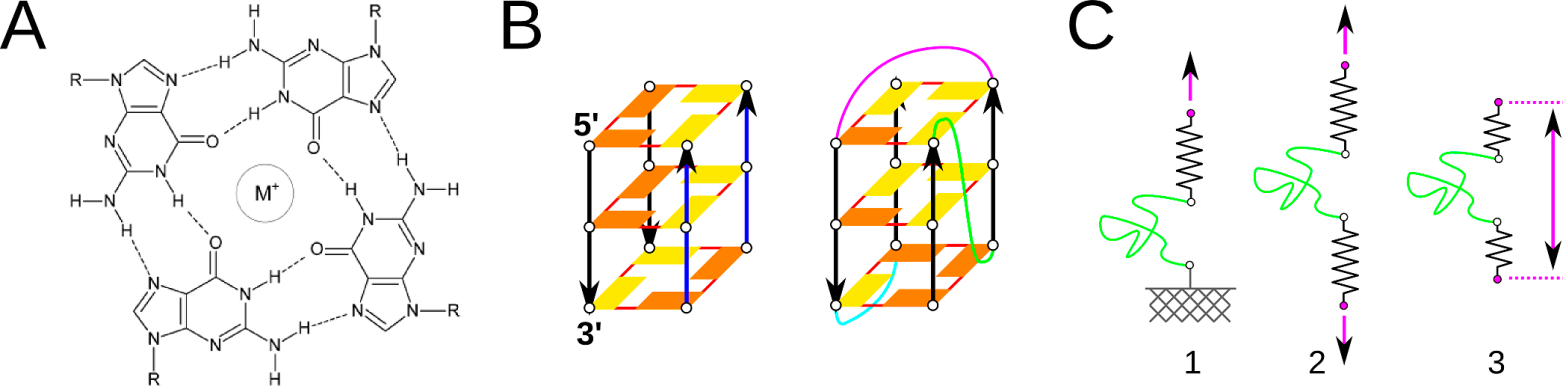
(A) G-quartet with a metal cation in the central cavity. “R” represents the sugar-phosphate moiety. (B) Illustrative GQ models. Guanines with the *anti* and *syn* glycosidic torsion χ orientation are displayed as rectangles in yellow and orange, respectively. Solid red lines indicate *cis* Watson–Crick Hoogsteen (*c*WH) base pairing. The bottom quartet is of opposite directionality than the middle and top ones. In the left model, the two blue arrows represent mutually parallel strands, and so do the two black arrows, but the black and blue strands are mutually antiparallel. In the right model, propeller, diagonal, and lateral loops are shown as green, purple, and cyan lines, respectively. The quartet including G closest to the 5′-end, is called “first”, and the indexing continues from it. See Figure 2 for examples of GQ polymorphism. (C) Illustrative schemes of common force-induced unfolding simulation techniques: (1) One selected point (white circle) of a molecule (green line) is connected by a virtual spring to a virtual particle (purple circle) that is moving in a predefined direction and one point is fixed in space (marked as a connection to the gray wall). (2) Instead of a fixed point, there is another connection by the second spring to a particle moving in the opposite direction. (3) Two points connected to a molecule are moving away from each other but not in a predefined direction, only their mutual distance is time-dependent. The springs are not independent but behave as if being just one, i.e., potential energy added by the spring depends on the molecule’s end-to-end distance as in (1) but without a fixed point. Pulling protocols used in this work employ the implementation displayed as option (3).

A basic structural unit of GQ is a G-quartet (quartet). It is composed of four Gs bound in a cyclical arrangement, where each G is connected to two neighboring Gs by *cis*-Watson–Crick–Hoogsteen (*c*WH) H-bonding (Figure 1).^16^ GQ is formed by stacking of quartets together. A channel, whose walls are covered by carbonyl O6 atoms, runs through the GQ center and is occupied by positively charged cations (e.g., K^+^ or Na^+^). Intramolecular GQs have loops, which are nucleotide segments connecting the G-strands (G-stretches, columns) (Figure 1). Loops connecting two neighboring G-strands running in the same direction, i.e., parallel, are called propeller; lateral loops connect two neighboring antiparallel strands, while diagonal loops connect two antiparallel strands across the GQ. G-strand directionality is interdependent with the *syn*/*anti* conformation of the Gs’ glycosidic torsion angle χ.^17−19^ If two Gs in a quartet are in mutually parallel strands, they have the same χ conformation. If they are in antiparallel strands, they have opposite χ conformation. These rules have implications for the structural polymorphism and folding pathways of GQs.^20^ The human telomeric sequence d(GGGTTA)_*n*_ is a prime example of such a highly polymorphic sequence, forming at least six known stable GQ conformations.^21−29^

Recent studies have suggested that, globally, GQ folding is a complicated process best described by kinetic partitioning.^30−33^ It has been suggested that the idealized folding process of a GQ ensemble can be divided into two stages.^20,34^ The first stage is characterized by fast folding of the initial ensemble into various mostly misfolded GQs with non-native *syn*/*anti* combination and/or reduced number of quartets in comparison to fully folded GQs. The second stage of the folding process is the slow refining of the initial GQ population into the final (native) GQs population. Structural transitions between the misfolded and native GQs happen via various ensembles of other structures. For example, depending on the sequence and target GQ topology, incomplete or perturbed GQs,^35−40^ G-triplexes,^38−51^ G-hairpins,^36,38,39,41,43,45,52^ or cross-like species^34,53−55^ have been hypothesized to participate in the process. The exact nature of these ensembles also depends on external factors, such as temperature, ionic strength, the presence of cosolvents, ligands, specific cations, or other nearby structural elements.^20^

A way to affect the free-energy landscape area that the GQ folding sequence traverses is to put the molecule under tension. The external force typically pulling the sequence ends away from each other results in exploration of a lower-entropy section of its free-energy surface. External forces acting on DNA are actually common in biological systems and many of its functions are related to its mechanochemical properties.^56,57^ DNA needs to be unwound to be readable for polymerases or telomerase; additional specialized enzymes—helicases—are often recruited to do the job, probably acting by moving along a DNA strand, exerting force on the GQ until it eventually unfolds.^6,56−60^ Another source of tension in GQ structures, relevant mostly for promoter GQs, comes from superhelical coiling. Force-induced unfolding can be studied in *in vitro* by single-molecule force spectroscopy techniques, such as magnetic, optical, and nanopore tweezers or atomic force microscopy (AFM). These have been applied to investigate the conformational and mechanical behavior of biopolymers and their interactions, and they can operate using various force-exertion regimes, typically: (i) constant velocity, (ii) force ramp, (iii) force clamp, (iv) pulse chase, and (v) zig-zag force ramp.^61−64^ Usually long handles/linkers are connected to the studied biopolymer; one handle is fixed, and the other is connected to a moving surface, which is moved toward and away from the fixed end, and the tension is transferred through the linker to the studied biomolecule.

Human telomeric sequence GQs have been widely studied by force spectroscopy techniques. It has been shown that the conformations formed in Na^+^ (presumably the basket topology) are mechanically less stable than the topologies folded in K^+^ (3 + 1 hybrid topology) (e.g., by rupture forces of ∼10 pN vs ∼20 pN at loading rates of ∼2–5 pN/s).^65−69^ The conformational polymorphism of the telomeric sequence in the presence of K^+^ has been demonstrated too, with the detection of a few distinct states,^66,70^ later extended to at least six distinct states.^67^ Surprisingly, the authors of the latter study also found that having more than four repeats of the telomeric sequence actually decreases the conformational diversity.^71^ A study focused on the effect of pyridostatin has revealed that the ligand promotes folding of the human telomeric GQ.^65^ Non-telomeric GQs have been investigated too. It has been shown that GQs with bulges and decreased number of quartets are less stable than complete GQs.^72^ Conversely, increasing K^+^ concentration has been shown to increase the stability of a three- and four-quartet antiparallel bimolecular GQ.^73^ High mechanical stability (40–55 pN) of parallel-stranded GQs formed by the c-MYC (force load 0.2 pN/s),^74^ hTERT (force load 5.5 pN/s),^75^ and BCL-2 (force load 2 pN/s)^76^ promoter sequences has been reported; regarding BCL-2, the same study also suggests that the parallel conformer is more stable than hybrid (20–30 pN) topologies. Similarly, high stability of various other parallel-stranded GQs has been reported recently (force load 2 pN/s).^68^ Notice that direct comparison of stability of such three-quartet promoter GQs with the telomeric GQs might be misleading because promoter GQs operate under additional superhelical stress in vivo, and they are often formed by non-repetitive sequences, so their topology could be more complicated (e.g., containing V-loops or bulges). On the other hand, an investigation of four-quartet GQs revealed that unfolding of antiparallel GQs required higher rupture forces than the parallel ones (force loads 2–24 pN/s).^77,78^ In this context, the stall (arrest) force of polymerases is ∼15 pN^79−81^ (for comparison, the stall force of the motor protein kinesin is just ∼5 pN^82^), which means that GQs may act as roadblocks and common polymerases have to employ other strategies to overcome the GQ obstacle.^58^ The coexistence of many GQ structures in the human telomeric sequence and possibly even more folding intermediates is a potential source of ambiguities. For example, several force spectroscopy studies suggest that the human telomeric GQ unfolds via a G-triplex intermediate,^44,46,47^ while there is no unambiguous evidence thereof in a recent one.^67^

Theoretical modeling approaches are an alternative tool to study the GQ mechanical unfolding with the potential to complement the experimental data. Among the methods, molecular dynamics (MD) simulations have been widely employed. The short potential of mean force simulations of the 15-thrombin binding aptamer revealed unfolding of the GQ by unzipping its strands one by one so that a G-triplex and G-hairpin were identified as intermediates under the stretching conditions.^83^ Steered MD (SMD) simulations of parallel GQ formed by the human telomeric sequence led to the identification of two different unfolding pathways, specifically the division of the GQ into two G-hairpins and strand detachment with a G-triplex remaining.^84^ The SMD method was later applied to three human telomeric sequence GQ topologies—parallel, hybrid-1, and antiparallel;^85^ all topologies unfolded into a G-triplex (or a perturbed one), but the first two by strand detachment, while the antiparallel GQ experienced a step-wise unzipping of nucleotides from both ends. A comparison of the force curves revealed that the rupture force decreased in the order antiparallel > hybrid > parallel GQ. However, a common drawback of those pioneering computational studies^84,85^ is that the authors, due to the limited computational resources, had to resort to short simulations with drastically higher (by approximately 12 orders of magnitude) force loads than those commonly applied in the force spectroscopic studies of GQs, which led to unrealistically high rupture forces. This limitation could have affected the unfolding pathways and prevented the detection of important intermediates during the unfolding process. A mesoscopic model has also been developed with the aim to apply lower force loads during SMD simulations of the parallel human telomeric GQ.^86^ However, the simplistic nature of the model (one bead per nucleotide) led to GQ unfolding via unusual G-quartet rotations (i.e., major changes in GQ helicity) and elongations. Hence, fully atomistic explicit solvent models still remain the gold standard to model the structural dynamics of GQs.

In this study, we applied the all-atom SMD method on the parallel, hybrid-1, and antiparallel topology of the human telomeric sequence and three other GQs derived from the native ones, as well as on six G-triplex structures related to the telomeric GQs. SMD simulations belong to enhanced sampling methods and are able to drag systems from initial configurations to final ones along predefined degrees of freedom called collective variables.^87^ SMDs were designed to mimic (and complement) experimental force spectroscopy techniques, with two notable differences in addition to the abovementioned gap in used force loads: (i) molecules may be allowed to freely rotate in space during SMD simulations and thus fully adjust to the direction of an external force and (ii) presence of additional molecules, called linkers, that are attached to the studied system and could (to unknown degree) affect the studied molecule’s free-energy surface during experimental measurements, is not required during SMD simulations (Figure 1C).^88−90^ We probed the effect of topology, *syn/anti* patterns, loops, and direction of force on the stability of GQs during their unfolding pathways. The investigation was carried out under four different pulling setups with force loads ranging from ∼10^8^ to 10^12^ pN/s. We show that the unfolding pathways are dependent not only on—not unexpectedly—the GQ structure but also on the pulling protocol. Independent simulations of the same system under faster pulling lead to less diverse unfolding pathways with fewer intermediates, which is indicative of a rugged free-energy landscape. We also demonstrate that diverse unfolding intermediates in the context of the whole DNA molecule sometimes have the same or very similar end-to-end distance. It indicates that structural interpretation of experimental data based on the molecule’s end-to-end distance may be a quite complex problem.

## Materials and Methods

### Starting Structures and Simulation Setup

**Figure 2:**
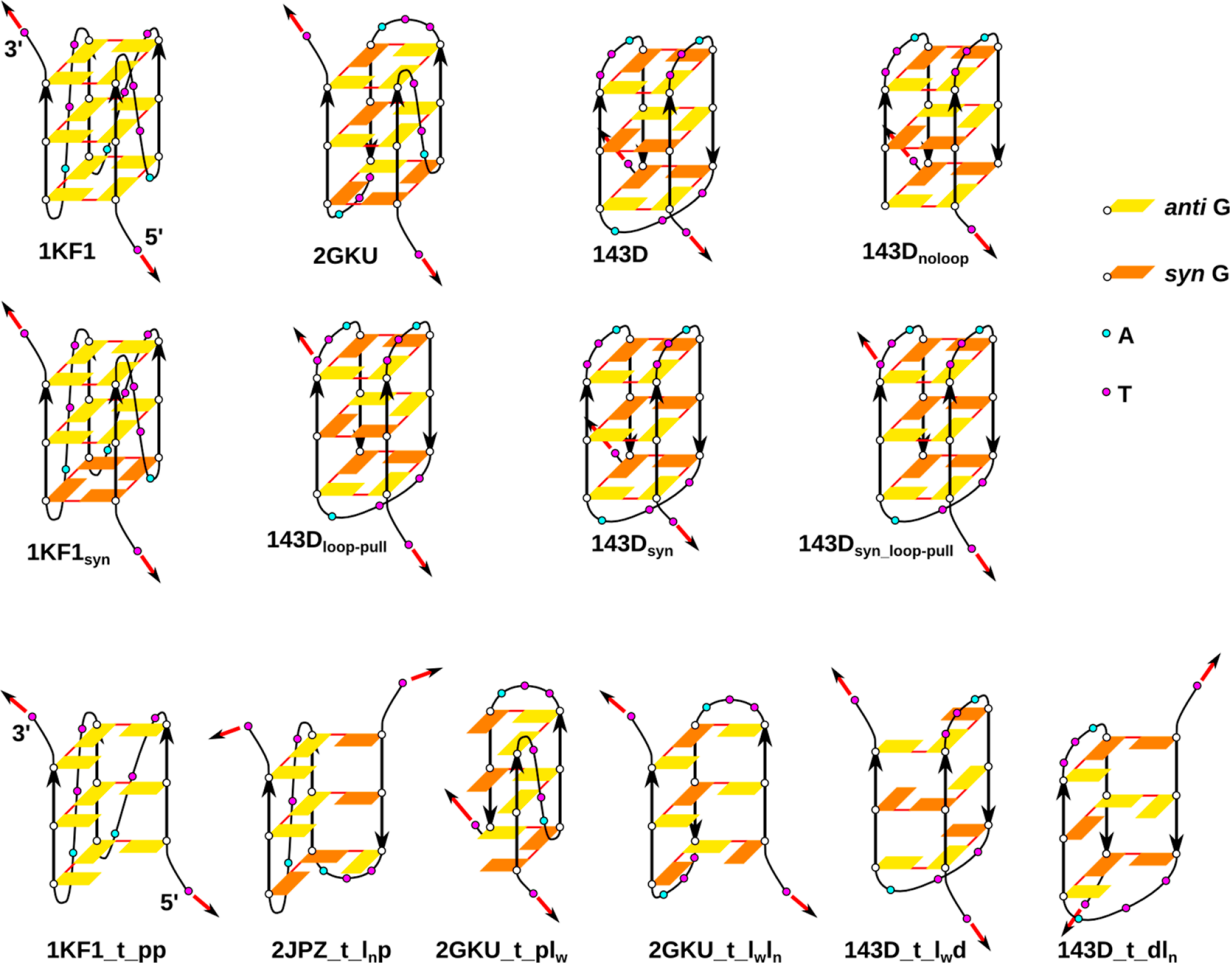
GQ and G-triplex models used in the pulling simulations. The red arrows indicate which two T’s were pulled away and the initial direction of the pulling force.

We investigated six different three-quartet GQ structures (Figure 2); three of them were native human telomeric sequence GQs: (i) parallel-stranded GQ with an all-*anti* pattern (PDB ID 1KF1(26)); (ii) 2 + 2 antiparallel GQ with an *anti*-*syn*-*anti* pattern (PDB ID 143D(22)); and (iii) 3 + 1 hybrid type-1 GQ with a *syn*-*anti*-*anti* pattern (PDB ID 2GKU(23)). G-stems with loops were taken from the PDB files and thymine nucleotides were attached at both the 5′- and 3′-ends, which resulted in the same d(TGGGTTAGGGTTAGGGTTAGGGT) sequence for the three native human telomeric GQs. The other three structures we used were modified GQs derived from the previous ones (Figure 2): (iv) antiparallel GQ 143D, where the middle diagonal loop was deleted (named as 143D_noloop_); (v) antiparallel GQ 143D with all four guanines in the middle quartet having flipped their χ dihedral angle from *anti* to *syn* orientation in two Gs and vice versa in the other two Gs, to obtain *anti–anti*–*anti* (and *syn–syn–syn*) patterns in the strands (143D_*syn*_); and (vi) parallel GQ 1KF1, with all four guanines from the first quartet [near the 5′-terminal thymine (T_1_)] having a flipped χ dihedral angle from *anti* to *syn* orientation to obtain a *syn–anti–anti* pattern (1KF1_*syn*_). We also studied five unique G-triplex structures that can be obtained by truncation of either the first or last strand and the adjacent loop from the studied GQs: 1KF1_t_pp, 2GKU_t_pl_w_, 2GKU_t_l_w_l_n_, 143D_t_l_w_d, and 143D_t_dl_n_; “t” means “G-triplex” and “p”, “d”, “l_n_” and “l_w_” stand for propeller, diagonal, lateral narrow, and lateral wide loops, respectively, present in the G-triplex; in addition, the 2JPZ_t_l_n_p triplex structure, taken from the 3 + 1 hybrid type-2 GQ (PDB ID 2JPZ(91)), was investigated (Figure 2). Coordinates of all starting structures are attached in the Supporting Information (PDB files).

The starting topologies and coordinates were prepared by using the tLEaP module of the AMBER 16 program package.^92^ Two K^+^ were manually placed inside the GQ channel if no cations were present in the PDB structure. GQ systems were built in an OL15 DNA force field,^93^ SPC/E water model,^94^ and with ∼0.15 M KCl salt using the Joung and Cheatham parameters.^95^ The 143D GQ was also simulated under ∼0.15 M NaCl salt (143D_NaCl_ label) in order to test the possible effects of the presence of different monovalent ions on GQ stability. G-triplex models were built from GQs with channel cations in the latest OL21 DNA force field,^96^ SPC/E water model, and with ∼0.15 M KCl salt.^95^ In most of the simulations, we used a rectangular periodic box with a minimum distance between box walls and solute of 30 Å, resulting in an ∼95 × 95 × 95 Å^3^ box size (∼8×10^4^ atoms in total). That setup allowed us to observe the initial stages of unfolding but did not always lead to the complete unfolding of GQ models; thus, we also performed a few simulations with a ∼150 × 150 × 150 Å^3^ box (∼3×10^5^ atoms in total; simulations labeled as “box150Å”, Supporting Information, Table S1).

All systems were subjected to equilibration and thermalization using a common protocol.^97^ AMBER topologies and coordinates were then converted into GROMACS inputs via PARMED^98^ and subsequent pulling simulations were run in GROMACS 2018^99^ in combination with PLUMED 2.5.^100^ Pulling simulations were performed at a constant temperature and pressure of 298 K and 1 atm, respectively.^101,102^ Hydrogen mass repartitioning with a 4 fs integration time step was used.^103^

### Pulling Simulation Strategies

We employed constant velocity pulling using two terminal thymine nucleotides (T_1_ and T_23_ or T_17_ for GQ or G-triplex models, respectively) and pushed them away from each other by harmonic springs. Specifically, we increased the distance between two centers of mass (COM) formed by C2, C4, and C6 atoms of pyrimidine rings of terminal T_1_ and T_23_/T_17_ from 5′- and 3′-ends, respectively. This setup was performed for each model. In addition, we used a modified pulling setup for the two antiparallel 143D and 143D_*syn*_ GQ structures and applied different directions of pulling involving COM of terminal T_1_ and T_17_ from the diagonal loop (instead of the terminal T_23_ nucleobase). These are labeled as 143D_loop-pull_ and 143D_*syn*_loop-pull_ (derived from the 143D and 143D_*syn*_ structures, respectively), and the aim was to employ the same relative direction of force with respect to the G-stem as in 1KF1 and 2GKU. In summary, we investigated forced unfolding in eight different GQ pulling models (one under two ionic conditions) and six different G-triplex models (Figure 2).

**Figure 3:**
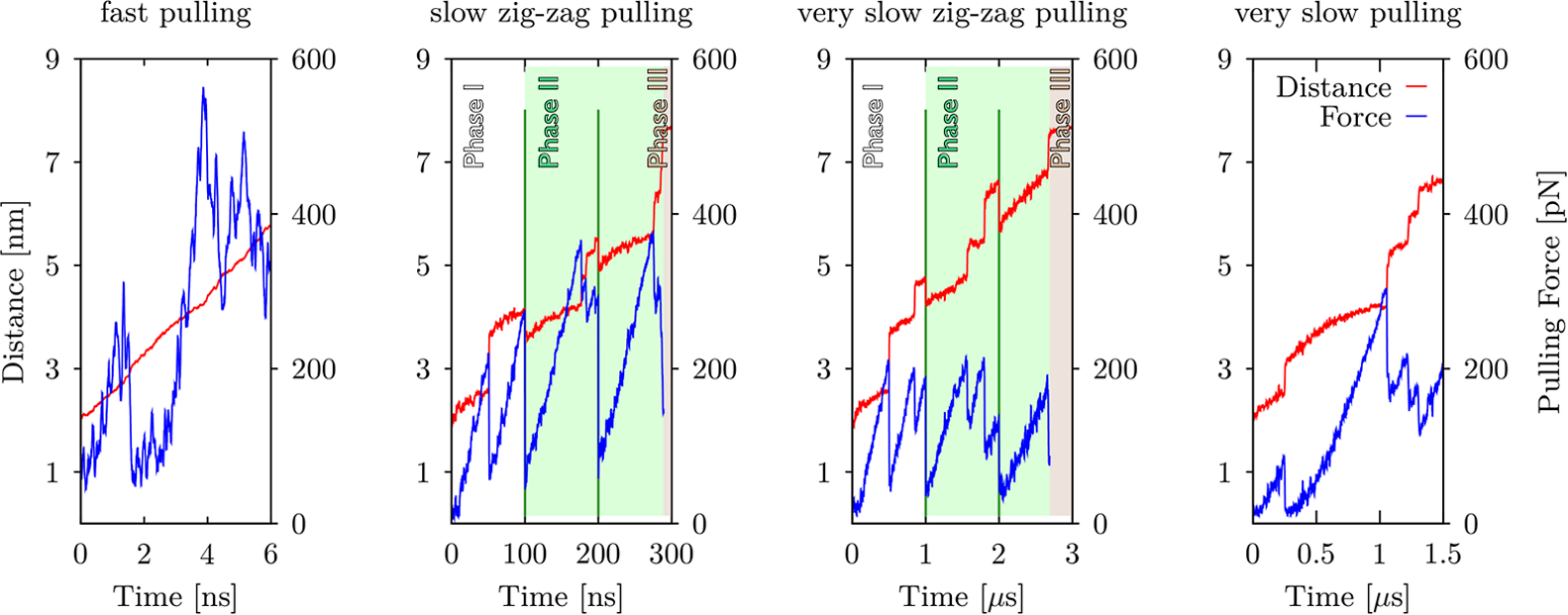
Illustration of the four applied pulling protocols as plots showing evolution of the pulling force (in blue) and end-to-end distance (in red) vs time. Protocols differ mainly by the timescale (horizontal axes), resulting in different pulling velocities (see Methods and Tables S1–S8). Zig-zag pulling protocols (plots in the middle) contain (up to) three different phases. Phase I (white area) resembles common constant velocity pulling (comparable to *fast* and *very slow pulling* protocols). Phase II (green area) is characterized by designed force drops at certain times (green vertical lines), i.e., at 100 ns, 200 ns, 1 μs, and 2 μs for *slow zig-zag pulling* and *very slow zig-zag pulling* simulations. Force drops allowed brief relaxation (often partial refolding) of systems manifested by a decrease in the end-to-end distance in the graphs. Occasionally, Phase III is reached, where systems are kept under constant maximum allowed distance between pulling centers; forces are not shown for this stage (brown area; see Materials and Methods for details). Notice that data for each plot were chosen from pulling simulations of different GQ topologies and thus, detailed side-by-side comparison of forces and distances in these plots would be misleading.

We tried out several testing runs (data not shown) to find the optimal pulling parameters with the aim to probe, especially, the initial parts of GQ unfolding. We eventually settled for four pulling setups (Figure 3): (i) *fast pulling* with a hard force constant (*k*_0_ = ∼1660 pN/nm) and a pulling velocity υ = ∼0.7 nm/ns (force load ∼10^12^ pN/s; setup comparable to the previous works by Li et al.^84^ and Bergues-Pupo et al.^85^), (ii) *slow zig-zag pulling* with a soft force constant (*k*_0_ = ∼150 pN/nm), an initial pulling velocity υ = ∼0.04 nm/ns (force load ∼6 × 10^9^ pN/s), followed by a force drop(s) and slower pulling velocity (more information below), (iii) *very slow zig-zag pulling* with a soft force constant (*k*_0_ = ∼150 pN/nm), 1 order of magnitude smaller initial pulling velocity (υ = ∼0.004 nm/ns; force load ∼6 × 10^8^ pN/s), followed by a force drop(s) and slower pulling velocity, and (iv) *very slow pulling* simulations with a soft force constant (*k*_0_ = ∼150 pN/nm) and a smaller pulling velocity (υ = ∼0.004 nm/ns; force load ∼6 × 10^8^ pN/s) without any force drop. We performed three independent runs of a given simulation setup (Supporting Information, Table S1). In total, we performed 134 independent pulling simulations.

Trajectories of GQ models were run for 6 ns and 1.5 μs for *fast pulling* and *very slow pulling*, respectively. GQ models in the big box were run for 16.5 ns and 2.75 μs for *fast pulling* and *very slow pulling*, respectively. The *very slow pulling* simulations of all six G-triplex models were run for 1 μs. *Slow zig-zag pulling* and *very slow zig-zag pulling* GQ simulations were run in a way that introduced force drops at regular intervals of 100 ns and 1 μs, respectively, and the simulations were prolonged as needed (typically twice to reach a simulation length of 300 ns and 3 μs, respectively) until a significant or complete unfolding of the molecule was reached (Figure 3, Table S1). The simulation setup before the first force drop resembles a common constant velocity pulling and is labeled as phase I, while the whole simulation course with the drops resembles the zig-zag force ramp protocols used in some recent experiments.^104^ The pulling velocity was approximately halved after the first drop. The part with decreased pulling velocity and subsequent force drops (if any) until the maximum allowed extension was reached (limitation of the simulation water box) or until the end of the simulation—whichever occurred first—is termed phase II. If the maximum allowed extension was reached before the end of the simulation, the DNA molecule was kept outstretched at the extension given by the box limit. This final phase (phase III) is characterized as a *constant end-to-end distance regime*; note that the force magnitude in this phase is meaningless. Notice that it is not possible to predict in advance the appearance of phase III for particular GQ models, i.e., if or when a given GQ would arrive at the maximum allowed extension range. A side-to-side comparison of applied pulling protocols is shown in Figure 3. Exact pulling parameters used in particular pulling simulations/phases, namely force constants κ_0_, initial (*x*_0_) and final (*x*_τ_) distances between pulling centers, simulation times (τ), corresponding pulling velocities (υ), and force loads (loading rates, *Ḟ*), are listed in the Supporting Information, Tables S2—S8.

## Results

### Unfolding Intermediates and Transitions Seen in the Simulations

In the course of the pulling simulations, we have seen a wide spectrum of intermediate structures and transitions, which are described in the text below.

**Figure 4:**
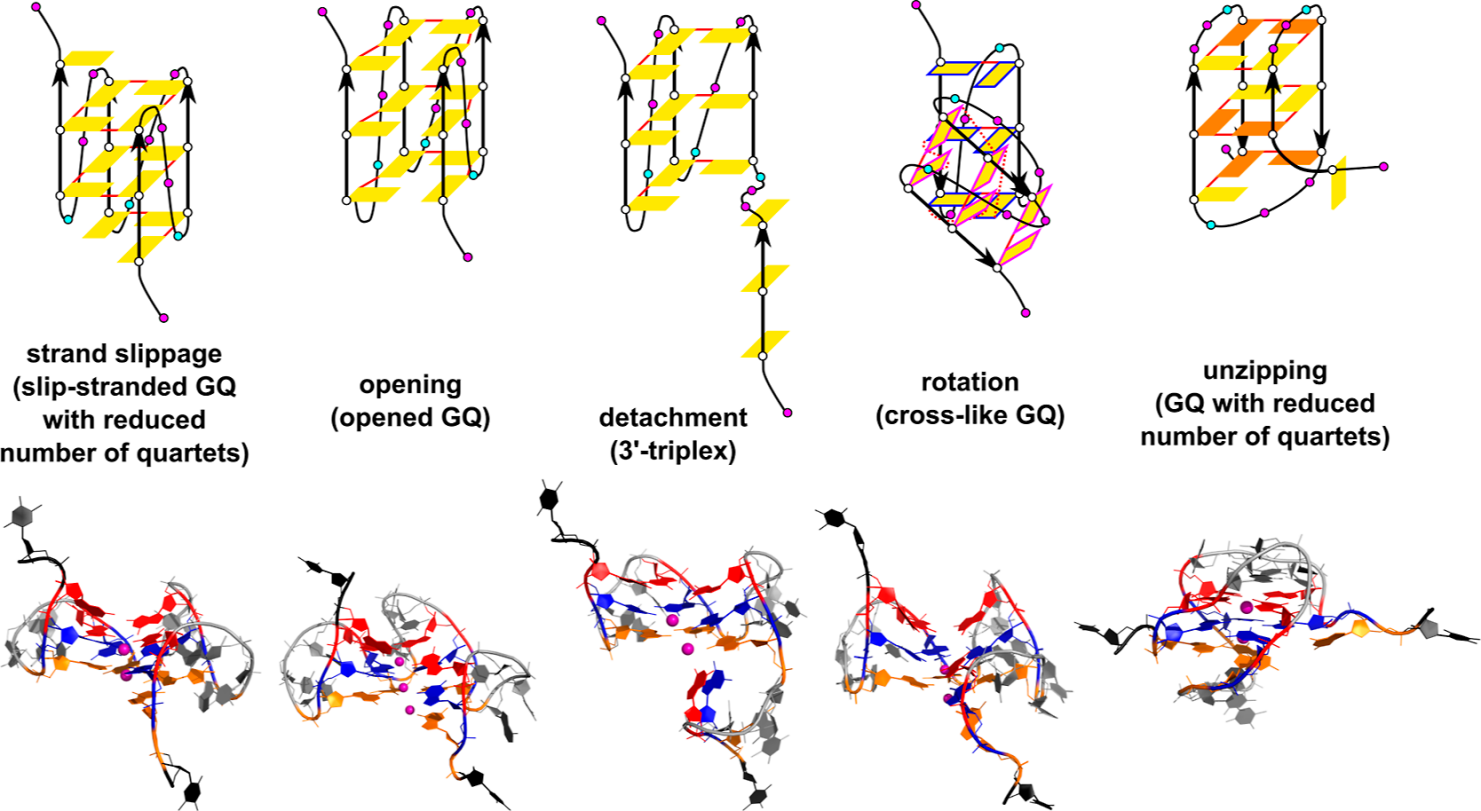
Common structural transitions and structures. The initial state of the transitions depicted in the top row is a native fully folded GQ. In principle, intermediates, such as G-triplexes, may also undergo all shown transitions (except for opening). In addition to the legend used in Figure 2, the color of the perimeter in the cross-like GQ (cross-like structure) indicates which bases are stacked together or are coplanar, solid red lines indicate cWH base pairing and the dashed lines indicate non-*c*WH base pairing. The bottom row depicts the corresponding snapshots of actual intermediates observed in pulling simulations; Gs from the first, second, and third G-quartet are highlighted in orange, blue, and red, respectively. Both terminal T residues (i.e., pulling centers) are shown in black, the remaining DNA residues are in gray, and channel K^+^ ions are shown as purple spheres.

Unfolding of GQs usually follows a few characteristic conformational transitions with formation of intermediates (Figure 4). *GQ with a reduced number of quartets* is a GQ that has fewer quartets than the native GQ. *G-triplex* is a structure which looks like a GQ, but one G-strand is detached. *5′-triplex* is a G-triplex located near the 5′-end of the sequence, while *3′-triplex* is located near the 3′-end. A *symmetric triplex* is a G-triplex structure in which the gap left after the missing fourth strand is closed by the remaining strands so that each strand is H-bonded with the other two strands using its Watson–Crick and Hoogsteen edges.^42^*G-hairpin* is a structure with only two G-strands connected by the *c*WH or related *t*WW H-bonding. *5′-hairpin* is located near the 5′-end of the sequence, and *3′-hairpin* is located near the 3′-end; in addition, the *middle hairpin* is formed by strands in the middle of the sequence. In case of 143D and its derivatives, the middle hairpin is called a *diagonal hairpin* because the strands were once connected by a diagonal loop and thus were not H-bonded together in the native GQ. *Opened GQ* is a GQ with H-bonding disrupted along one side (groove). The opening occurs between strands that are not connected by a loop (e.g., the first and last strand of 2GKU). *Cross-like GQ* (also known as *cross-like structure*) consists of two mutually rotated G-hairpins, which are held together by a network of H-bonds. *Cross-triplex* looks like a cross-like GQ with one G-strand removed. *Cross-hairpin* is just two G-strands held together by H-bonds forming a cross-shape. *Cross–cross structure* encompasses a broad ensemble of structures that are assembling three strands mutually in cross orientation.

*Strand slippage* is a vertical movement of a G-strand shifting its Gs one or more levels upward or downward with respect to the rest of the G-stem.^36,53^ It is allowed in G-stems if Gs in one strand stacked on each other have the same χ orientation. The *spiral structure* is a slip-stranded intermediate, in which the slippage is incomplete in such a way that Gs of the slipping strand is connected to the original quartet at one edge, and the other edge is H-bonded with Gs in the quartet above or below. *Opening* of GQ is a disruption of the cyclical bonding of quartets along one side of GQ, and it typically occurs between the first and last strand of GQ. An opened GQ may undergo *division*, which is its disintegration into two separated G-hairpins. *Unzipping* is a movement during which a G is pulled out of GQ. Multiple back-to-back unzipping events in a single strand may happen, which is strand unzipping; a special case of strand unzipping is *detachment*, in which all Gs of a single G-strand are unzipped abruptly (almost) at once. *Rotation* of strands is accompanied by partial breaking of native *c*WH H-bonding and potential formation of new non-native H-bonds. The above-described motions are not limited to GQs; some of them may happen during unfolding of intermediates themselves, such as G-triplexes. Representative structures of all mentioned intermediates are attached in the Supporting Information (PDB files).

Not all unfolding/rupture events can be characterized by the transitions and intermediates shown in Figure 4. Some detected events are due to unfolding of structures not directly related to the GQ core, *e.g.*, we observed a rupture of a stacked intercalated structure formed by a previously unfolded G-strand and the adjacent loop. This further underlines the enormous richness and multidimensionality of the conformational space of GQs. Major unfolding structural transitions mostly coincide with force drops and the molecule’s extension (see Supporting Information, Figures S1–S45). Sometimes the event in the graphs seems to lag behind the force drop; the reason is that the force drop may be associated with, for example, loss of two H-bonds during unzipping, but we take the time of the event when it is fully developed, *i.e.*, all four H-bonds holding the G within a quartet are disrupted.

### Antiparallel Telomeric GQ Is Toughest, Parallel Is Weakest

The highest rupture forces—and the greatest variance of their magnitude in independent simulations of a given system—were found for the *fast pulling* protocol, and the lowest forces in *very slow* and *very slow zig-zag pulling* protocols (Table 1). We consistently observed that the antiparallel basket GQ in K^+^ 143D required the highest rupture forces, followed by the hybrid GQ 2GKU, and the parallel GQ 1KF1 resisted the least. Disruption of the antiparallel basket required a lower force in Na^+^ than in K^+^ but still higher than K^+^-stabilized hybrid and parallel GQs.

**Table 1:** GQ Rupture Force Values (in pN)^a^

system^b^	*fast pulling*	*slowzig-zagpulling*	*very slowzig-zagpulling*	*very slow pulling*
1KF1	420, 420, 420, 570^c^, 450^c^, 350 + 360^c^	200, 250, 200	150 + 160, 150 + 180, 210	150 + 160, 140, 180
1KF1_syn_	450, 450, 450, 420^c^, 400 + 510^c^, 420^c^	200, 270, 270	150, 190, 190	
2GKU	430, 390, 510, 490^c^, 380^c^, 440^c^	300, 300, 380	250, 220, 220	200, 200, 200
143D	730, 650, 550, 750^c^, 560^c^, 600^c^	380, 360, 340	300, 300, 300	330, 300, 370, 310^c^, 280 + 320^c^
143D_syn_	700, 530, 580, 700 + 720^c^, 630^c^, 510 + 570^c^	340 + 340, 350 + 370, 360 + 370	200, 300, 290	
143D_noloop_	580, 580, 580, 540^c^, 570 + 640^c^, 530^c^	150 + 420, 200 + 380, 290 + 450	200 + 250, 210 + 320, 200 + 250	
143D_l__oop-pull_	440 + 800, 580 + 700, 510 + 680, 640^c^, 450^c^, 660^c^	300, 380, 380 + 410	350, 300, 250	
143D_syn_loop-pull_	510, 510, 510, 560 + 750^c^, 520^c^, 510 + 650^c^	420, 220, 410	260, 260, 270	
143D_NaCl_	410, 420 + 660, 440 + 480, 460 + 540^c^, 420 + 510^c^, 780^c^			170 + 230, 190 + 270, 210 + 320

a The values come from three independent simulations. Two values are shown (*X* + *Y*) if the highest force reached in a given simulation does not correspond to the first major GQ structural perturbation; the first value shows the force reached when the first GQ perturbation event occurred, while the second value shows the highest observed force in that simulation later on. Complete force vs time graphs for all GQ pulling simulations are shown in Figures S1–S39 followed by structural figures of important unfolding intermediates.

b See Methods and Figure 2 for details about labeling of GQ topologies.

c Simulations performed in a big ∼150 × 150 × 150 Å^3^ box.

Supporting Information, Figures S46 and S47, shows the evolution of pulling work (*W*) in both time and pulling distance, as well as their exponential average through the Jarzynski equation.^105,106^ Unfortunately, the calculations did not provide more insights than the force vs time graphs (Supporting Information, Figures S1–S39).

### Quartets of Opposite Directionality and Presence of Loops Increase Mechanical Stability

Table 1 also shows that (i) GQs containing a mixture of quartet directionalities resist the force better—regardless of the direction of the initial force, as judged from the stability order of the pairs 1KF1 < 1KF1_syn_, 143D_syn_ < 143D, and 143D_syn_loop-pull_ < 143D_loop-pull_ and (ii) the diagonal loop of the antiparallel GQ plays a stabilizing role in blocking the unzipping as shown by the pair 143D_noloop_ < 143D.

### Force Drops in the Zig-Zag Pulling May Alter Unfolding Resistance in Both Directions

**Figure 5:**
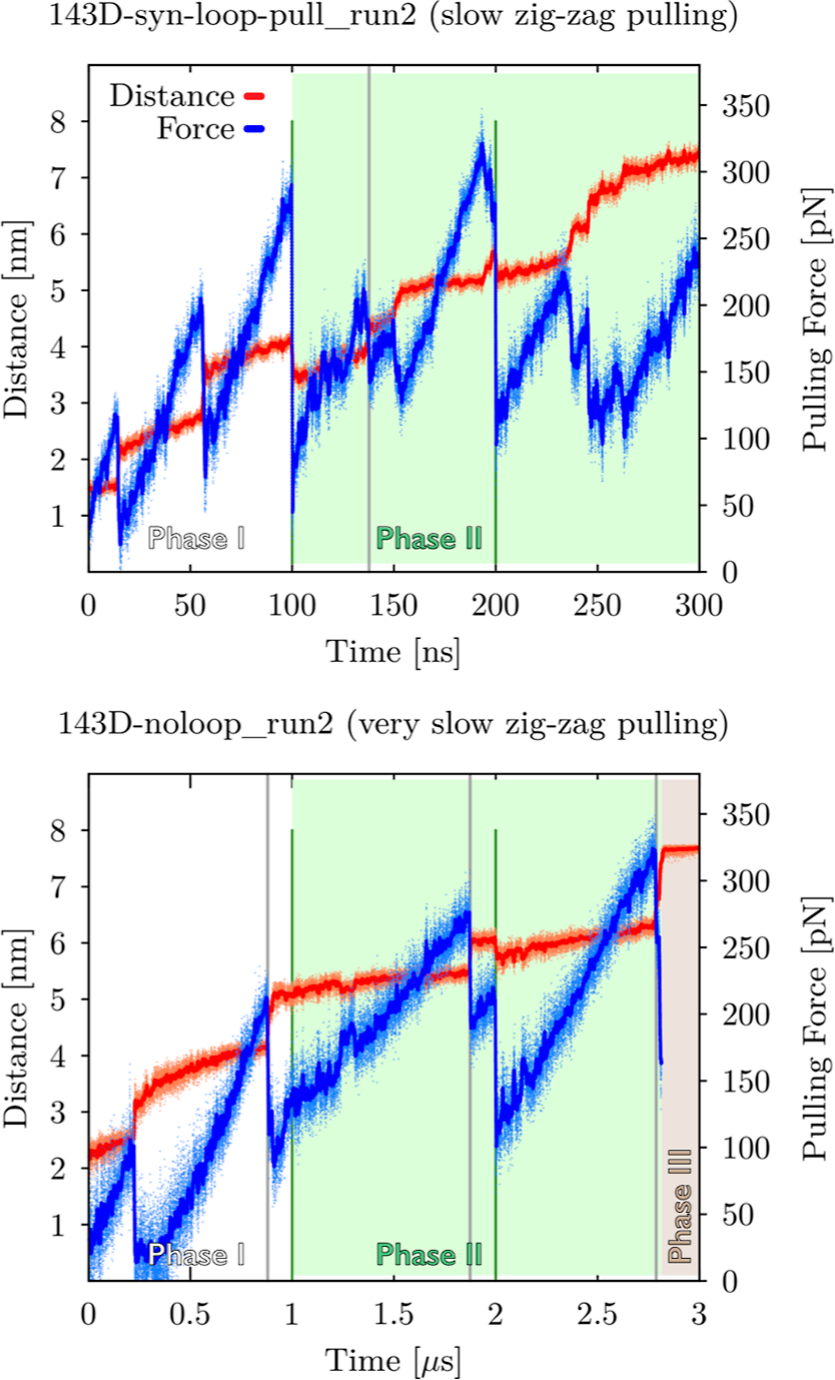
Unfolding of 143D_*syn*_loop-pull_ and 143D_noloop_ GQ models during *slow zig-zag pulling* simulation (top panel) and *very slow zig-zag pulling* simulation (bottom), respectively. The pulling phases and intended force drops are highlighted by green lines (see Figure 3 and Materials and Methods for details). The pulling force reached ∼290 pN without having any effect on the 143D_*syn*_loop-pull_ model. After the externally induced force drop at 100 ns, an unfolding event occurred at a force of just ∼220 pN (gray vertical line, top panel). The bottom panel shows an example of the second and third quartets resisting a force higher than needed to disrupt the previous quartet; unfolding of the first quartet required ∼210 pN force (leftmost gray vertical), the second (middle) quartet resisted to ∼280 pN (middle gray vertical line), and the third quartet resisted to a force up to ∼320 pN (rightmost gray vertical). Notice that the first two drops of the pulling force in the top panel and the first drop in the bottom panel correspond to unstacking of the terminal Ts, which is typical for almost all simulations.

The *very slow zig-zag pulling* simulations usually followed similar unfolding pathways as the *very slow pulling* simulations. Nevertheless, a few times in the zig-zag setup, we observed that unfolding occurred after an external (i.e., due to the zig-zag protocol) force drop at a force smaller than the peak force just before the drop (Figure 5). After the force drop, the system apparently takes advantage of the temporary relaxation and follows degrees of freedom orthogonal to the pulling collective variable to more efficiently overcome the rupture barrier.

In some (not all) simulations of the antiparallel GQ, the force needed to disrupt a second or third quartet is similar to or even higher than that needed to disrupt the first quartet (Figure 5). It suggests that the force drop allows for relaxation and settling down of the remaining quartets. In fact, this behavior is not entirely unique to the zig-zag protocol, such relaxations can be found even in standard *very slow* pulling after unzipping of a base when the end-to-end distance is suddenly prolonged.

### Two Main Structural Determinants of Initial Unfolding Mechanism—Collocation of GQ’s Termini and Its *Syn*/*Anti* Pattern

The mutual position of GQ termini translates into the force direction exerted on a given GQ (Figure 2). If both the termini are connected to the same quartet, such as in the antiparallel basket 143D, the force acts across a single quartet, i.e., it is nearly perpendicular to the GQ vertical axis, and base unzipping is the dominant initial unfolding mechanism (Table 2 and Figure 4).

**Table 2:** Main Unfolding Pathways and Intermediates Observed in GQ Pulling Simulations^a^

system	*fast pulling* (see Tables S9 and S13)	*slowzig-zagpulling* (see Table S10)	*very slowzig-zagpulling* (see Table S11)	*very slow pulling* (see Tables S12 and S14)
1KF1	strand slippage to G-triplex; rotation into cross-like GQ and its division	strand slippage leading to G-triplex; rotation into cross-like GQ and its division	mixture of strand slippage, strand detachment, base unzipping, and rotation into cross-like structures	mixture of base unzipping, strand slippage, and strand detachment
1KF1_*syn*_	rotation into cross-like GQ, GQ opening leading to G-triplex or division into G-hairpins	rotation into cross-like GQ, unfolding to a G-triplex or G-hairpin; strand unzipping to G-triplex	GQ opening and rotation into cross-like GQ, followed by complex base unzipping and strand detachments	
2GKU	GQ opening and rotation into cross-like GQ, its division into G-hairpins or detachment to cross-triplex	GQ opening and rotation into cross-like GQ, unfolding to G-triplex or G-hairpin	GQ opening and rotation into cross-like GQ, unzipping and detachments, rotation into cross-like structures	GQ opening and rotation into cross-like GQ, then a mixture of base unzipping and detachments
143D	unzipping of terminal Gs; unzipping to G-triplex followed by detachment to G-hairpin	unzipping of the first strand leading to G-triplex, then unzipping to G-hairpins or rotation to cross-like triplex	unzipping of terminal Gs to incomplete triplexes or unstable diagonal hairpins	unzipping of terminal Gs to incomplete G-triplex or diagonal hairpins
143D_*syn*_	unzipping of terminal Gs; unzipping to G-triplex followed by detachment to middle hairpin	unzipping of terminal Gs, strand unzipping to imperfect triplexes	unzipping of terminal strands, followed by detachment of adjacent strand to G-hairpin	
143D_noloop_	unzipping of the terminal strand, detachment of the adjacent strand	unzipping of the terminal strand, detachment of the adjacent strand	unzipping of terminal Gs, detachment of adjacent strand	
143D_loop-pull_	GQ opening leading to G-triplex; unzipping of strands leading to G-hairpin	complex unfolding pathways including strand unzipping, GQ opening, rotation into cross-like structures, strand detachment	base unzipping combined with GQ opening and strand detachment, 3′-hairpin remained	
143D_*syn*_loop-pull_	incomplete strand slippage leading to a spiral structure, followed by GQ opening and base unzipping to G-hairpin	complex unfolding pathways including incomplete strand slippage to spiral structures, base unzipping, GQ opening, rotation into cross-like triplexes	incomplete strand slippage to a spiral structure, then complex unfolding pathways including GQ opening, strand detachment, and unzipping	
143D_NaCl_	unzipping to G-triplex, followed by detachment to G-hairpin; unzipping of terminal Gs			unzipping of terminal Gs to incomplete G-triplex or diagonal hairpins

a Only key points mentioned here; the detailed outcome of each simulation can be found in the Supporting Information, Tables S9—S14.

If the termini are connected to the quartets at opposite ends of the GQ, the force acts across several quartets in a direction more parallel to the GQ axis, and then the *syn*/*anti* pattern of the GQ is important: (i) when a mixture of *syn* and *anti* Gs is present (2GKU), GQ opening is typically followed by rotation into cross-like GQ and (ii) in GQs with no alteration of *syn*/*anti* Gs within a G-strand, i.e., when all quartets are of the same directionality (1KF1), vertical strand slippage may occur (Figure 6). Detailed simulation outcomes for each system can be found in the Supporting Information (Supporting Results, Tables S9–S15 and Figures S1–S45).

**Figure 6:**
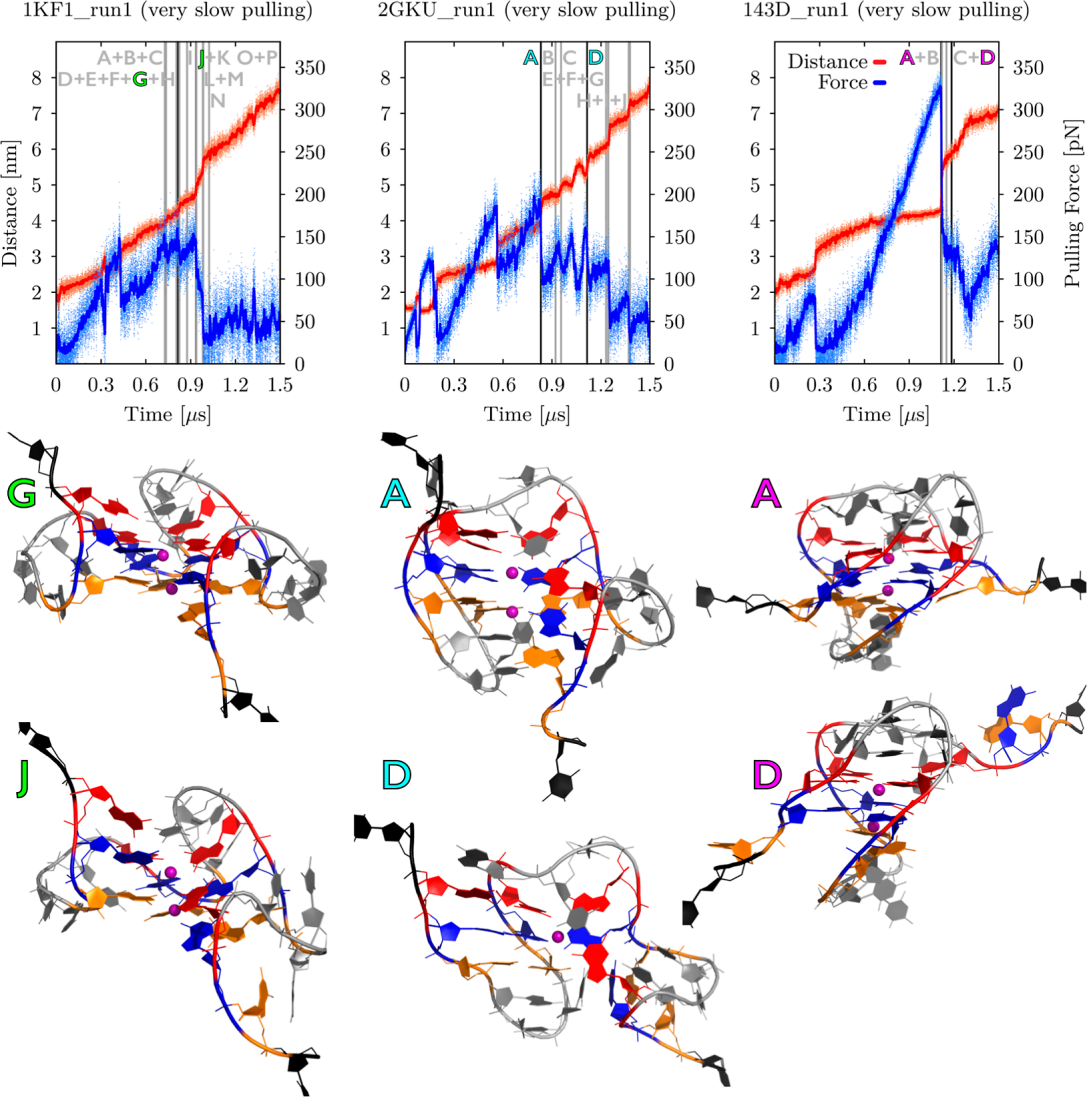
Side-by-side comparison of enforced unfolding of three main GQ systems, i.e., parallel 1KF1, hybrid 2GKU, and antiparallel 143D. Plots at the top show evolution of pulling force (in blue) and end-to-end distance (in red) vs time during *very slow pulling* simulations. It is clearly visible that parallel 1KF1 unfolded the most willingly, whereas antiparallel basket 143D GQ required significantly higher rupture forces (see Table 1 for details). Major structural events are shown (vertical lines) and labeled (capital letters). The most important pulling intermediates are highlighted (black vertical lines with letters in specific colors, i.e., green for 1KF1, cyan for 2GKU, and magenta for 143D) and shown as structural snapshots under the plots. See the legend in Figure 4 for the color scheme of the intermediate structures. See Supporting Information, Figures S25–S27, for snapshots of all the unfolding intermediates from these particular simulations and complete data from all three independent pulling simulations.

In terms of molecular interactions, the GQ stem unfolding in all tested systems was initiated by breaking of H-bonds of a G with neighboring Gs and losing its coordination with the channel cation, regardless of the unfolding mechanism.

### Richest Structural Dynamics Is under the Slowest Pulling Velocity

Comparison of the unfolding pathways between the tested pulling schemes reveals that, regardless of the pulled GQ, the *very slow pulling* leads to a sampling of a higher number of intermediate structures to reach a given unfolded state than the *fast pulling*, which is characterized by generally larger structural jumps (Supporting Information, Tables S9—S14). The pulling speed may also affect the likelihood of following a given unfolding pathway, for example, multiple-strand slippage events and strand detachments were more likely in *fast pulling*.

### Dynamics in Unfolded Ensembles—Refolding Is Possible Even with Increasing End-to-End Distance

When a molecule unfolds under force, the unfolded parts do not necessarily behave like extended rigid rods. The backbone of the unfolded part is where the strain is usually absorbed (after possible GQ rotation in space) by extending to a straighter conformation. If the unfolded part is sufficiently long and the whole molecule’s end-to-end extension is short enough, the unfolded part may undergo complex dynamics, even leading to partial refolding events; for example, GQ tearing-up into two G-hairpins may be followed by unfolding of one of the hairpins and the freed G-strand may attach to the other hairpin to form a G-triplex or cross-triplex. The refolding is facilitated by the structural memory of the molecule, and it requires enough time, so we observed it mostly in *very slow* and *very slow zig-zag pulling* conditions. Refolding attempts usually occurred after major unfolding events, which were accompanied by the relaxation of emerged structures.

### Molecules Rotate in Space upon Force Exertion

The relative direction of the force acting on a given GQ changed during the simulation, as the structure was getting unfolded. The direction of force often differed significantly from the initial direction shown in Figure 2. Because the folded part is stiffer than the unfolded part, the former naturally tended to rotate itself in space, while the already unfolded part can undergo conformational changes that relieve the stress. Thus, the greatest changes in relative force direction occurred when a loop or multiple G’s at once got ruptured. The molecule’s rotation in space to maximize the length of the folded part in the direction of the force was less likely in the *fast pulling* simulations because the unfolding was too fast and the GQ did not have enough time to fully respond.

## Discussion

We present an extended set of atomistic pulling MD simulations focused on the human telomeric GQs to better understand their unfolding process under tension and their folding landscape. Results of all 134 individual pulling simulations are fully documented in the Supporting Information, Tables S9–S15 and Figures S1–S45. The main outcome of the work is perhaps the richness of conformational space which participates in the unfolding once we apply gentler pulling. The slower the process and softer the spring, the greater structural diversity is seen. In the later stages of pulling, partial relaxations of the previously ruptured structural elements are more likely in the slower pulling. We suggest that under pulling experiments, which commonly apply even smaller pulling velocities and force loads, the GQ sequences could experience even richer dynamics than seen in our simulations.

### Impact of the Pulling Protocol on Unfolding

We have used four different pulling schemes (*fast*, *slow zig-zag*, *very slow zig-zag*, and *very slow*; see Materials and Methods). The *fast pulling* protocol with a force load of ∼10^12^ pN/s mimics the setup that was used in the previous two SMD studies on human telomeric GQs,^84,85^ and thus we can compare the previous results with our results and with the other three protocols. In general, our *fast pulling* SMD simulations have provided similar results (Tables 1 and 2) as the previous studies. Differences arise, however, when we compare these results with the other three protocols employing smaller pulling velocities and spring hardness (i.e., smaller force loads of ∼10^8^ to 10^10^ pN/s). Using these pulling protocols, rupture forces decreased by a few hundreds of pN (Table 1), and more importantly, structural dynamics with more intermediates was observed (Table 2). Although the widely recognized intermediate states, such as incomplete G-triplexes, cross-triplexes, G-hairpins, and cross-hairpins, could be reached regardless of the pulling velocity (Supporting Information, Tables S9–S15), the molecule was being shifted from equilibrium faster under higher force loads, so it had less time to respond to actual conditions and the structural transitions were often bigger “jumps”; smaller force loads enabled the molecule to undergo smaller conformational changes, relieving the stress more evenly, so a higher number of intermediates was found (and even more partial refolding events were observed). This also means that the probability to follow a given unfolding pathway may differ under different force loads.

In this sense, it is worth mentioning that none of the setups, neither the *fast*, *very slow*, nor any even slower pulling in the future, is more correct than the others. The unfolding pathways with their respective intermediates just correspond to the non-equilibrium situation introduced by the given force load.

GQs follow unfolding pathways with various structural intermediates (Figure 4), which differ for different GQ topologies and *syn/anti* patterns. Antiparallel GQs, in which the force acts within a single G-quartet, have been suggested to unfold by sequential unzipping of terminal Gs.^78,83,85^ Our current SMD simulations corroborate this idea under all unfolding conditions for 143D, 143D_NaCl_, 143D_noloop_, and 143D_syn_ models and furthermore show that the unzipping can be symmetric from both termini or asymmetric when one terminal strand unfolds more than the other; faster pulling seems to support asymmetric unzipping and vice versa (Table 2 and Supporting Information, Tables S9–S14).

The hybrid-1 GQ 2GKU unfolding was initiated by opening, followed by rotation into cross-like GQ in all tested pulling schemes, which further unfolded mostly by division into hairpins, base unzipping, or strand detachments, with slower pulling setups offering more diverse mixtures of mechanisms (Table 2 and Supporting Information, Tables S9–S13). On the contrary, previous SMD of the hybrid human telomeric GQ showed strand detachment leading to 3′-triplex,^85^ which we observed only once in slower pulling.

The impact of the pulling protocol is also manifested in the SMD of the parallel-stranded GQ 1KF1. Under the conditions of *fast pulling*, G-triplex formation^84,85^ and GQ division into two hairpins^84^ have been mentioned. Our *fast pulling* simulations also lead to G-triplex (by strand slippage in 1KF1 and by strand detachment in 1KF1_syn_) or to GQ division into G-hairpins (Table 2 and Supporting Information, Tables S9–S13). The slower pulling protocols have shown other possible—and more complex—unfolding pathways, including steps of base unzipping and strand detachment.

The importance of force direction is further shown when we applied the force on the antiparallel GQ in the direction along the groove (143D_loop-pull_ and 143D_syn_loop-pull_ models), i.e., the force acted in a similar direction as in the 1KF1 and 2GKU GQs. Terminal base unzipping was no longer so dominant, and other mechanisms, such as GQ opening, strand slippage (in the 143D_syn_loop-pull_ model), and strand detachment, emerged (Table 2 and Supporting Information, Tables S9–S13).

Another example of how applied bias may affect the unfolding pathway is the directionality of rotation into the cross-like structure. Here, we observed the formation of a prolonged propeller loop (Figure 4), which is opposite to the direction of rotation seen in unbiased simulations, as well as biased, but not using the molecule end-to-end distance acting as a collective variable.^34,36,42^ The reason is that the direction of exerted force is given by the position of strand termini and eventually the force overcame the strain in the loop. It is a nice example of how a collective variable for pulling affects exploration of the free-energy landscape and how unfolding movements orthogonal to the collective variable can be missed.

### Mechanical Stability of Human Telomeric GQs

Comparison of results from available theoretical and experimental studies of GQs is unfortunately not straightforward. SMD simulations provide detailed atomistic descriptions of the unfolding intermediates but are limited by their timescale (and thus orders of magnitude higher force loads) and unreliable statistics of forces and extensions in comparison to single-molecule force spectroscopies experiments, so the two approaches rather complement each other.

The stability of different GQ topologies of human telomeric sequences has been a matter of debate. The mechanical stability trend predicted from our current data (under all four pulling conditions) and also from the previous fast SMD simulations^85^ suggests the order antiparallel (even in Na^+^) > hybrid > parallel topology. Given that these trends take place regardless of the pulling velocity and spring hardness, one could expect the same trends to occur even under lower force loads used in the experimental measurements. Multiple experiments have suggested that the antiparallel human telomeric GQ in Na^+^ solutions is actually mechanically less stable than hybrid and parallel topologies in comparable K^+^ solutions.^65−68,70^

The contradiction might originate from multiple reasons. First off, the sheer difference in force loads of about 10 orders of magnitude might simply mean the simulation results are not transferable to current experiments (and vice versa). Given the structural polymorphism of the human telomeric sequence in conjunction with the limited structural resolution of the single-molecule techniques, the interpretation of the experiments might have been affected by the inability to directly determine which GQ topologies are present in the solution.^70,107−109^ Still, it is possible that the experiments may find some even more complex unfolding–refolding processes than we see in the SMD simulations. Obviously, we cannot rule out that the simulation force field underestimates the stability of the parallel-stranded GQ possibly because of problems in the description of propeller loops.^110^

Currently, the hope to overcome the barrier between experiments and simulations is aimed at high-speed AFM,^111,112^ which closes the gap in timescale, pulling velocity and force load between SMD simulations and experiments.^89,111^ The SMD simulations in the *very slow pulling* regime performed here (force constant *k*_0_ of ∼150 pN/nm, pulling velocity υ of ∼0.004 nm/ns, force load *Ḟ* of ∼6 × 10^8^ pN/s) already belong to the range accessible to high-speed AFM.

Mechanical stability of GQs is important in vivo when GQs are formed in ssDNA chains that are being read by proteins. Stall forces of polymerases are ∼15 pN,^79−81^ which is less than the measured rupture force of promoter GQs (∼20–55 pN depending on topology and sequence^67,68,71,74−76^); so these proteins do have difficult times when encountering a GQ, and they often need to recruit specialized helicases that resolve GQs.^6,56,58^ Our zig-zag pulling SMD simulations show a few instances of a GQ being disrupted at a lower force after the force drop in phase II than was the peak force in phase I (Figure 5). Provided that our results on telomeric GQs can be applied to promoter GQs (or at least those with canonical structure), we hypothesize that a part of GQs could eventually be resolved even by a “weaker” polymerase after repeated unwinding attempts (e.g., if the DNA slips) in a sequence of force ramps and drops.

### Mechanical Stability of G-Triplexes and Hairpins

It has been hypothesized that GQ (un)folding may proceed via G-triplex or hairpin intermediates. The existence of parallel G-triplex and hairpin remains questionable,^34,42^ but there is evidence suggesting stability of antiparallel and hybrid species coming from both experimental and computational studies.^35,38,39,41−44,46−52,70,85^ Nevertheless, the true importance of these intermediates during forced unfolding has been doubted recently, as their mechanical stability (if any) has been estimated to be below a few pN, while GQs can resist forces an order of magnitude higher.^67^ The study found no detectable formation of G-triplexes upon truncation of the human telomeric sequence to three hexanucleotide repeats. Our current data rather support the possible intermediary role of G-triplexes in unfolding of GQs. The simulations show that G-triplexes and G-hairpins are indeed formed during the mechanical unfolding (and also refolded in a few events). Mechanical stability of G-triplexes corresponds to non-negligible rupture forces, about two to five times smaller than those of GQ, even in the dedicated G-triplex pulling simulations (Supporting Information, Figures S40–S45). Still, it could be a consequence of the force loads of the SMD simulations, which could have led to steric clashes increasing the required rupture force of the G-triplex. Therefore, the role of G-triplexes in non-equilibrium unfolding may depend on the speed of unfolding and while the simulations suggest a role of G-triplexes in fast pulling, it does not imply their role in folding under equilibrium conditions. We reiterate that the pulling protocol biases the free-energy landscape by dimensionality reduction (as a collective variable), and the faster the pulling, the larger the bias. We also note that due to the kinetic partitioning of the GQ folding landscape, unexpected GQ species might form during the folding phases of some experiments, including weaker two-quartet GQs.^20,34,40^ We hypothesize that some of them could be occasionally interpreted as G-triplex states.

### Cations Binding Requires Three Interacting G-Strands

Channel cations are essential for the structural stability of GQs. A question remains of the stage at which the cations get bound to the DNA during the folding process. Our pulling simulations show that GQs keep binding the channel cations even if a whole G-strand has departed. Upon larger unfolding, if the remaining structure still contains remnants of at least G-triads or strands in the cross-like orientation, it is still able to bind one cation. This picture is corroborated by the G-triplex pulling simulations, where at least one cation occupied the channel until one strand was completely separated. At the G-hairpin stage, cations may remain bound, but when it turns into a cross-hairpin, the binding is lost, unless, rarely, a cation-binding pocket within the cross-hairpin is formed. Our previous MD simulations starting with G-triplexes showed that they do bind cations, even in the cross-triplex state;^34,42^ G-hairpins, on the other hand, displayed rather weak cation binding.^34,52^ It thus seems that the structural cations can be bound essentially anytime during the folding process. The probability is low for a hairpin-like intermediate and increases to almost certainty when three G-strands are involved.

### Determination of Structures Based on Their End-to-End Distances Is Not Unambiguous

**Figure 7:**
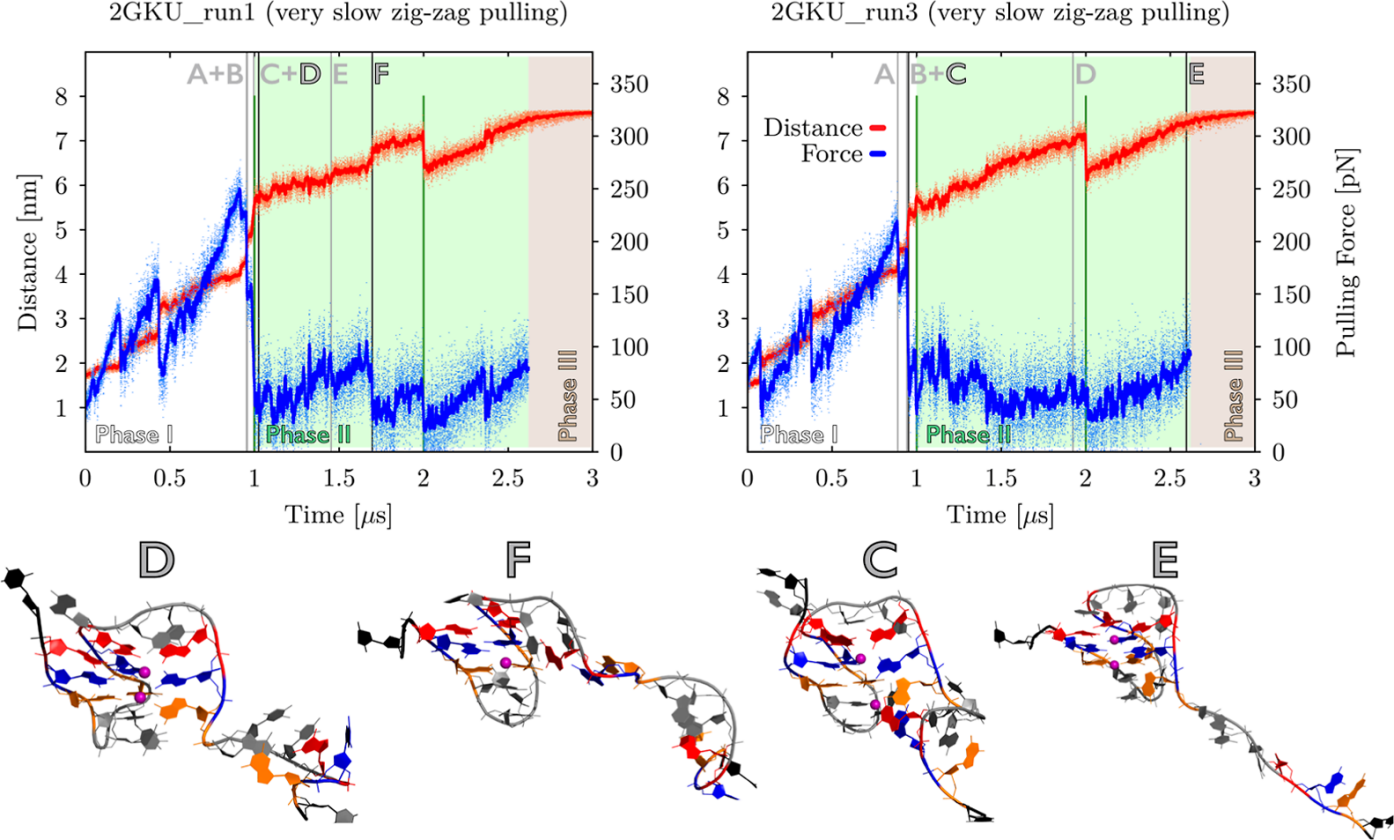
Pulling force vs. time and end-to-end distance vs. time graphs of two *very slow zig-zag pulling* simulations of the 3 + 1 hybrid 2GKU. G-hairpin (run1, letter “F”) can have a shorter end-to-end distance than G-triplex (run3, letter “E”) when taken in the context of the whole molecule. Notice that both the unfolding pathways proceeded via a very similar G-triplex state (run1, letter “D” and run3, letter “C”). Pulling phases and intended force drops in the *very slow zig-zag* protocol are highlighted (see Figure 3 and Materials and Methods for details). Important events are labeled by gray capital letters in the graphs and two key structures from each simulation (corresponding to the highlighted letters) are shown as snapshots below the graphs (see the legend of Figure 4 for details about the coloring scheme). Detailed descriptions and snapshots of all intermediates are in the Supporting Information, Table S11 and Figure S19.

Structural interpretation of single-molecule experiments is usually based on the molecule’s extension at a given moment. Typically, one measures the end-to-end distance in a GQ (or a putative G-triplex, etc.) in the structure deposited in the PDB database and adds a multiple of standard nucleotide lengths, corresponding to the tether and supposedly unfolded parts of the GQ, and seeks a match between the calculation and actual measurement. However, our simulations suggest that such a structural determination may be ambiguous in some cases; for example, we show that a G-triplex found in one simulation and a G-hairpin in another one may both have very similar end-to-end distance within the whole molecule’s context (Figure 7). While this is certainly not a rule, it demonstrates the variability of nucleic acid chains and therefore the limitations of the end-to-end calculation-based approaches.

## Conclusions

We investigated the mechanochemical properties of three human telomeric sequence GQs, three related GQs, and other six associated G-triplex sequences using SMD simulations. Four pulling setups differing in pulling velocity and force constants were tested, including two setups with the parameter values accessible to modern high-speed AFM.

We observed a few unfolding trends depending on the GQ topology: (i) the more perpendicular to the GQ channel axis was the force, the more likely was a step-wise base unzipping mechanism (typical for antiparallel GQ 143D), (ii) if the force was more parallel to the GQ channel axis and the quartet directionality was mixed (hybrid-1 GQ 2GKU), GQ opening followed by rotation into cross-like GQ was typical, and (iii) if all quartets had the same directionality (parallel GQ 1KF1), strand slippage mechanism could take place. Unfolding pathways following these initial steps were diverse, but we recurrently observed various G-triplexes, G-hairpins, and their cross-like analogues.

Our study showed that pulling parameters affect the outcome of the simulations. In general, more complex pathways with more structural intermediates and more partial refolding attempts emerged from slower pulling. It goes hand in hand with observed changes in probabilities of following a given unfolding pathway in different pulling setups. It also suggests that experiments operating at much slower pulling velocities and lower force loads could be even richer in sampling structural intermediates. Furthermore, the zig-zag pulling protocol, i.e., pulling with force drops, showed a possible way to bypass unfolding transition states high in free energy. This all is indicative of a quite rugged free-energy landscape of the telomeric sequence.

Interestingly, we observed that the GQ unfolding pathways could proceed via structurally different intermediates with almost identical overall DNA end-to-end distance, revealing possible limitations of force spectroscopy data interpretation.

The advance in experimental techniques promises that our theoretical data could be complemented by experimental studies using a similar pulling setup in the near future. It would also be worth investigating whether the same conclusions apply to various non-canonical GQ structures, which are typical for gene promoter regions.

## Data Availability

The data underlying this article will be shared on reasonable request to the corresponding author due to the large size of the raw simulation trajectories.

## Supporting Information Available

Additional methodological details, results, force and extension vs time graphs, and snapshots of intermediate structures (PDF)
Coordinates of the starting systems and representative key intermediate structures (ZIP)
