# Supplementary material for: Complexity of Guanine Quadruplex Unfolding Pathways Revealed by Atomistic Pulling Simulations
Source: J Chem Inf Model. 2023 Jul 17;63(15):4716–31. doi: 10.1021/acs.jcim.3c00171 (PMC10428220; doi:10.1021/acs.jcim.3c00171)
Supplement: Supplementary file 1 — ci3c00171_si_001.pdf [file ci3c00171_si_001.pdf]

SUPPORTING INFORMATION

Complexity of Guanine Quadruplex Unfolding Pathways Revealed by Atomistic Pulling Simulations

Petr Stadlbauer[a§], Vojtěch Mlýnský[a§], Miroslav Krepl[a] and Jiří Šponer[a]*

[a] Institute of Biophysics of the Czech Academy of Sciences, Královopolská 135, Brno, 612 00, Czech Republic

* Email: stadlbauer@ibp.cz

§ P.S. and V.M. contributed equally to this paper.

TABLE OF CONTENS

SUPPORTING RESULTS .... - 2 -
SUPPORTING TABLES .... - 5 -
SUPPORTING FIGURES .... - 19 -
REFERENCES .... - 218 -

SUPPORTING RESULTS

Unfolding dynamics. The results in the following paragraphs describe main developments seen in the simulations. Detailed outcome of all the individual simulations can be found in Tables S9–S15. The events and key intermediates are selected based on visual observation of individual trajectories, with focus on guanines and breaking/formation of their interactions in the G-stem. Detailed plots showing pulling force vs. time and end-to-end distance vs. time from all simulations with corresponding figures summarizing main structural events are shown in Figures S1-S45.

Parallel-stranded GQ. The force acting on 1KF1 was almost in the vertical direction along the groove. This, combined with the all-*anti* pattern of the GQ, facilitated vertical strand slippage, making it a common unfolding mechanism under all pulling conditions. The slipping strand usually was the first or the last one. In a few cases, triple strand-slippage led directly to 3'-triplex or 5'-triplex, respectively. The triplex could unfold further. The simulations that did not lead to G-triplex went via rotation into the cross-like GQ, which was then unfolded either by division into two unstable hairpins or by consecutive detachments of terminal strands. The richest structural dynamics during the 1KF1 unfolding was observed in *slow zig-zag* and *very slow zig-zag pulling* simulations. In *very slow pulling* simulations, we observed a few refolding attempts. Of note, the direction of rotation into cross-like GQ observed in one pulling simulation was the opposite of unbiased MD simulations; if the molecule is unrestrained, the rotation happens in the direction of the propeller loop spanning shorter distance, while in the pulling simulation the rotation happened in the direction of exerted force.

On the other hand, $1KF1_{syn}$, derived from 1KF1 by flipping its 5'-quartet to all-*syn*, did not undergo any strand slippage, which proves that the *syn-anti* strand mix sterically prohibits such movement. Instead, we observed puling-velocity-dependent scenarios. In *fast pulling* simulations, opening of the GQ was observed. Then horizontal detachment of the last G-strand could take place, *i.e.*, a cross-triplex was formed, followed by removal of the third strand, resulting in an unstable 5'-hairpin. Division of the opened GQ in the half into two separated G-hairpins also occured. In *slow zig-zag* and *very slow zig-zag pulling* simulations, the unfolding was more complicated, with multiple unfolding steps seen. Base unzipping, strand detachment and rotations into cross-like ensembles in various order were the most employed mechanisms.

3+1 hybrid-1 GQ. 2GKU felt the force in the same direction as 1KF1 and $1KF1_{syn}$ models. All the tested pulling velocity protocols led most commonly to opening of the GQ followed by formation of a cross-like GQ. In *fast pulling* box150Å simulations, the unfolding proceeded either by division into two G-hairpins or gradual detachment of strands. Under *slow zig-zag pulling*, the unfolding proceeded further from cross-like GQ to G-hairpin or G-triplex. Both *very slow zig-zag* and *very slow pulling* simulations revealed the most diverse outcomes, i.e., we identified nearly all the movement types described in the main text paragraph *Unfolding intermediates and transitions*, except for strand slippage and division. Also the terminal state in the simulations differed, i.e., we obtained unfolded chain, 3'-hairpin, cross-hairpin and incomplete 5'- or 3'-triplex (two G-triads and one GG pair). Importantly, we found out that the end-to-end distance of the molecule bearing the 3'-hairpin structure was the same as the one with incomplete 3'-triplex (Figure 7 in the main text). This observation is a reminder that structural interpretations based on just end-to-end distances measured in experiments without atomistic resolution may be ambiguous (see the main text for further details).

2+2 antiparallel GQ. Unlike in the previous systems, the force acting on 143D is directed diagonally across the 5'-quartet. Under *fast pulling* conditions, formation of 3'-triplex by strand

detachment or back-to-back unzipping was observed. Alternatively, we saw alternate unzipping of G's from the first and last strand. In the box150Å setup, the further unfolding led via complete unzipping of the first strand, followed by detachment of the second strand. In *slow zig-zag pulling* simulations, 3'-triplex was formed in all the simulations, but by unzipping of the first strand. Unfolding then proceeded in different ways, so that we obtained cross-triplex, imperfect 3'-triplex and 3'-hairpin. *Very slow zig-zag* and *very slow pulling* simulations revealed another general trend as unzipping of individual bases from the first and last strands happened in all three independent simulations. The unzipping under *very slow pulling* conditions ended up by unfolding of four or five bases from the GQ stem, so that either one quartet with two stacked diagonal G:G pairs or an incomplete 3'-triplex remained. In the box150Å simulations, we observed one unfolding via incomplete 3'-triplex followed by 3'-hairpin, and one via the structure with one quartet turning into diagonal hairpin. *Very slow zig-zag* unfolding eventually led to formation of 3'-hairpin or unstable diagonal hairpins, too. Both the *fast* and *very slow* simulations performed in Na^+ showed almost no difference in the unfolding mechanism from the K^+ simulations.

143D$_{syn}$ model had a strand *syn*/*anti* pattern allowing for strand slippage, however, the direction of the pulling force across the terminal quartet was thought not to promote this unfolding mechanism. Our expectations met with the reality, as unzipping of individual bases was the most common unfolding mechanisms yet again. Nevertheless, the system behaved similarly, but not exactly like the 143D model. In *fast pulling* simulations, formation of the 5'-triplex could also occur, followed by detachment of the first strand, so the structure tended to form a middle (diagonal) hairpin. Under *slow zig-zag pulling* simulations, base unzipping led to formation of incomplete 5'- and 3'-triplex, while the same conditions in 143D led via G-triplexes to further unfolding. *Very slow zig-zag pulling* led to extensive unfolding, 3'-hairpin was formed twice and 5'-hairpin, which further unfolded, once. Diagonal hairpin, like in the 143D model, was not found. 143D has both the first and last GpG dinucleotide step, which is disrupted by unzipping, in the same *anti*-*syn* order, while the 143D$_{syn}$ model has the first step *anti*-*anti* and the last one *syn*-*syn*. Therefore, one might expect that *syn*-*syn* step, which is thought to be less stable,[1, 2] would unzip preferentially; however, this was not the case in our simulations.

143D$_{noloop}$ simulations, *i.e.*, 143D without the diagonal loop, were designed to see if there was any effect of the diagonal loop, stacked on the terminal quartet, on the unfolding of the 143D model. In all pulling schemes, we consistently observed unzipping of individual bases from the first and last strand, which was followed by formation of triplex and hairpin intermediates. In some simulations of the 143D$_{noloop}$ model, we observed flipping of the now terminal 5'-G into the *syn* conformation; the flip happened after the major unfolding events and thus it did not affect the main results. Such *anti/syn* flips of terminal residues are common due to the fact that *syn* states are stabilized by the formation of an intramolecular H-bond between the terminal 5'-OH group and N3 nitrogen of the nucleobase.[1]

In 143D$_{loop\text{-}pull}$ simulations, the force acted on the GQ in the same relative direction as on the parallel and hybrid topologies (Figure 2). The most common outcome under *fast pulling* was GQ opening, followed by detachment of the first strand, followed by detachment of the second strand. In *slow zig-zag pulling* conditions we observed formation of a diagonal hairpin by an unzipping and/or detaching of the first and last strand, followed by unfolding of the diagonal hairpin and spontaneous refolding of 3'-hairpin. *Very slow zig-zag pulling* simulations offered the most complex unfolding dynamics including unzipping, strand detachment, cross-like GQ or G-triplex formation. Description of all those unfolding processes can be simplified as unbinding of the first strand, followed by the second strand, so that 3'-hairpin remained.

$143D_{syn\_loop\text{-}pull}$ model was under the same pulling force direction as the $143D_{loop\text{-}pull}$ model, but its modified *syn*/*anti* pattern theoretically allowed strand slippage, as in the 1KF1 model. *Fast pulling* setup provided diverse outcomes; unzipping of bases in the first and third strand, GQ opening, and incomplete strand slippage leading to spiral structures. In the box150Å simulations, the unfolding proceeded by removal of the first strand, followed by second strand, and 3'-hairpin remained. *Slow zig-zag pulling* showed a similar picture, including spiral intermediates, extended by additional unfolding. The first strand was always ultimately removed and the simulations led to partially unfolded 3'-triplexes or 3'-hairpin structures. *Very slow zig-zag pulling* protocol offered the most complex scenarios, again. Apart from GQ division, all the described unfolding motions were observed, leading to 3'-hairpins; several partial refolding events were also identified. We observed similar spiral intermediates as observed during *fast* and *slow zig-zag pulling* simulations and also events of proper strand slippage. We suspect that complete strand slippage is unlikely on this time scale (few μs), because as the distance between the connected strand ends increases during the slippage, so has to extend the lateral loop backbone, whose flexibility is limited by interaction of the loop bases with the G-stem. In comparison, the distance between connected ends during strand slippage decreases in the 1KF1 model, so the propeller loop does not need to be extended (strand slippage in the other direction, in which the distance would increase, has not been observed). In overall, $143D_{syn\_loop\text{-}pull}$ system behaved like a mixture of 1KF1 and 2GKU models.

G-triplexes. The pulling simulations were run only in the *very slow pulling* regime. Unfolding transition movements of G-triplexes were in general of similar kind as in GQ simulations. Strand slippage was common in the parallel-stranded G-triplex 1KF1_t_pp. The triplex with two lateral loops $2GKU\_t\_l_wl_n$ we observed rotation into the cross-like triplex, and then base unzipping or detachment of the strand in the cross orientation. In "hybrid" G-triplexes $2GKU\_t\_pl_w$ and $2JPZ\_t\_l_np$, *i.e.*, with one propeller and one lateral loop, unzipping of terminal G's was common. Perhaps surprisingly (when comparing with the stability of loops in 1KF1 and 1KF1_t_pp), the lateral loop unfolded before the propeller one. Both the G-triplexes with a lateral and diagonal loop, $143D\_t\_l_wd$ and $143D\_t\_dl_n$, showed that the pulling force promoted unbinding of first strand from third strand and rebinding in the order 1-2-3. In other words, the diagonal loop tended to be transformed into a lateral one. The triplet adjacent to the diagonal loop typically fell apart, however, the following transition into a G-triplex with two lateral loops was not completed, because the unfolding continued by rotation of the partially released strand into cross-orientation and its detachment. Thus, the diagonal loop fell apart before the lateral one. Not surprisingly, G-triplex rupture forces were in general smaller than those observed in corresponding full GQs; the forces reached up to 100 pN in the simulations of the parallel G-triplex 1KF1_t_pp, and the other G-triplexes they ranged mostly between 100 and 160 pN. Importantly, these values are on par with those corresponding to G-triplex unfolding events in full GQ pulling simulations.

SUPPORTING TABLES

Table S1: Overview of all performed pulling simulations.[a]

GQ System	Pulling Setup	Simulation Length [μs] [b]
1KF1		3 × 0.006
$1KF1_{syn}$		3 × 0.006
2GKU		3 × 0.006
143D		3 × 0.006
$143D_{syn}$	*fast pulling*	3 × 0.006
143_{noloop}		3 × 0.006
$143D_{loop\text{-}pull}$		3 × 0.006
$143D_{syn\_loop\text{-}pull}$		3 × 0.006
$143D_{NaCl}$		3 × 0.006
1KF1		3 × 0.2[c]
$1KF1_{syn}$		3 × 0.2[c]
2GKU		3 × 0.3[c]
143D	*slow*	3 × 0.3[c]
$143D_{syn}$	*zig-zag pulling*	3 × 0.3[c]
$143D_{noloop}$		3 × 0.3[c]
$143D_{loop\text{-}pull}$		3 × 0.3[c]
$143D_{syn\_loop\text{-}pull}$		3 × 0.3[c]
1KF1		3 × 2[d]
$1KF1_{syn}$		3 × 2[d]
2GKU		3 × 3[d]
143D	*very slow*	3 × 4[d]
$143D_{syn}$	*zig-zag pulling*	3 × 3[d]
$143D_{noloop}$		3 × 3[d]
$143D_{loop\text{-}pull}$		3 × 3[d]
$143D_{syn\_loop\text{-}pull}$		3 × 2[d]
1KF1		3 x 1.5
2GKU	*very slow pulling*	3 x 1.5
143D		3 x 1.5
$143D_{NaCl}$		3 x 1.5
$1KF1_{box150Å}$		3 × 0.0165[e]
$1KF1_{syn\text{-}box150Å}$		3 × 0.0165[e]
$2GKU_{box150Å}$		3 × 0.0165[e]
$143D_{box150Å}$		3 × 0.0165[e]
$143D_{syn\text{-}box150Å}$	*fast pulling*	3 × 0.0165[e]
$143_{noloop\text{-}box150Å}$		3 × 0.0165[e]
$143D_{loop\text{-}pull\text{-}box150Å}$		3 × 0.0165[e]
$143D_{syn\_loop\text{-}pull\text{-}box150Å}$		3 × 0.0165[e]
$143D_{NaCl\text{-}box150Å}$		3 × 0.0165[e]
$143D_{box150Å}$	*very slow pulling*	2 x 2.75[e,f]
**G-triplex System**	**Pulling Setup**	**Simulation Length [μs] [b]**
1KF1_t_pp		3 x 1
2JPZ_t_l_np		3 x 1
2GKU_t_pl_w	*very slow pulling*	3 x 1
2GKU_t_$l_w l_n$		3 x 1
143D_t_l_wd		3 x 1
143d_t_dl_n		3 x 1

[a] see Methods in main text and Tables S1-S8 for details about each system and pulling setups. Pulling directions for each model are visualized in Figure 2 in main text.

[b] "*n* × *t*" means that we performed *n* independent simulations of a system, each *t* μs long.

[c] force drops introduced every 0.1 μs

[d] force drops introduced every 1 μs

[e] GQ simulations with a big ~150×150×150 Å^3 box (box150Å) allowing complete unfolding

[f] only two independent simulations were performed for the $143D_{box150Å}$ model under the *very slow pulling* conditions due to enormous computational costs

Table S2: Detailed pulling parameters for *fast pulling* simulations of nine GQ models.[a]

System	κ_0 [pN/nm]	x_0 [nm]	x_τ [nm]	τ [ns]	υ [nm/ns]	$\dot{F}$ [pN/s]
1KF1	1660	2.0	6.0	6	0.667	$1.1\cdot10^{12}$
$1KF1_{syn}$	1660	2.0	6.0	6	0.667	$1.1\cdot10^{12}$
2GKU	1660	2.0	6.0	6	0.667	$1.1\cdot10^{12}$
143D	1660	2.0	6.0	6	0.667	$1.1\cdot10^{12}$
$143D_{syn}$	1660	2.0	6.0	6	0.667	$1.1\cdot10^{12}$
$143D_{noloop}$	1660	2.4	6.0	6	0.600	$1.0\cdot10^{12}$
$143D_{loop\text{-}pull}$	1660	2.1	6.0	6	0.650	$1.1\cdot10^{12}$
$143D_{syn\_loop\text{-}pull}$	1660	1.7	6.0	6	0.717	$1.2\cdot10^{12}$
$143D_{NaCl}$	1660	2.1	6.0	6	0.650	$1.1\cdot10^{12}$

[a] We ran three independent simulations (identical pulling settings) for each GQ model characterized by force constant (κ_0), initial (x_0) and final (x_τ) distances between pulling centers, simulation timescale (τ), corresponding pulling velocity (υ) and loading rate ($\dot{F}$).

Table S3: Detailed pulling parameters for *slow zig-zag pulling* simulations of eight GQ models.[a]

	Phase I						Phase II					
System	κ_0 [pN/nm]	x_0 [nm]	x_τ [nm]	τ [ns]	υ [nm/ns]	$\dot{F}$ [pN/s]	κ_0 [pN/nm]	x_0 [nm]	x_τ [nm]	τ [ns]	υ [nm/ns]	$\dot{F}$ [pN/s]
1KF1	150	2.0	6.0	100	0.040	$6.0\cdot10^{9}$	150	6.0	7.5	100	0.015	$2.3\cdot10^{9}$
$1KF1_{syn}$	150	2.0	6.0	100	0.040	$6.0\cdot10^{9}$	150	6.0	7.5	100	0.015	$2.3\cdot10^{9}$
2GKU	150	2.0	6.0	100	0.040	$6.0\cdot10^{9}$	150	6.0	9.0	200	0.015	$2.3\cdot10^{9}$
143D	150	2.0	6.0	100	0.040	$6.0\cdot10^{9}$	150	6.0	9.0	200	0.015	$2.3\cdot10^{9}$
$143D_{syn}$	150	2.0	6.0	100	0.040	$6.0\cdot10^{9}$	150	4.0	9.0	200	0.025	$3.8\cdot10^{9}$
$143D_{noloop}$	150	2.4	6.0	100	0.036	$5.4\cdot10^{9}$	150	6.0	9.0	200	0.015	$2.3\cdot10^{9}$
$143D_{loop\text{-}pull}$	150	2.1	6.0	100	0.039	$5.9\cdot10^{9}$	150	6.0	9.0	200	0.015	$2.3\cdot10^{9}$
$143D_{syn\_loop\text{-}pull}$	150	1.7	6.0	100	0.043	$6.5\cdot10^{9}$	150	4.0	9.0	200	0.025	$3.8\cdot10^{9}$

[a] We ran three independent simulations (identical pulling settings) for each GQ model. Pulling Phases I and II (see Methods in main text) are characterized by force constant (κ_0), initial (x_0) and final (x_τ) distances between pulling centers, simulation timescale (τ), corresponding pulling velocity (υ) and loading rate ($\dot{F}$).

Table S4: Detailed pulling parameters for *very slow zig-zag pulling* simulations of eight GQ models.[a]

	Phase I						Phase II					
System	κ_0 [pN/nm]	x_0 [nm]	x_τ [nm]	τ [ns]	υ [nm/ns]	$\dot{F}$ [pN/s]	κ_0 [pN/nm]	x_0 [nm]	x_τ [nm]	τ [ns]	υ [nm/ns]	$\dot{F}$ [pN/s]
1KF1	150	2.0	6.0	1000	0.004	$6.0\cdot10^{8}$	150	6.0	7.5	1000	0.002	$3.0\cdot10^{8}$
$1KF1_{syn}$	150	2.0	6.0	1000	0.004	$6.0\cdot10^{8}$	150	6.0	7.5	1000	0.002	$3.0\cdot10^{8}$
2GKU	150	2.0	6.0	1000	0.004	$6.0\cdot10^{8}$	150	6.0	9.0	2000	0.002	$3.0\cdot10^{8}$
143D	150	2.0	6.0	1000	0.004	$6.0\cdot10^{8}$	150	6.0	9.0	3000	0.001	$1.5\cdot10^{8}$
$143D_{syn}$	150	2.0	6.0	1000	0.004	$6.0\cdot10^{8}$	150	4.5	9.0	2000	0.002	$3.0\cdot10^{8}$
$143D_{noloop}$	150	2.4	6.0	1000	0.004	$6.0\cdot10^{8}$	150	6.0	9.0	2000	0.002	$3.0\cdot10^{8}$
$143D_{loop\text{-}pull}$	150	2.1	6.0	1000	0.004	$6.0\cdot10^{8}$	150	6.0	9.0	2000	0.002	$3.0\cdot10^{8}$
$143D_{syn\_loop\text{-}pull}$	150	1.7	6.0	1000	0.004	$6.0\cdot10^{8}$	150	4.5	7.5	1000	0.003	$4.5\cdot10^{8}$

[a] We ran three independent simulations (identical pulling settings) for each GQ model. Pulling Phases I and II are characterized by force constant (κ_0), initial (x_0) and final (x_τ) distances between pulling centers, simulation timescale (τ), corresponding pulling velocity (υ) and loading rate ($\dot{F}$).

Table S5: Detailed pulling parameters for *very slow pulling* simulations of four GQ models.[a]

System	κ_0 [pN/nm]	x_0 [nm]	x_τ [nm]	τ [ns]	υ [nm/ns]	$\dot{F}$ [pN/s]
1KF1	150	1.9	8.0	1500	0.004	$6.0\cdot10^{8}$
2GKU	150	1.7	8.0	1500	0.004	$6.0\cdot10^{8}$
143D	150	2.1	8.0	1500	0.004	$6.0\cdot10^{8}$
$143D_{NaCl}$	150	2.1	8.0	1500	0.004	$6.0\cdot10^{8}$

[a] We ran three independent simulations (identical pulling settings) for 1KF1, 2GKU, 143D, and $143D_{NaCl}$ GQ models. Simulations are characterized by force constant (κ_0), initial (x_0) and final (x_τ) distances between pulling centers, simulation timescale (τ), corresponding pulling velocity (υ) and loading rate ($\dot{F}$).

Table S6: Detailed pulling parameters for *fast pulling* box150Å simulations of nine GQ models.[a]

System	κ_0 [pN/nm]	x_0 [nm]	x_τ [nm]	τ [ns]	υ [nm/ns]	$\dot{F}$ [pN/s]
$1KF1_{box150Å}$	1660	1.9	13.0	16.5	0.673	$1.1{\cdot}10^{12}$
$1KF1_{\mathit{syn}\text{-}box150Å}$	1660	1.9	13.0	16.5	0.673	$1.1{\cdot}10^{12}$
$2GKU_{box150Å}$	1660	1.9	13.0	16.5	0.673	$1.1{\cdot}10^{12}$
$143D_{box150Å}$	1660	2.1	13.0	16.5	0.661	$1.1{\cdot}10^{12}$
$143D_{\mathit{syn}\text{-}box150Å}$	1660	2.1	13.0	16.5	0.661	$1.1{\cdot}10^{12}$
$143D_{noloop=box150Å}$	1660	2.1	13.0	16.5	0.661	$1.1{\cdot}10^{12}$
$143D_{loop\text{-}pull\text{-}box150Å}$	1660	1.9	13.0	16.5	0.673	$1.1{\cdot}10^{12}$
$143D_{\mathit{syn}\_loop\text{-}pull\text{-}box150Å}$	1660	1.9	13.0	16.5	0.773	$1.3{\cdot}10^{12}$
$143D_{NaCl\text{-}box150Å}$	1660	2.1	13.0	16.5	0.661	$1.1{\cdot}10^{12}$

[a] We ran three independent simulations (identical pulling settings) for each GQ model characterized by force constant (κ_0), initial (x_0) and final (x_τ) distances between pulling centers, simulation timescale (τ), corresponding pulling velocity (υ) and loading rate ($\dot{F}$).

Table S7: Detailed pulling parameters for *very slow pulling* box150Å simulations of the 143D GQ model.[a]

System	κ_0 [pN/nm]	x_0 [nm]	x_τ [nm]	τ [ns]	υ [nm/ns]	$\dot{F}$ [pN/s]
$143D_{box150Å}$	150	2.1	13.0	2750	0.004	$6.0{\cdot}10^{8}$

[a] Simulation is characterized by force constant (κ_0), initial (x_0) and final (x_τ) distances between pulling centers, simulation timescale (τ), corresponding pulling velocity (υ) and loading rate ($\dot{F}$).

Table S8: Detailed pulling parameters for *very slow pulling* simulations of six G-triplex models.[a]

System	κ_0 [pN/nm]	x_0 [nm]	x_τ [nm]	τ [ns]	υ [nm/ns]	$\dot{F}$ [pN/s]
$1KF1_{t\text{-}pp}$	150	2.4	8.0	1000	0.006	$9.0{\cdot}10^{8}$
$2GKU_{t\text{-}ll}$	150	1.7	8.0	1000	0.006	$9.0{\cdot}10^{8}$
$2GKU_{t\text{-}pl}$	150	1.2	8.0	1000	0.007	$1.1{\cdot}10^{9}$
$143D_{t\text{-}dl}$	150	1.8	8.0	1000	0.006	$9.0{\cdot}10^{8}$
$143D_{t\text{-}ld}$	150	1.6	8.0	1000	0.006	$9.0{\cdot}10^{8}$
$2JPZ_{t\text{-}lp}$	150	1.0	8.0	1000	0.007	$1.1{\cdot}10^{9}$

[a] We ran three independent simulations (identical pulling settings) of each G-triplex model. Simulations are characterized by force constant (κ_0), initial (x_0) and final (x_τ) distances between pulling centers, simulation timescale (τ), corresponding pulling velocity (υ) and loading rate ($\dot{F}$).

Table S9: Outcome of simulations with the *fast pulling* setup.[a]

System	Outcome	Figures
1KF1	run1: first strand slipped three levels in the 5'-direction (A) leading to 3'-triplex	S1B
	run2: spiral structure formed and GQ opened (A), last G unzipped (B), last strand slipped in the 3'-direction (C), first strand slipped in the 5'-direction (D), third and fourth strand slipped in the 3'-direction two levels (E), 5'-hairpin unfolded (F)	S1C
	run3: last strand slipped three levels in the 3'-direction (A) leading to 5'-triplex	S1B
$1KF1_{syn}$	run1: GQ opening and formation of cross-like GQ (A), last G unzipped (B)	S2B
	run2: GQ opening and formation of cross-like GQ (A), last G unzipped (B), last strand detached (C), so that 5'-cross-triplex remained	S2B
	run3: GQ opening and formation of cross-like GQ (A), last G unzipped (B), last strand detached (C), and 5'-triplex refolded	S2C
2GKU	run1: GQ opening and formation of cross-like GQ (A)	S3B
	run2: GQ opening and formation of cross-like GQ (A)	S3B
	run3: GQ opening and formation of cross-like GQ (A), last (B) and first G unzipped (C)	S3B
143D	run1: first (A) and second G unzipped (B)	S4B
	run2: first (A) and second G unzipped (B)	S4B
	run3: first (A) and last G unzipped (B)	S4B
$143D_{syn}$	run1: first (A) and last (B) G unzipped	S5B
	run2: first (A), and second G unzipped (B)	S5B
	run3: first (A), and second G unzipped (B)	S5B
$143D_{noloop}$	run1: first (A) and last G unzipped (B)	S6B
	run2: first and last G unzipped (A)	S6B
	run3: first (A) and last G unzipped (B)	S6B
$143D_{loop\text{-}pull}$	run1: first G unzipped (A), GQ opening (B)	S7B
	run2 first G unzipped (A), GQ opening (B), first strand rotated into cross-orientation (C)	S7B
	run3: first G unzipped (A), GQ opening (B), last G of third strand unzipped (C)	S7C
$143D_{syn\_loop\text{-}pull}$	run1: GQ opening (A), first (B) and last G of the third strand unzipped (C)	S8B
	run2: first strand slipped in the 5'-driection to form spiral structure (A), last G of third strand unzipped (B), GQ opening (C), first G unzipped (D)	S8C
	run3: GQ opening (A), first strand rotated into cross-orientation (B)	S8C

143D$_{NaCl}$	run1: first G (A) and second G unzipped (B)	S28B
	run2: first G unzipped (A), last and second to last G's unbound from third and second G of second strand (B)	S28B
	run3: first G (A) and second G unzipped (B)	S28B

[a] The capital letters (A, B, C…) denote the events and structural snapshots in the respective Supporting Figures.

Table S10: Outcome of simulations with the *slow zig-zag pulling* setup.[a]

System	Outcome	Figures
1KF1	run1: first strand rotated into cross-orientation to the last strand (A), then triple strand slippage of the first strand in the 5'-direction (B), so that 3'-hairpin remained	S9B
	run2: spiral structure formed (A), last G unzipped (B), GQ opening and rotation into cross-like GQ (C), triple strand slippage of the last strand in the 3'-direction, (**II**) the other strands reformed 5'-triplex (D), last G of third strand unzipped (E)	S9C
	run3: third and last strand slipped in the 3'-direction (A), last strand rotated into cross-orientation to the first strand (B), last strand slipped in the 3'-direction (C), first G unzipped (D), third strand detached from second strand (E), so that 5'-hairpin and 3'-cross-hairpin are formed, (**II**) the latter unfolds by strand detachment (F), the former eventually turns into cross-hairpin	S9D
$1KF1_{syn}$	run1: GQ opening and rotation into cross-like GQ (A), last (B), (**II**) first (C), second to last (D) and third to last G unzipped (E), 5'-triplex with two triplets refolded	S10B
	run2: spiral structure formed (A), (**II**) last G unzipped (B), GQ opening and rotation into cross-like GQ (C), first G unzipped (D), first (E) and last strand detached (F), second G reattached (G), middle hairpin turned into cross-hairpin (H), third G reattached (I), third strand detached (J)	S10C
	run3: (**II**) first G unzipped (A), GQ opening and rotation of first strand (with first G of second strand stacked onto second G) into cross-like orientation (B), first strand detached (C), so that 3'-triplex with two triplets remained	S10D
2GKU	run1: (**II**) GQ opening and rotation into cross-like GQ (A), first (B) and last G unzipped (C), division into two G-hairpins (D), 5'-hairpin unfolded (E), perturbed 3'-hairpin remained	S11B
	run2: (**II**) last G unzipped (A), GQ opening and rotation into cross-like GQ (B), second to last G unzipped and 5'-triplex with one quartet refolded (C), third to last G unzipped (D)	S11C
	run3: GQ opening and rotation into cross-like GQ (A), (**II**) first G unzipped (B), structure divided into two hairpins (C), 5'-hairpin unfolded (D), so that 3'-hairpin remained	S11D
143D	run1: (**II**) first (A), second (B) and third G unzipped (C), then rotation of second strand, resulting into cross-like triplex (D)	S12B
	run2: (**II**) first (A), second (B), last (C) and third G unzipped (D), so that 3'-triplex with two triplets remained	S12C
	run3: (**II**) first (A), second (B), third (C) and (**III**) last G unzipped (D), second strand rotated to form a cross-triplex (E), then detached (F), resulting into imperfect 3'-hairpin	S12D
$143D_{syn}$	run1: (**II**) first (A), last (B), second (C), and second to last G unzipped (D)	S13B
	run2: (**II**) last (A), first (B), second (C), and third G unzipped (D), second strand slipped in 5'-direction (E), resulting into imperfect 3'-G-triplex	S13C
	run3: (**II**) first (A), last (B), second to last (C), and third to last G unzipped (D), resulting into imperfect 5'-G-triplex	S13D
$143D_{noloop}$	run1: first (A), last (B), second (C) and (**II**) third G unzipped (D), then second strand detached, migrated to third strand (E) and rotated to form a cross-like 3'-	S14B

	triplex (F)	
	run2: first (A), last (B), second (C) and (**II**) third G unzipped (D), then second strand detached (E), so the structure was separated into parts, 3'-hairpin remained	S14C
	run3: (**II**) first (A), last (B) and second to last G unzipped (C)	S14D
$143D_{loop\text{-}pull}$	run1: last G of third strand unzipped (A),* GQ opening (B), first strand rotated into cross position (C), second G of third strand unzipped (D), first and second G detached (E), (**II**) second strand rotated into cross position (F), third G unzipped (G), second strand detached (H), 3'-hairpin refolded (I)	S15B
	run2: (**II**) last G of third strand (A) and second G of third strand unzipped (B), first and second G detached (C), third G unzipped (D), second strand detached (E), 3'-hairpin refolded (F)	S15C
	run3: (**II**) first (A), second (B), and third G unzipped (C), then gradual opening between third and fourth strand, third strand rotated into cross orientation (D), then last G of third strand unzipped (E) and first and second G of third strand detached (F), leaving middle hairpin, which turned into cross-hairpin (G), third strand eventually gradually bound to fourth strand in cross orientation, so that cross-cross structure was formed	S15D
$143D_{syn\_loop\text{-}pull}$	run1: (**II**) first and second G detached (A), third G rotated into cross orientation (B), last G of third strand (C) and third G unzipped (D), then last G of third strand incorporated back into 3'-triplex (E), and unzipped again (F), (**III**) first G of second strand unzipped (G), so that 3'-triplex with two triads remained	S16B
	run2: (**II**) spiral structure formed (A), first G unzipped (B), second and third G detached (C), then third strand (only) slipped in the 3'-direction and rotated (D), so 3'-cross-triplex was formed	S16C
	run3: (**II**) spiral structure formed (A), first G unzipped (B), GQ opening (C), last G of third strand unzipped and GQ closed the opening (D), second and third G detached (E), last G of third strand incorporated back into 3'-triplex (F), second strand rotates to form 3'-cross-triplex (G)	S16D

[a] The capital letters (A, B, C…) denote the events and structural snapshots in the respective Supporting Figures. Roman numerals II and III denote events that happened after the first external force drop, i.e. in phase II, and after reaching maximum allowed extension, i.e. phase III, respectively.
*T1 unzipped after the marked unfolding event

Table S11: Outcome of simulations with the *very slow zig-zag pulling* setup.[a]

System	Outcome	Figures
1KF1	run1: last G unzipped (A), the last strand slipped in the 3'-direction (B) and got detached (C), (**II**) the resultant G-triplex turned into a cross-triplex by rotation of the third strand (D), the first strand gradually unzipped (E), the remaining cross-hairpin unfolded (F)	S17B
	run2: last strand slipped in the 3'-direction (A), first strand slipped in the 5'-direction (B), last strand unzipped (C), (**II**) the resultant G-triplex turned into cross-triplex by rotation of first strand (D), then briefly turned into symmetric triplex (E), cross-triplex was formed by rotation of second strand (F), reformation of G-triplex (G) was followed by strand slippage of the third strand in the 3'-direction (H), then cross-triplex reappeared by rotation of first strand (I), which was then detached (J), the remaining middle hairpin unfolded via cross-hairpin (K)	S17C
	run3: last G unzipped (A), followed by incomplete strand slippage resulting into the spiral structure (B), first G unzipped (C), the last strand completed the slippage in the 3'-direction (D) and (**II**) then detached (E), 5'-G-triplex unfolded and refolded several time by back-and-forth detaching and reattaching first strand (without first G), eventually whole third strand unzipped (F), the resultant 5'-G-hairpin unfolded by strand slippage (G)	S17D
$1KF1_{syn}$	run1: first G unzipped (A), GQ opening and rotation into cross-like GQ (B), (**II**) last strand gradually unzipped and cross-triplex formed (C), first strand and first G of second strand detached (first G of second strand was stacked onto second and third G of first strand) (D), remaining middle G-hairpin eventually unfolded (E)	S18B
	run2: first (A) and last G unzipped (B), cross-like GQ formed (C), (**II**) second to last G unzipped (D), first strand and first G of second strand detached (E), third to last G unzipped (F), resultant middle G-hairpin unfolded (G)	S18C
	run3: first G unzipped (A), GQ opening and rotation into cross-like GQ (B) first strand and first G of second strand detached, second strand rotated back to form G-triplex (C), (**II**) first G of the second strand unzipped, last strand gradually unzipped (D), resultant middle G-hairpin H-bonded with last G of the first strand	S18D
2GKU	run1: GQ opening (A), last G unzipped (B), first strand gradually unzipped (C), (**II**) last G incorporated back into the 3'-G-triplex (D), which later turned into a cross-triplex by rotation of second strand (E), eventually second strand detached (F) and 3'-G-hairpin remained	S19B
	run2: first G unzipped (A), GQ opening and rotation into cross-like GQ (B), (**II**) first strand unzipped and cross-triplex formed (C), last two Gs unzipped (D), middle cross-hairpin with first G of the last strand remained	S19C
	run3: first G unzipped (A), GQ opening and rotation into cross-like GQ (B), first strand detached and 3'-triplex remained (C), then (**II**) back-and-forth transitions to cross-like triplex (D), first G of the second strand unzipped (E)	S19D
143D	run1: (**II**) first (A) and second G unzipped (B), last (C) and second to last G unzipped (D), third G unzipped (E), resultant diagonal hairpin gradually unfolded	S20B
	run2: (**II**) first and last G unzipped (A), second G unzipped (B), third G unzipped (C), then second strand slipped by one level in the 5'-direction to form a misfolded G-triplex (*t*WH pairing, D) and eventually detached (E), so that 3'-G-hairpin remained	S20C
	run3: (**II**) first (A) and last G unzipped (B), second G unzipped (C), second to last G unzipped (D), third G unzipped (E), remaining structure gradually unfolded	S20D

$143D_{syn}$	run1: first (A), (**II**) second (B) and third G unzipped (C), then second strand detached (D), so that 3'-hairpin remained	S21B
	run2: (**II**) last (A), first (B), second (C) and third G unzipped (D), second strand detached (E), then bound to third strand to form a cross-triplex (F) with brief visits of G-triplex, (**III**) eventually second strand detached (G), so that incomplete 3'-hairpin remained	S21C
	run3: (**II**) first (A), last (B), second to last (C), third to last G unzipped (D), third strand detached (E), resultant 5'-hairpin unfolded	S21D
$143D_{noloop}$	run1: first and last G unzipped (A), (**II**) second G unzipped (B), third G unzipped (C), symmetric triplex formed (D), then second strand unbound from last strand and bound to third strand to form a new G-triplex (E), (**III**) first G of second strand unzipped (F), rotation of second strand to form a cross-like triplex (G), eventually second strand detached and the two molecule parts got separated (H); the 5'-G reversibly visited the *syn* conformation	S22B
	run2: last (A) and first G unzipped (B), (**II**) second G unzipped (C), second to last G unzipped (D), third G unzipped (E), the remainder unfolded and the two molecule parts got separated (F)	S22C
	run3: first (A) and last G unzipped (B), then (**II**) 5'-G flipped to *syn*, second (C), second to last (D), third (E), and (**III**) third to last G unzipped (F), so middle (diagonal) hairpin remained	S22D
$143D_{loop\text{-}pull}$	run1: (**II**) first G unzipped (A), GQ opening (B), first strand detached (C), second strand detached (D), so that 3'-hairpin remained	S23B
	run2: third G of the third strand unzipped (A), first G unzipped (B), (**II**) rotation of strands into cross-like GQ (C), then second (D) and third G unzipped (E), second strand detached (F), so that perturbed 3'-hairpin remained	S23C
	run3: third (A) and second G of the third strand unzipped (B), first strand detached (C), (**II**) 3'-triplex with two G-triplets refolded (D), second strand detached (E), so that perturbed 3'-hairpin remained	S23D
$143D_{syn\_loop\text{-}pull}$	run1: spiral structure formed (A), GQ opening (B) followed by strand rotation to cross-like GQ, (**II**) last G of third strand unzipped (C), spiral structure then partially refolded, second G of the third strand unzipped (D) and 5'-triplex with one quartet refolded, spiral structure formed (E), first G of the third strand slipped in the 3'-direction (F), first G of the last strand unzipped (G), first G of the third strand unzipped and fourth strand rotated into cross-triplex (H), first and second G slipped in the 5'-direction (I), last strand detached (J), 5'-G-hairpin gradually unfolded	S24B
	run2: (**II**) spiral structure formed (A), last G of the third strand pulled out (B), GQ opening (C), third strand detached (D), first strand detached (E), 3'-triplex refolded (F), second strand detached (G), 3'-hairpin began to unfold	S24C
	run3: spiral structure formed (A), then (**II**) first (B), second (C) and third G unzipped (D) and 3'-triplex refolded, second strand detached (E), so that 3'-G-hairpin remained	S24D

[a] The capital letters (A, B, C…) denote the events and structural snapshots in the respective Supporting Figures. Roman numerals II and III denote events that happened after the first external force drop, i.e. in phase II, and after reaching maximum allowed extension, i.e. phase III, respectively.

Table S12: Outcome of simulations with the *very slow pulling* setup.[a]

System	Outcome	Figures
1KF1	run1: GQ opening (A), last G unzipped + GQ refolded (B), last G stacked under third strand (C), GQ opening between third and fourth strand (D), GQ reformed (E), quick opening and reformation (F), last strand slipped down one level (G), last G unstacked (H), first G unzipped (I), spiral 1-4 formed (J), opening (K), last strand slipped one level downwards (L), last strand detached (M), third strand slipped one level downwards (N), third strand slipped one level downwards (O), third strand detached (P), incomplete 5'-G-hairpin remained	S25B
	run2: last G unzipped (A), third+last strand slipped down one level (B), first G unzipped (C), last strand slipped one level downwards (D), second to last G detached (E), last strand detached (F), incomplete 5'-G-triplex remained	S25C
	run3: spiral structure (A), first strand slipped upwards (B), first G unzipped (C), last G unzipped (D), first strand slipped upwards (E), first strand detached (F), last G reattached to reform a perfect G-triplex (G), first G of second strand unzipped (H), last G slipped downwards one level (I), last strand slipped one level downwards (J), second strand slipped one level downwards (K), last strand detached (L), second strand slipped one level upwards (M), incomplete middle G-hairpin remained	S25D
2GKU	run1: GQ opening and rotation into cross-like GQ (A), first G unzipped (B), first G of second strand detached + second G unzipped (lever to detach first G of second strand) (C), last G unzipped (D), first strand + second G of second strand detached (E), last G rebound (F), last G of second strand unzipped and 3'-hairpin remained (G), last G unzipped (H), second to last G unzipped (I), last strand unzipped (J), attempts to refold 3'-hairpin until the end of the simulation, up to two G:G pairs formed	S26B
	run2: GQ opening and rotation into cross-like GQ (A), last G unzipped (B), second to last G unzipped (C), fist G unzipped (D), last strand unzipped (E) and third strand derotated to form an incomplete 5'-triplex, last G of third strand detached (F), imperfect 5'-triplex remained	S26C
	run3: GQ opening and rotation into cross-like GQ (A), first G unzipped (B), last G unzipped (C), first strand + first G of second strand detached (D), imperfect 3'-triplex remained	S26D
143D	run1: first G unzipped (A), second G unzipped (B), last G unzipped (C), third G unzipped (D), so that an incomplete 3'-triplex remained	S27B
	run2: first G unzipped (A), last G unzipped (B), second G unzipped (C), second to last G unzipped (D), so that a quartet + diagonal hairpin remained *– had three cations in the "channel"*	S27C
	run3: first G unzipped (A), last G unzipped (B), second to last G unzipped (C), second G unzipped (D), so that a quartet + diagonal hairpin remained – *had three cations in the "channel" (at different sites)*	S27D
$143D_{NaCl}$	run1: first G unzipped(A); second G unzipped (B); last G unzipped (C); second to last G unzipped (D); so that a quartet + diagonal hairpin remained	S29B
	run2: first quartet opened and divided into two G:G pairs (strands 1+3 and 2+4) (A); last G unzipped (B); first G unzipped C); second G unzipped (D); so that a quartet + triplet remained	S29C
	run3: first G unzipped (A); second G unzipped (B), third G unzipped (C); last G unzipped (D), so that an incomplete 3'-triplex remained	S29D

[a] The capital letters (A, B, C…) denote the events and structural snapshots in the respective Supporting Figures.

Table S13: Outcome of simulations with the *fast pulling* box150Å setup.

System	Outcome	Figures
$1KF1_{box150Å}$	run1: spiral structure (A), opening and rotation into cross-like GQ (B), first strand slipped in 5'-direction (C), division into two G-hairpins (D), reversible unzipping of last and later two last G's (E), last strand detached (F), first strand detached (G)	S30B
	run2: last G unzipped (A), GQ opening (B), last strand detached (C), third strand and last G of second strand detached (D), first strand slipped in 5'-direction (E)	S30C
	run3: last strand slipped in 3'-direction (A), third and last strand slipped in 3'-direction (B), last strand detached (C), middle G of third strand unzipped (D), first G of third strand detached (E), first strand slipped in 5'-direction (F), incomplete 5'-hairpin unfolded (G)	S30D
$1KF1_{syn\text{-}box150Å}$	run1: rotation into cross-like GQ (A), GQ opening (B), last G unzipped (C), first G unzipped (D), last strand detached (E), last G of third strand unzipped (F), third strand detached (G), last G of second strand unzipped (H), remaining incomplete 5'-hairpin unfolded	S31B
	run2: rotation into cross-like GQ (A), first G unzipped (B), last G unzipped (C), last strand detached (D), third strand detached (E), incomplete 5'-hairpin unfolded (F)	S31C
	run3: rotation into cross-like GQ (A), GQ opening (B), last G unzipped (C), first G unzipped and second to last G unzipped (D), last strand detached (E), last G of third strand unzipped (F), second G of third strand unzipped (G), third strand detached (H), incomplete 5'hairpin unfolded (I)	S31D
$2GKU_{box150Å}$	run1: GQ opening and rotation into cross-like GQ (A), second G unzipped (B), first G unzipped (C), last G unzipped (D), first strand detached (E), first G of second strand unzipped (F), second G of second strand unzipped (G), second strand detached (H), second to last G unzipped (I)	S32B
	run2: rotation into cross-like GQ (A), GQ opening (B), first G unzipped (C), last G unzipped (D), GQ divided into two incomplete hairpins (E), second to last G unzipped (F), last G of second strand unzipped (G), 3'-hairpin unfolded (H)	S32C
	run3: rotation into cross-like GQ (A), GQ opening and last G unzipped (B), second to last G unzipped (C), first G unzipped (D), GQ divided into two hairpins (E), last G of second strand unzipped (F), 5'-hairpin unfolded (G), 3'-hairpin unfolded (H)	S32D
$143D_{box150Å}$	run1: first G unzipped (A), last G unzipped (B), second G unzipped (C), second to last G unzipped (D), third G unzipped (E), second strand unbound from last strand and formed an imperfect diagonal hairpin (F), second strand detached (G), last strand detached (H)	S33B
	run2: first G unzipped (A), last G unzipped (B), second G unzipped (C), third G unzipped (D), second strand unbound from last strand and formed an imperfect diagonal hairpin (E), second strand detached (F), second to last G unzipped (G)	S33C
	run3: first G unzipped (A), second G unzipped (B), third G unzipped (C), third G-triplet disrupted (D), first G of second strand bound to third G of third strand (E), second G detached (F), last G unzipped (G), second to last G unzipped (H)	S33D
$143D_{syn\text{-}box150Å}$	run1: first G unzipped (A), last G unzipped (B), second to last G unzipped (C), second G unzipped (D), last strand detached (E), third strand rotated and H-bonded to second strand (F), first strand detached (G), last G of third strand unzipped (H), middle hairpin unfolded (I)	S34B

	run2: last G unzipped (A), first G unzipped (B), second to last G unzipped (C), second G unzipped (D), last strand detached (E), first strand detached (F), *no middle hairpin formed, so the structure disintegrated*	S34C
	run3: last G unzipped (A), second to last G unzipped (B), last strand detached (C), first G unzipped (D), last G of third strand unzipped (E), second G unzipped (F), diagonal hairpin progressively unzipped (G), incomplete 5'-hairpin unfolded	S34D
$143D_{noloop\text{-}box150Å}$	run1: first G unzipped (A), 3'-triplet fluctuating geometry (H-bonds between G's from strands 2-4-3, 2-3-4, symmetric, 2 + 3-4) (B), second G unzipped (C), third G unzipped (D), last G unzipped (E), second strand rotated into cross-triplex (F), first G of second strand unzipped (G), second strand unbound (H)	S35B
	run2: first G unzipped (A), last G unzipped (B), second G unzipped (C), first strand detached (D), first G of second strand unzipped (E), second strand slipped in (its) 5'-direction (*2x misfolded tWH G:G base pair*) (F), second strand unbound (G), 3'-hairpin refolded (H)	S35C
	run3: first G unzipped (A), last G unzipped (B), second G unzipped (C), first strand detached (D), second strand rotated into cross-triplex (*opposite polarity than in run1*) (E), second to last G unzipped (F), second strand unbound (G), last G rebound (H), 3'-hairpin refolded (I)	S35D
$143D_{loop\text{-}pull\text{-}box150Å}$	run1: last G of third strand unzipped (A), GQ opening (B), third strand detached (C), first G unzipped (D), second G unzipped (E), first strand detached (F), first G of second strand unzipped (G), second G of second strand unzipped (H), second strand detached (I)	S36B
	run2: first G unzipped (A), GQ opening (B), second G unzipped (C), last G of third strand unzipped (D), first strand detached (E), second strand detached (F), 3'-hairpin refolded (G)	S36C
	run3: last G of third strand unzipped (A), first G unzipped (B), GQ opening (C), second G unzipped (D), first strand detached (E), first G of second strand unzipped (F), second G of second strand unzipped (G), second strand detached (H)	S36D
$143D_{syn\_loop\text{-}pull\text{-}box150Å}$	run1: first strand in cross-orientation (*sort of imperfect spiral structure*) (A), first G unzipped (B), GQ opening (C), second strand in cross-orientation (D), second G unzipped and second strand derotated (E), last G of third strand unzipped (F), first strand detached (G), second strand detached (H)	S37B
	run2: spiral structure (A), GQ opening (B), first G unzipped (C), last G of third strand unzipped (D), second G unzipped (E), first strand detached (F), first G of second strand unzipped (G), second G of third strand unzipped (H), second strand detached (I), 3'-hairpin refolded (J)	S37C
	run3: spiral structure (A), GQ opening and rotation of first strand to cross-orientation (B), gradual slippage of first strand and ultimately first strand detached (C), first G of second strand unzipped (D), last G of third strand unzipped (E), second strand detached (F), 3'-hairpin refolded (G)	S37D
$143D_{NaCl\text{-}box150Å}$	run1: first G unzipped (A), second G unzipped (B), third G unzipped (C), last G unzipped (D), second strand detached (E), last G re-attached (F), last G unzipped (G), second to last G unzipped (H), last strand detached (I)	S38B
	run2: first G unzipped (A), second G unzipped (B), third G unzipped (C), last G unzipped (D), first G of second strand unzipped (E), second strand detached (F), last G re-attached (G), last G unzipped (H), second to last G unzipped (I), last strand detached (J)	S38C

	run3: first G unzipped (A), second G unzipped (B), third G unzipped (C), second strand detached (D), last G unzipped (E), second to last G unzipped (F), last strand detached (G)	S38D

[a] The capital letters (A, B, C…) denote the events and structural snapshots in the respective Supporting Figures.

Table S14: Outcome of simulations with the *very slow pulling* box150Å setup.[a]

System	Outcome	Figures
$143D_{box150Å}$	run1: first G unzipped (A), last G unzipped (B), second G unzipped (C), second G of third strand unbound from second to last G (D), second G of third strand rebound to second to last G (E), second G of second strand unbound from second to last G (F), second to last G unzipped (G), last quartet divided into two G:G pairs (strands 1+2, 3+4) (H), last strand detached (I), first strand detached (J), diagonal hairpin started opening (K) and gradually unfolded	S39B
	run2: first G unzipped (A), last G unzipped (B), second G unzipped (C), second G of second strand unzipped from second to last G (D), second to last G rotated into cross-orientation (E), second to last G unzipped (F), third G unzipped (G), second strand bound to third strand (H), first and second G of second strand detached (I), second strand detached (J), last strand detached (K)	S39C

[a] The capital letters (A, B, C…) denote the events and structural snapshots in the respective Supporting Figures.

Table S15: Outcome of simulations with the *very slow pulling* setup of all G-triplex models.[a]

System	Outcome	Figures
1KF1_t_pp	run1: strand slippage first strand (A), strand slippage last strand (B), third strand detached (C), strand slippage first strand back to original position (D), rotation into cross-like hairpin and eventual unfolding (E)	S40B
	run2: strand slippage first strand (A), first strand detached (B), 3'-hairpin rotated into cross (C) and eventually unfolded	S40C
	run3: first strand detached (A), last strand slipping back and forth (B), *gradual detachment of two G's until the next event*, rotation into cross and eventual unfolding (C)	S40C
$2JPZ\_t\_l_np$	run1: last G unzipping back and forth (A) until ultimate unzipping, first G unzipped (B), first strand detached (C), remaining hairpin unfolded (D)	S41B
	run2: last G unzipped (A), first and second G unzipped (B), third G unzipped (C), last G returned and 3'-hairpin refolded completely (D)	S41C
	run3: last G unzipped (A), second to last G unzipped (B), first and second G unzipped (C), third G unzipped (D), second to last G returned (E)	S41D
$2GKU\_t\_pl_w$	run1: first G unzipped (A), back and forth unzipping of last G (B) *until ultimate unzipping*, last strand detached (C), incomplete 5'-hairpin unfolded (D)	S42B
	run2: last strand rotated into cross-triplex (A), last strand derotated and last G detached (B), last strand detached (C), last pair of 5'-hairpin broken (D)	S42C
	run3: back and forth unzipping of last G (A), unzipping of first G (B), last strand detached (C), formation of a dynamic misfolded G-triplex involving base pairs between first and last strand (C), *ultimate disintegration*	S42D
$2GKU\_t\_l_wl_n$	run1: rotation of first strand into cross-triplex (A), first G unzipped (B), last G unzipped (C), first strand detached (D)	S43B

	run2: rotation of first strand into cross-triplex (A), first G unzipped (B), last two G's unzipped (C), first strand detached (D), *remaining incomplete 3'-hairpin eventually unfolded*	S43C
	run3: rotation of first strand into cross-triplex (A), first G unzipped, first strand derotated (B), first strand detached (C), last G unzipped (D), internal strand slippage (E), first G of second strand unzipped, internal strand slippage disappeared (F), gradual unfolding of incomplete 3'-hairpin (G)	S43D
143D_t_l_wd	run1: first G of last strand unpaired from first G and paired with the middle strand (*via cross-triplex intermediate, sometimes symmetric G-triad*) (A), last strand turned into cross-triplex (B), first G unzipped (C), last two G's detached (D), last strand detached (E), *incomplete imperfect 5'-hairpin remained*	S44B
	run2: last strand slipped downwards (*last G paired with first G of middle strand*) (A), last strand detached (*first strand rotated into 5'-cross-hairpin for a while*) (B), first strand detached (C)	S44C
	run3: first G of last strand paired with last G of middle strand (A), last strand unbound from first strand, bound to middle strand (B), last strand rotated to cross-triplex (*gradual loss of correct H-bonding of the last two G's*) (C), first G detached (D), second and third G lost base pairing with middle strand (E), rotation of first strand to cross-orientation (F), first strand detached (G), last two G's detached (H), last base pair unfolded (I)	S44D
143D_t_dl_n	run1: first G of middle strand unpaired (A), third G unpaired, i.e., third triplet fully disrupted (B), first strand unbound from strand last strand and rotated into cross-orientation (C), first strand detached (D), last G unzipped (E)	S45B
	run2: first G of middle strand unpaired (A), third G unpaired, i.e., third triplet fully disrupted (B), first strand rotated into cross-orientation, unbound from the last strand (C), derotation of the first strand and imperfect Hoogsteen bonding (one H-bond per pair) with the middle strand (D), first strand detached (E), last G detached (F), second to last G unzipped (G), *gradual unfolding of the 3'-hairpin remainder*	S45C
	run3: first G of middle strand unpaired (A), third G unpaired, i.e., third triplet fully disrupted (B), first strand rotated into cross-triplex (C), 5'-end of first strand moved towards last strand (D), first strand gradually detached (E), last strand detached (F)	S45D

[a] The capital letters (A, B, C…) denote the events and structural snapshots in the respective Supporting Figures.

SUPPORTING FIGURES

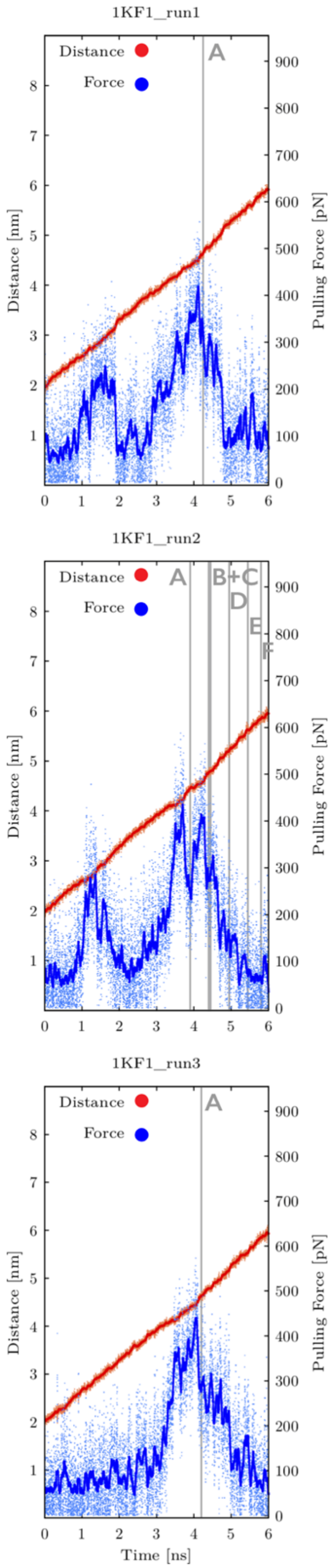

Figure S1A: Time evolution of distance between pulling centers and pulling force during three independent *fast pulling* simulations of 1KF1 GQ system. Snapshots were saved every 0.6 ps and plots are showing both instantaneous values (orange and light-blue dots for distance and force,

respectively) and smoothing, i.e., averaging over 100 consecutive snapshots (red and blue lines for distance and force, respectively). Main structural events are highlighted as grey vertical lines with labels (capital letters). See Figures S1B and S1C for inspection of structures corresponding to main structural events. Note that first major drops of the pulling force before the GQ unfolding event "A" are connected with repositioning of terminal T residues.

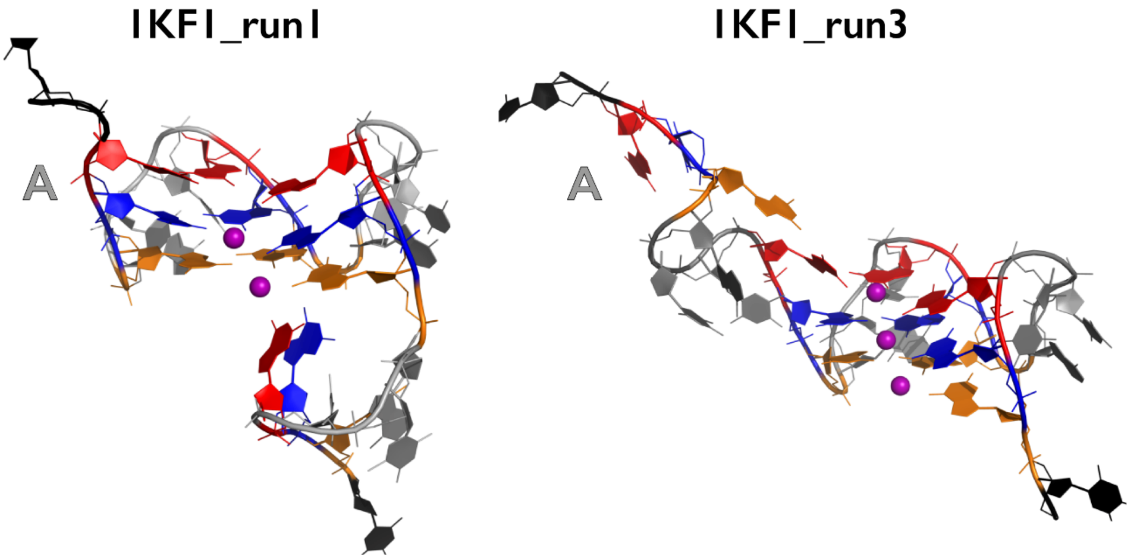

Figure S1B: Most important structural events during first and third independent *fast pulling* simulations of 1KF1 GQ system. G residues from first (5'-end), second and third quartet are highlighted in orange, blue and red, respectively. Pulling centers, i.e., either both terminal T residues or one terminal and other T residue from the loop (in some structures, see Methods in the main text for details), are shown in black. Remaining DNA residues are in gray and channel K^+ ions are shown as purple spheres. Additional K^+ ions, Cl^- ions, H-atoms and water molecules are not shown for clarity.

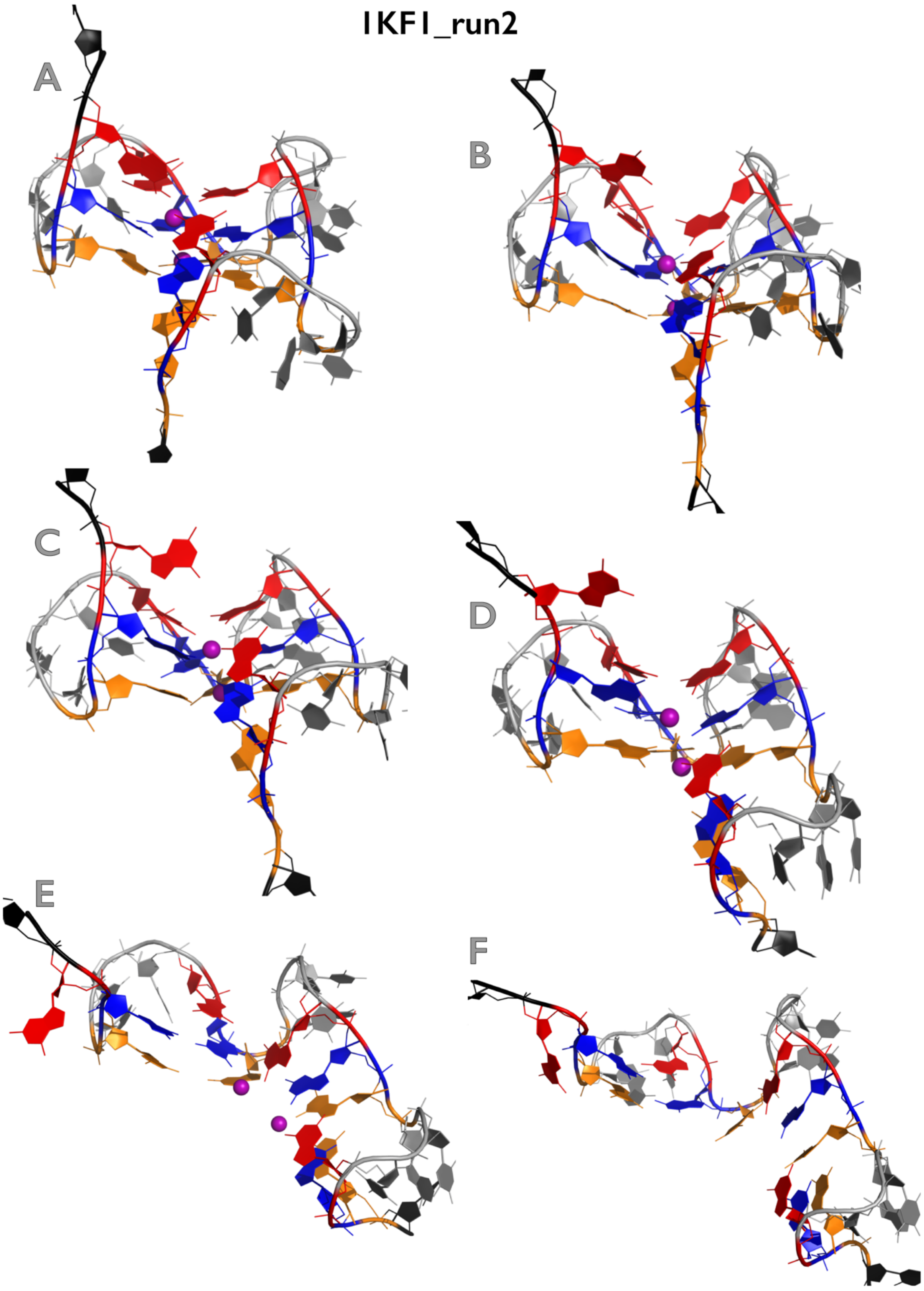

Figure S1C: Most important structural events during second independent *fast pulling* simulation of 1KF1 GQ system. See legend of Figure S1B for more details.

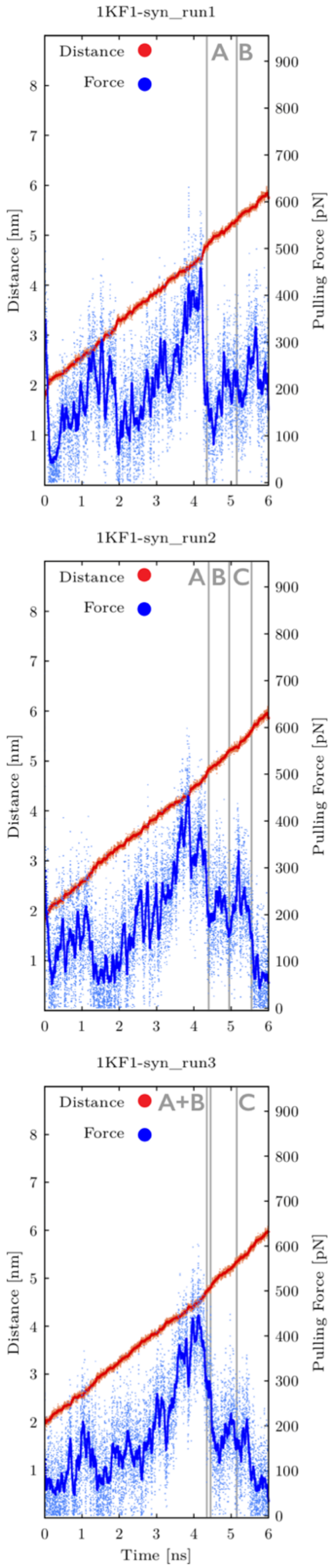

Figure S2A: Time evolution of distance between pulling centers and pulling force during three independent *fast pulling* simulations of $1KF1_{syn}$ GQ system (see legend of Figure S1A for more

details). See Figures S2B and S2C for inspection of structures corresponding to main structural events.

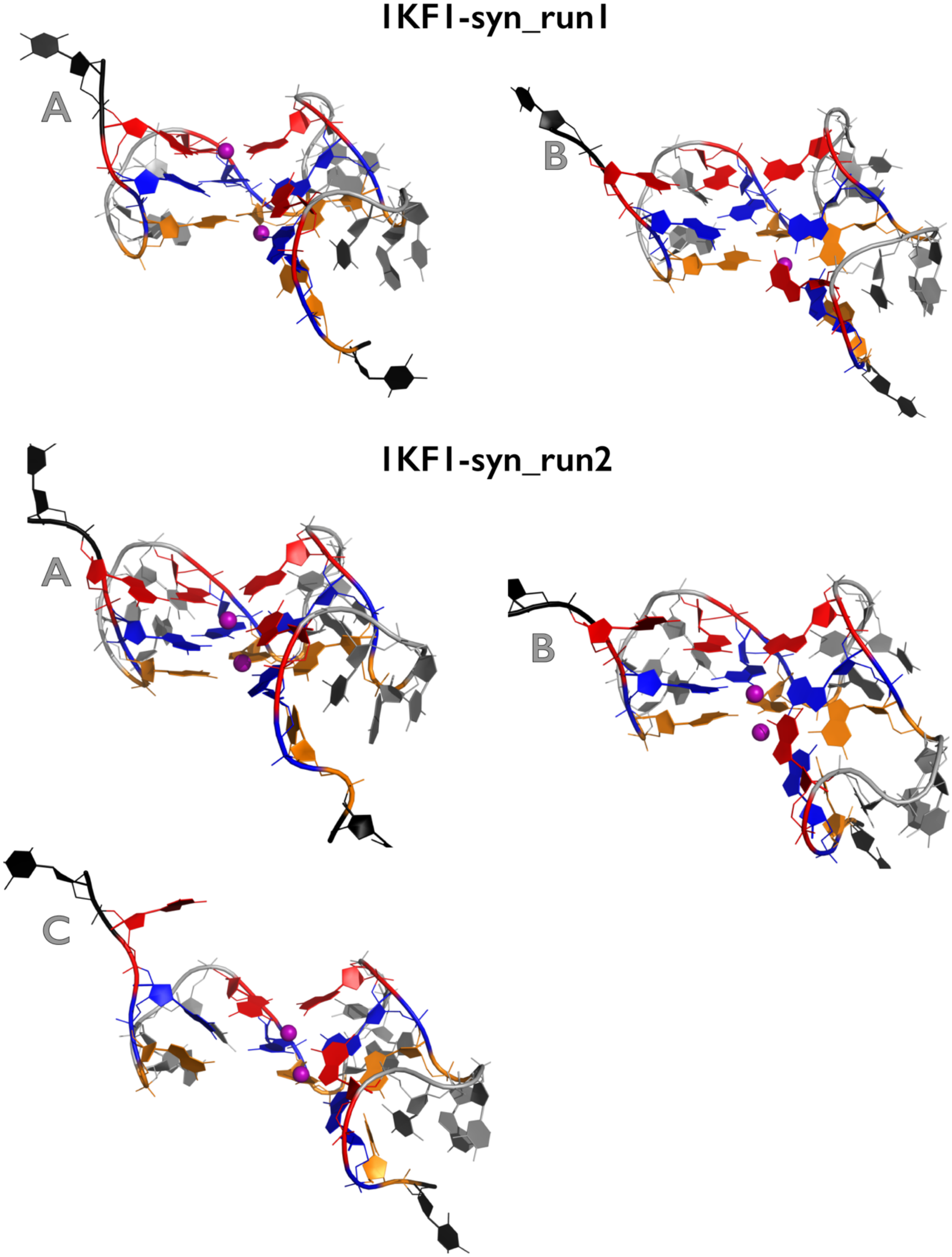

Figure S2B: Most important structural events during first and second independent *fast pulling* simulations of $1KF1_{syn}$ GQ system. See legend of Figure S1B for more details.

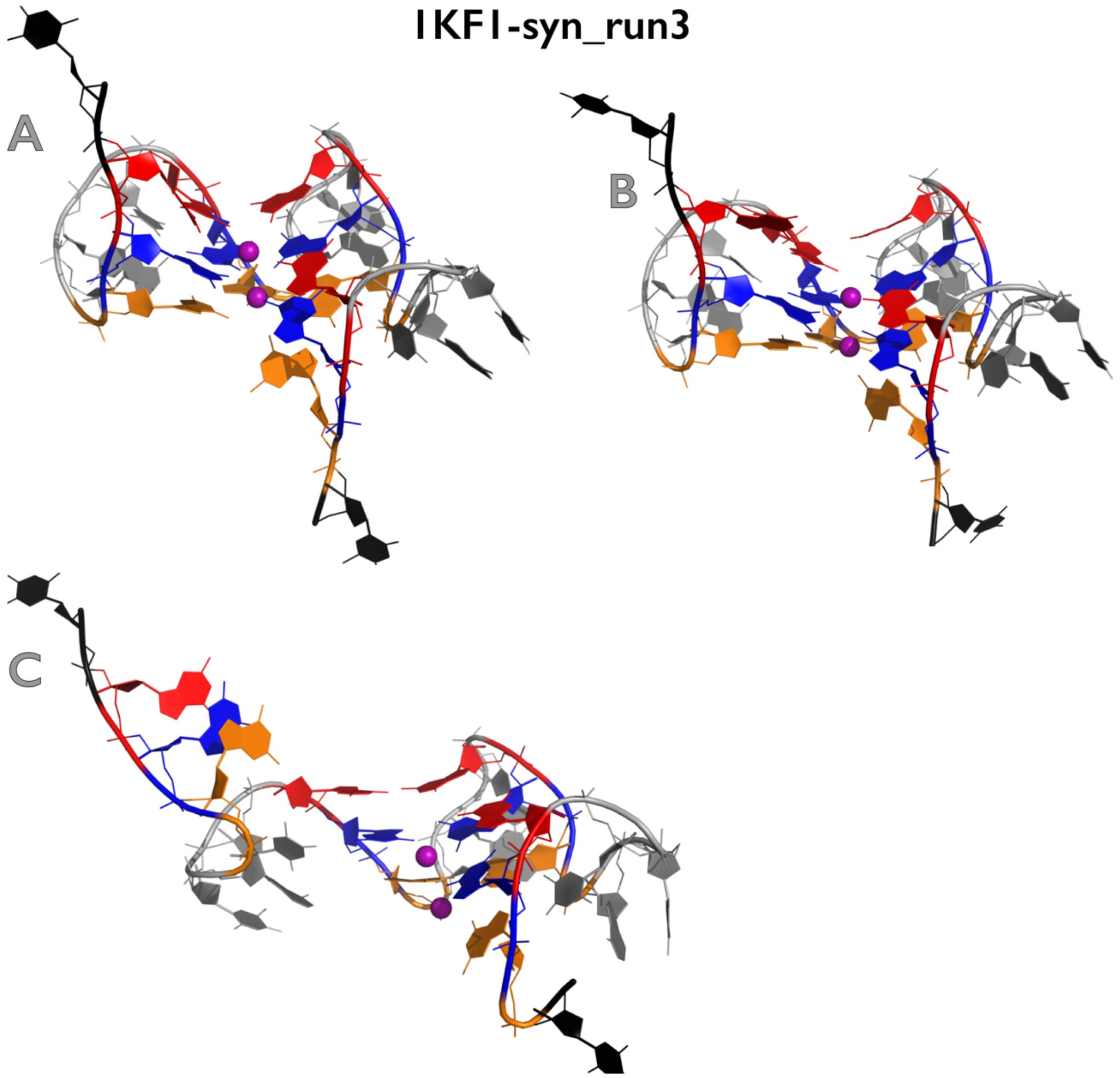

Figure S2C: Most important structural events during third independent fast pulling simulation of $1KF1_{syn}$ GQ system. See legend of Figure S1B for more details.

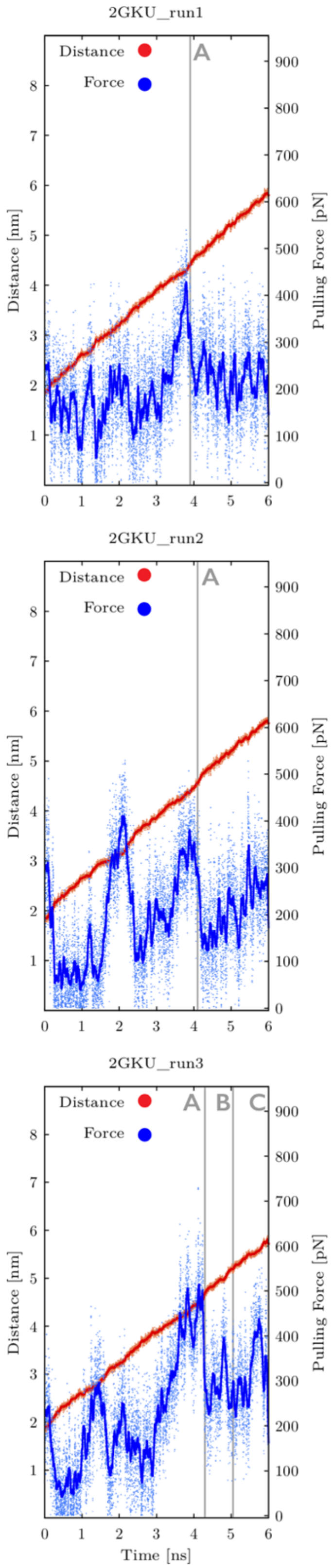

Figure S3A: Time evolution of distance between pulling centers and pulling force during three independent *fast pulling* simulations of 2GKU GQ system (see legend of Figure S1A for more details). See Figure S3B for inspection of structures corresponding to main structural events.

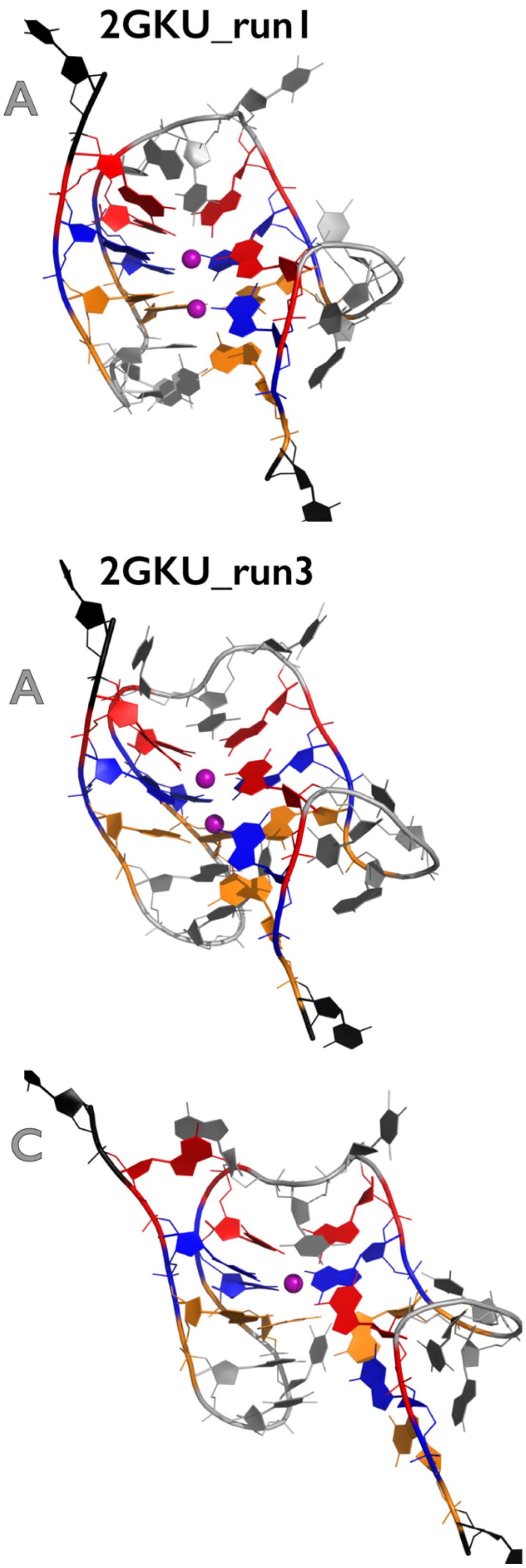

Figure S3B: Most important structural events during three independent *fast pulling* simulations of 2GKU GQ system. See legend of Figure S1B for more details.

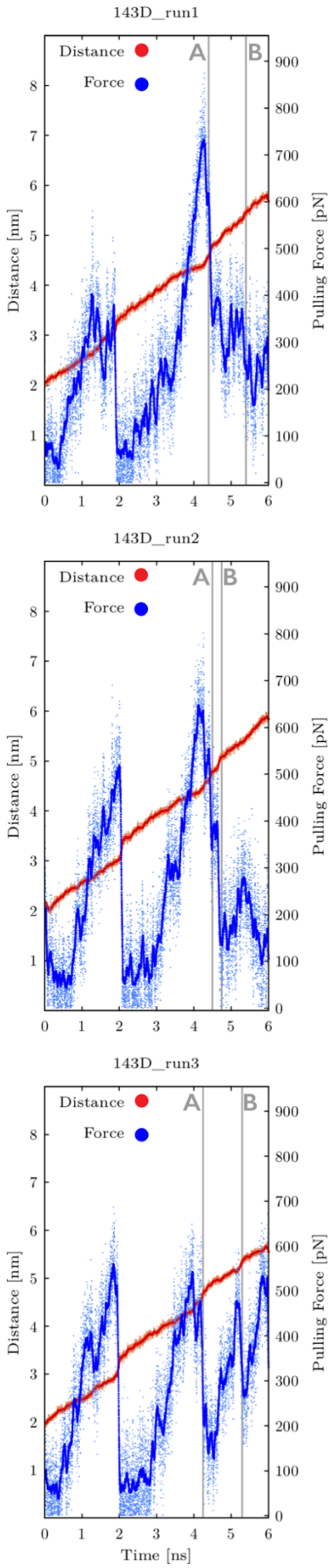

Figure S4A: Time evolution of distance between pulling centers and pulling force during three independent *fast pulling* simulations of 143D GQ system (see legend of Figure S1A for more details). See Figure S4B for inspection of structures corresponding to main structural events.

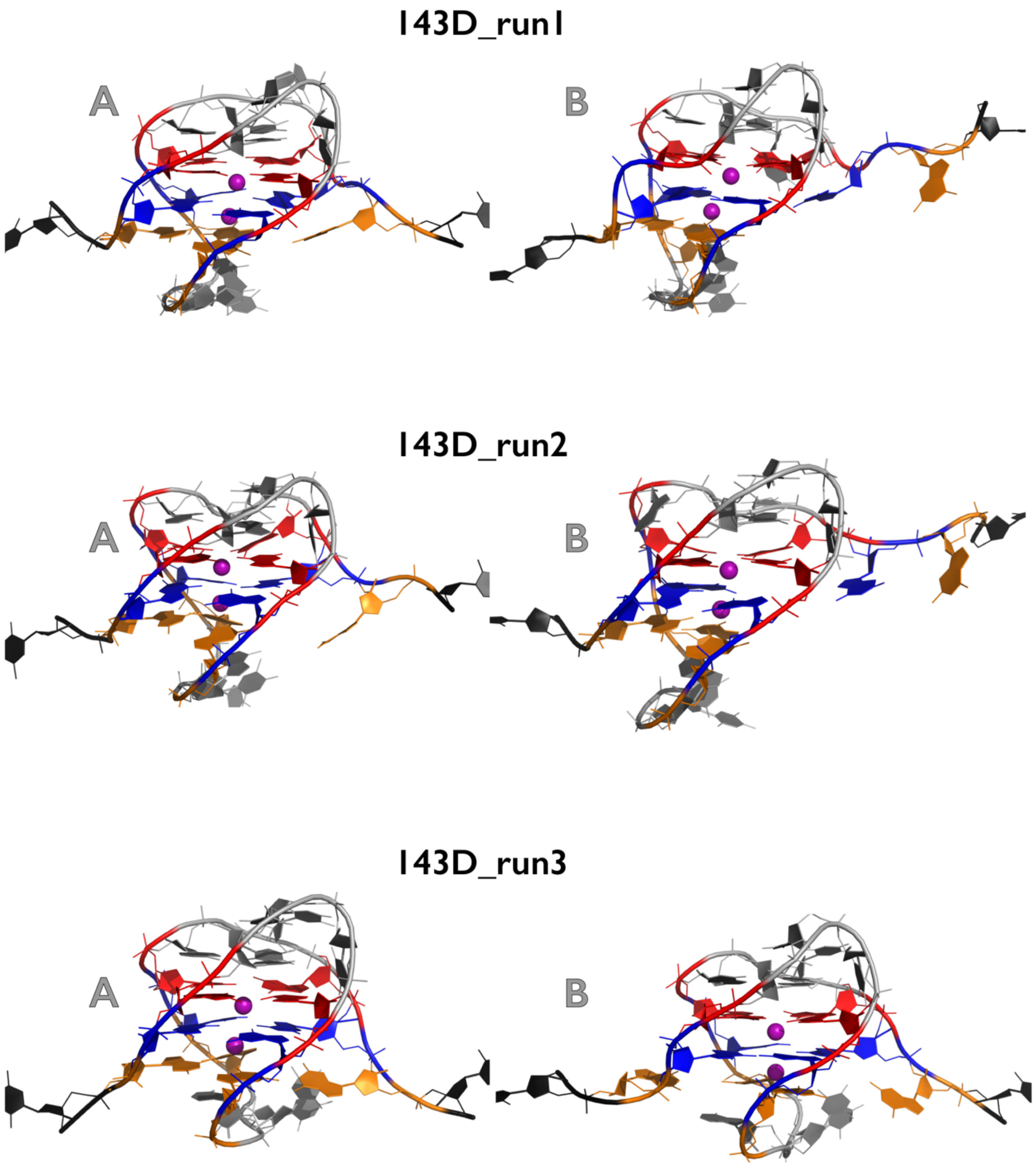

Figure S4B: Most important structural events during three independent *fast pulling* simulations of 143D GQ system. See legend of Figure S1B for more details.

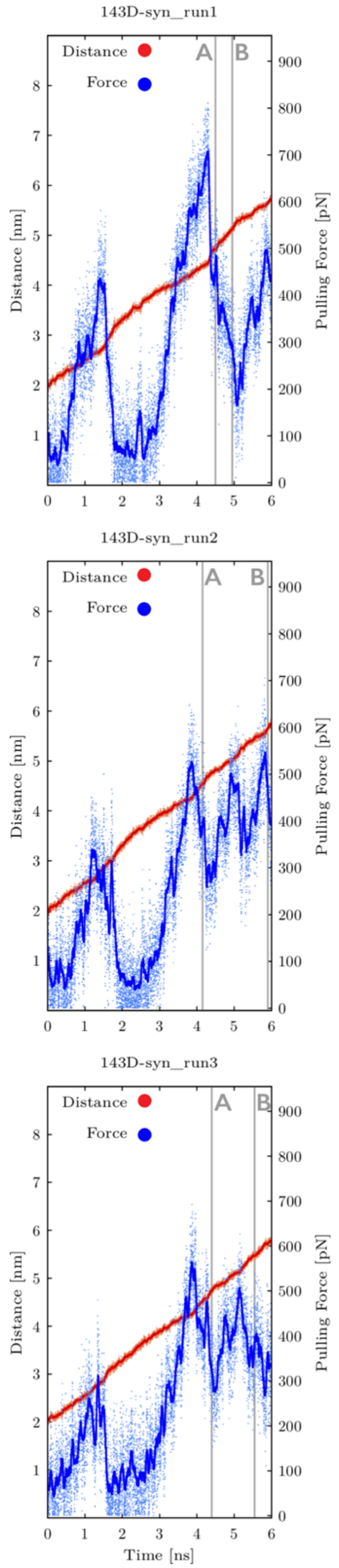

Figure S5A: Time evolution of distance between pulling centers and pulling force during three independent *fast pulling* simulations of 143D$_{syn}$ GQ system (see legend of Figure S1A for more details). See Figure S5B for inspection of structures corresponding to main structural events.

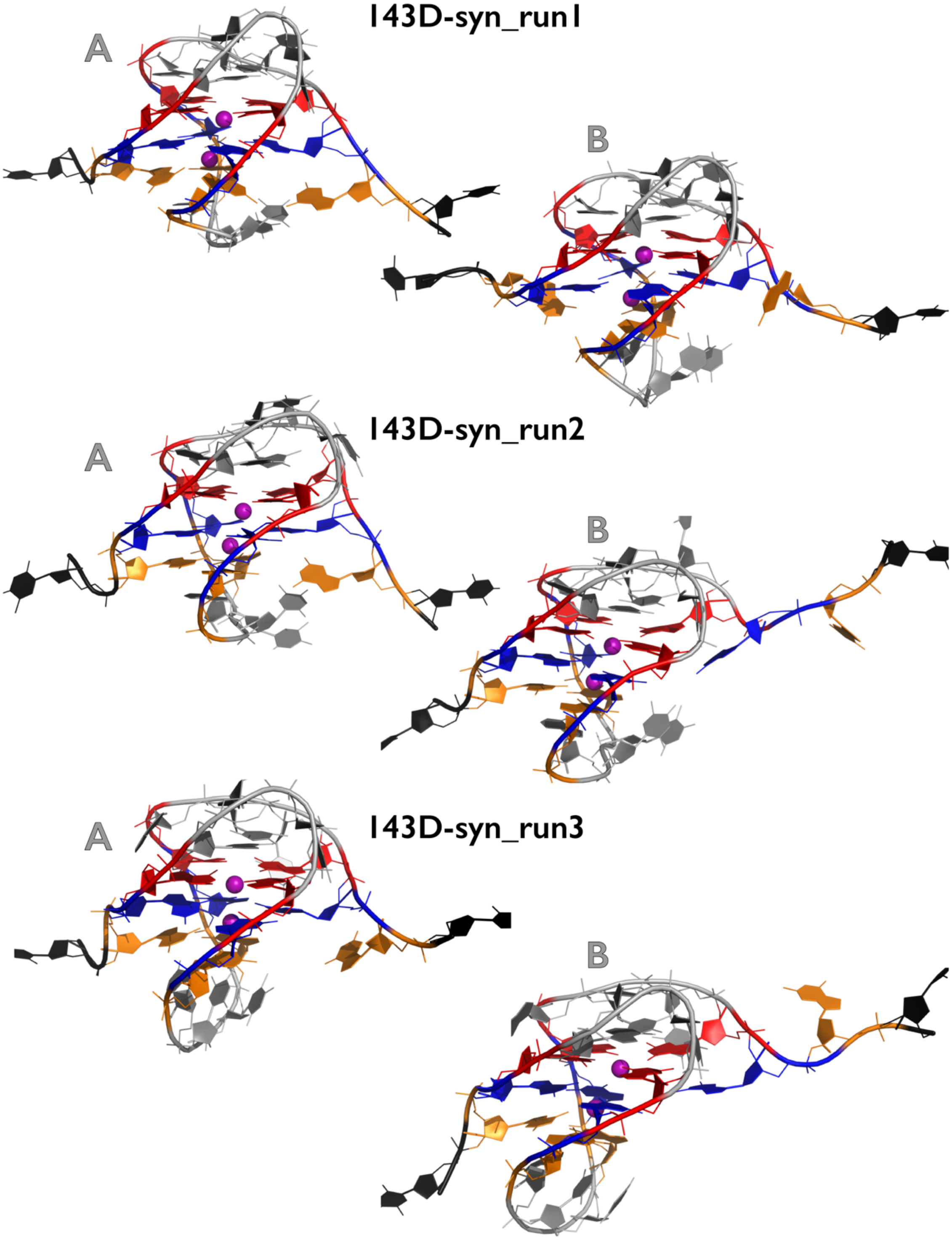

Figure S5B: Most important structural events during three independent *fast pulling* simulations of $143D_{syn}$ GQ system. See legend of Figure S1B for more details.

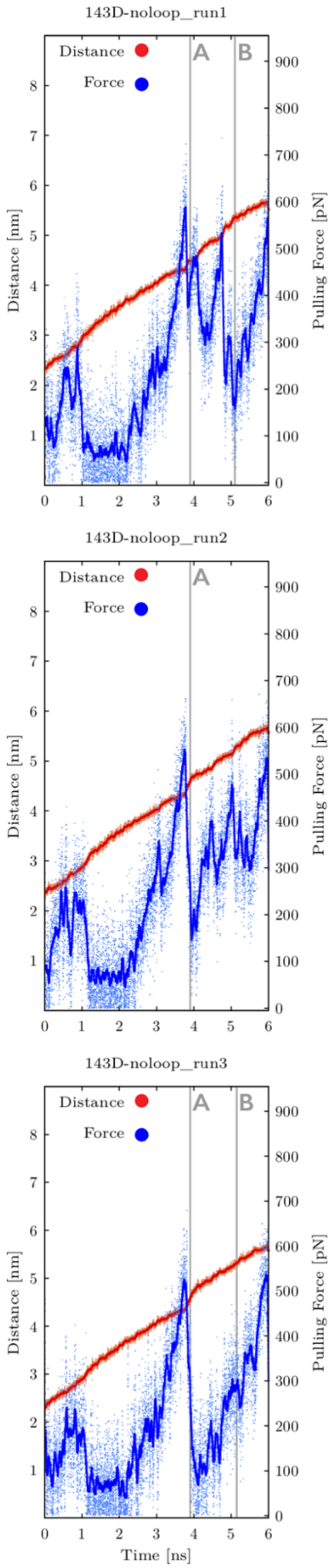

Figure S6A: Time evolution of distance between pulling centers and pulling force during three independent *fast pulling* simulations of $143D_{noloop}$ GQ system (see legend of Figure S1A for more details). See Figure S6B for inspection of structures corresponding to main structural events.

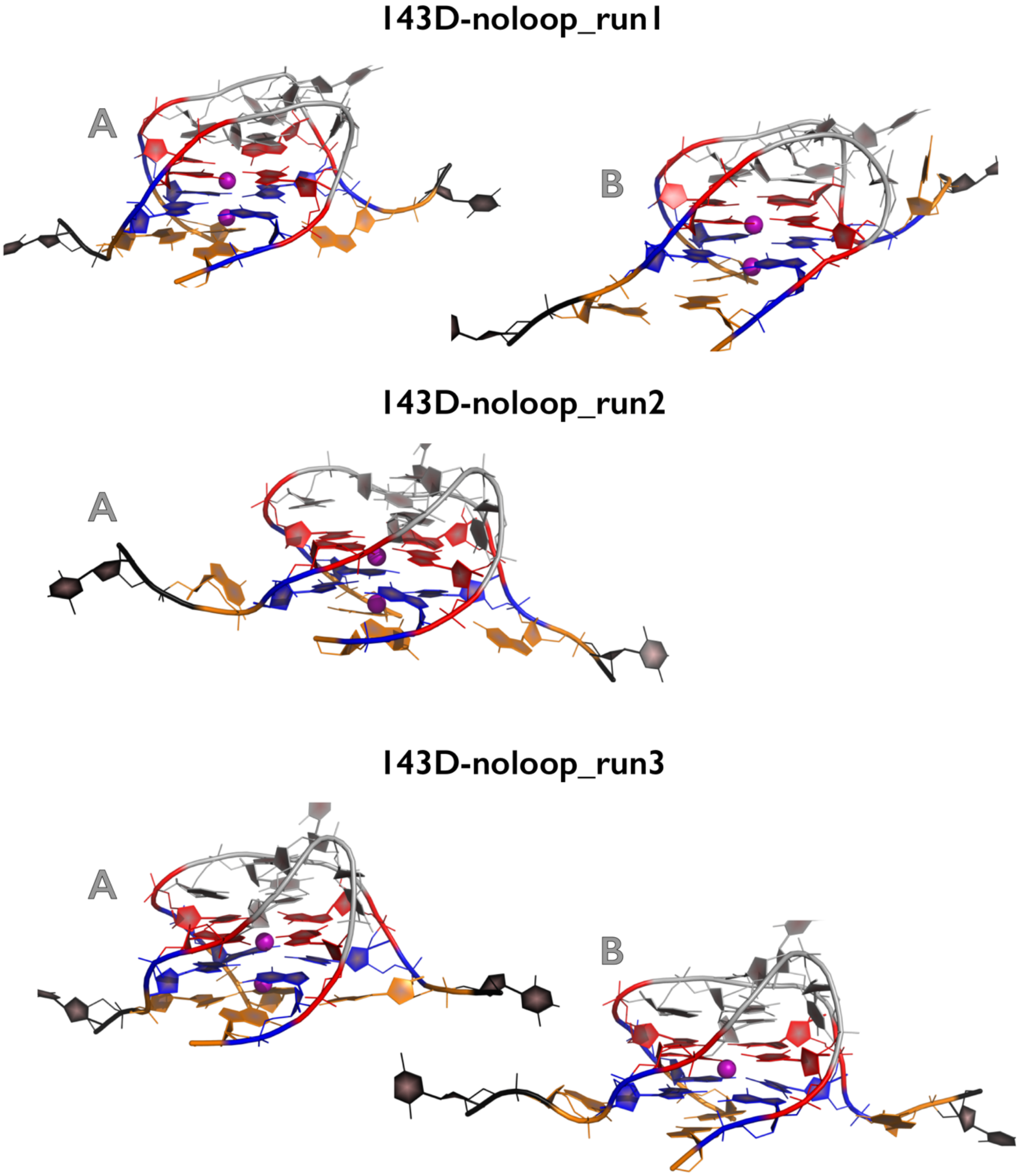

Figure S6B: Most important structural events during three independent *fast pulling* simulations of $143D_{noloop}$ GQ system. See legend of Figure S1B for more details.

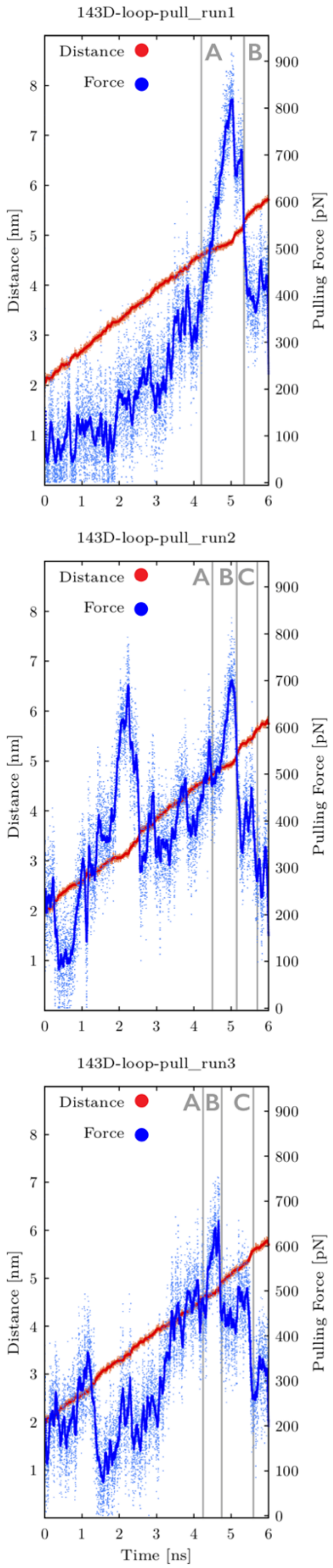

Figure S7A: Time evolution of distance between pulling centers and pulling force during three independent *fast pulling* simulations of 143D$_{loop-pull}$ GQ system (see legend of Figure S1A for more

details). See Figures S7B and S7C for inspection of structures corresponding to main structural events.

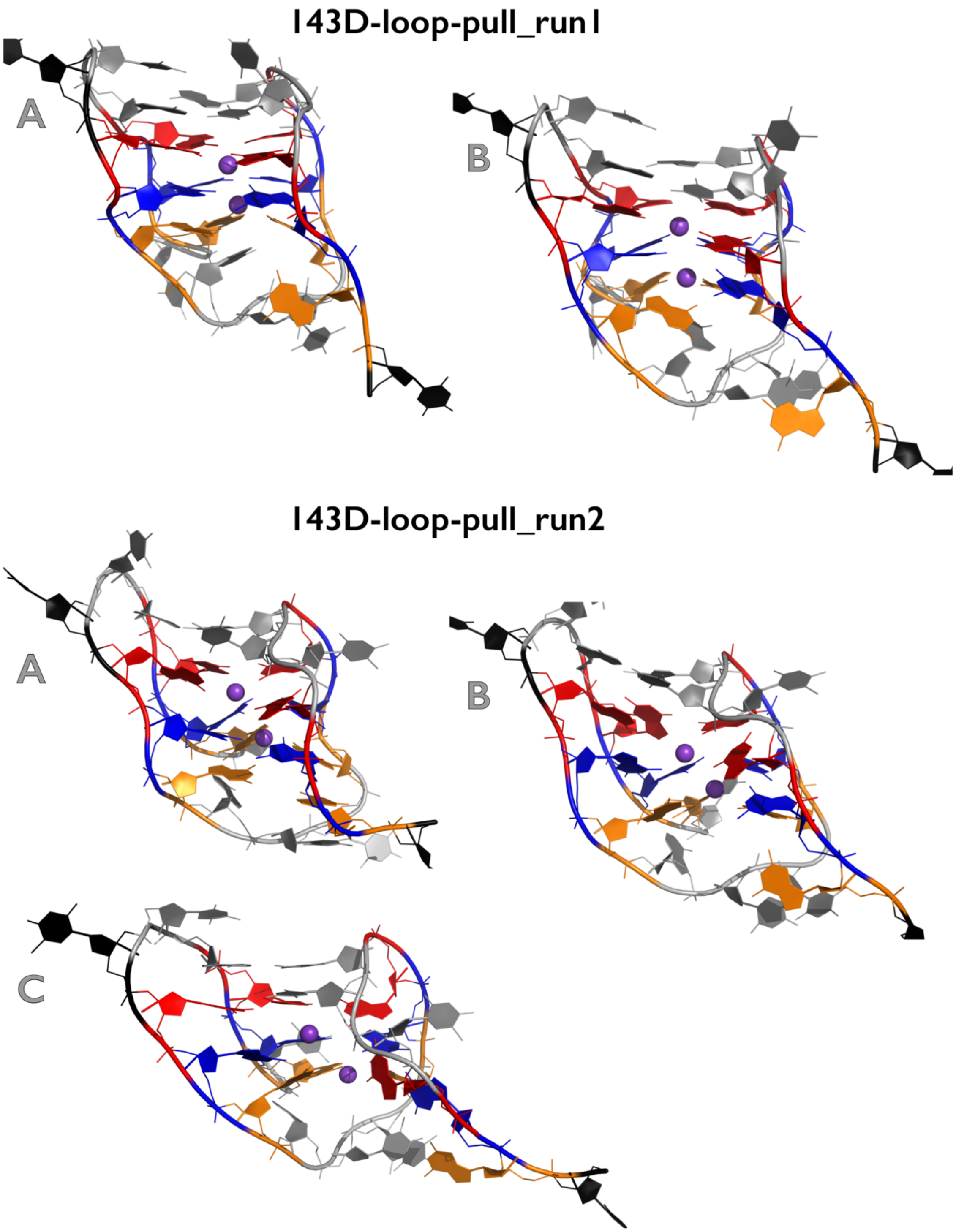

Figure S7B: Most important structural events during first and second independent *fast pulling* simulations of 143D$_{loop\text{-}pull}$ GQ system. See legend of Figure S1B for more details.

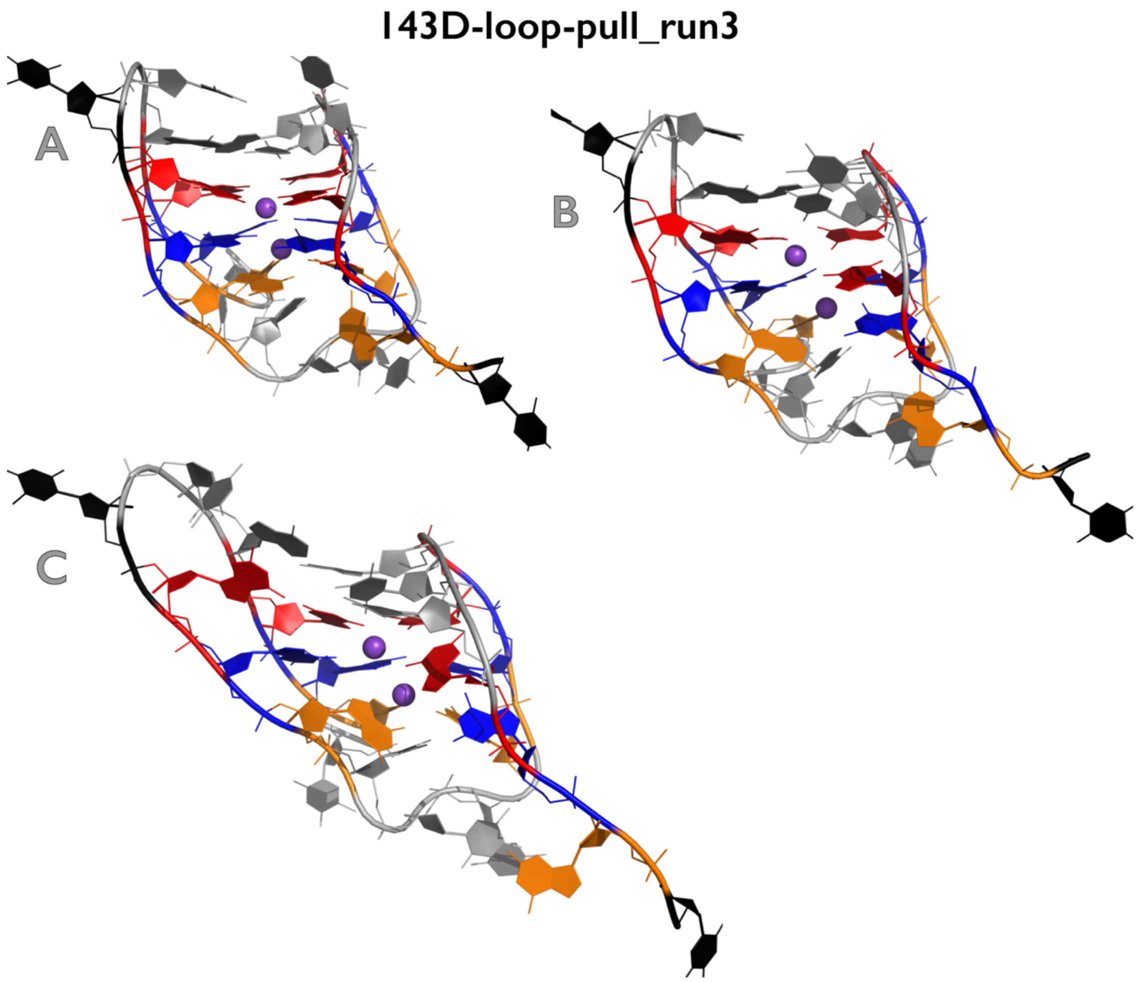

Figure S7C: Most important structural events during third independent *fast pulling* simulation of 143D$_{\text{loop-pull}}$ GQ system. See legend of Figure S1B for more details.

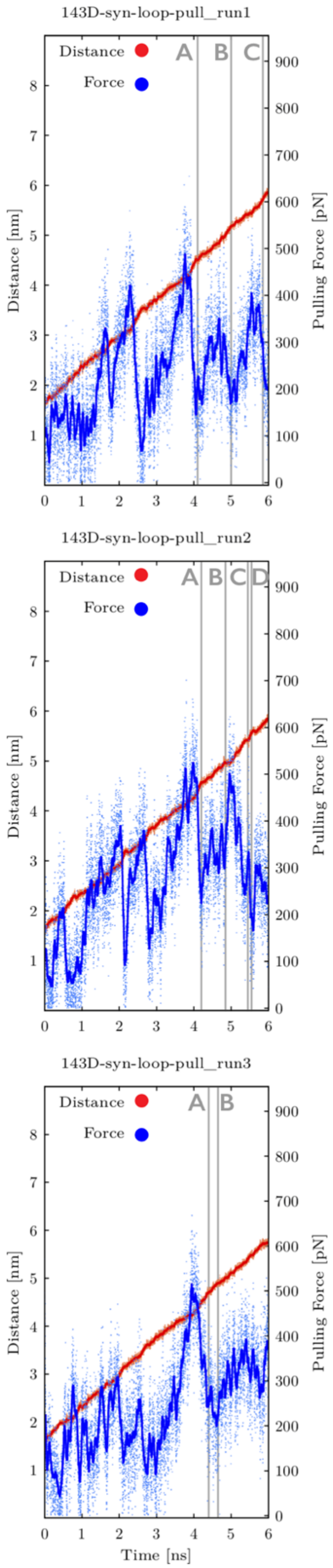

Figure S8A: Time evolution of distance between pulling centers and pulling force during three independent *fast pulling* simulations of 143D$_{syn_loop\text{-}pull}$ GQ system (see legend of Figure S1A for

more details). See Figures S8B and S8C for inspection of structures corresponding to main structural events.

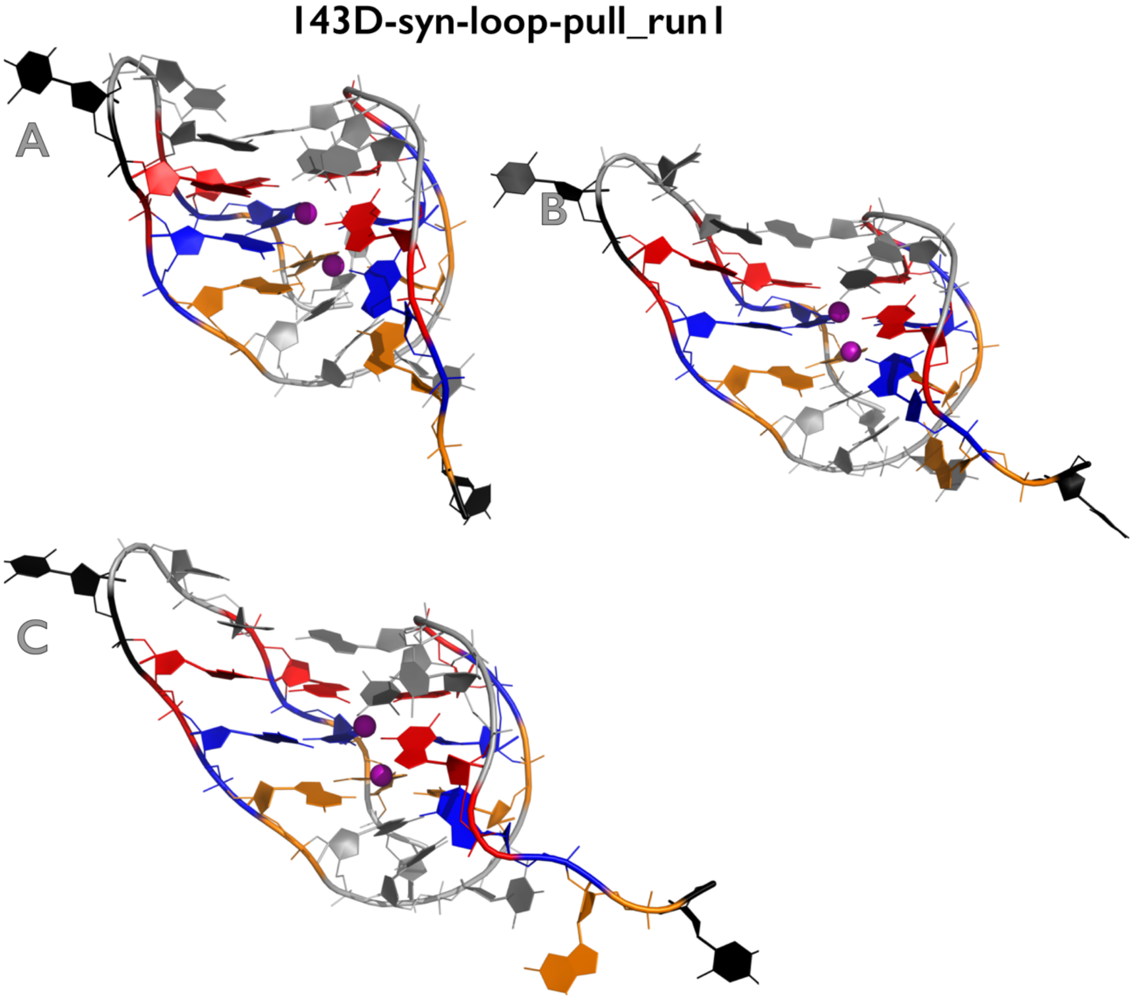

Figure S8B: Most important structural events during first independent *fast pulling* simulation of $143D_{syn\_loop\text{-}pull}$ GQ system. See legend of Figure S1B for more details.

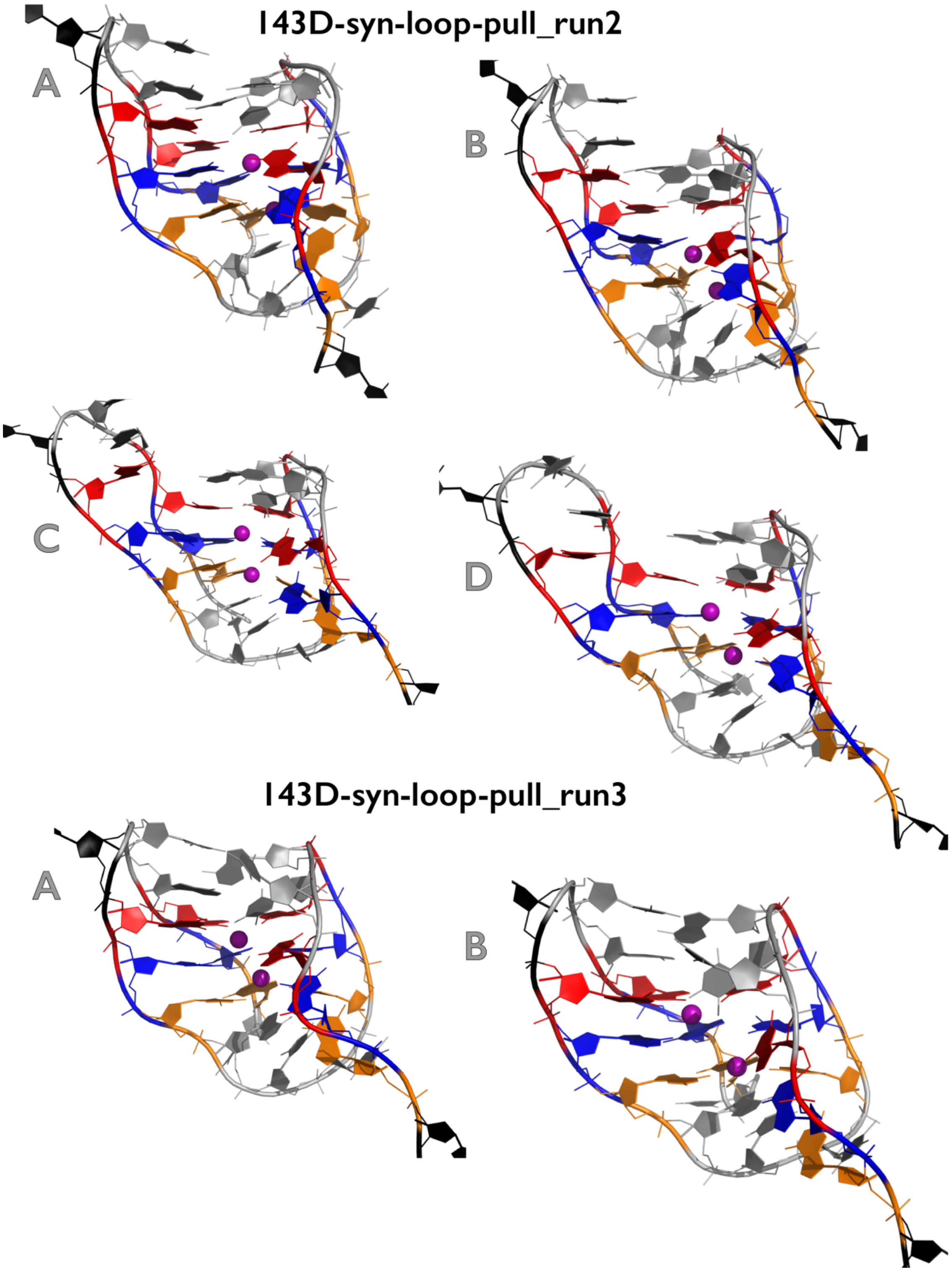

Figure S8C: Most important structural events during second and third independent *fast pulling* simulations of $143D_{syn\_loop-pull}$ GQ system. See legend of Figure S1B for more details.

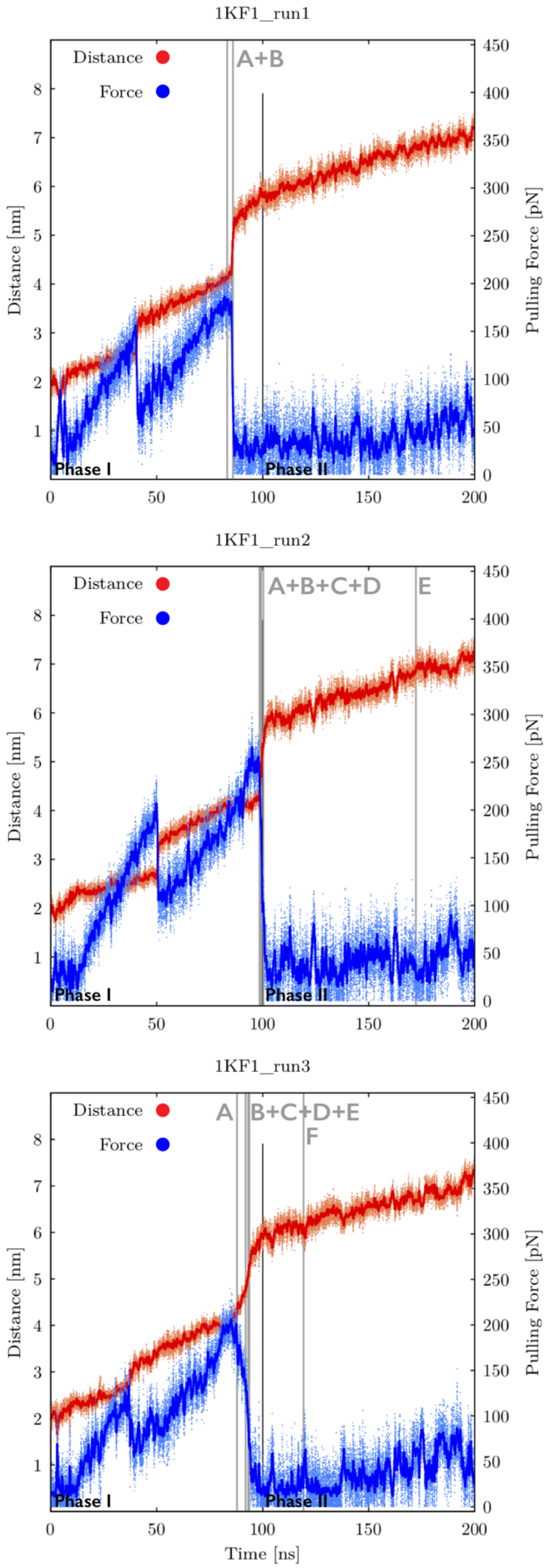

Figure S9A: Time evolution of distance between pulling centers and pulling force during three independent *slow zig-zag pulling* simulations of 1KF1 GQ system. Snapshots were saved every 5 ps and plots are showing both instantaneous values (orange and light-blue dots for distance and

force, respectively) and smoothing, i.e., averaging over 100 consecutive snapshots (red and blue lines for distance and force, respectively). Pulling phases are marked (see Methods in the main text for details) and main structural events are highlighted as grey vertical lines with labels (capital letters). See Figures S9B-S9D for inspection of structures corresponding to main structural events. Note that first major drops of the pulling force before the GQ unfolding event "A" (and notable prolongation of end-to-end distances) are connected with repositioning of terminal T residues.

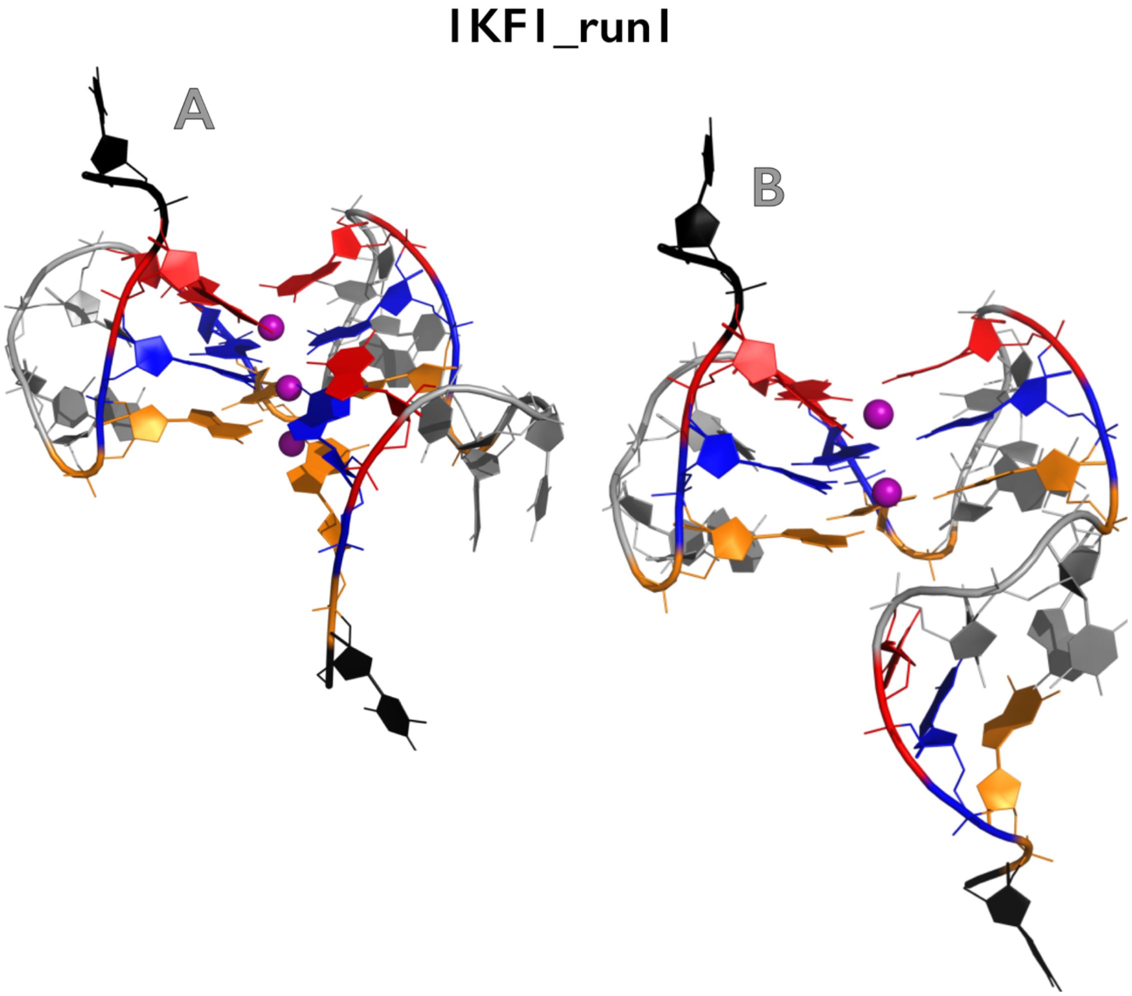

Figure S9B: Most important structural events during first independent *slow zig-zag pulling* simulation of 1KF1 GQ system. See legend of Figure S1B for more details.

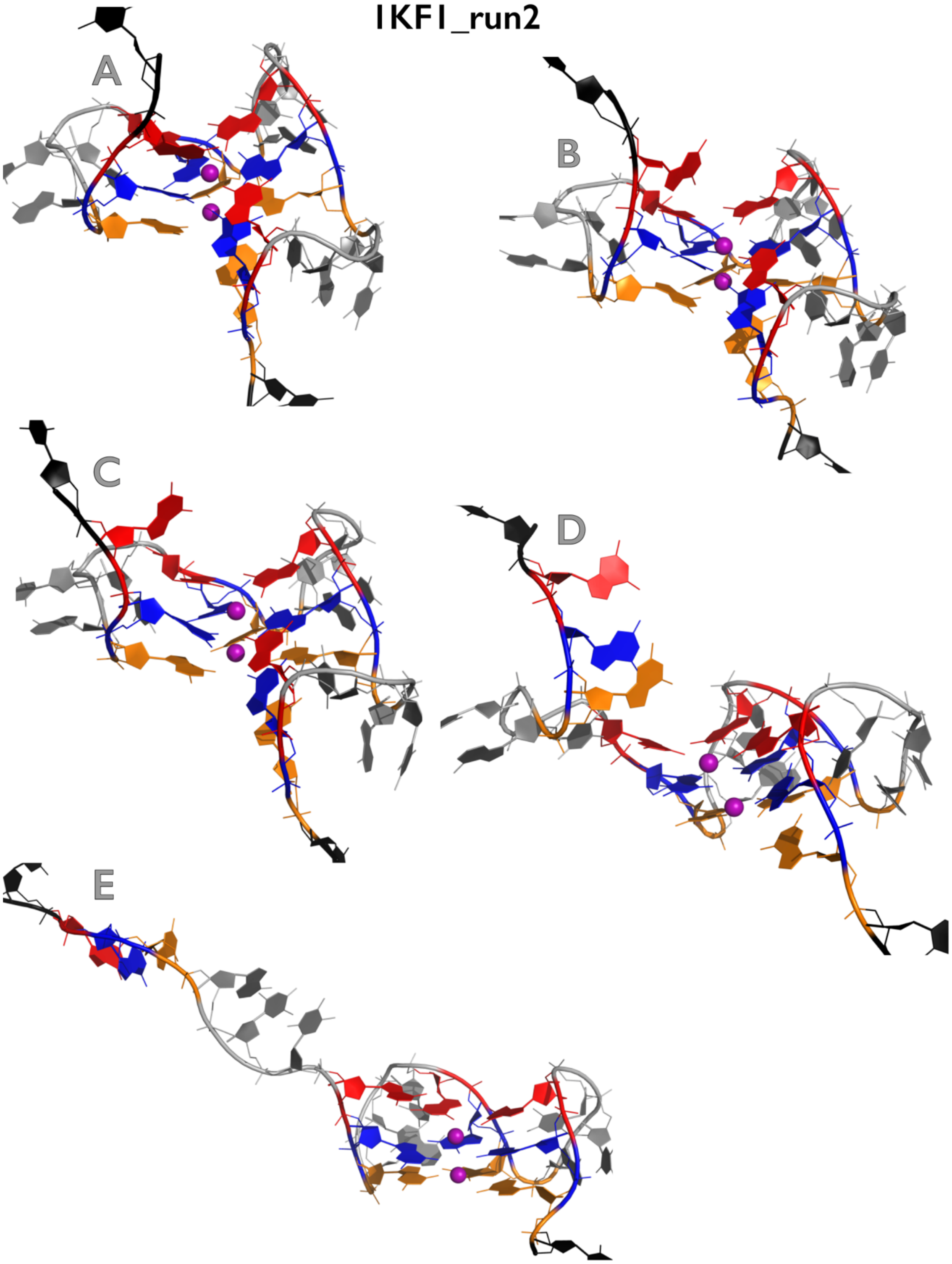

Figure S9C: Most important structural events during second independent *slow zig-zag pulling* simulation of 1KF1 GQ system. See legend of Figure S1B for more details.

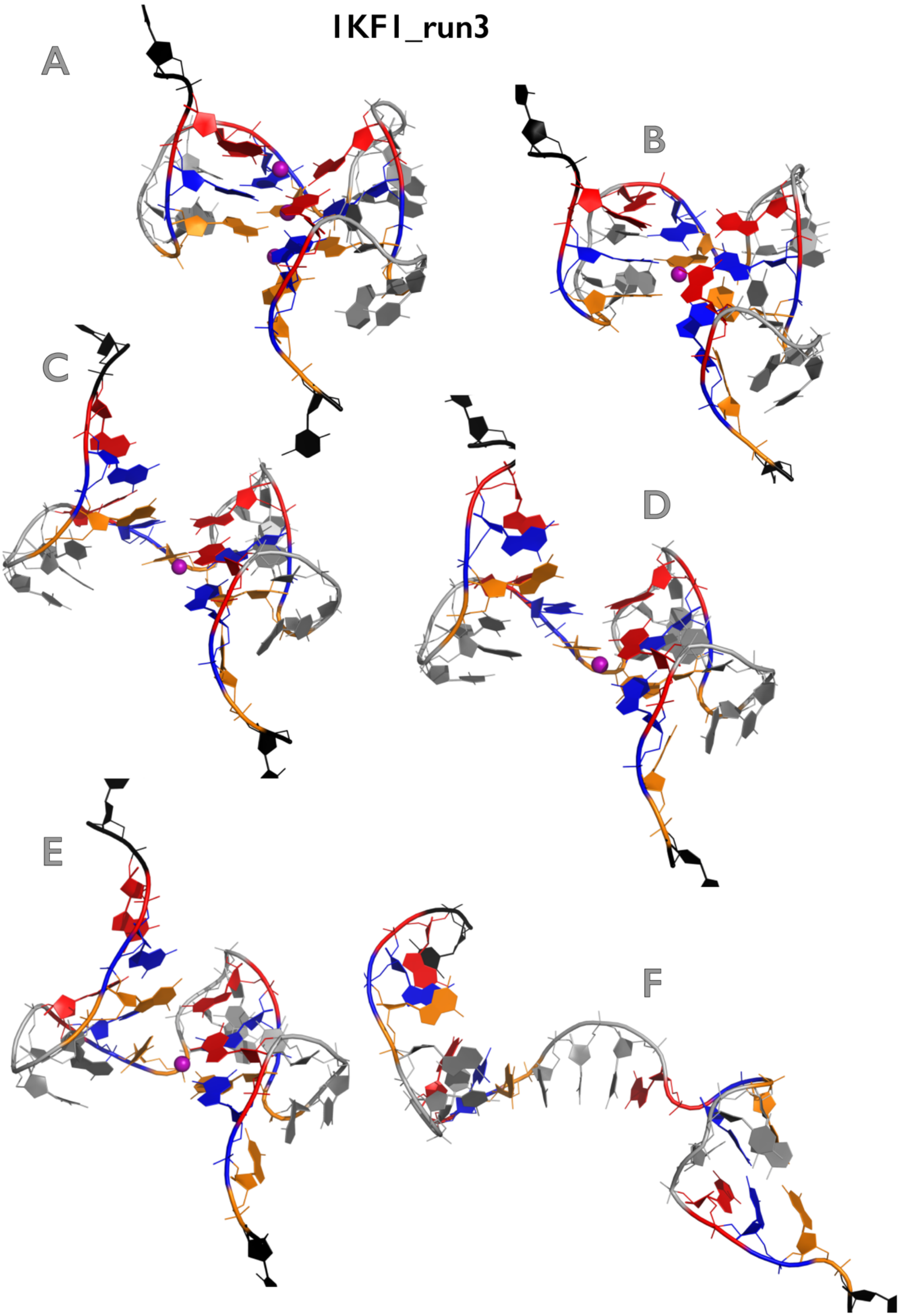

Figure S9D: Most important structural events during third independent *slow zig-zag pulling* simulation of 1KF1 GQ system. See legend of Figure S1B for more details.

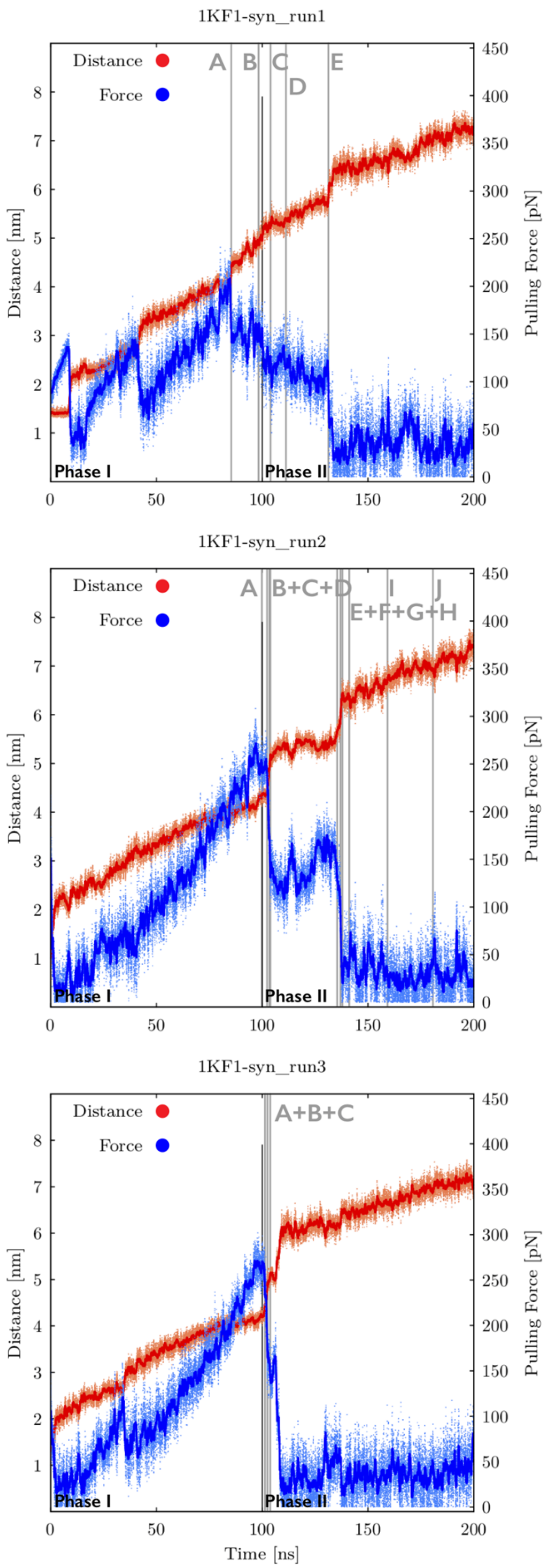

Figure S10A: Time evolution of distance between pulling centers and pulling force during three independent *slow zig-zag pulling* simulations of $1KF1_{syn}$ GQ system (see legend of Figure S9A for

more details). See Figures S10B-S10D for inspection of structures corresponding to main structural events.

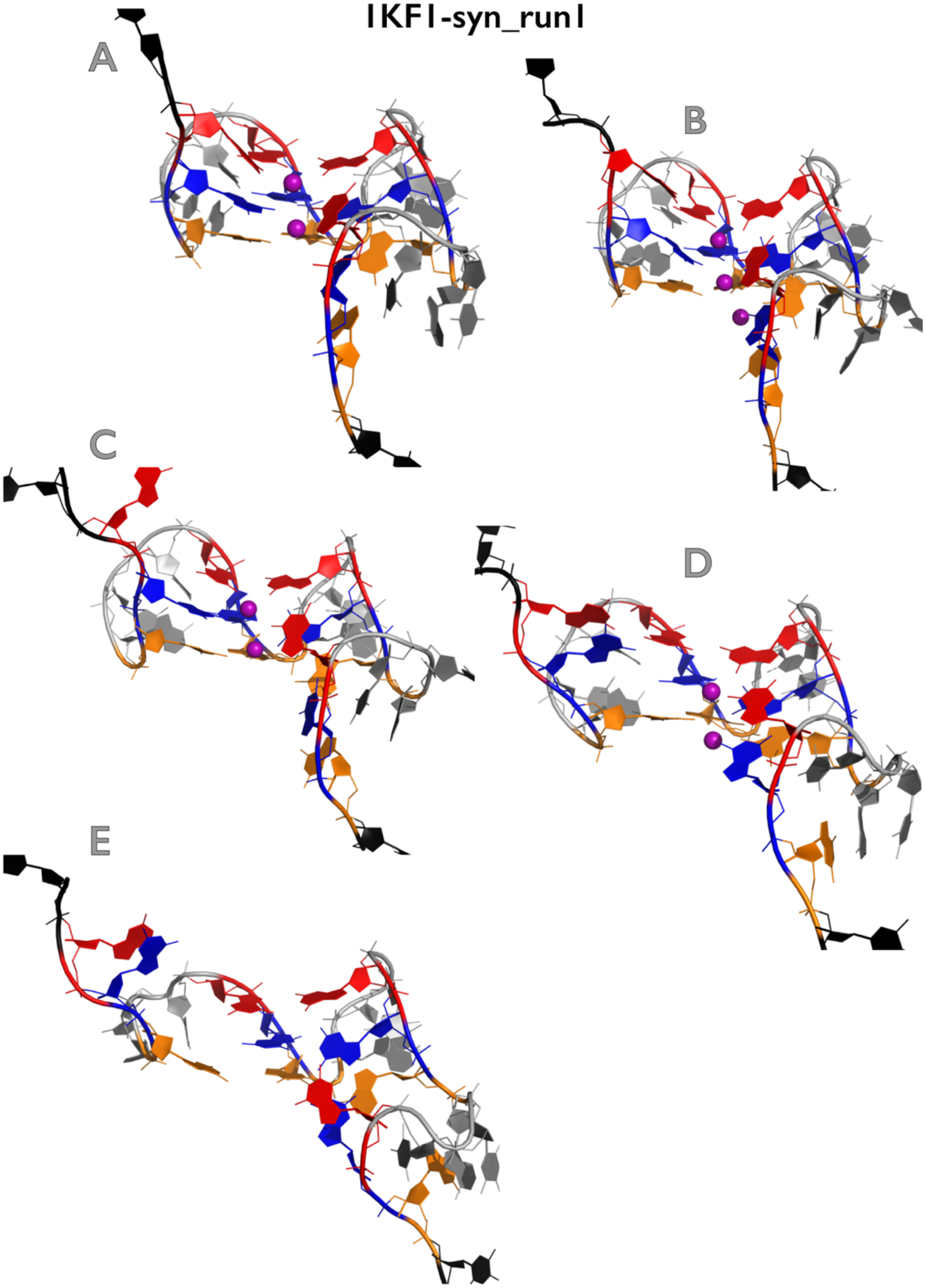

Figure S10B: Most important structural events during first independent *slow zig-zag pulling* simulation of $1KF1_{syn}$ GQ system. See legend of Figure S1B for more details.

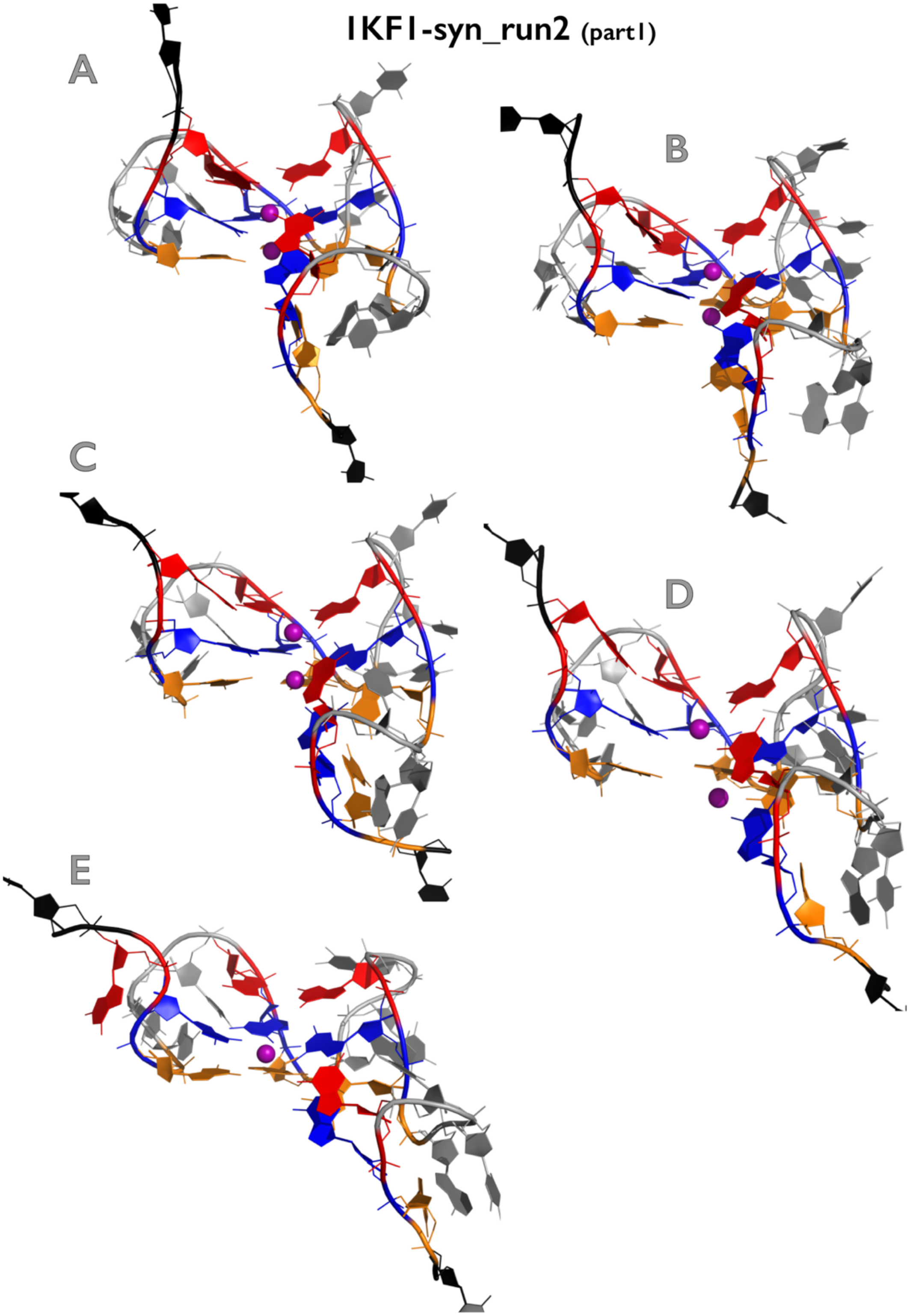

Figure continuing on the next page

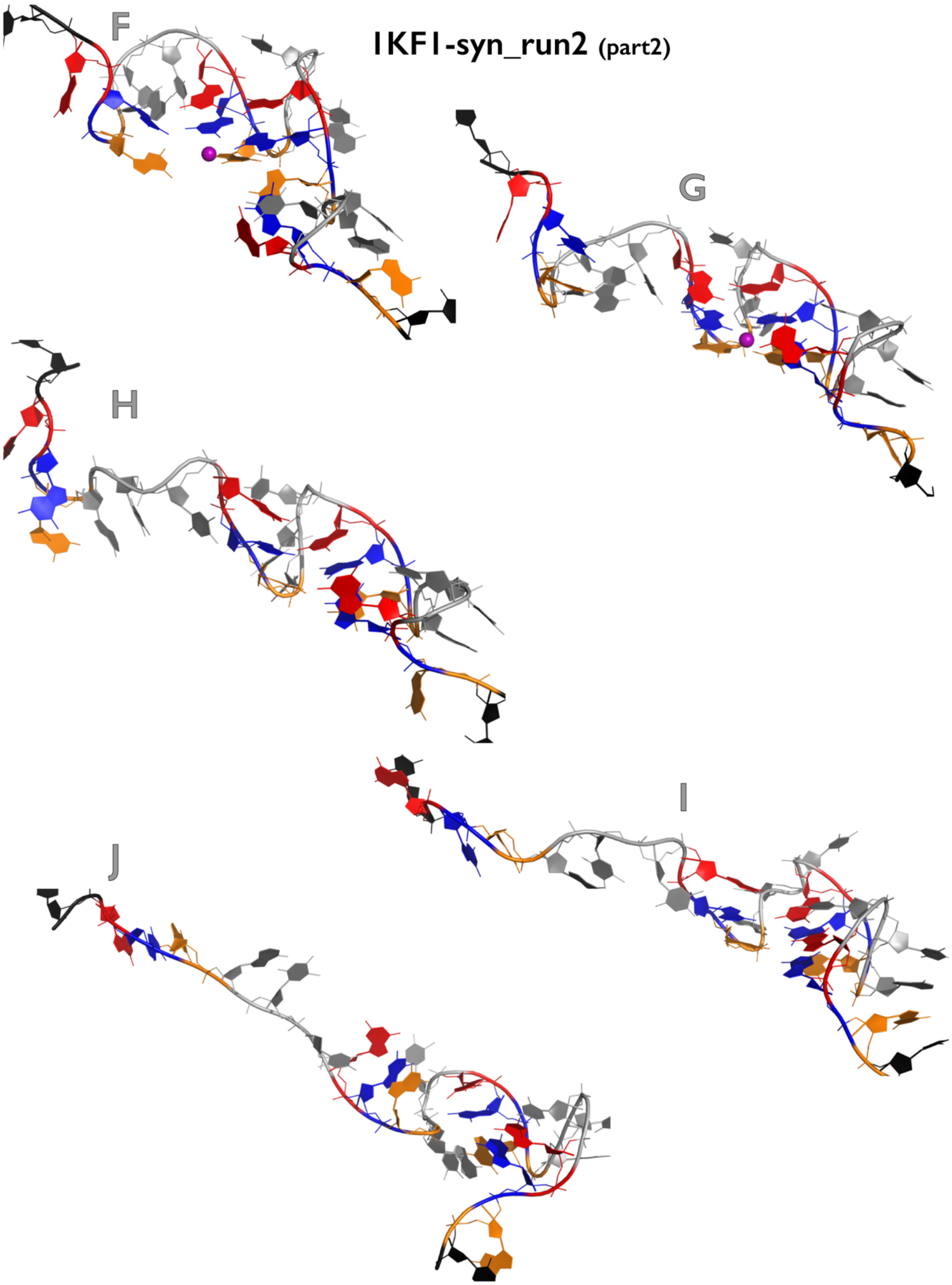

Figure S10C: Most important structural events during second independent *slow zig-zag pulling* simulation of $1KF1_{syn}$ GQ system. See legend of Figure S1B for more details.

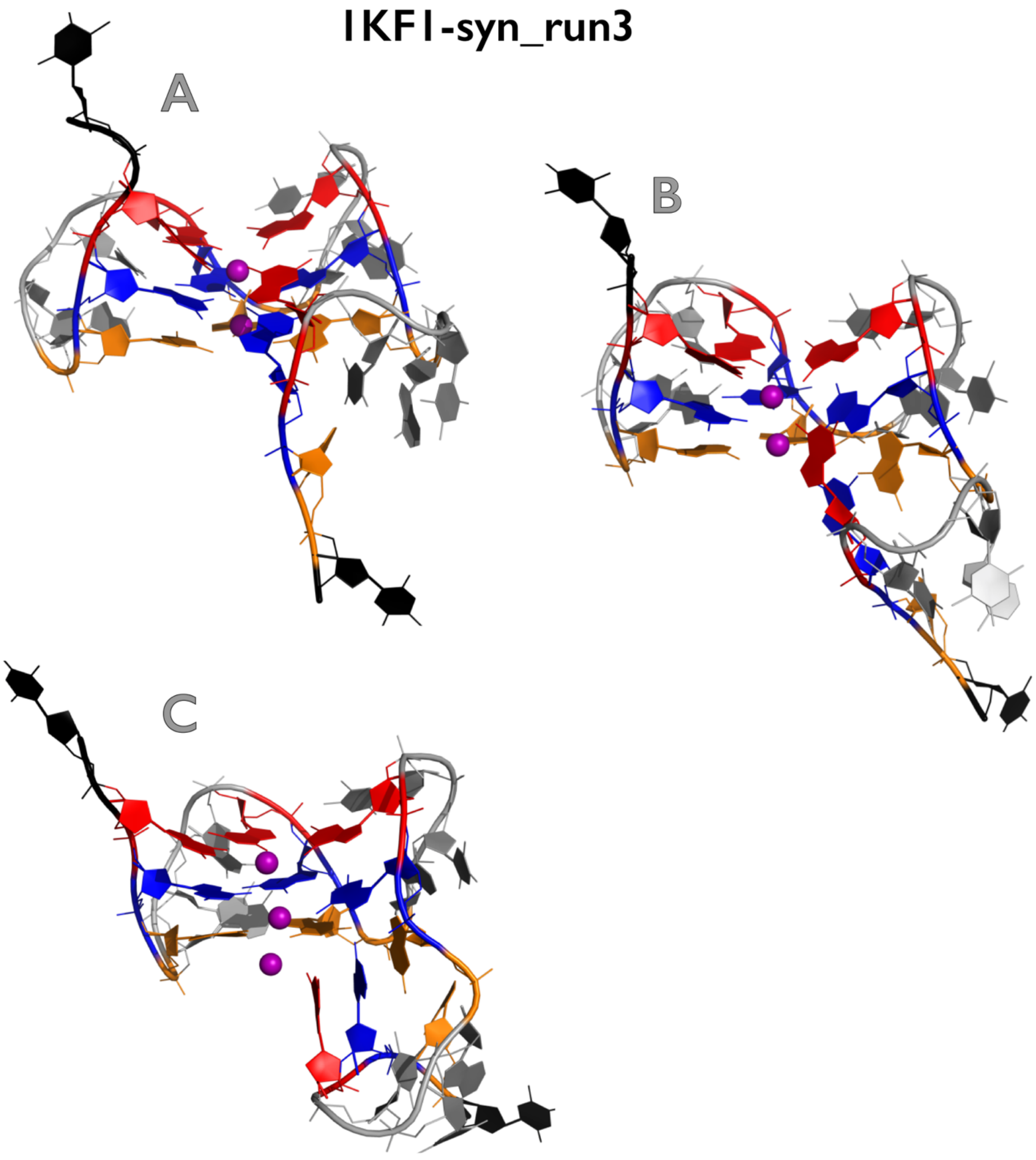

Figure S10D: Most important structural events during third independent *slow zig-zag pulling* simulation of $1KF1_{syn}$ GQ system. See legend of Figure S1B for more details.

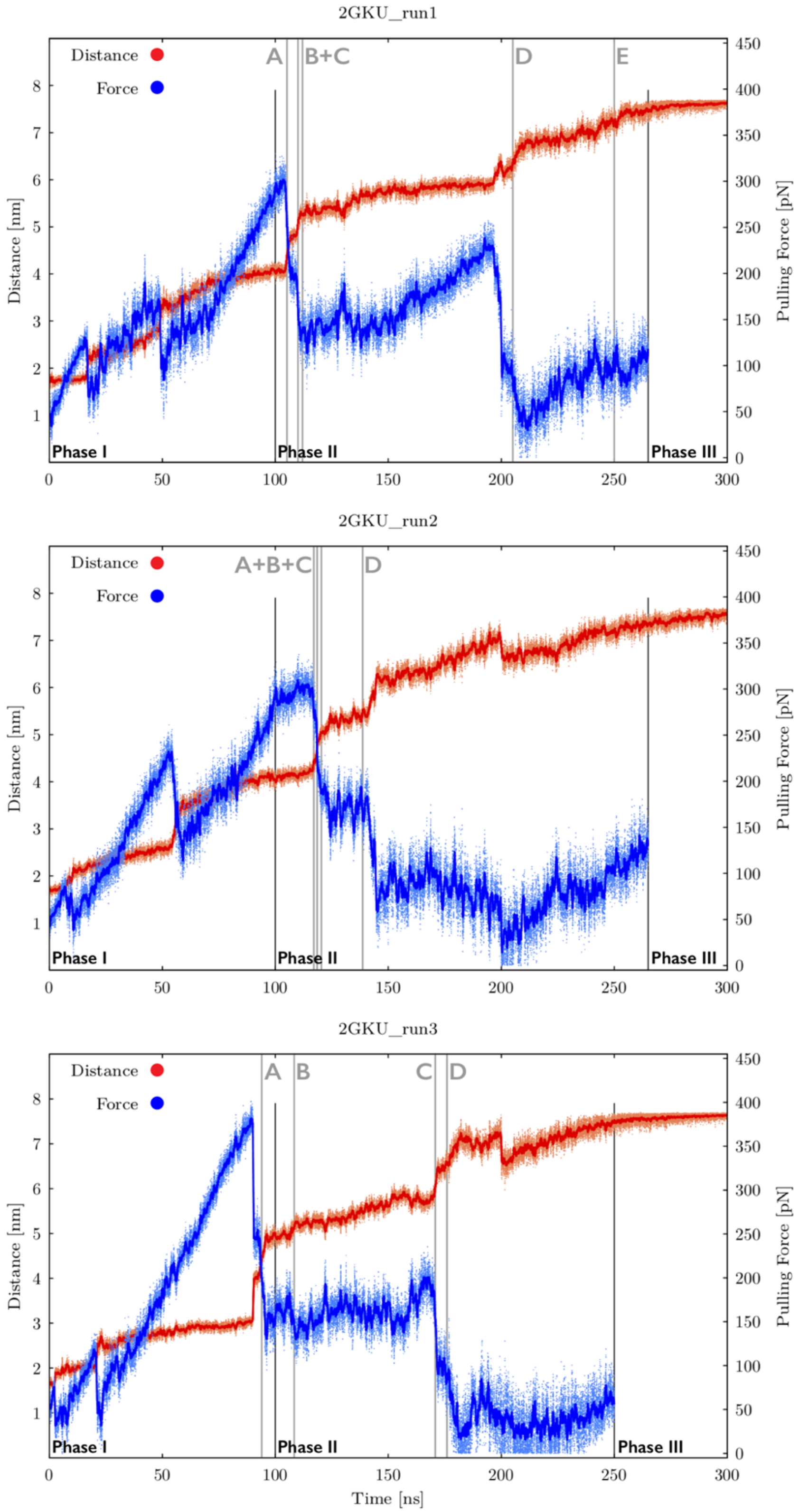

Figure S11A: Time evolution of distance between pulling centers and pulling force during three independent *slow zig-zag pulling* simulations of 2GKU GQ system (see legend of Figure S9A for

more details). See Figures S11B-S11D for inspection of structures corresponding to main structural events.

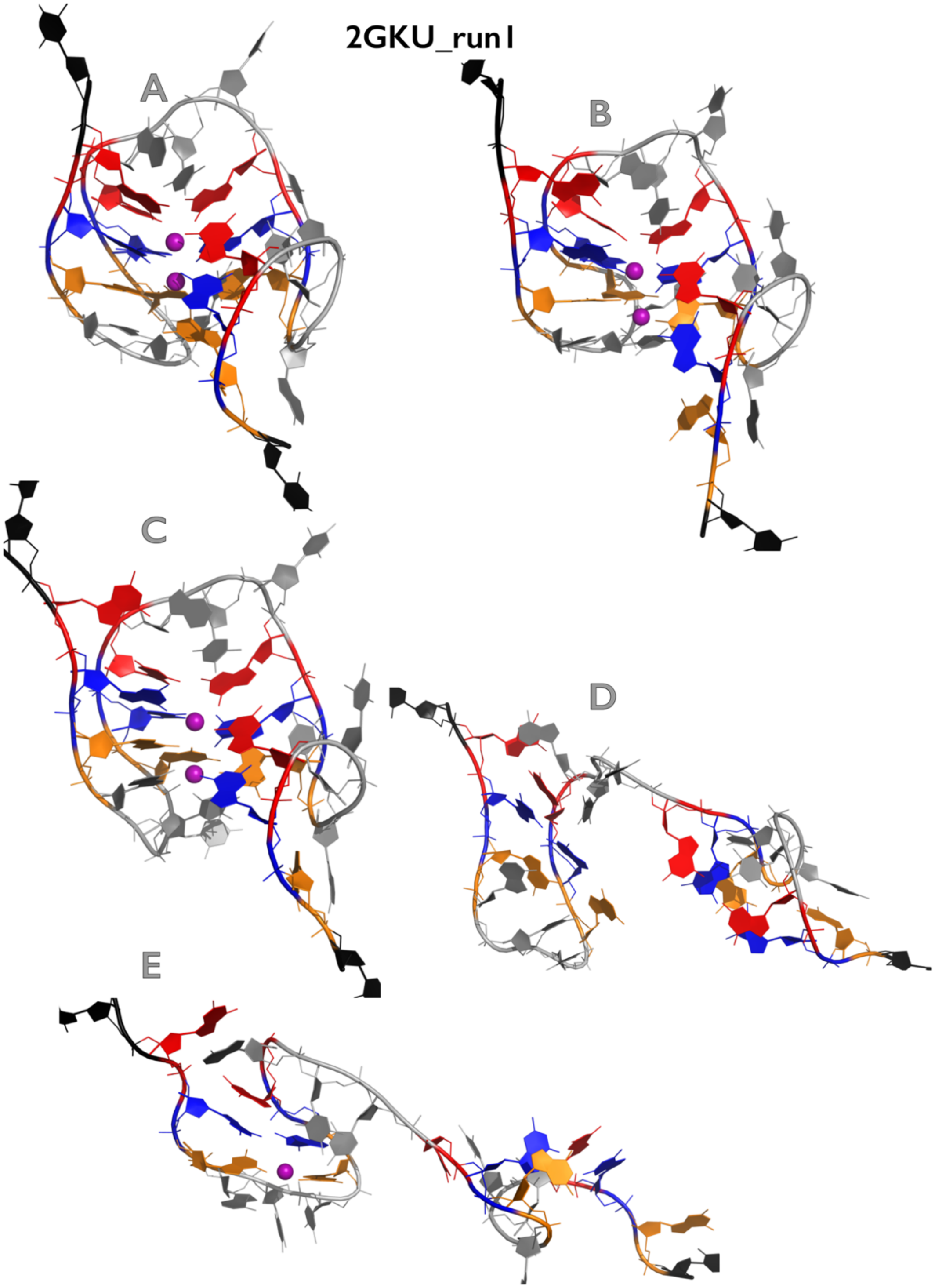

Figure S11B: Most important structural events during first independent *slow zig-zag pulling* simulation of 2GKU GQ system. See legend of Figure S1B for more details.

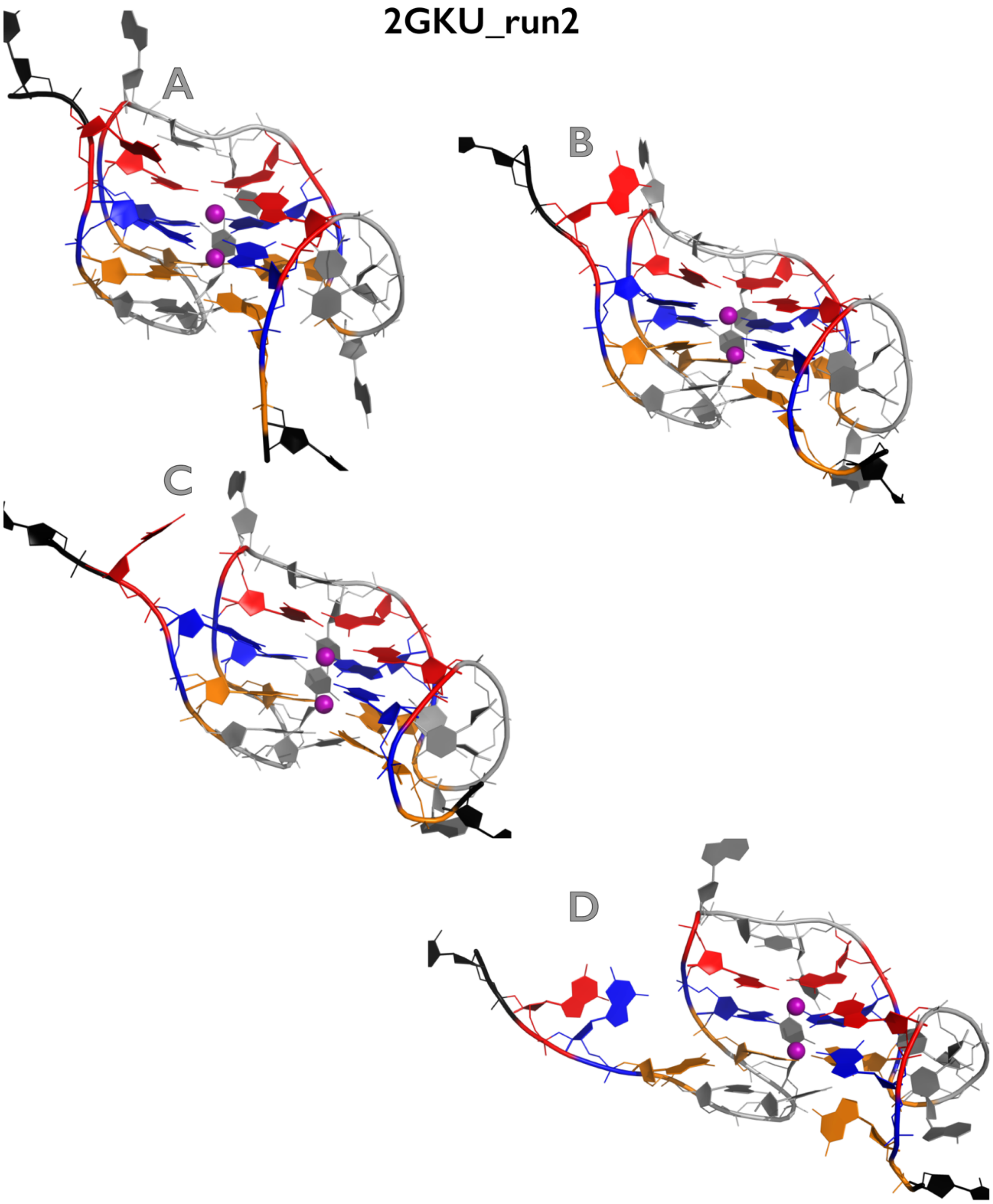

Figure S11C: Most important structural events during second independent *slow zig-zag pulling* simulation of 2GKU GQ system. See legend of Figure S1B for more details.

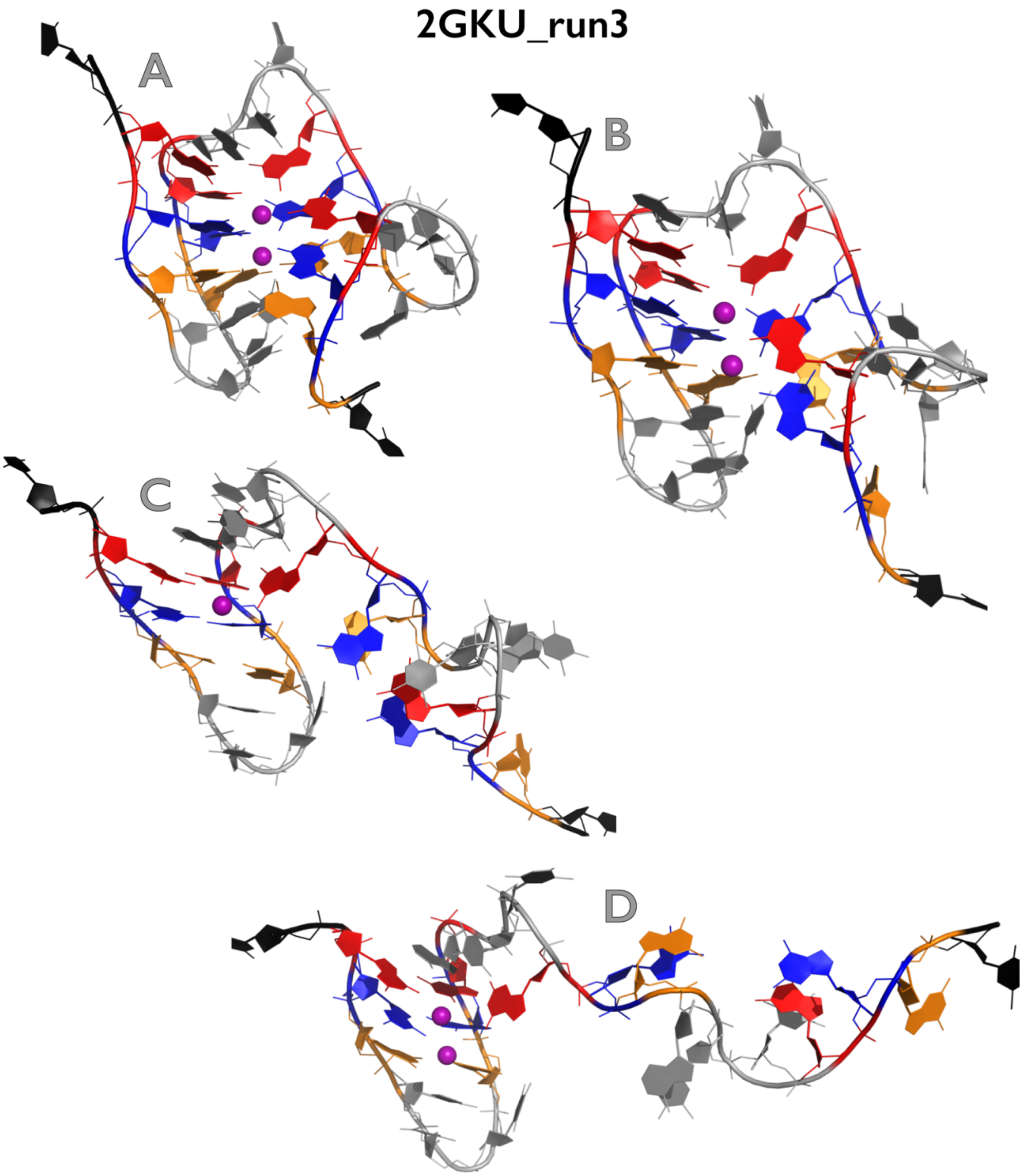

Figure S11D: Most important structural events during third independent *slow zig-zag pulling* simulation of 2GKU GQ system. See legend of Figure S1B for more details.

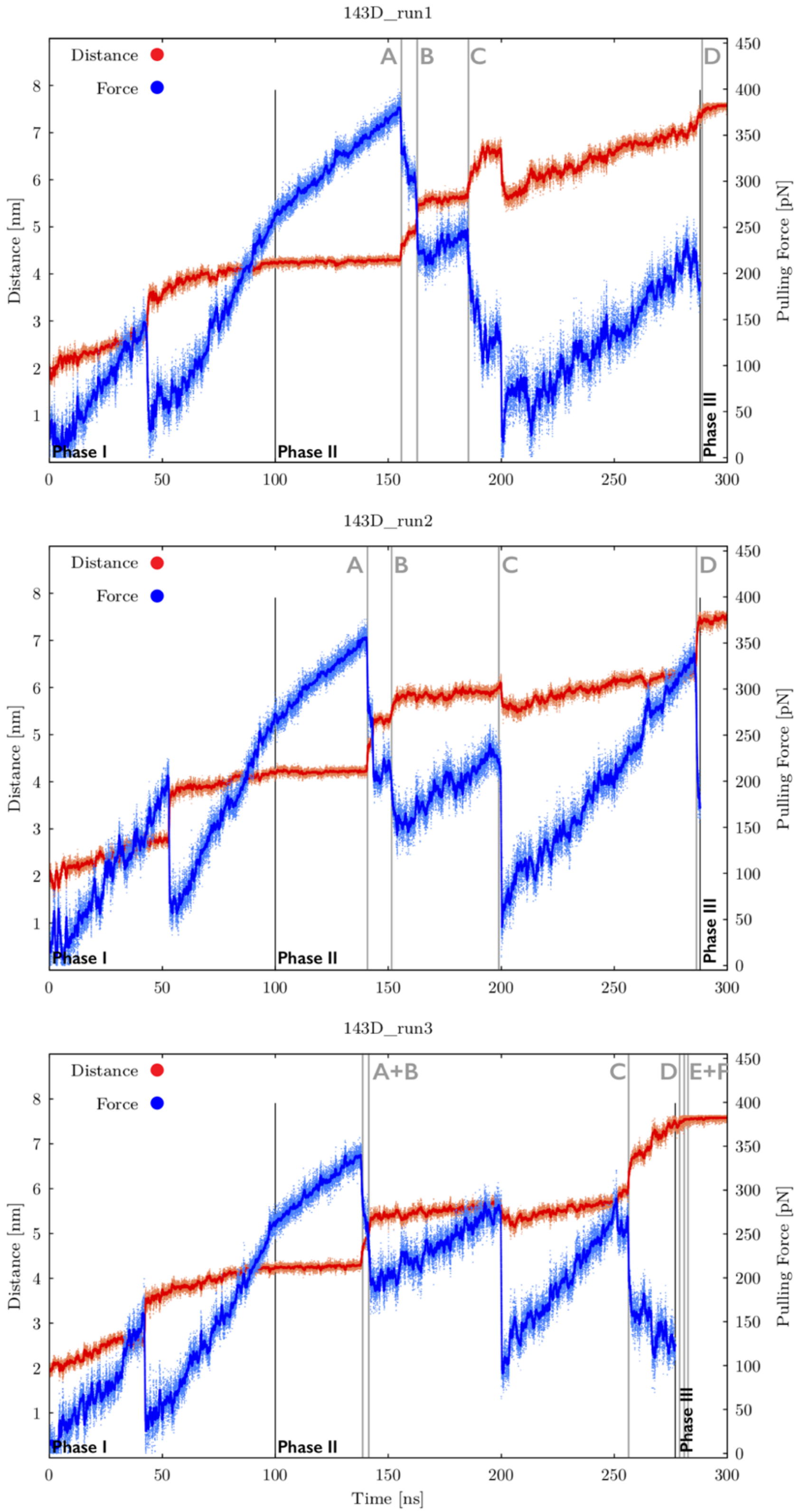

Figure S12A: Time evolution of distance between pulling centers and pulling force during three independent *slow zig-zag pulling* simulations of 143D GQ system (see legend of Figure S9A for

more details). See Figures S12B-S12D for inspection of structures corresponding to main structural events.

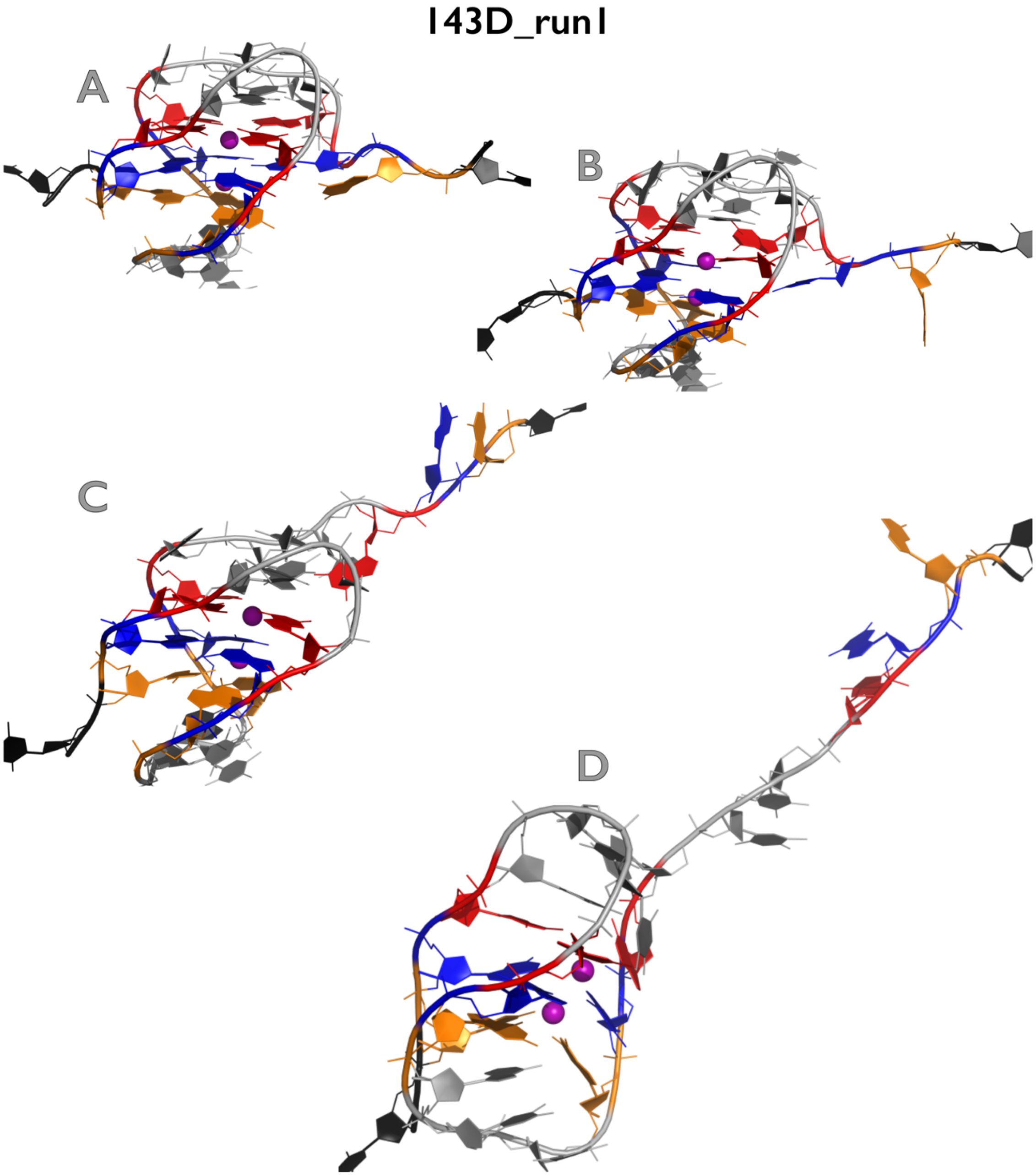

Figure S12B: Most important structural events during first independent *slow zig-zag pulling* simulation of 143D GQ system. See legend of Figure S1B for more details.

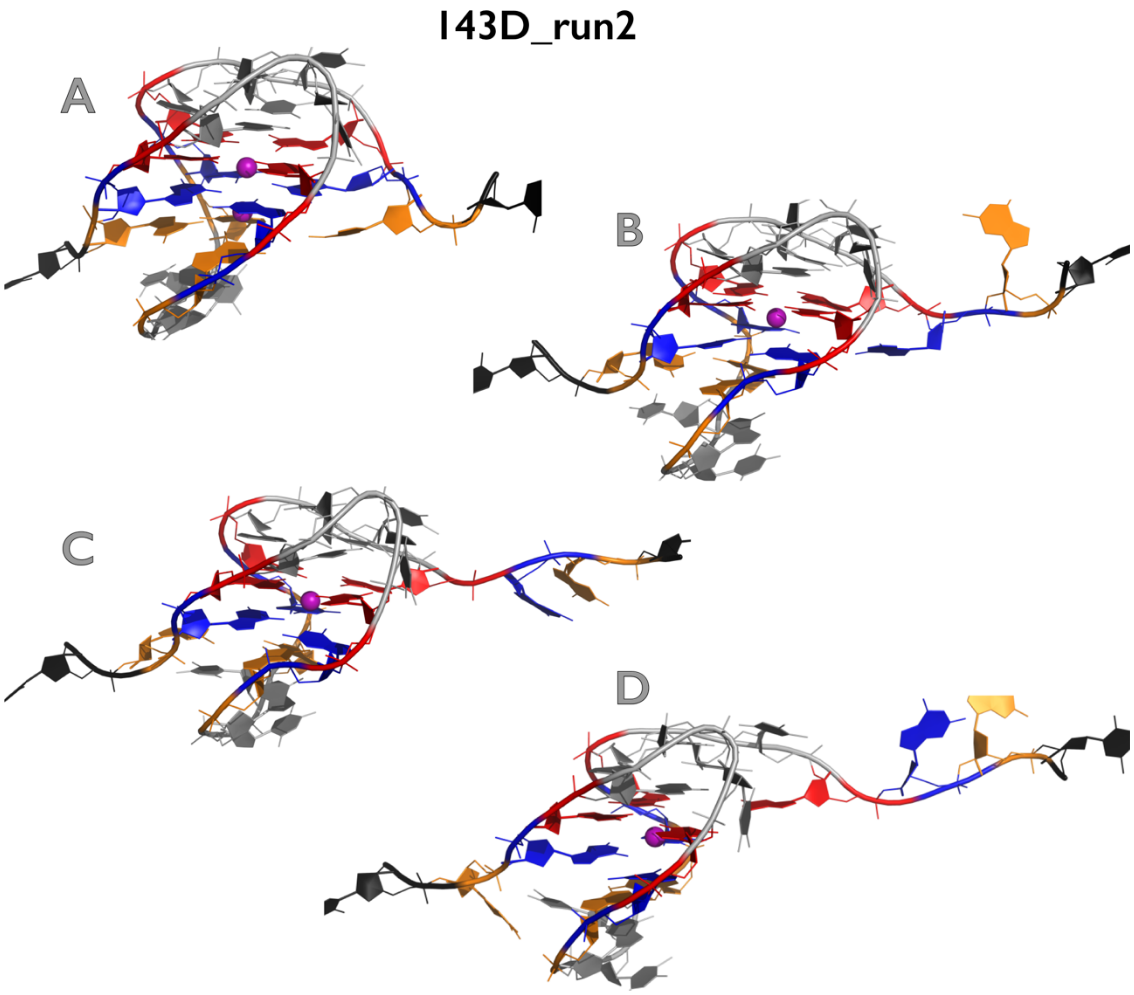

Figure S12C: Most important structural events during second independent *slow zig-zag pulling* simulation of 143D GQ system. See legend of Figure S1B for more details.

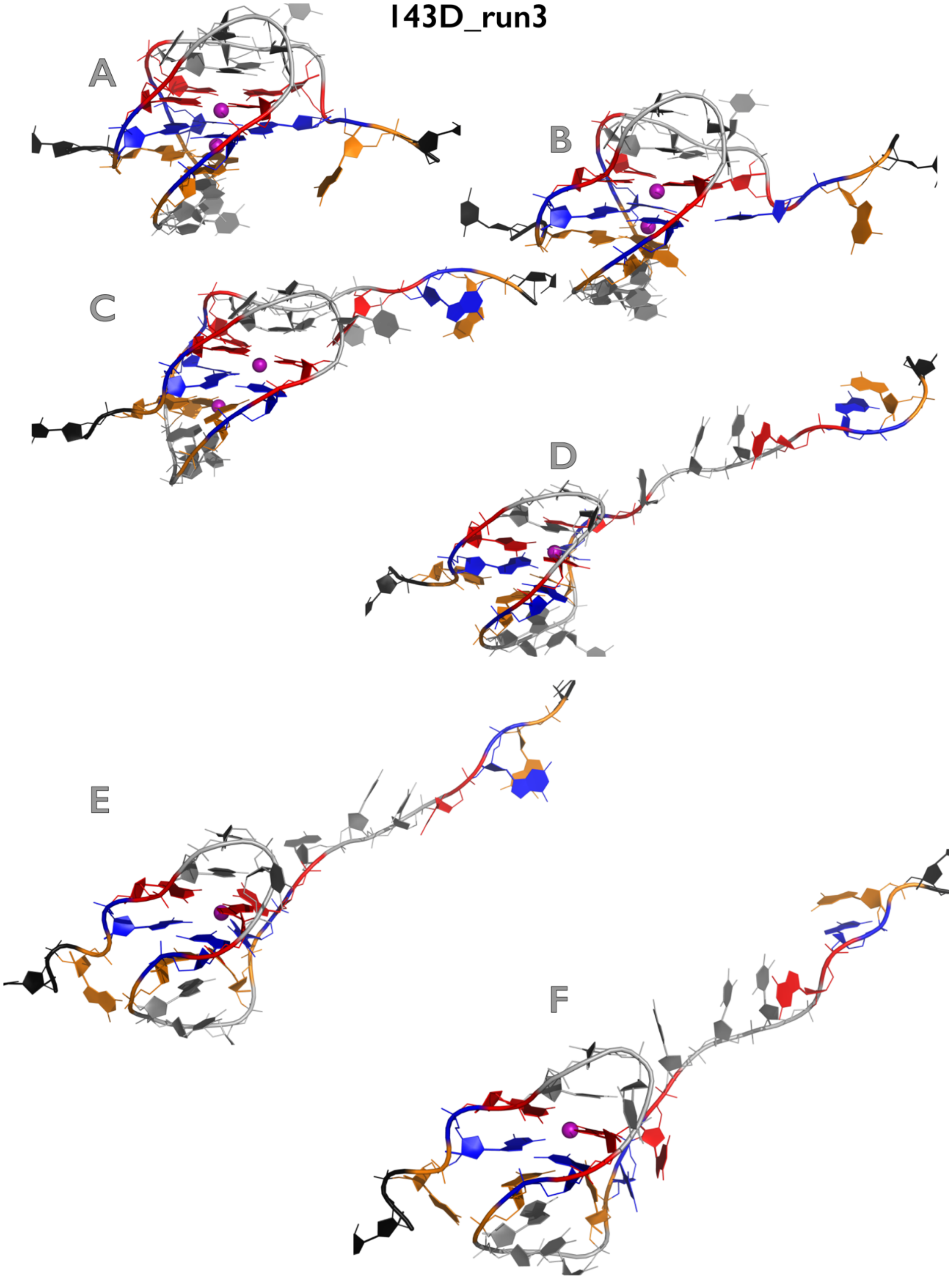

Figure S12D: Most important structural events during third independent *slow zig-zag pulling* simulation of 143D GQ system. See legend of Figure S1B for more details.

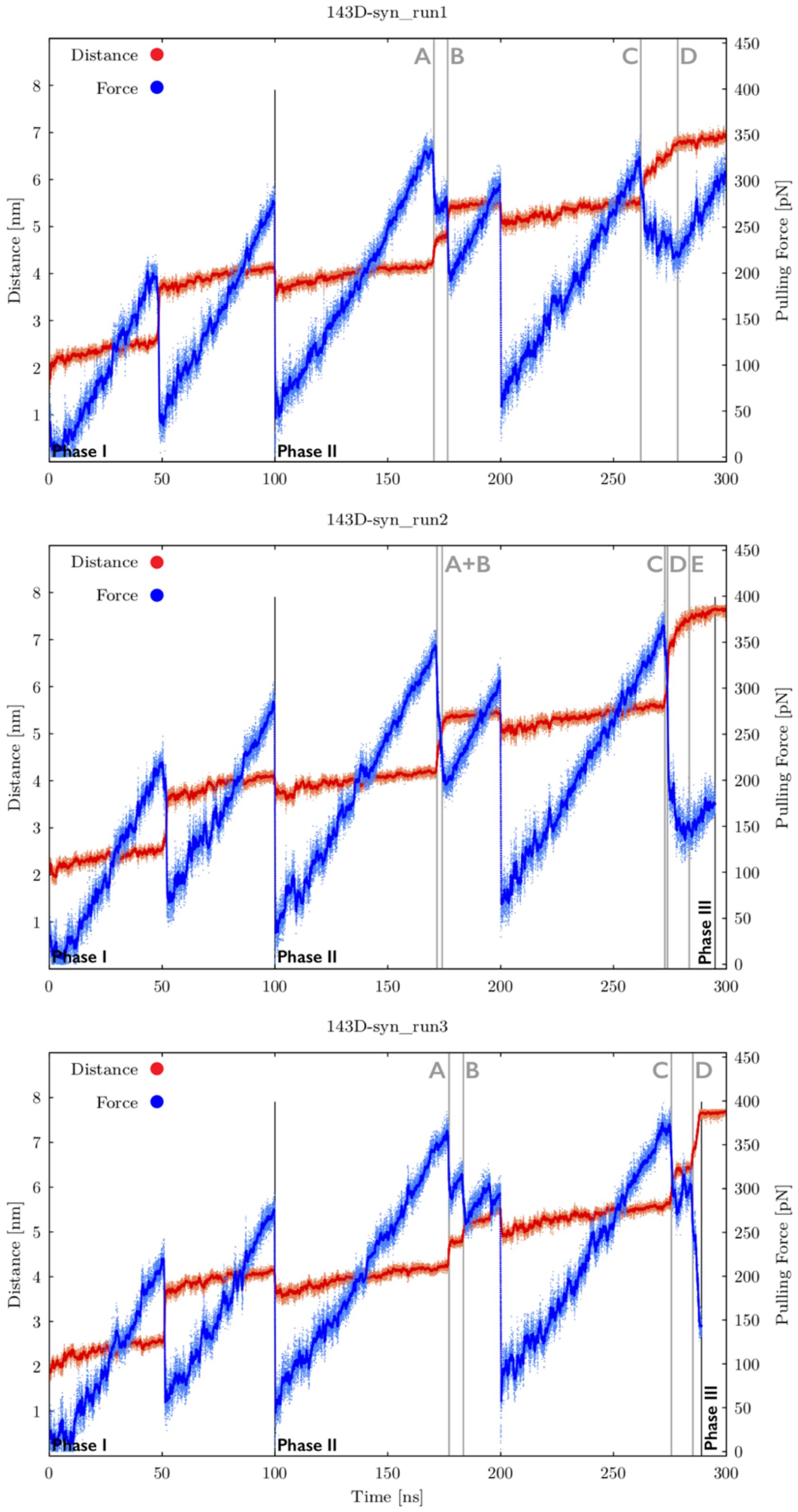

Figure S13A: Time evolution of distance between pulling centers and pulling force during three independent *slow zig-zag pulling* simulations of 143D$_{syn}$ GQ system (see legend of Figure S9A for

more details). See Figures S13B-S13D for inspection of structures corresponding to main structural events.

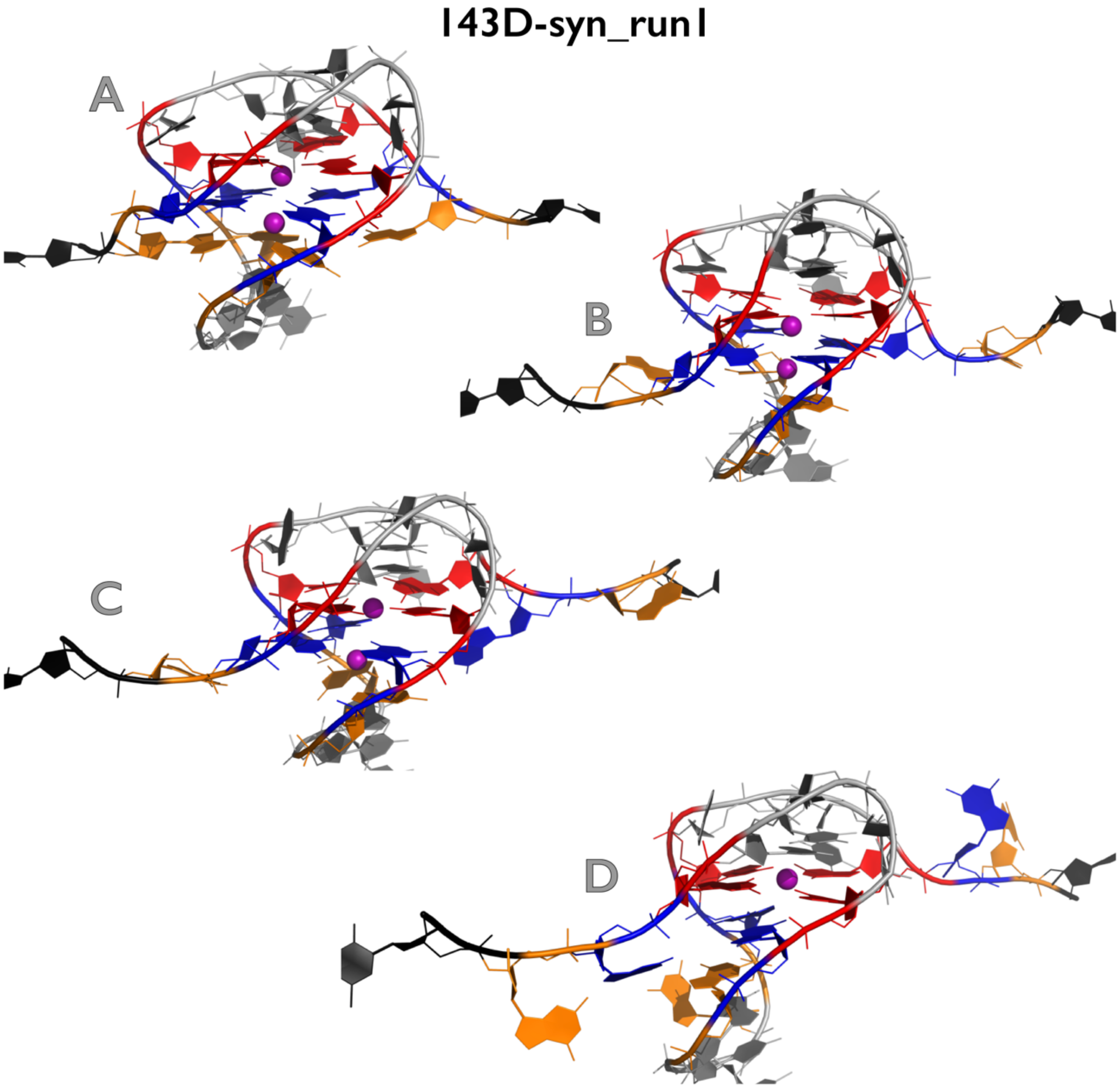

Figure S13B: Most important structural events during first independent *slow zig-zag pulling* simulation of $143D_{syn}$ GQ system. See legend of Figure S1B for more details.

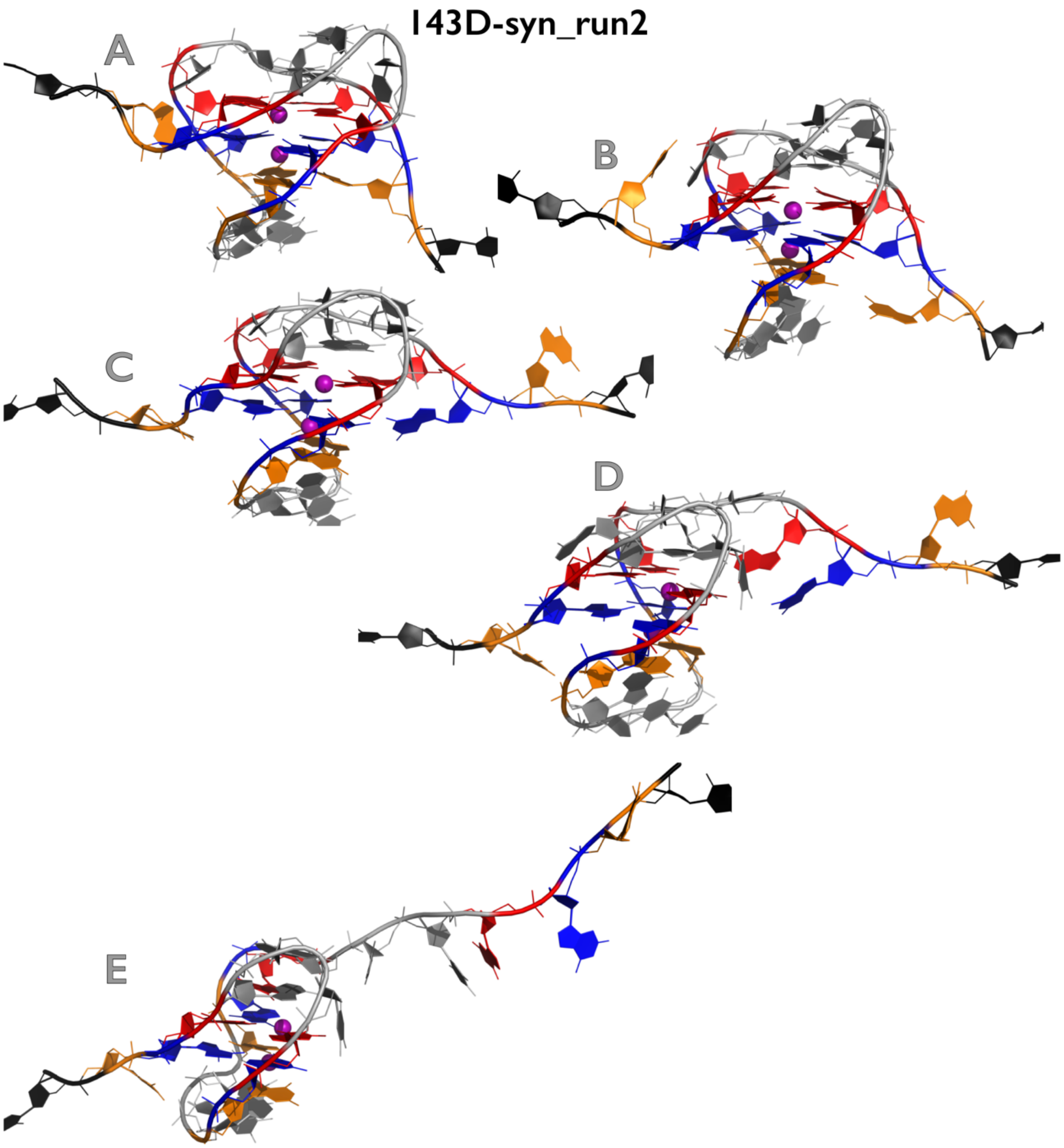

Figure S13C: Most important structural events during second independent *slow zig-zag pulling* simulation of 143D$_{syn}$ GQ system. See legend of Figure S1B for more details.

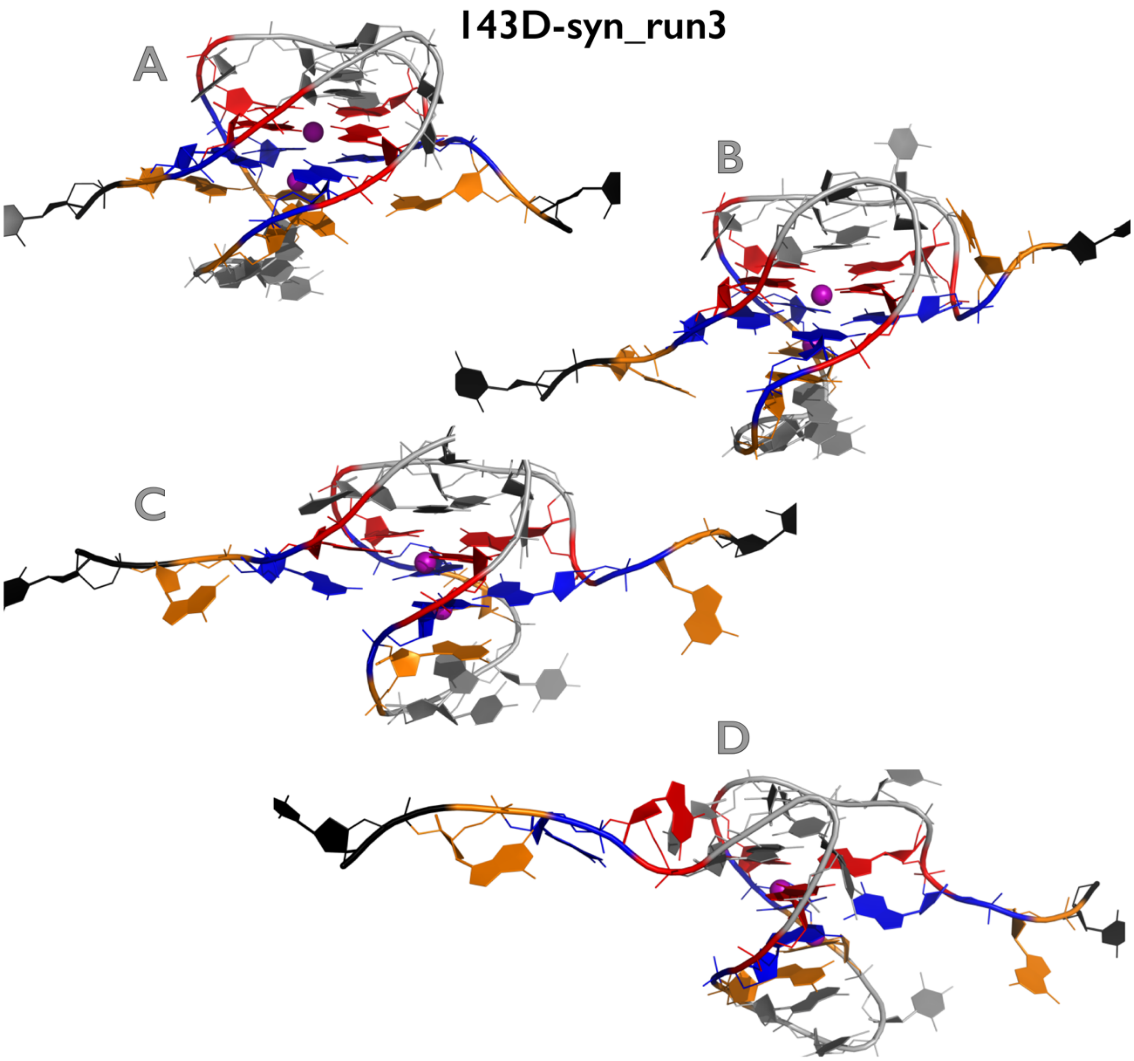

Figure S13D: Most important structural events during third independent *slow zig-zag pulling* simulation of $143D_{syn}$ GQ system. See legend of Figure S1B for more details.

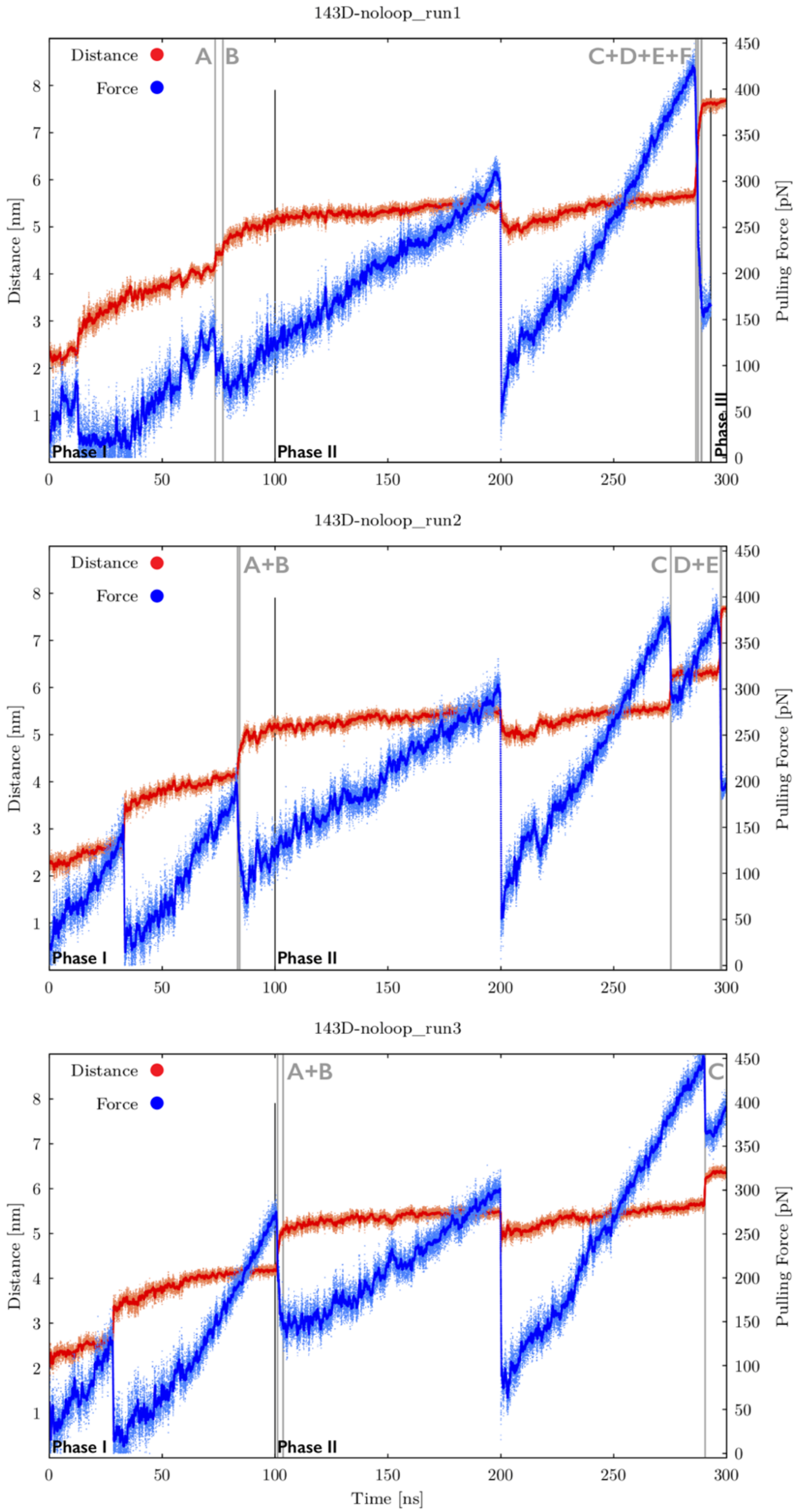

Figure S14A: Time evolution of distance between pulling centers and pulling force during three independent *slow zig-zag pulling* simulations of $143D_{noloop}$ GQ system (see legend of Figure S9A

for more details). See Figures S14B-S14D for inspection of structures corresponding to main structural events.

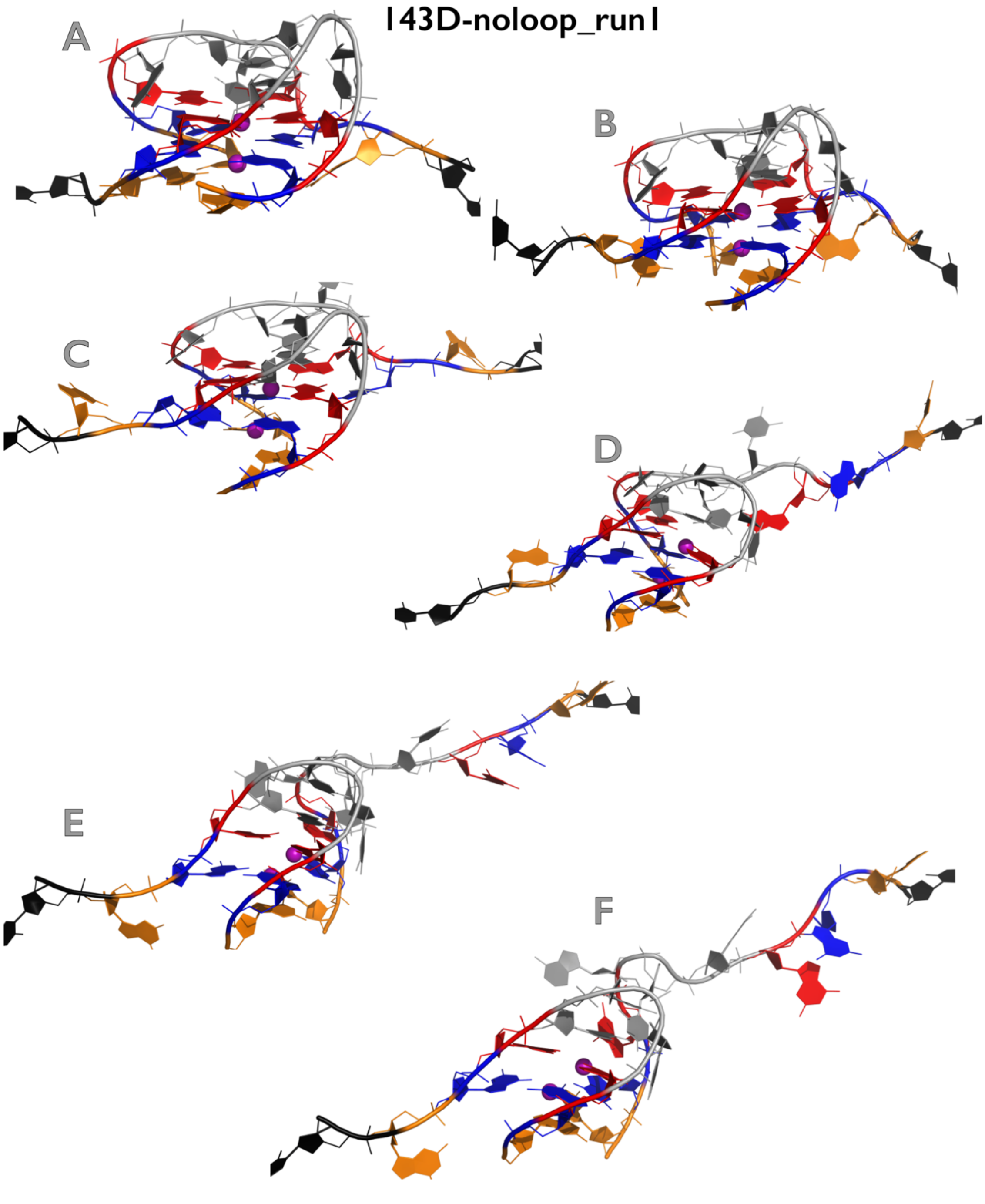

Figure S14B: Most important structural events during first independent *slow zig-zag pulling* simulation of 143D$_{noloop}$ GQ system. See legend of Figure S1B for more details.

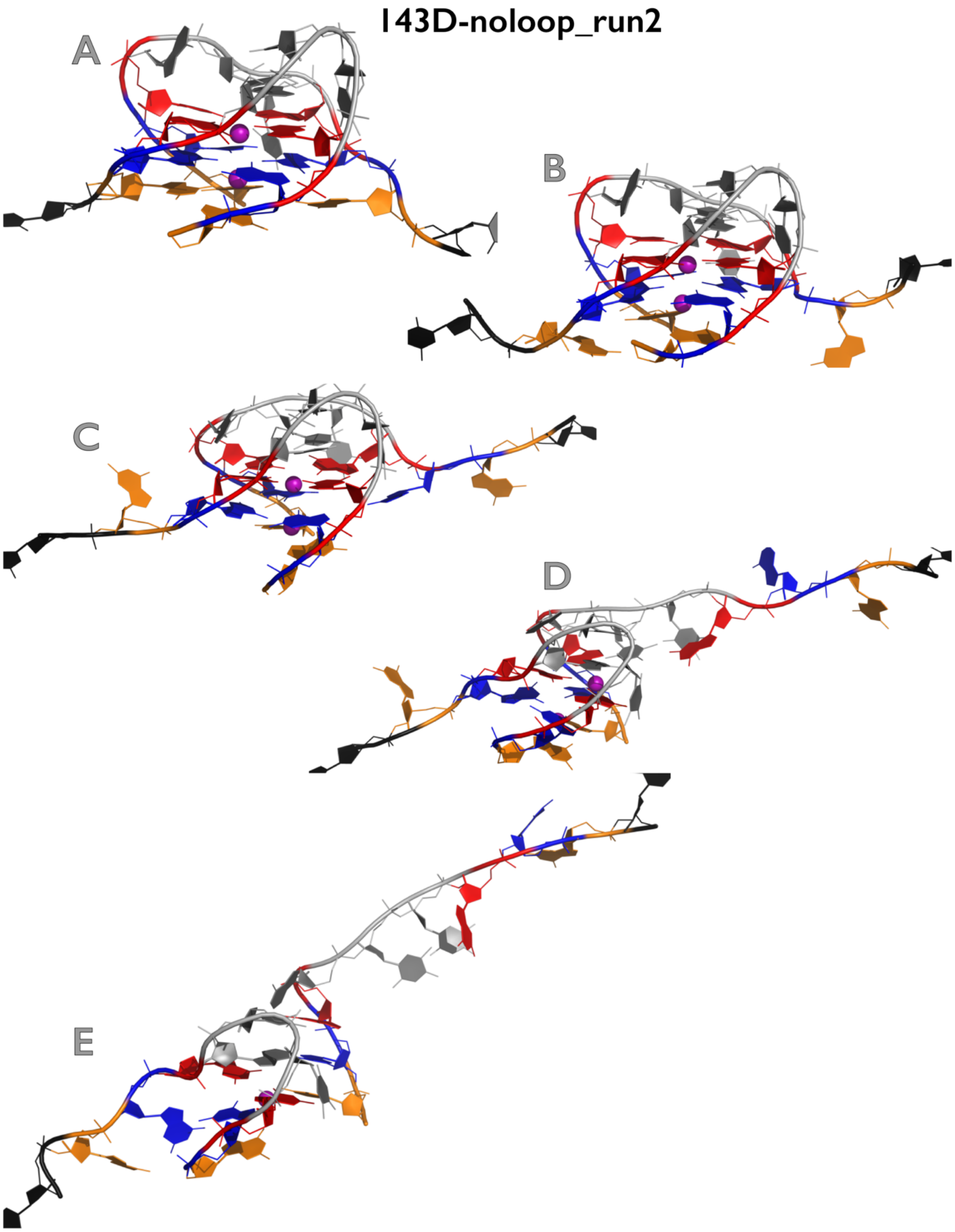

Figure S14C: Most important structural events during second independent *slow zig-zag pulling* simulation of $143D_{noloop}$ GQ system. See legend of Figure S1B for more details.

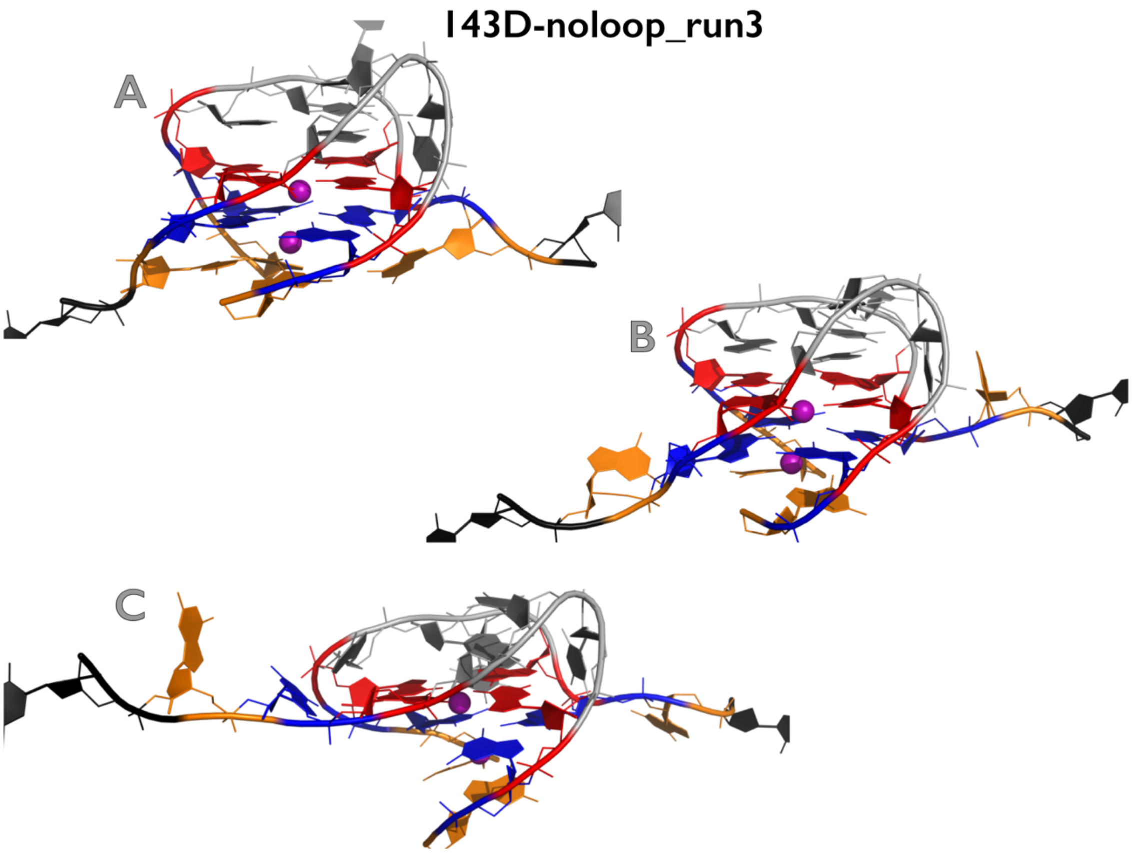

Figure S14D: Most important structural events during third independent *slow zig-zag pulling* simulation of 143D$_{noloop}$ GQ system. See legend of Figure S1B for more details.

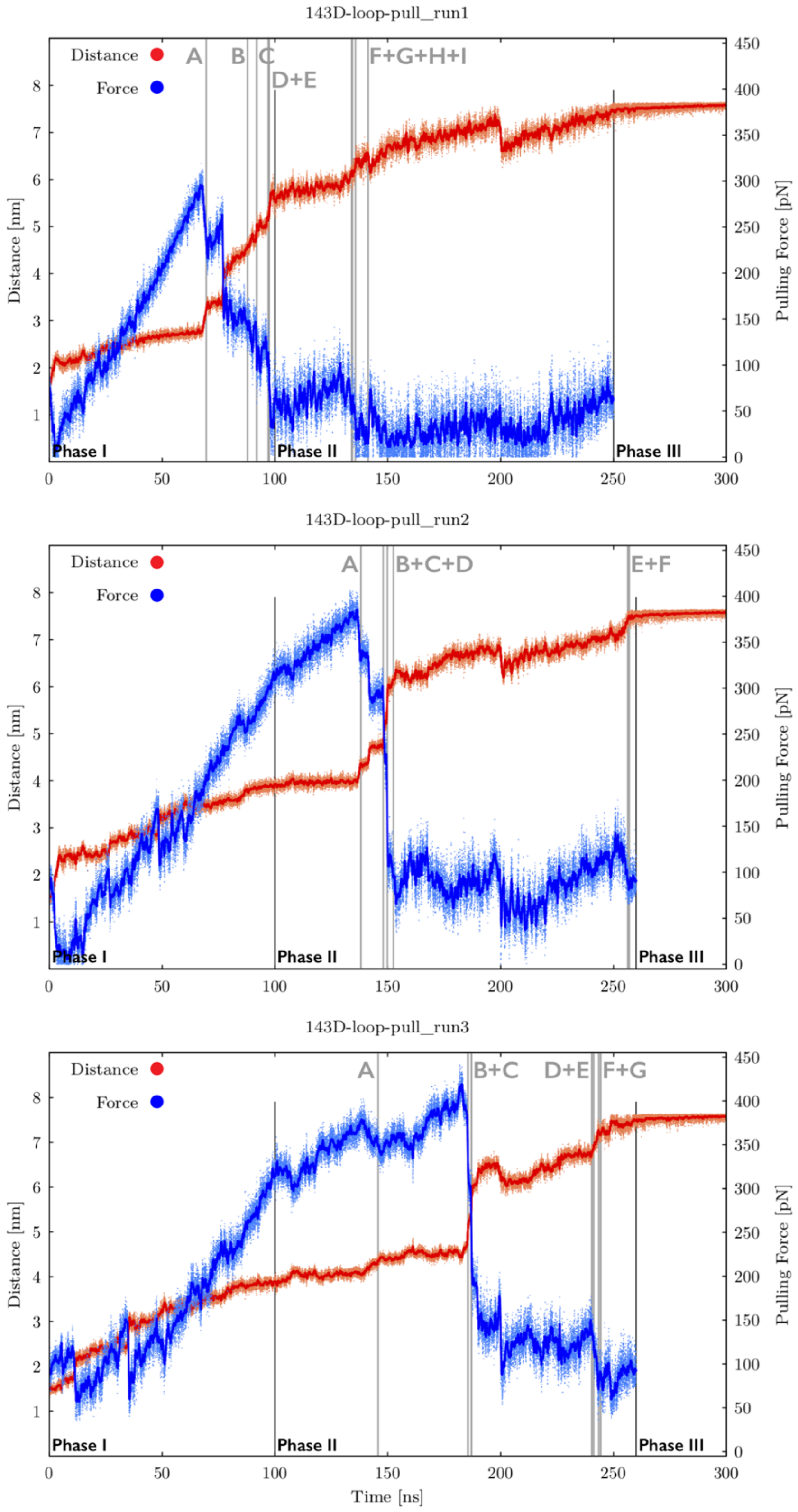

Figure S15A: Time evolution of distance between pulling centers and pulling force during three independent *slow zig-zag pulling* simulations of 143D$_{loop\text{-}pull}$ GQ system (see legend of Figure S9A

for more details). See Figures S15B-S15D for inspection of structures corresponding to main structural events.

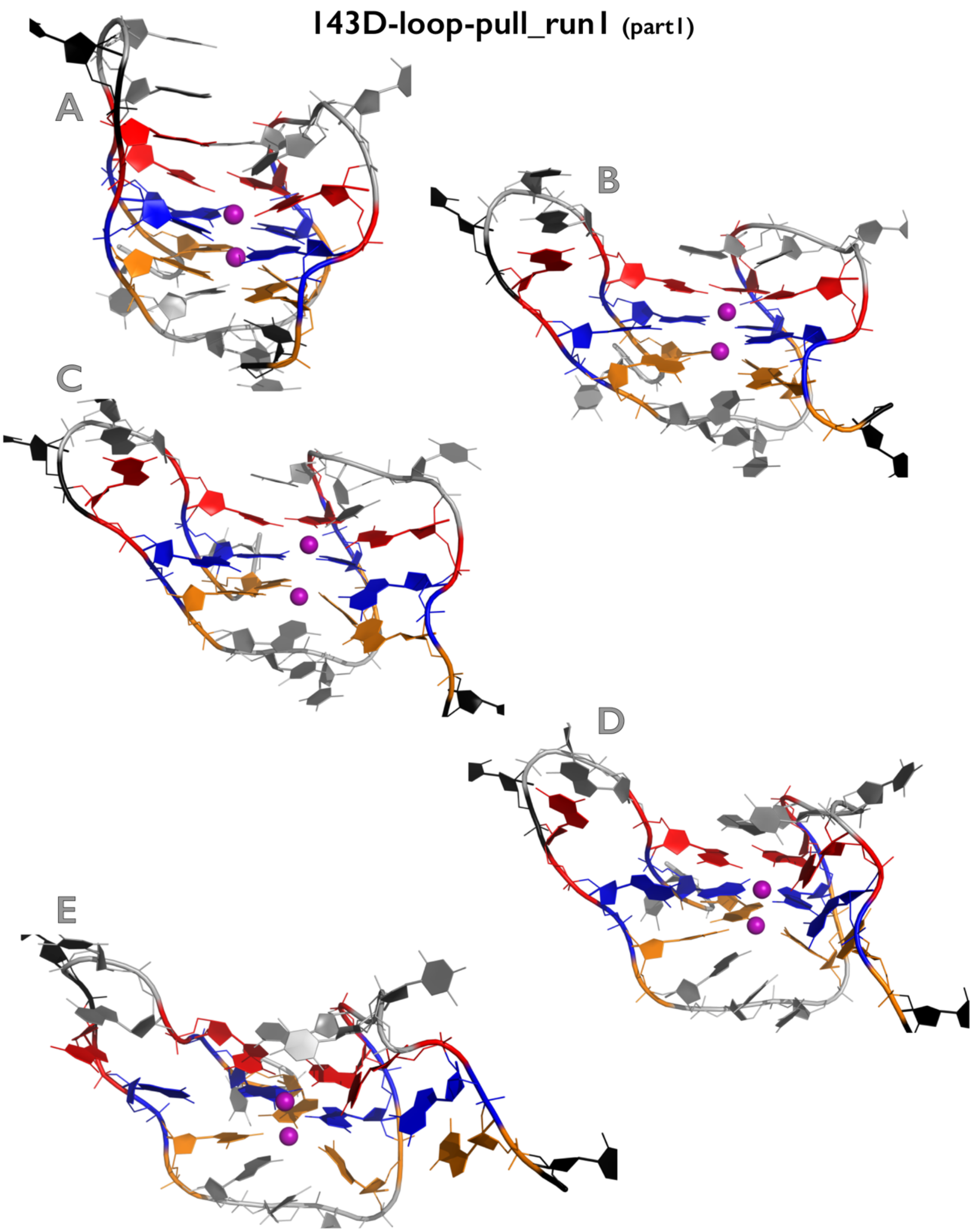

Figure continuing on the next page

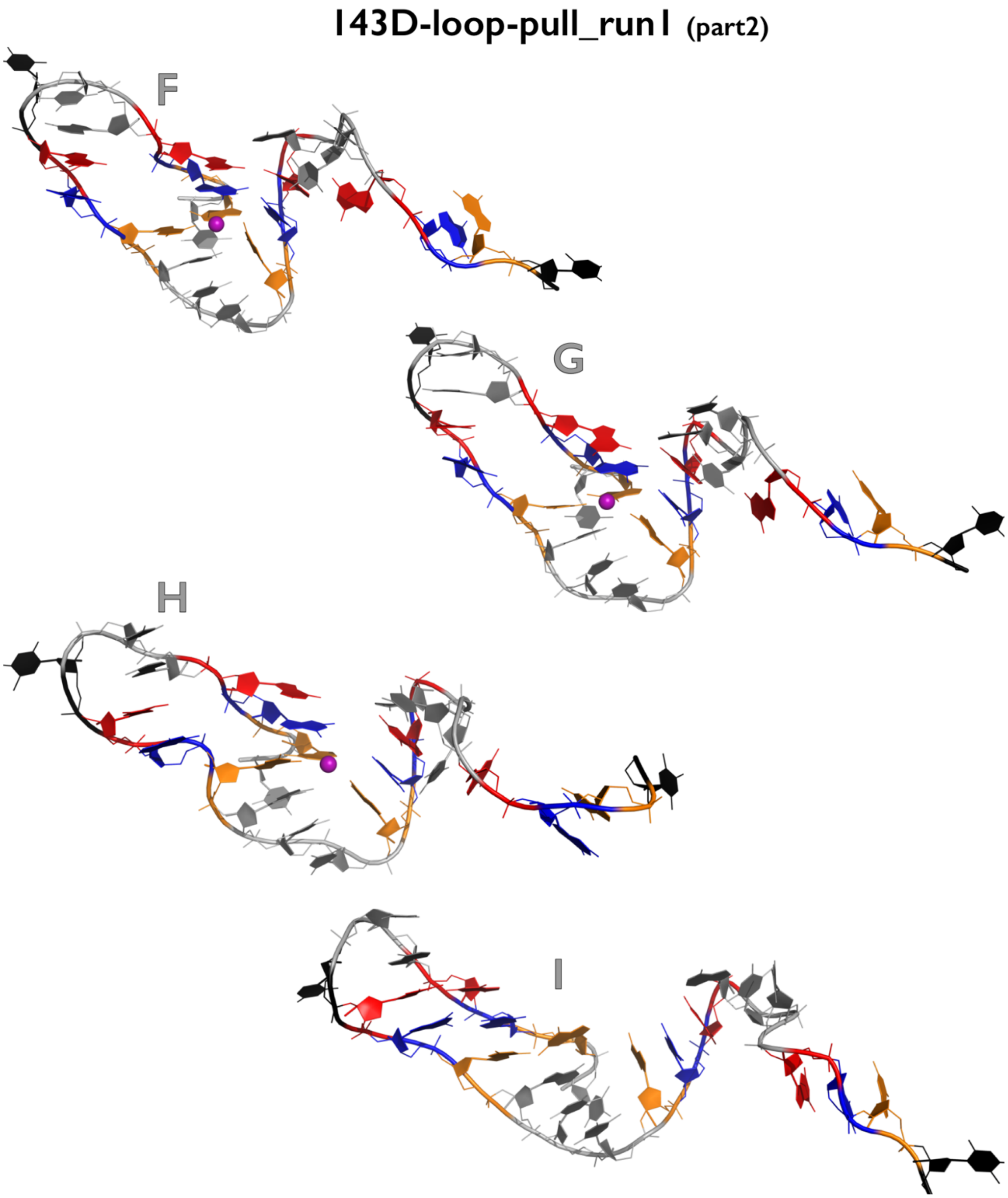

Figure S15B: Most important structural events during first independent *slow zig-zag pulling* simulation of $143D_{loop\text{-}pull}$ GQ system. See legend of Figure S1B for more details.

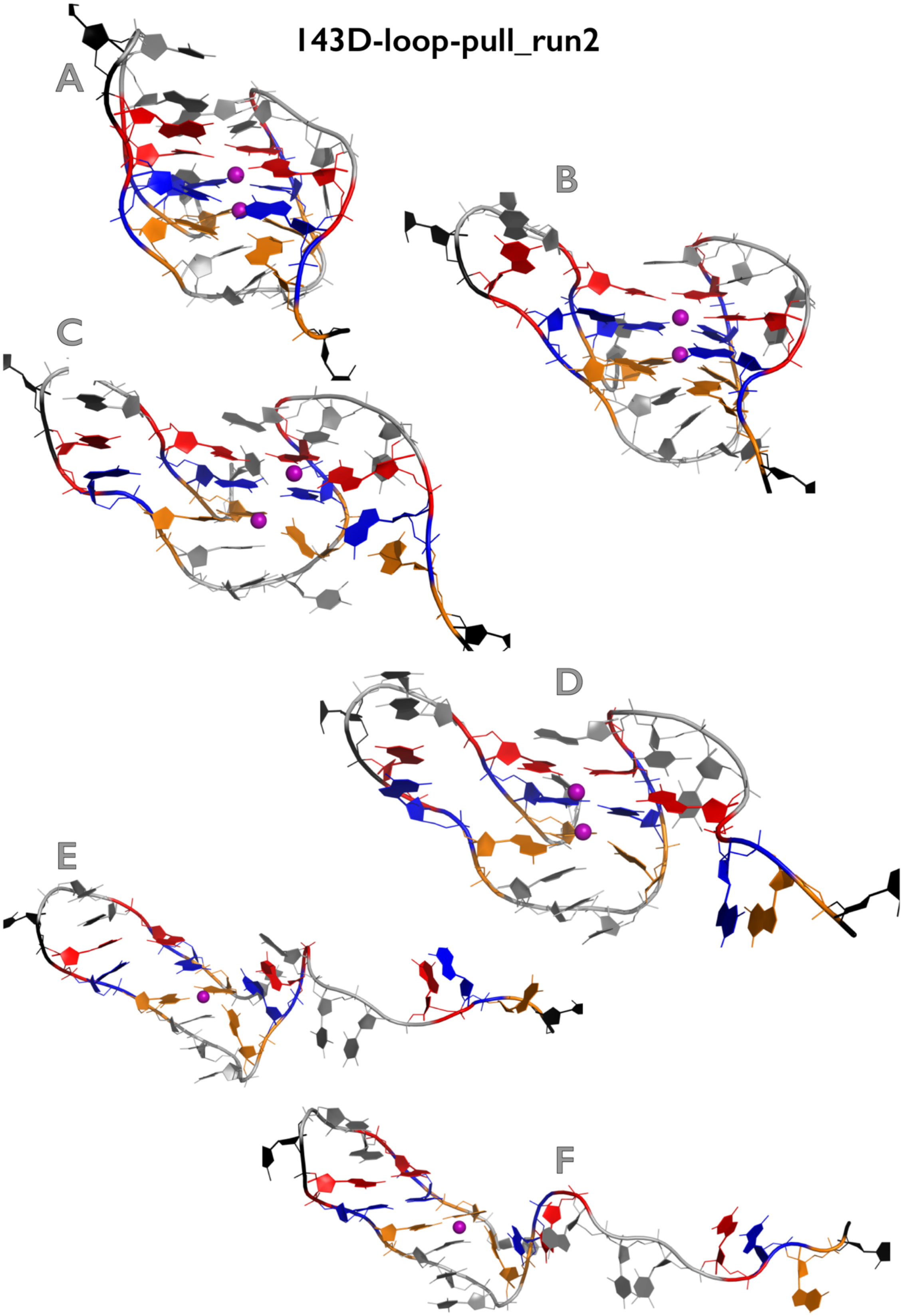

Figure S15C: Most important structural events during second independent *slow zig-zag pulling* simulation of 143D$_{loop\text{-}pull}$ GQ system. See legend of Figure S1B for more details.

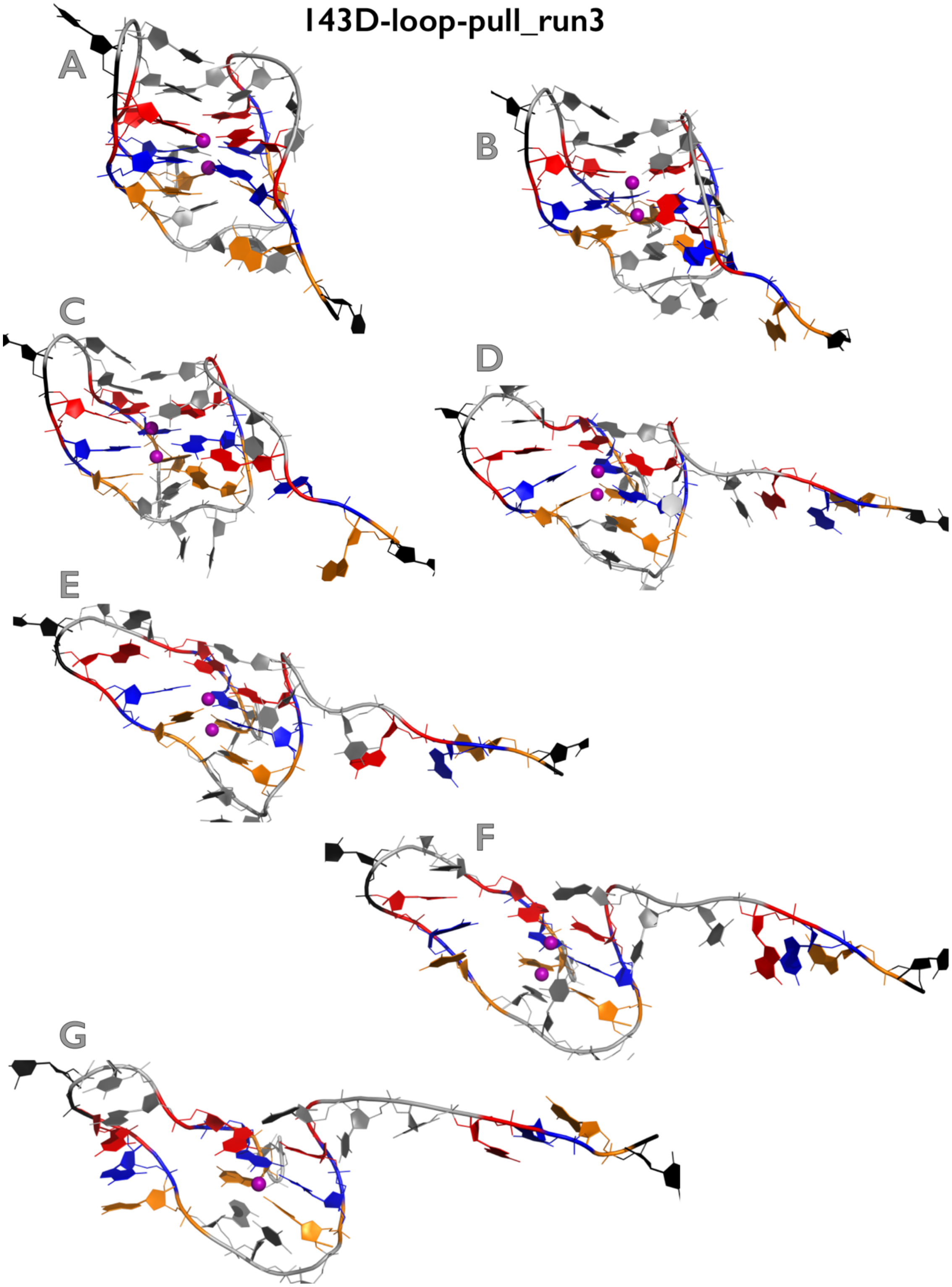

Figure S15D: Most important structural events during third independent *slow zig-zag pulling* simulation of $143D_{loop\text{-}pull}$ GQ system. See legend of Figure S1B for more details.

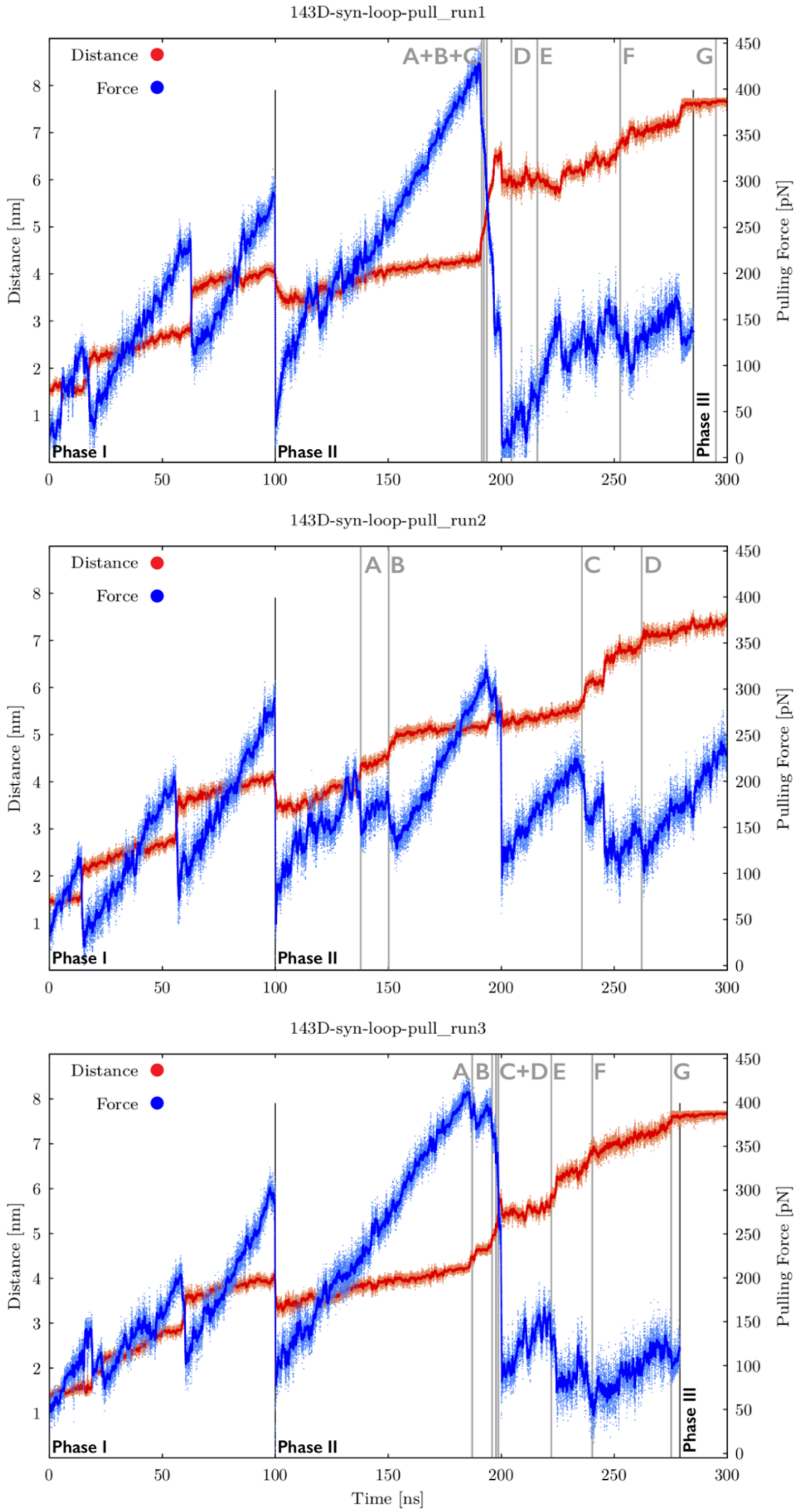

Figure S16A: Time evolution of distance between pulling centers and pulling force during three independent *slow zig-zag pulling* simulations of 143D$_{syn_loop\text{-}pull}$ GQ system (see legend of Figure

S9A for more details). See Figures S16B-S16D for inspection of structures corresponding to main structural events.

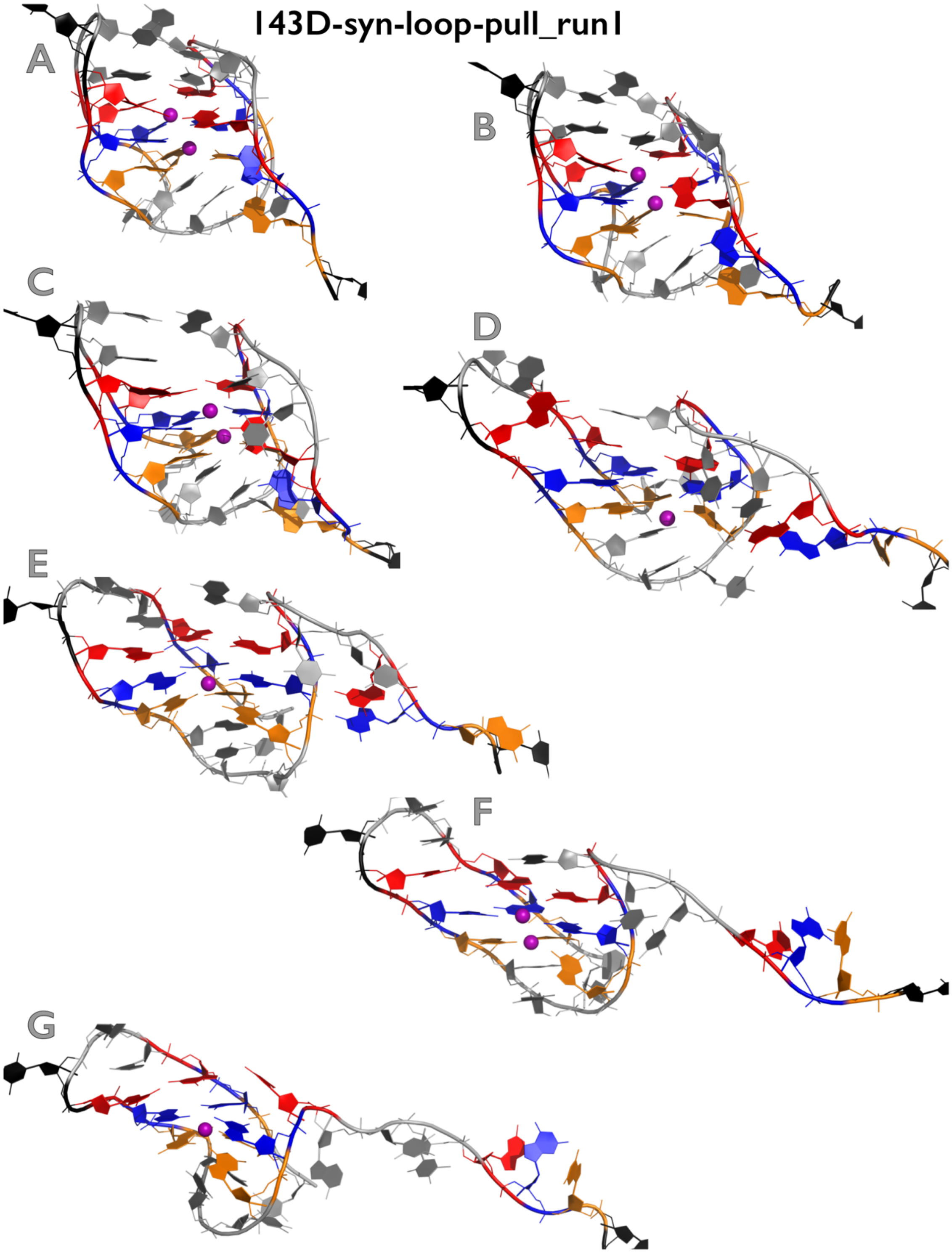

Figure S16B: Most important structural events during first independent *slow zig-zag pulling* simulation of $143D_{syn\_loop\text{-}pull}$ GQ system. See legend of Figure S1B for more details.

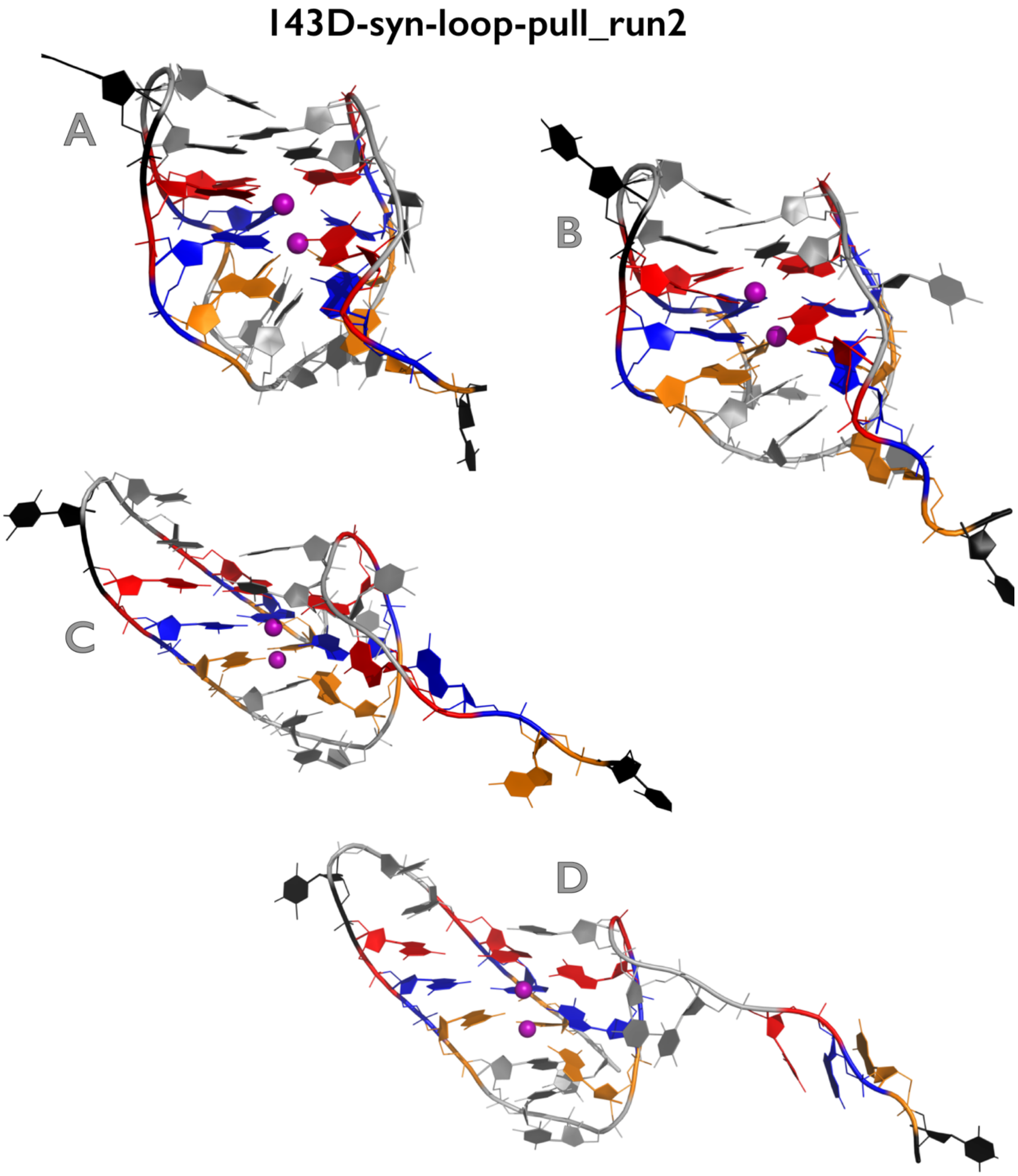

Figure S16C: Most important structural events during second independent *slow zig-zag pulling* simulation of 143D$_{syn_loop\text{-}pull}$ GQ system. See legend of Figure S1B for more details.

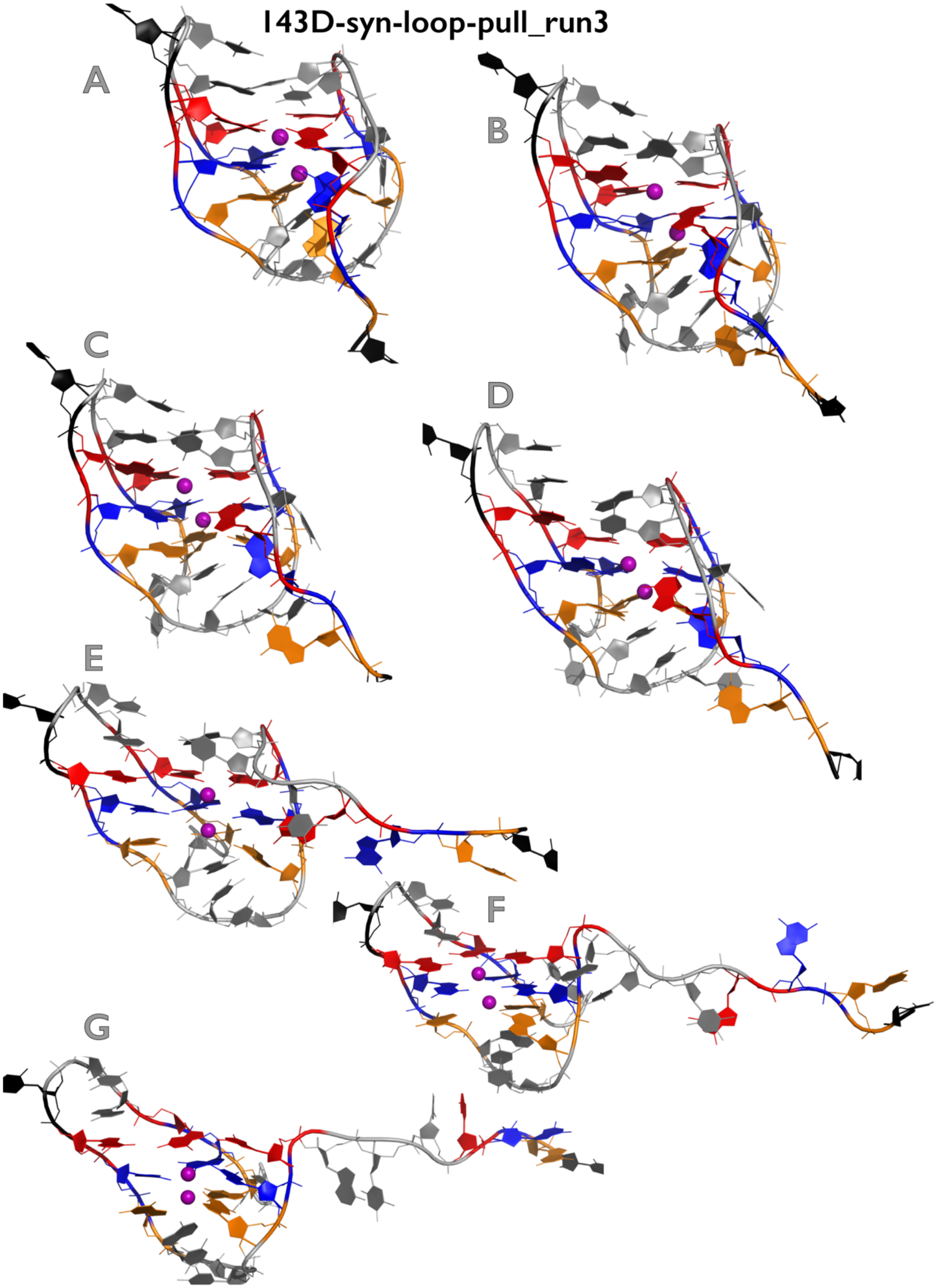

Figure S16D: Most important structural events during third independent *slow zig-zag pulling* simulation of $143D_{syn\_loop\text{-}pull}$ GQ system. See legend of Figure S1B for more details.

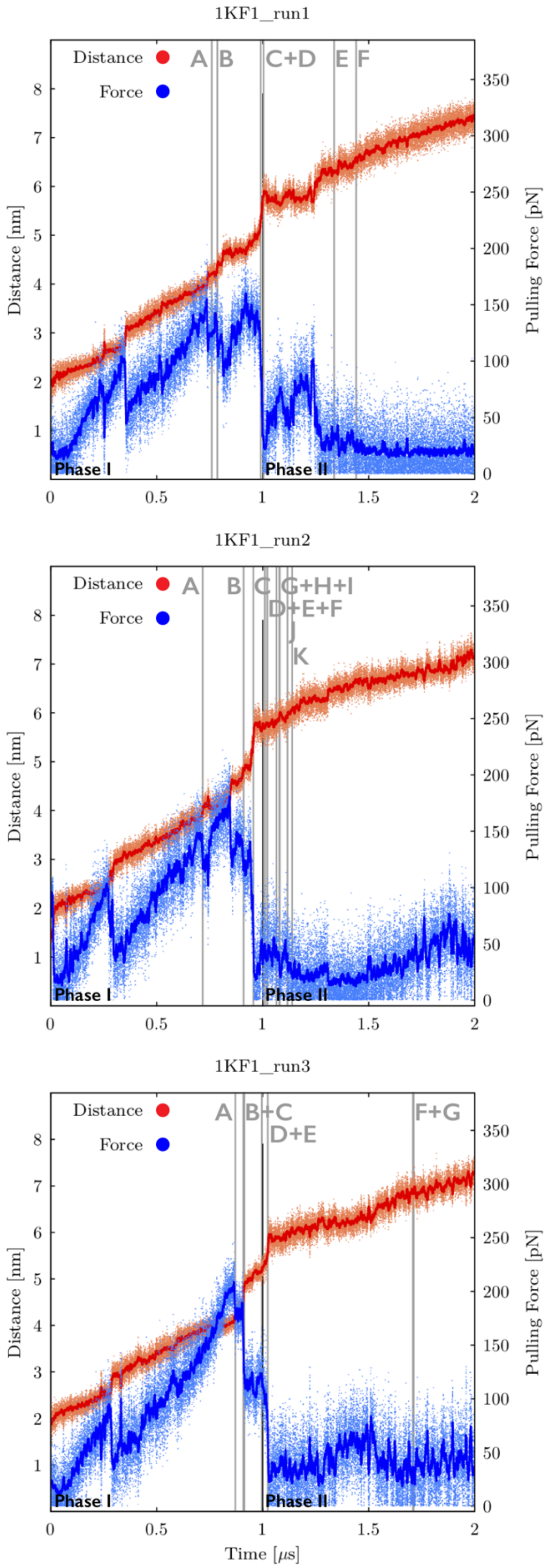

Figure S17A: Time evolution of distance between pulling centers and pulling force during three independent *very slow zig-zag pulling* simulations of 1KF1 GQ system. Snapshots were saved every 50 ps and plots are showing both instantaneous values (orange and light-blue dots for

distance and force, respectively) and smoothing, i.e., averaging over 100 consecutive snapshots (red and blue lines for distance and force, respectively). Pulling phases are marked (see Methods in the main text for details) and main structural events are highlighted as grey vertical lines with labels (capital letters). See Figures S17B-S17D for inspection of structures corresponding to main structural events. Note that first major drops of the pulling force before the GQ unfolding event "A" (and notable prolongation of end-to-end distances) are connected with repositioning of terminal T residues.

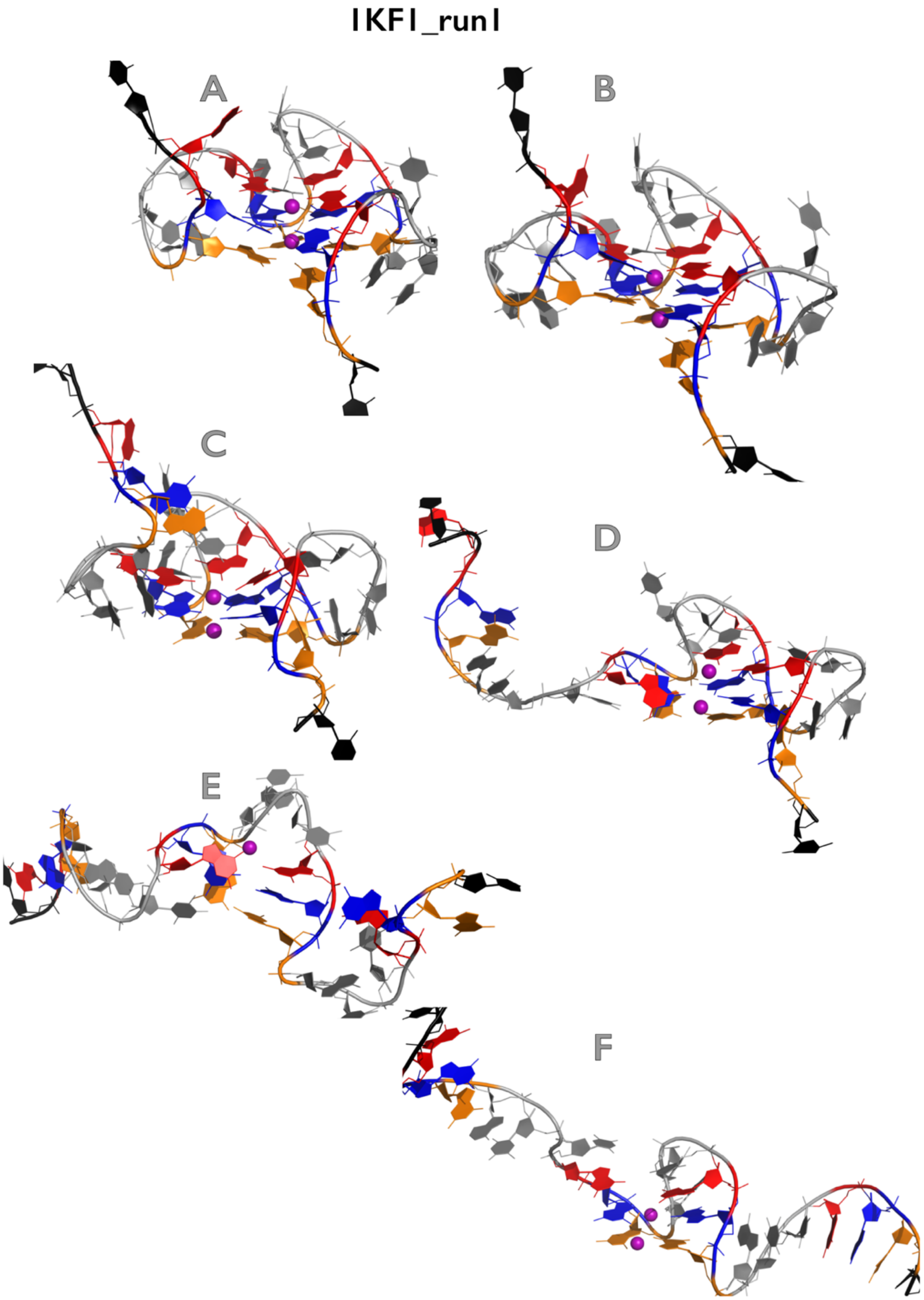

Figure S17B: Most important structural events during first independent *very slow zig-zag pulling* simulation of 1KF1 GQ system. See legend of Figure S1B for more details.

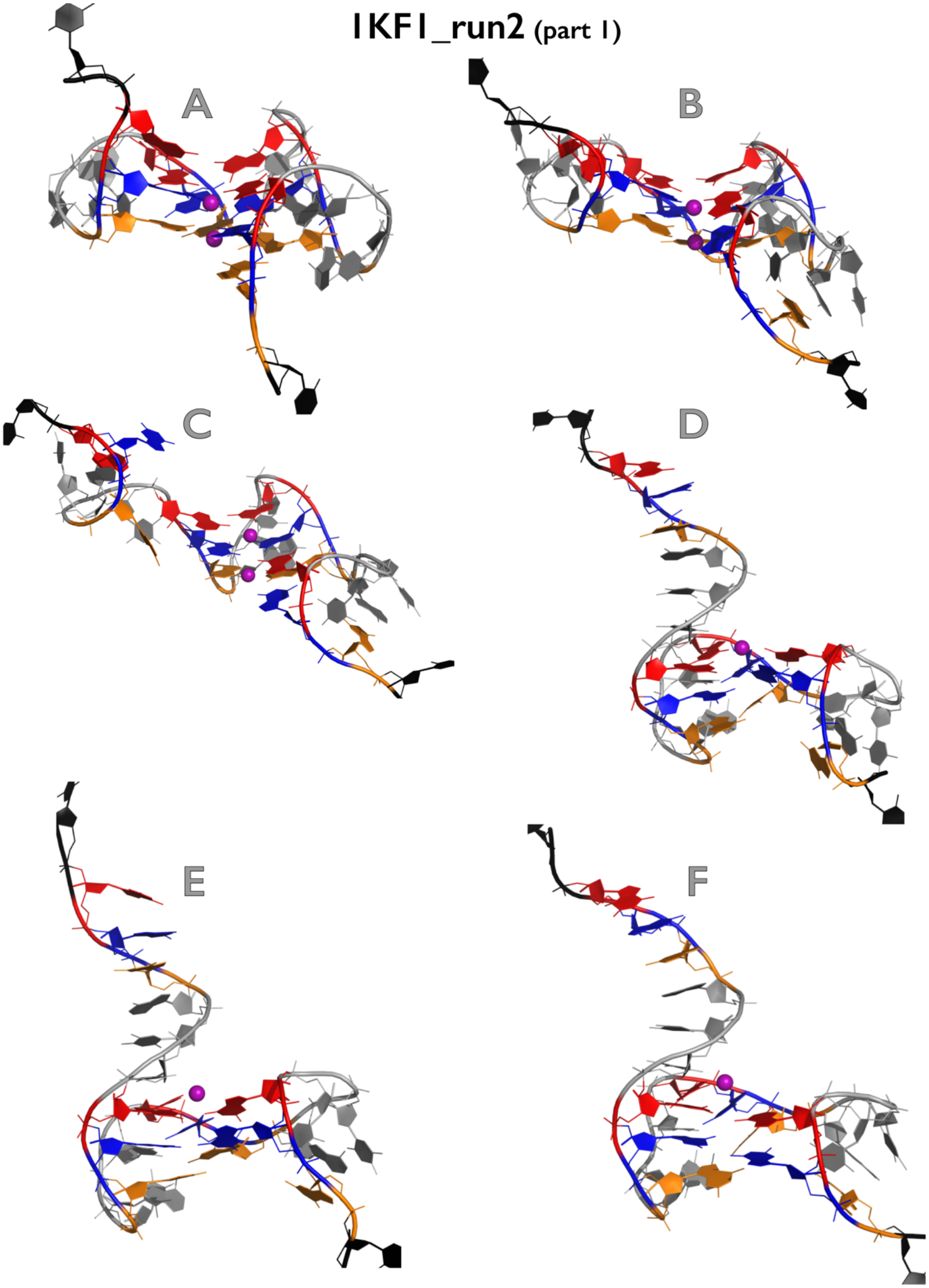

Figure continuing on the next page

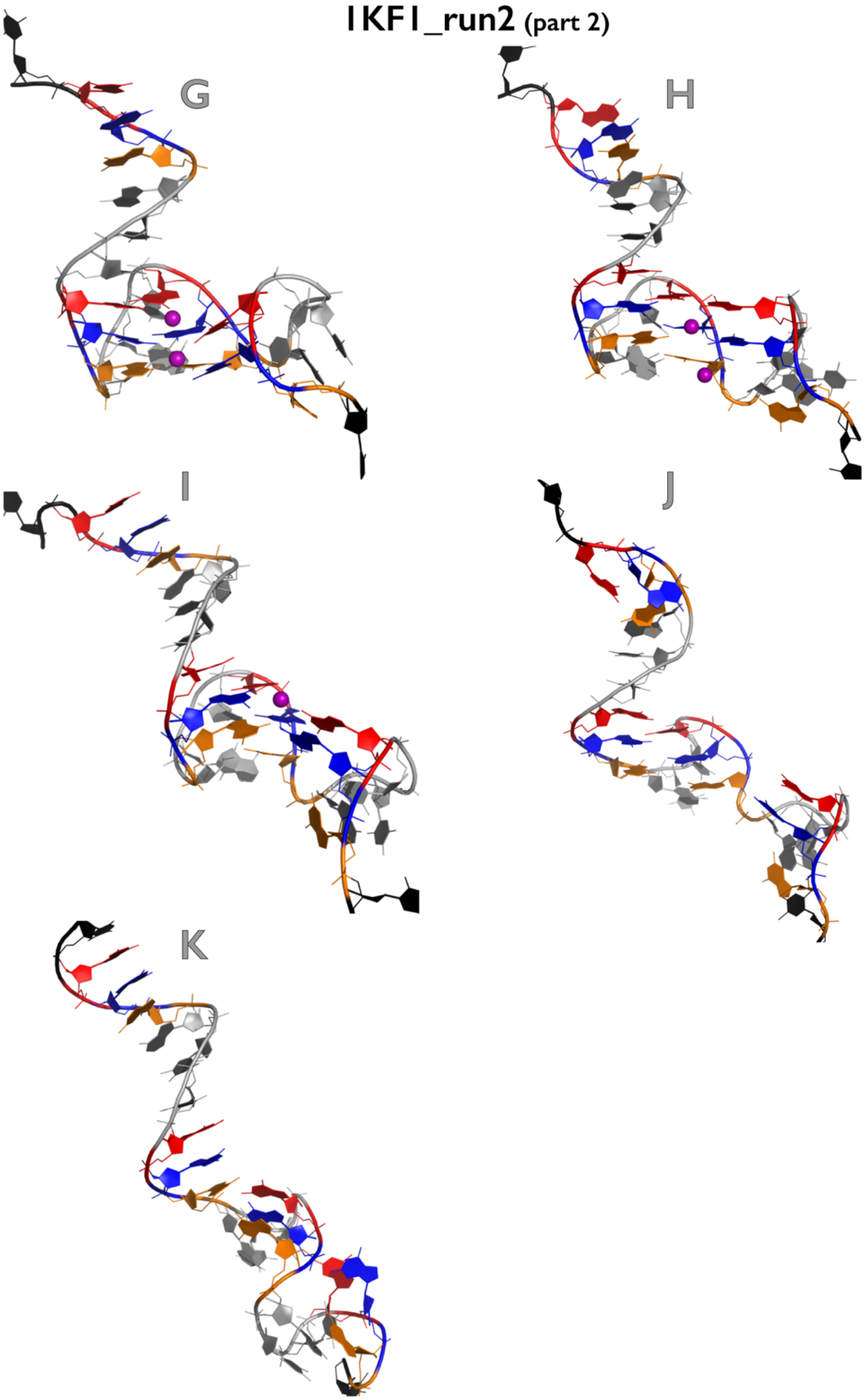

Figure S17C: Most important structural events during second independent *very slow zig-zag pulling* simulation of 1KF1 GQ system. See legend of Figure S1B for more details.

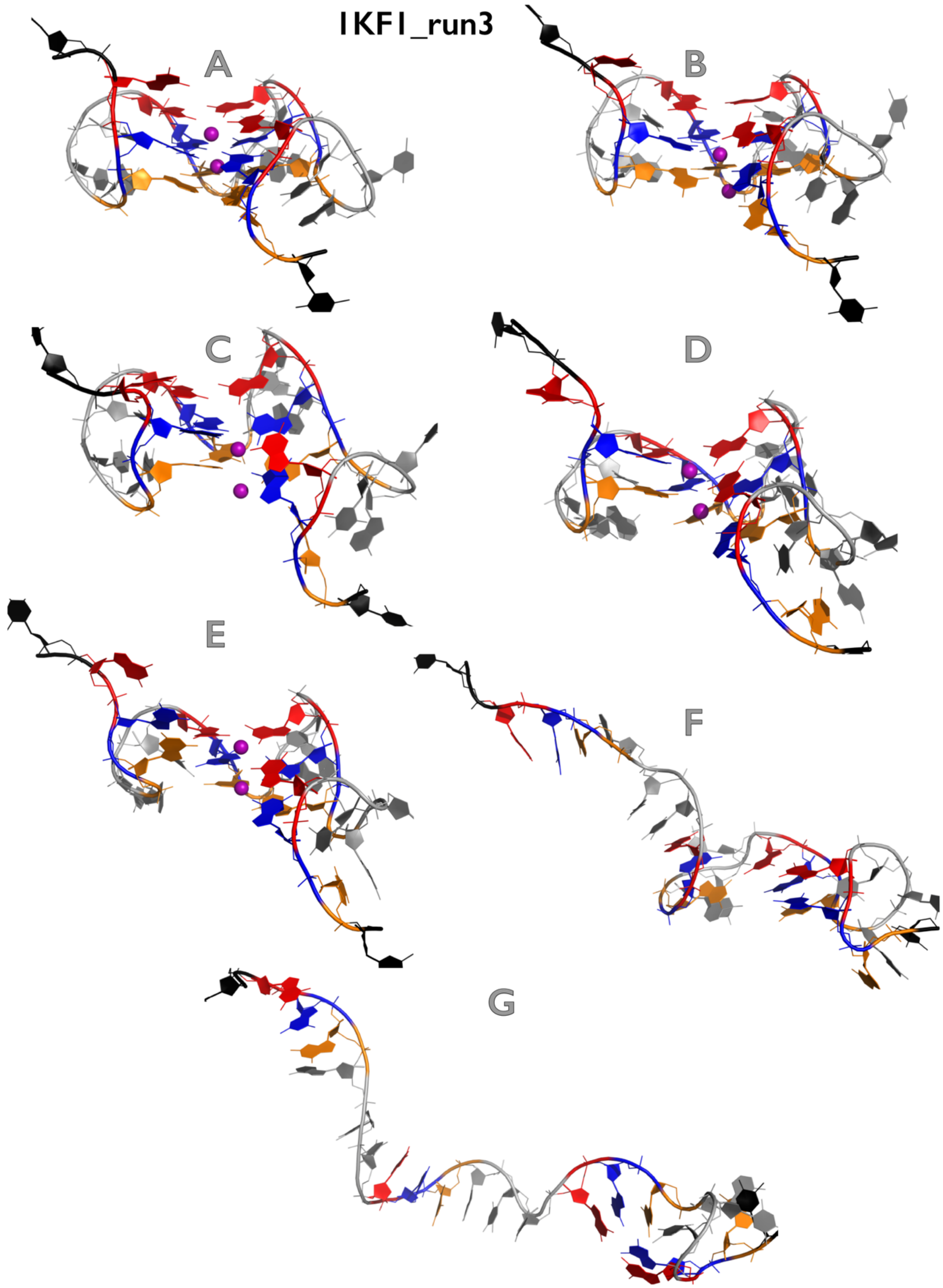

Figure S17D: Most important structural events during third independent *very slow zig-zag pulling* simulation of 1KF1 GQ system. See legend of Figure S1B for more details.

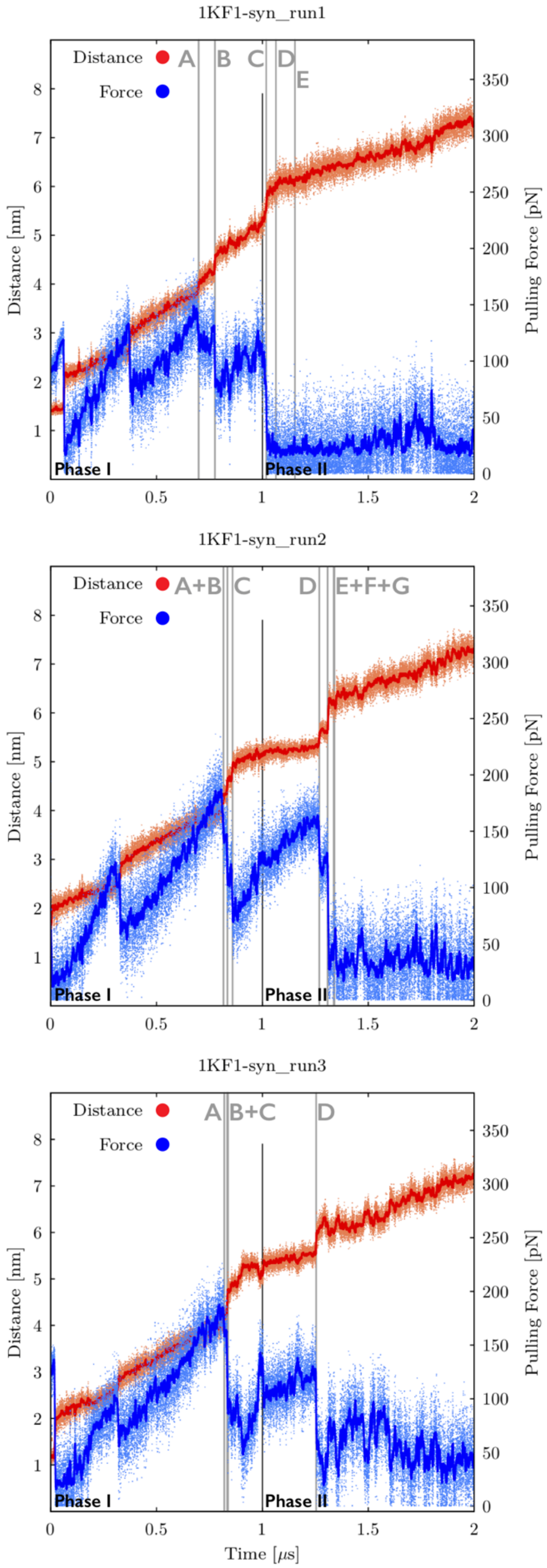

Figure S18A: Time evolution of distance between pulling centers and pulling force during three independent *very slow zig-zag pulling* simulations of 1KF1$_{syn}$ GQ system (see legend of Figure

S17A for more details). See Figures S18B-S18D for inspection of structures corresponding to main structural events.

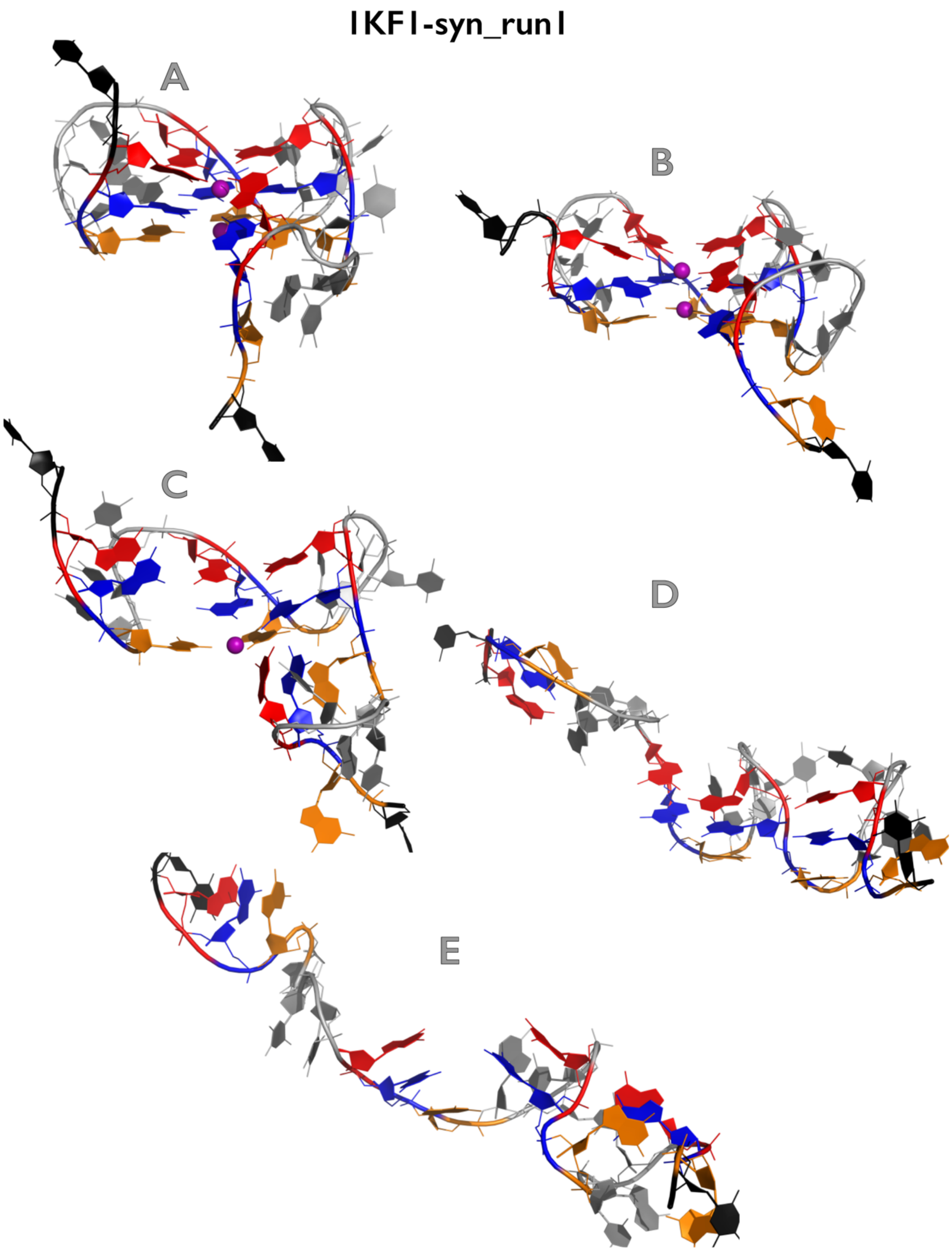

Figure S18B: Most important structural events during first independent *very slow zig-zag pulling* simulation of $1KF1_{syn}$ GQ system. See legend of Figure S1B for more details.

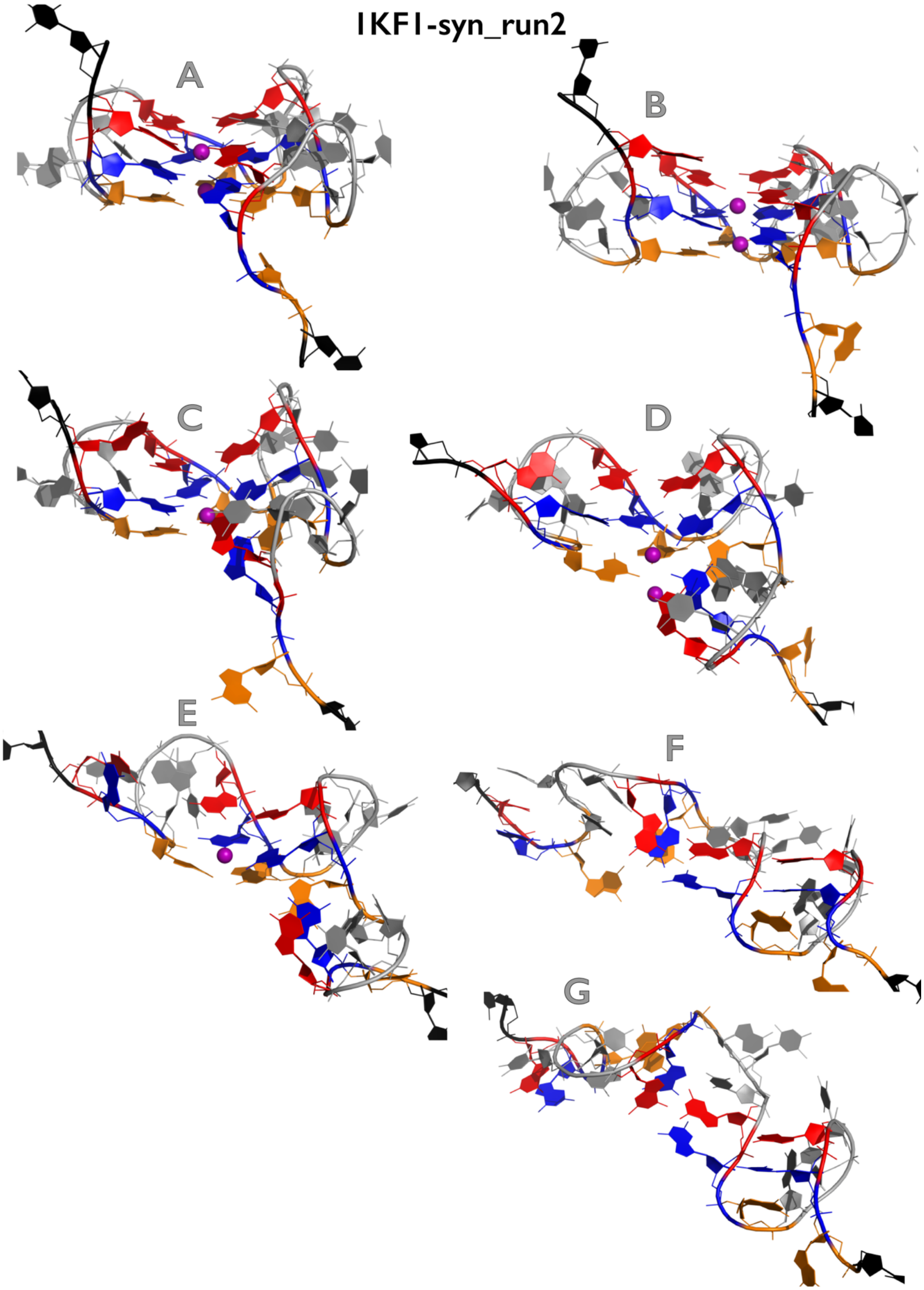

Figure S18C: Most important structural events during second independent *very slow zig-zag pulling* simulation of $1KF1_{syn}$ GQ system. See legend of Figure S1B for more details.

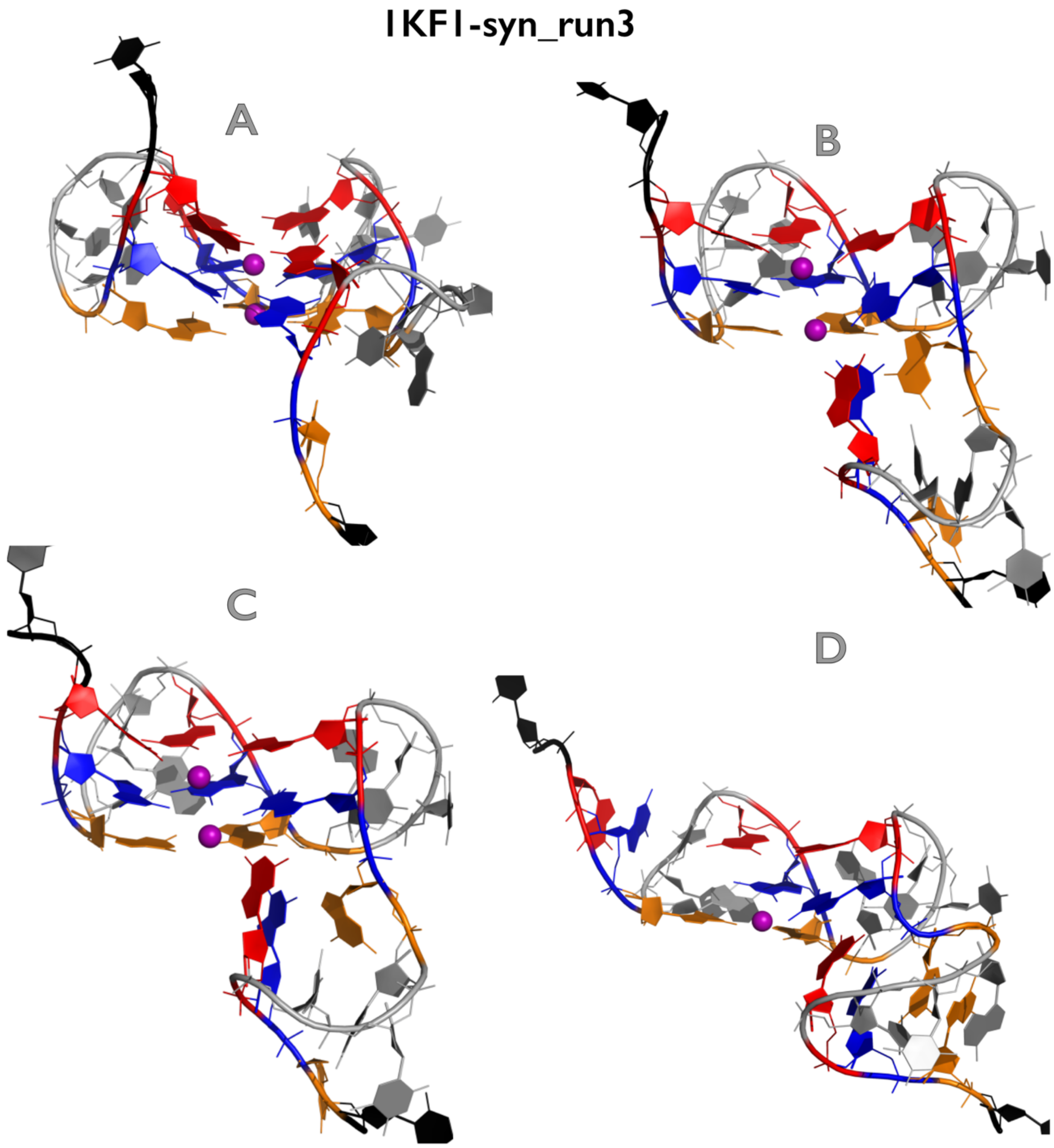

Figure S18D: Most important structural events during third independent *very slow zig-zag pulling* simulation of $1KF1_{syn}$ GQ system. See legend of Figure S1B for more details.

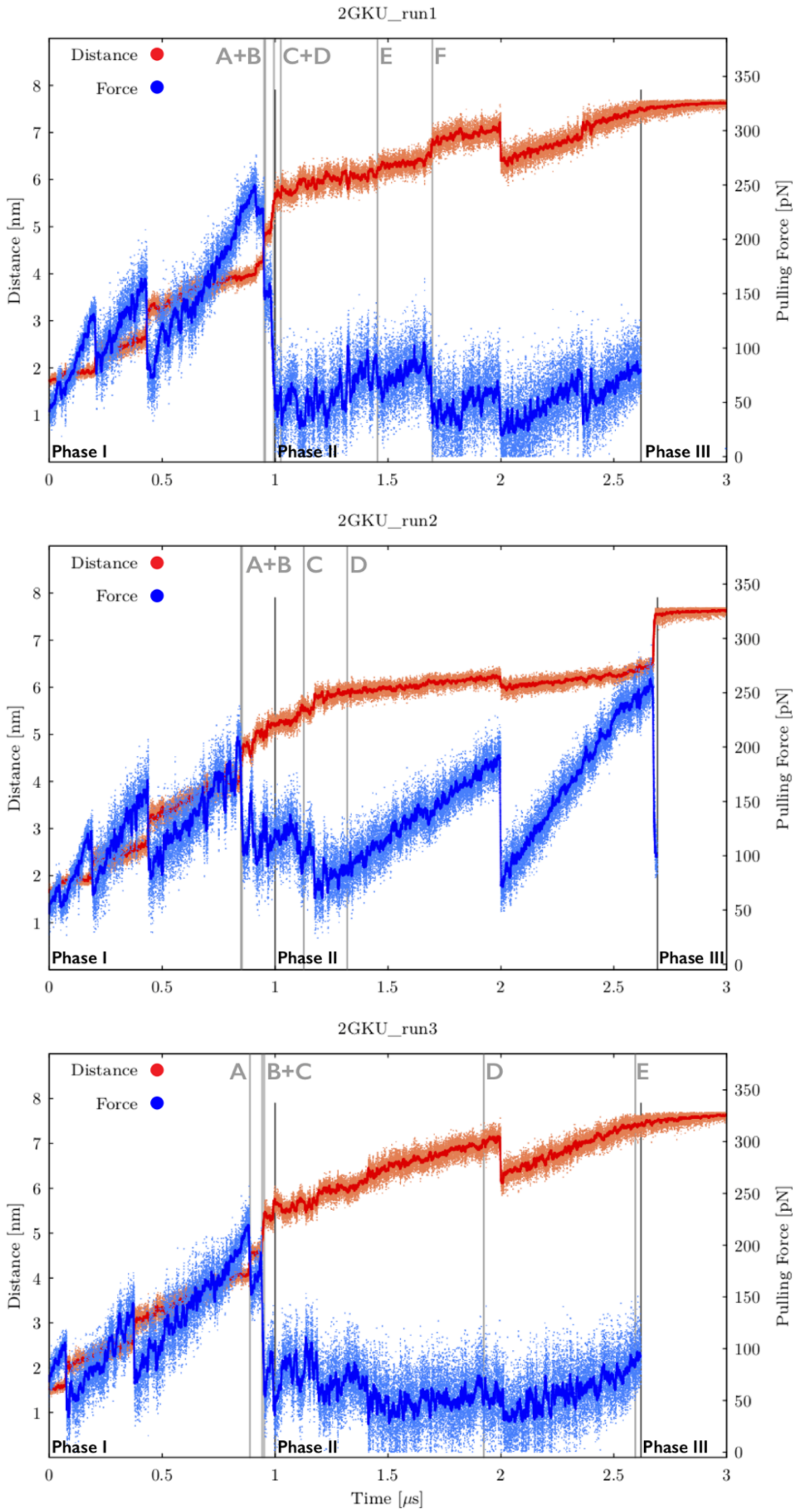

Figure S19A: Time evolution of distance between pulling centers and pulling force during three independent *very slow zig-zag pulling* simulations of 2GKU GQ system (see legend of Figure

S17A for more details). See Figures S19B-S19D for inspection of structures corresponding to main structural events.

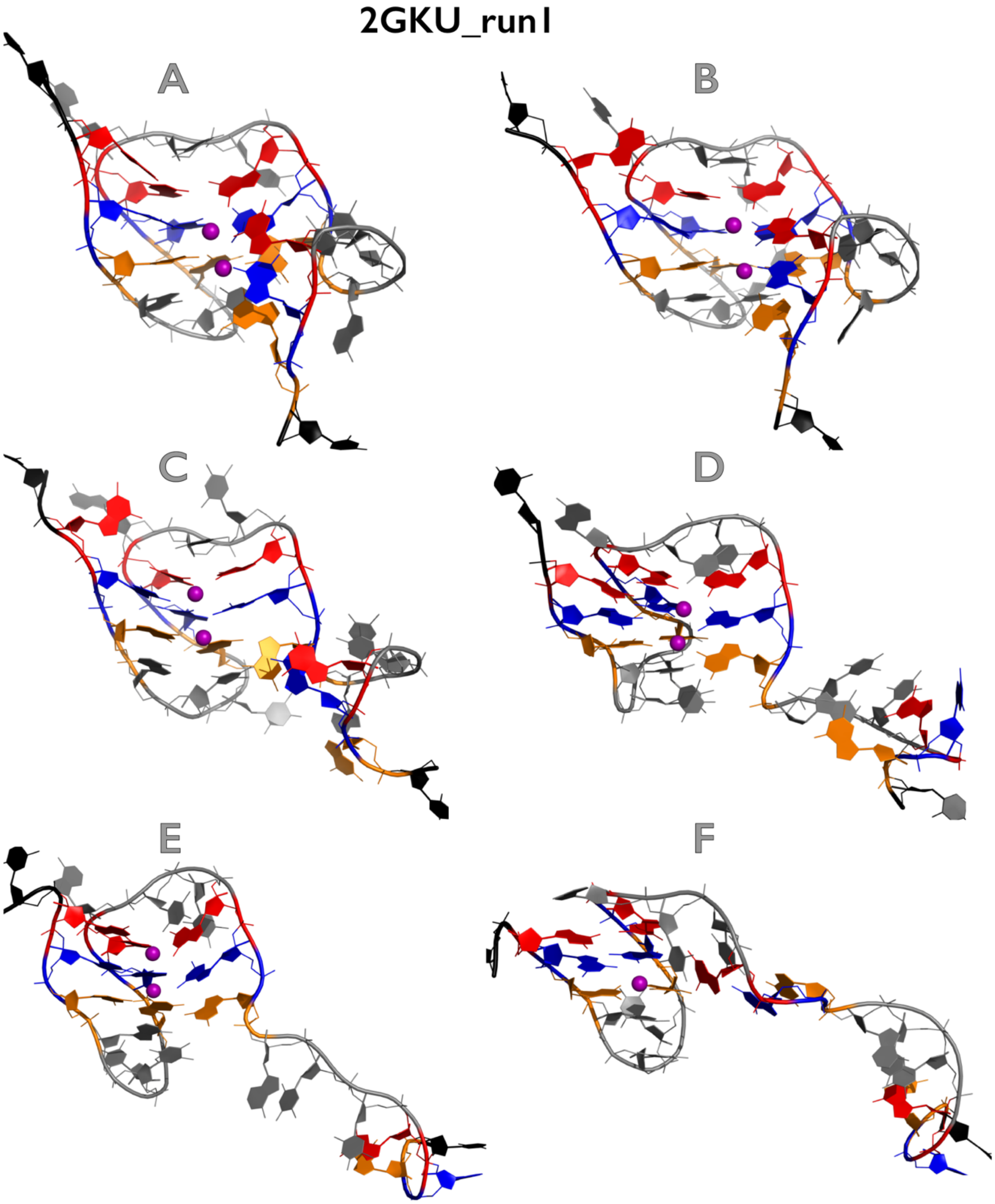

Figure S19B: Most important structural events during first independent *very slow zig-zag pulling* simulation of 2GKU GQ system. See legend of Figure S1B for more details.

2GKU_run2

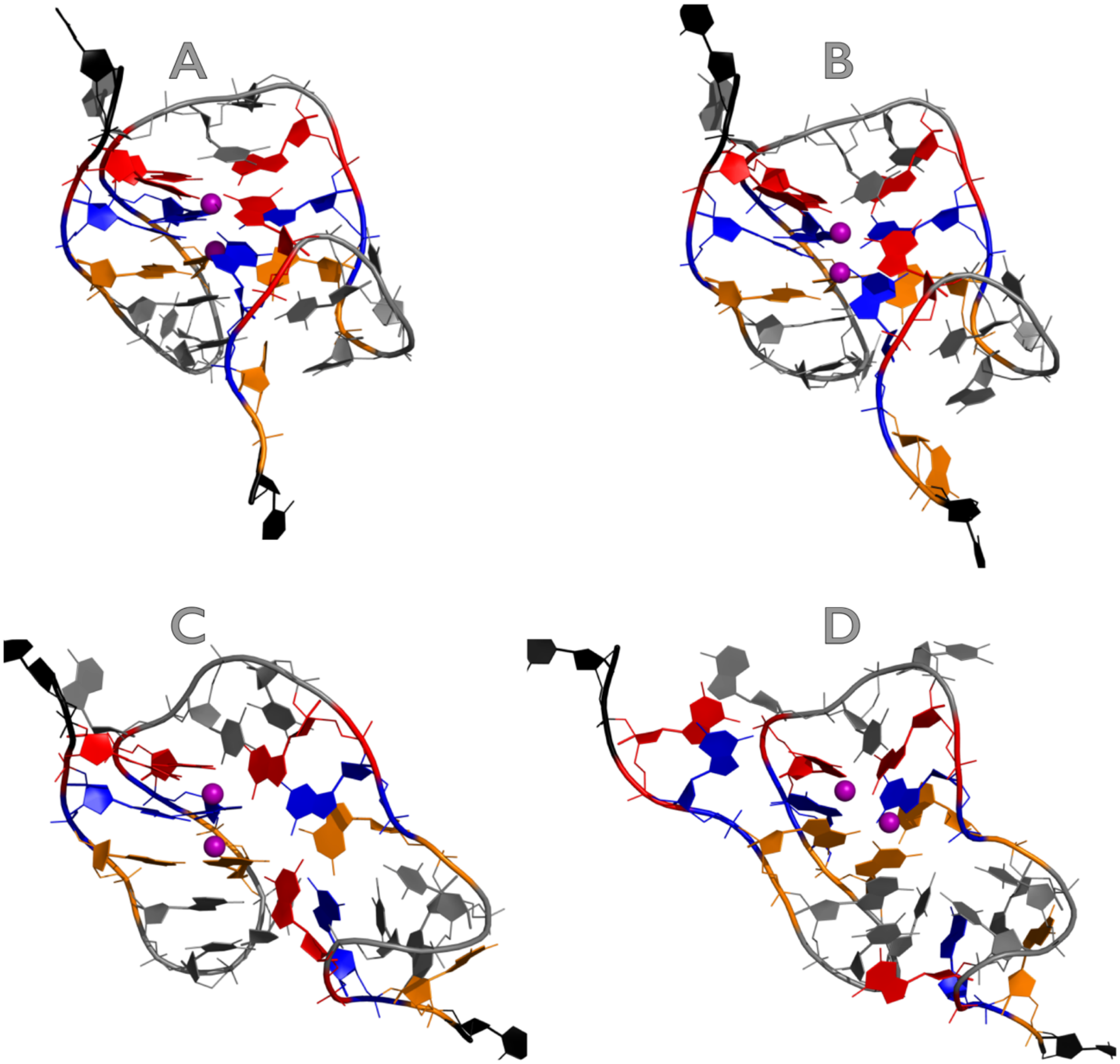

Figure S19C: Most important structural events during second independent *very slow zig-zag pulling* simulation of 2GKU GQ system. See legend of Figure S1B for more details.

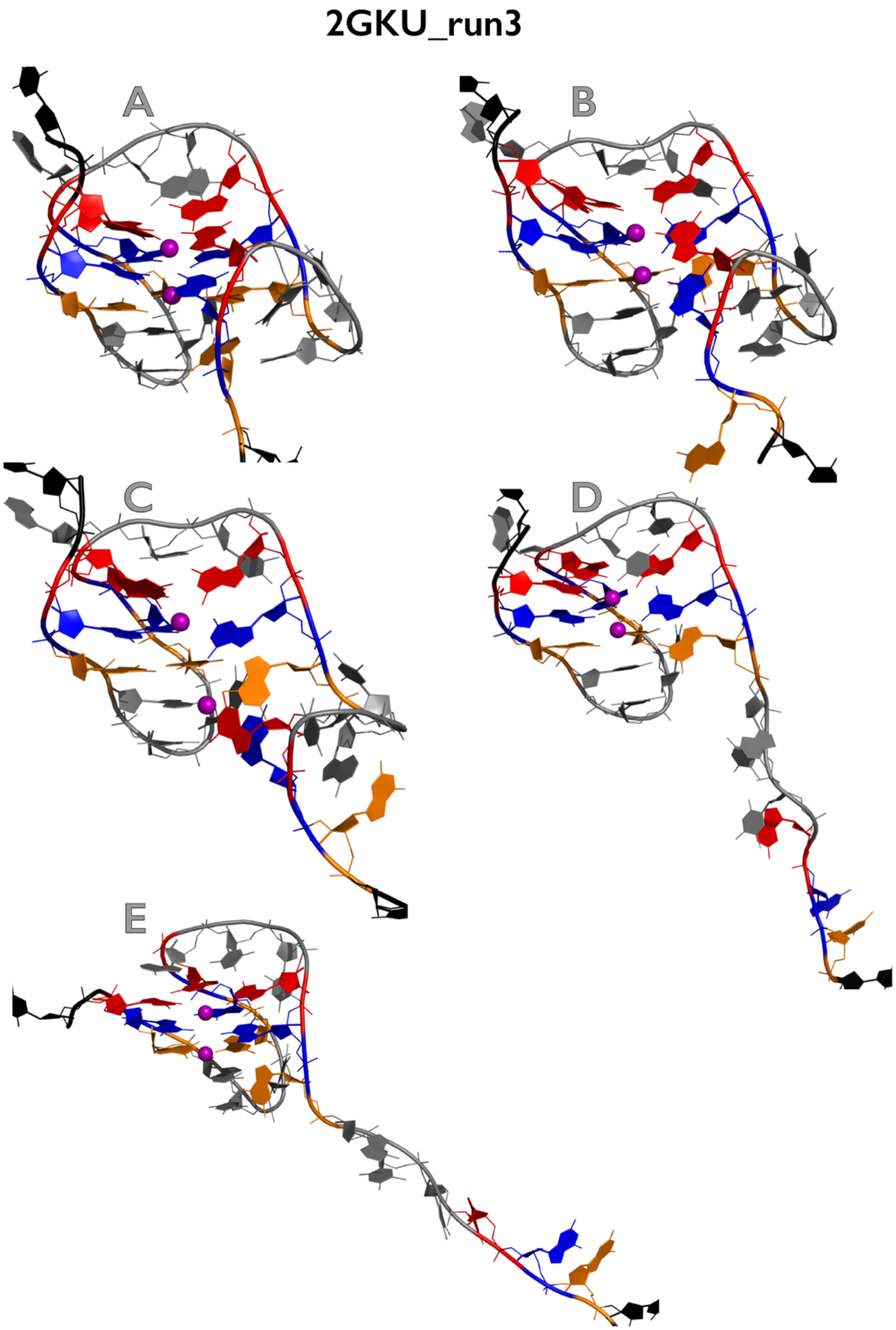

Figure S19D: Most important structural events during third independent *very slow zig-zag pulling* simulation of 2GKU GQ system. See legend of Figure S1B for more details.

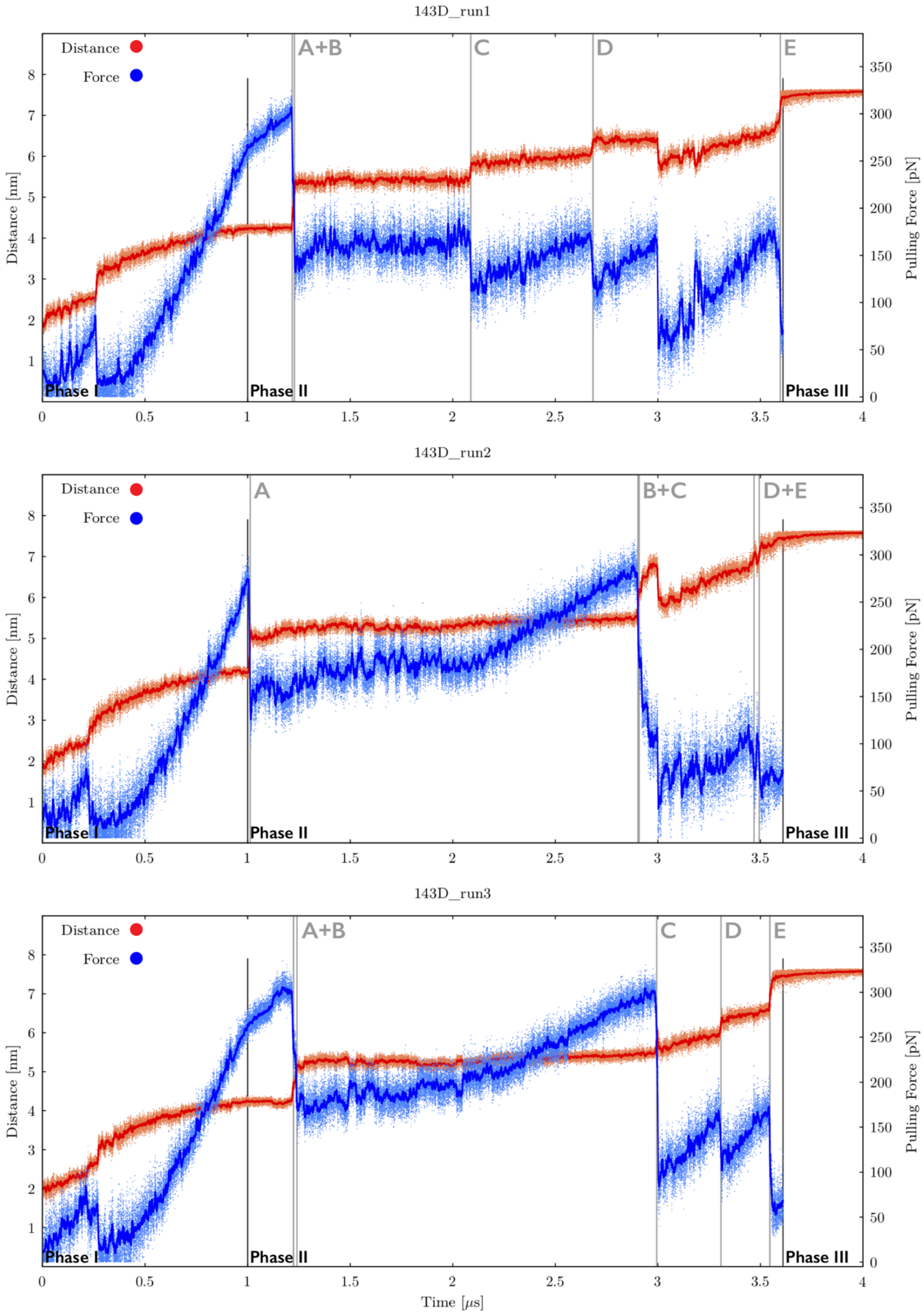

Figure S20A: Time evolution of distance between pulling centers and pulling force during three independent *very slow zig-zag pulling* simulations of 143D GQ system (see legend of Figure S17A

for more details). See Figures S20B-S20D for inspection of structures corresponding to main structural events.

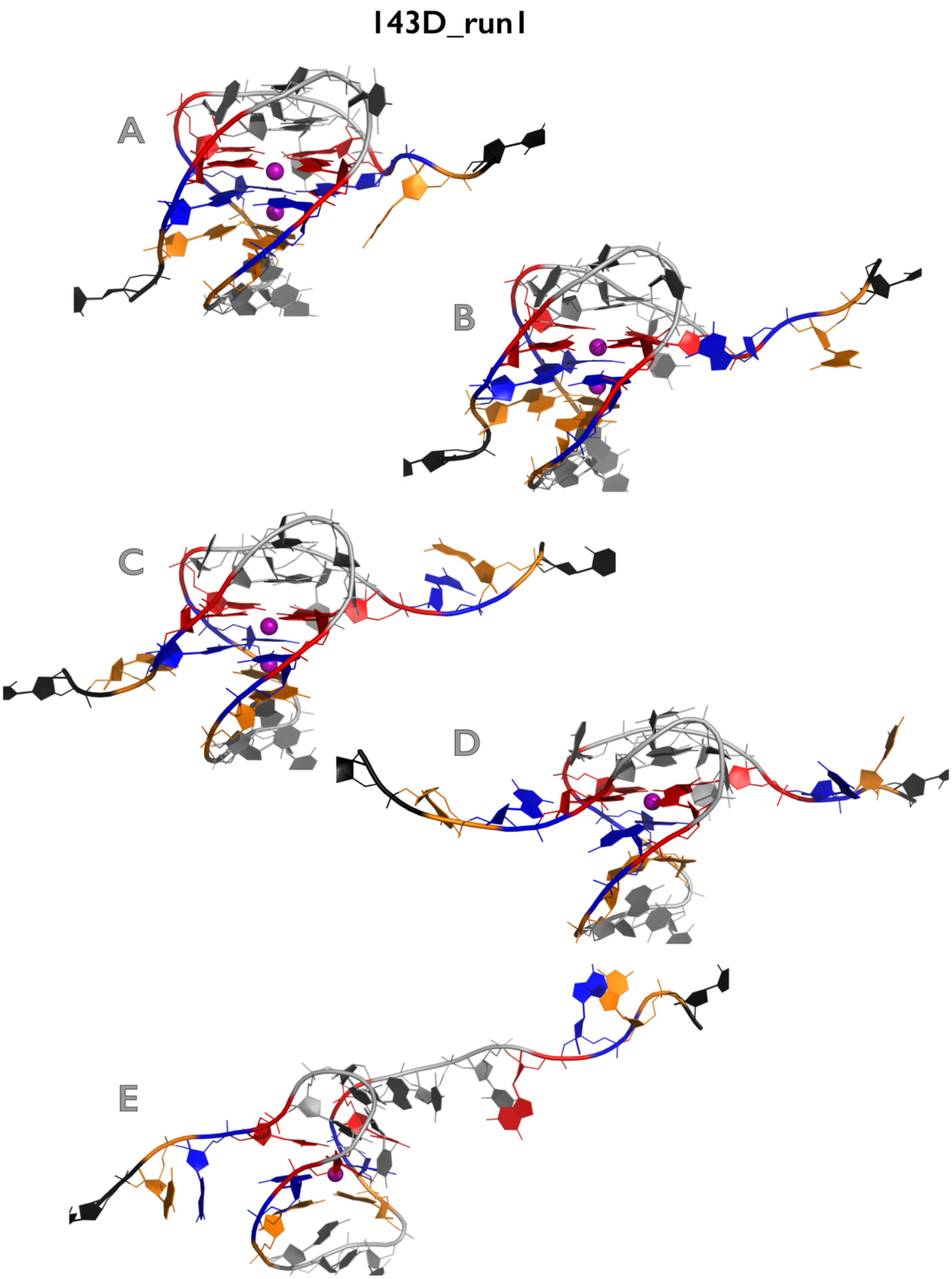

Figure S20B: Most important structural events during first independent *very slow zig-zag pulling* simulation of 143D GQ system. See legend of Figure S1B for more details.

143D_run2

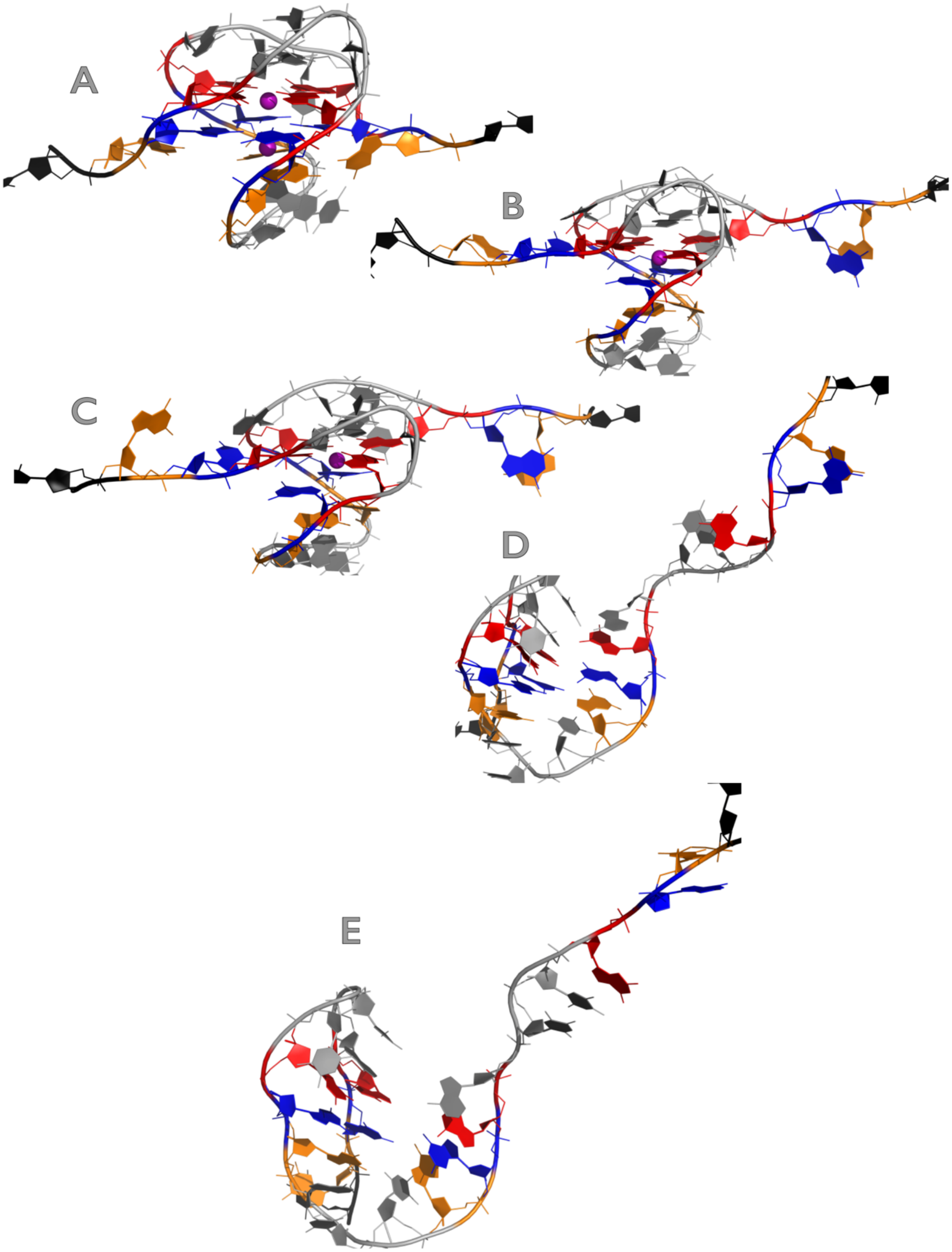

Figure S20C: Most important structural events during second independent *very slow zig-zag pulling* simulation of 143D GQ system. See legend of Figure S1B for more details.

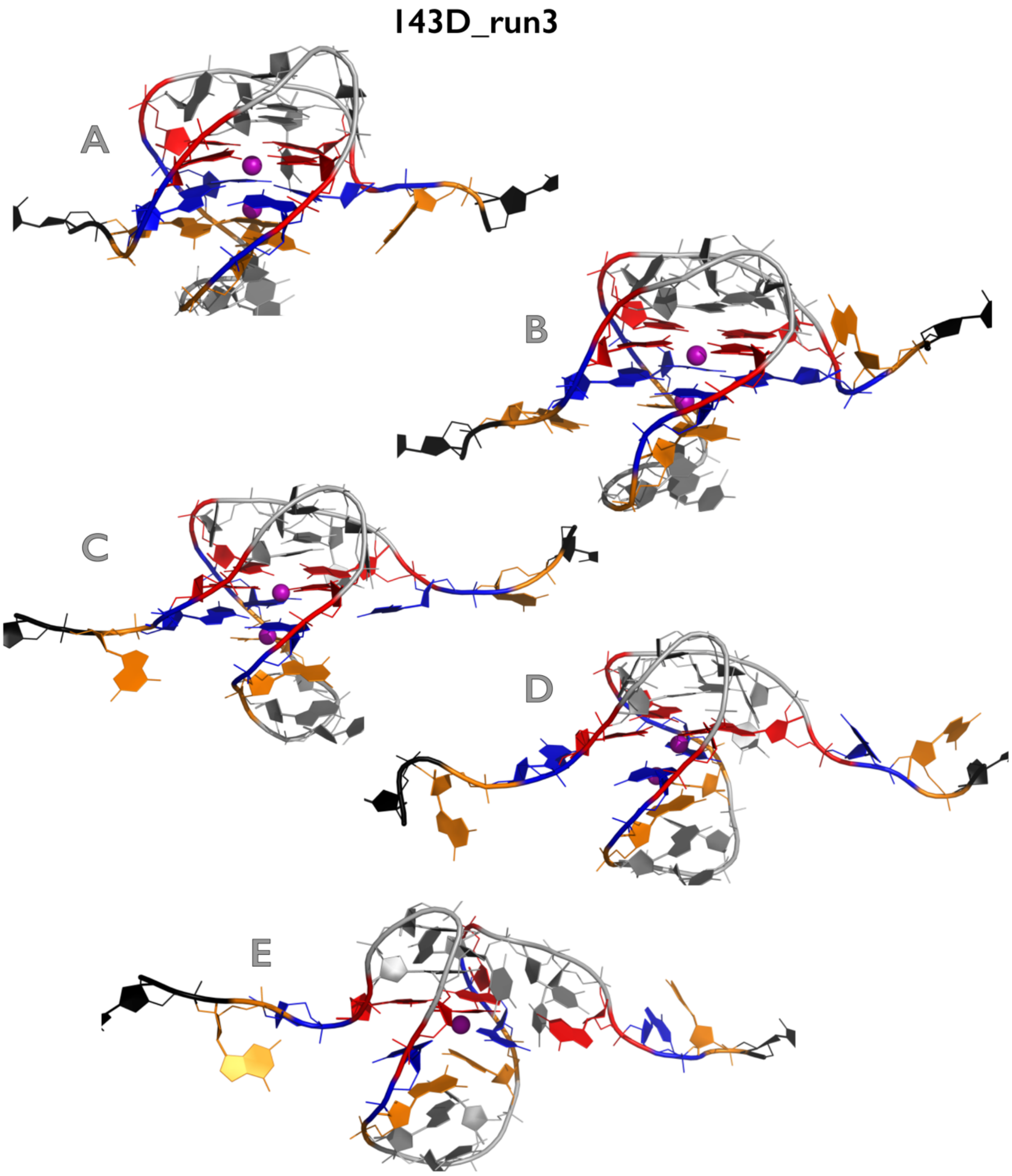

Figure S20D: Most important structural events during third independent *very slow zig-zag pulling* simulation of 143D GQ system. See legend of Figure S1B for more details.

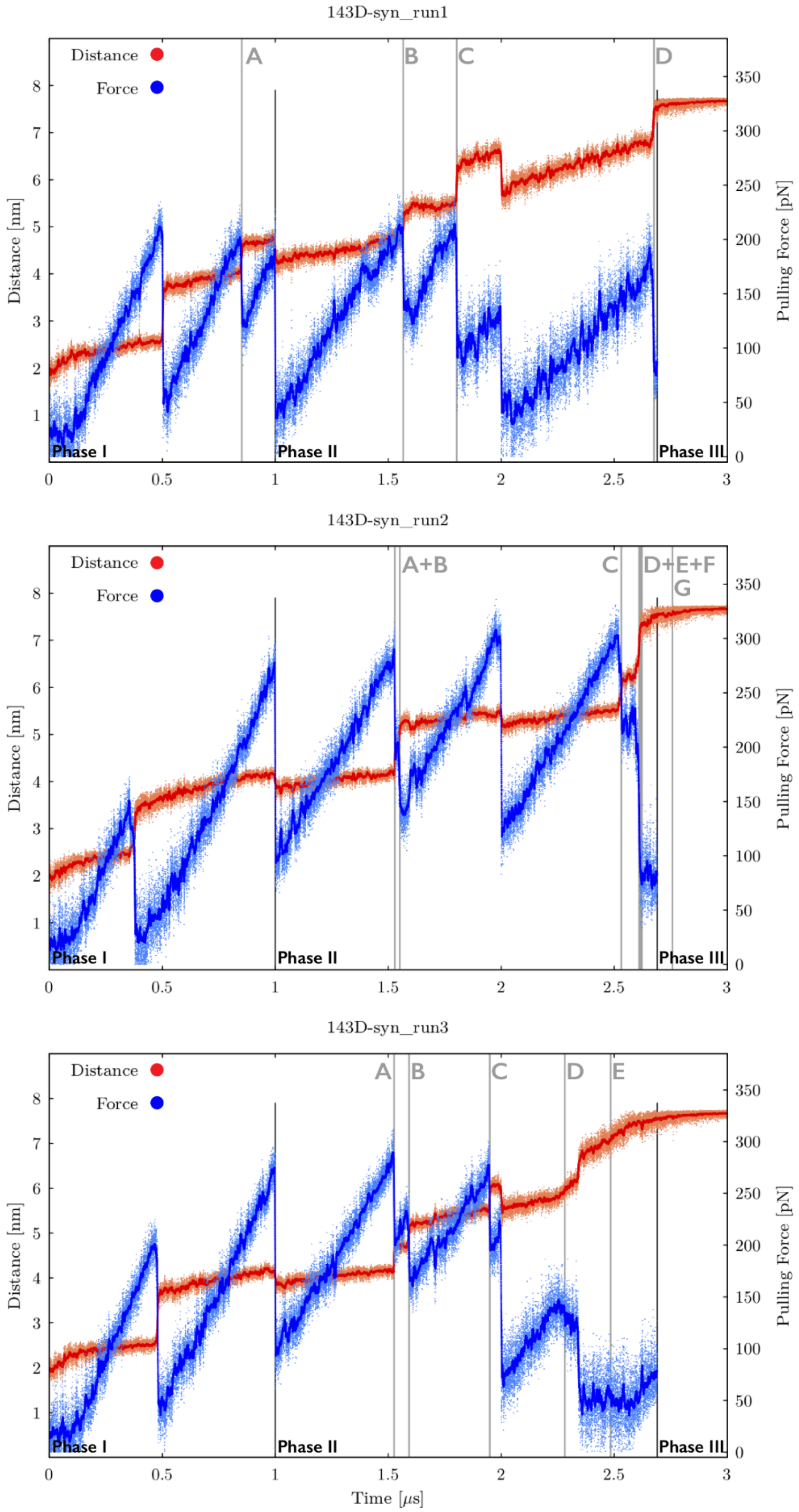

Figure S21A: Time evolution of distance between pulling centers and pulling force during three independent *very slow zig-zag pulling* simulations of 143D$_{syn}$ GQ system (see legend of Figure

S17A for more details). See Figures S21B-S21D for inspection of structures corresponding to main structural events.

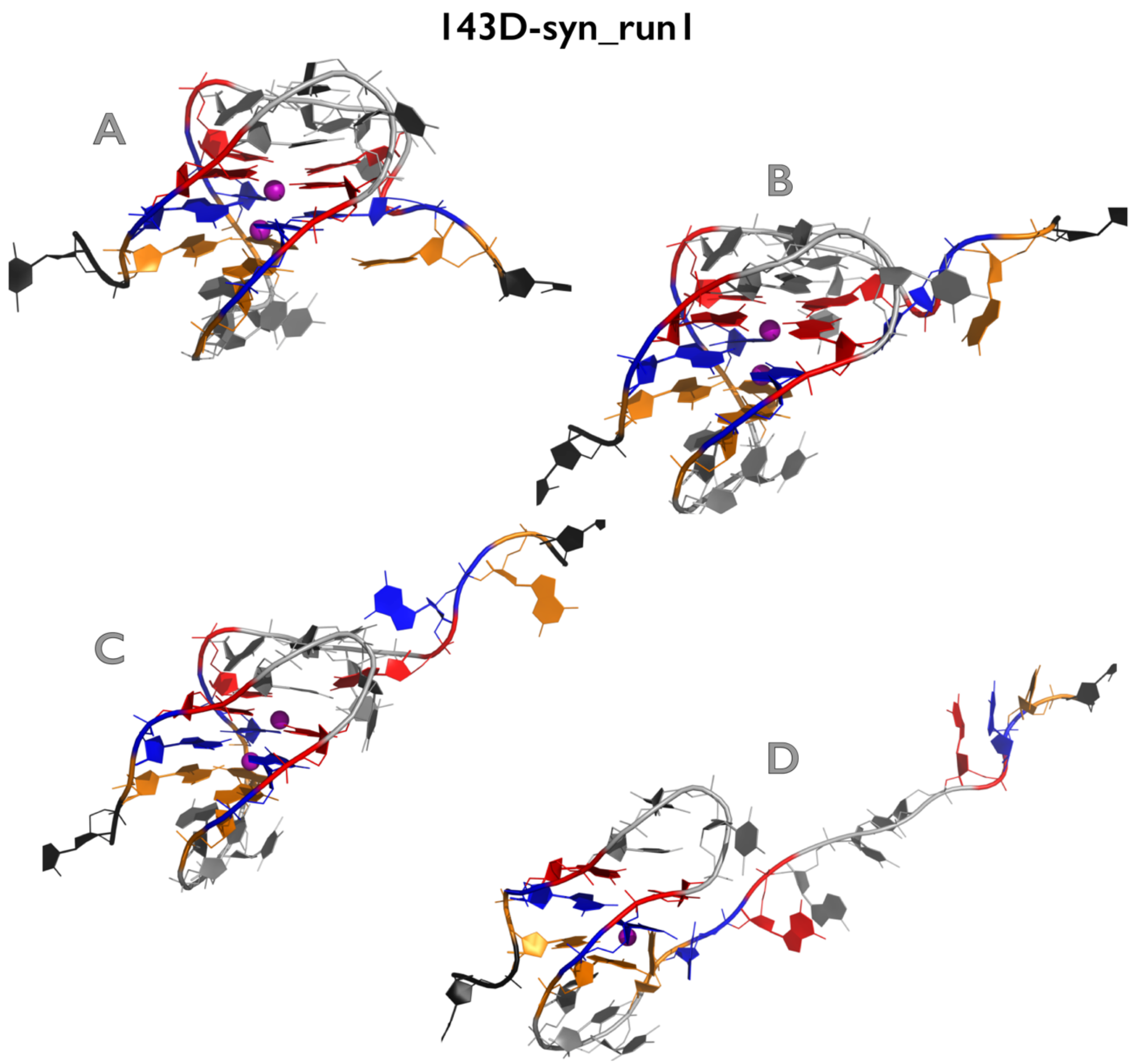

Figure S21B: Most important structural events during first independent *very slow zig-zag pulling* simulation of 143D$_{syn}$ GQ system. See legend of Figure S1B for more details.

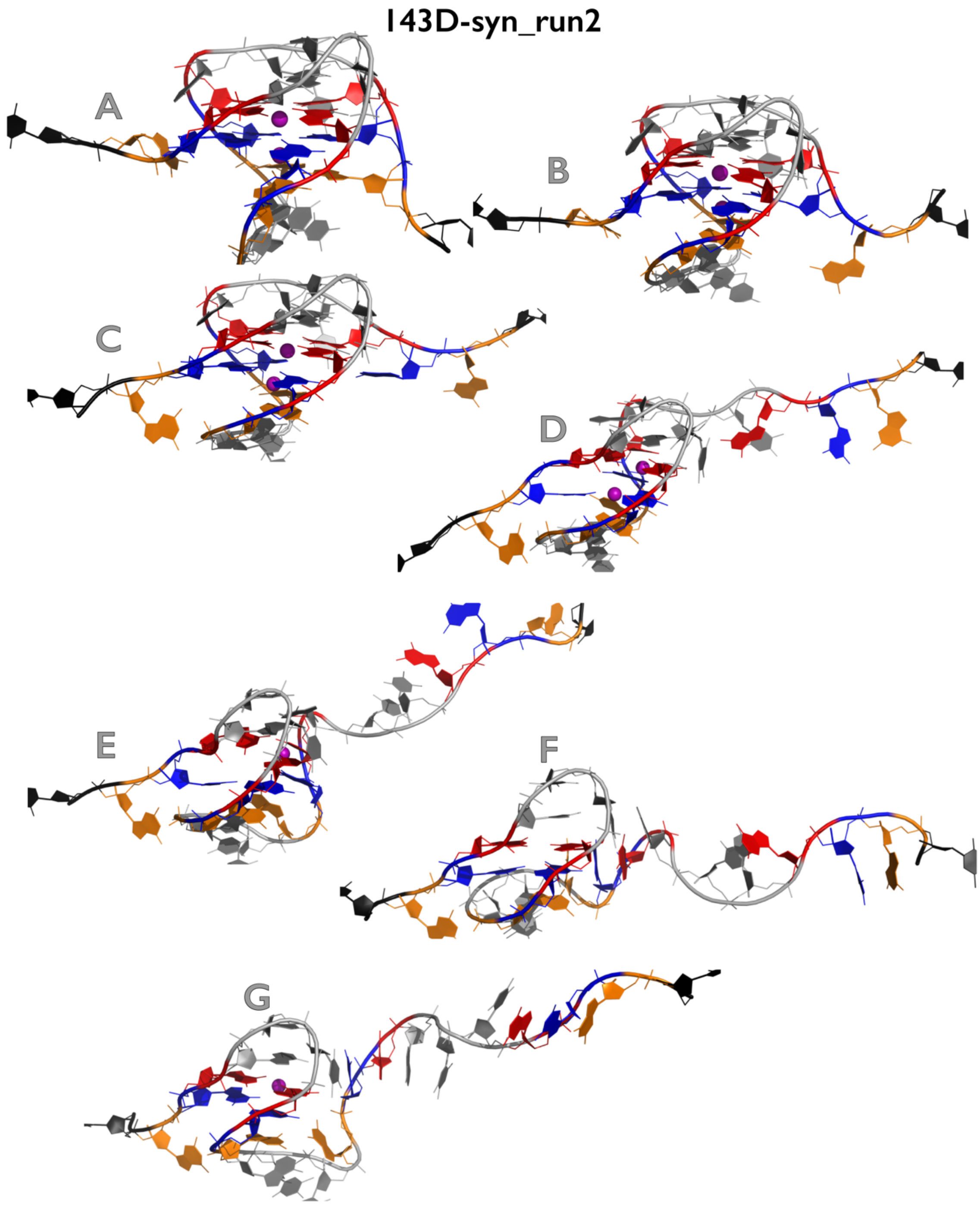

Figure S21C: Most important structural events during second independent *very slow zig-zag pulling* simulation of $143D_{syn}$ GQ system. See legend of Figure S1B for more details.

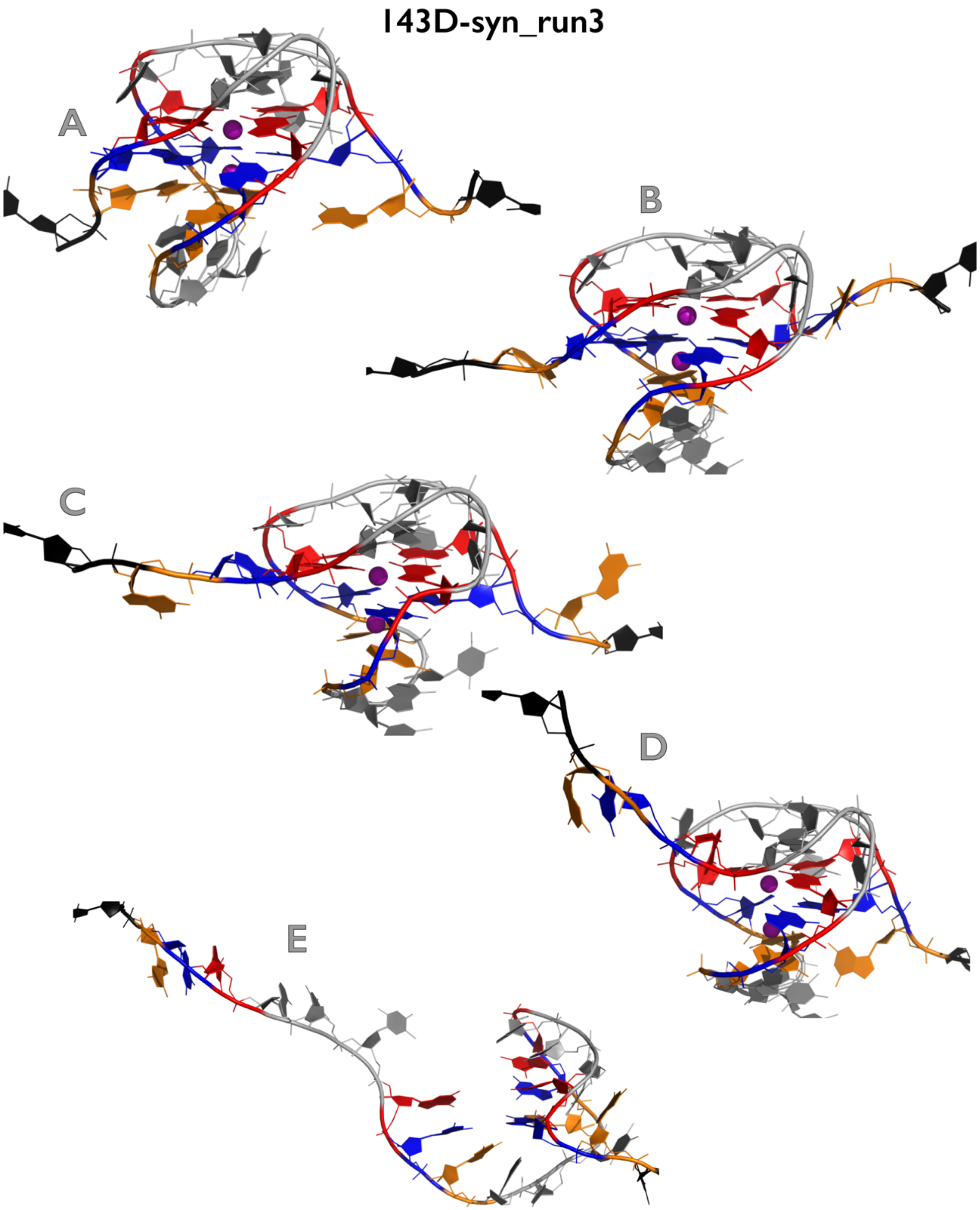

Figure S21D: Most important structural events during third independent *very slow zig-zag pulling* simulation of $143D_{syn}$ GQ system. See legend of Figure S1B for more details.

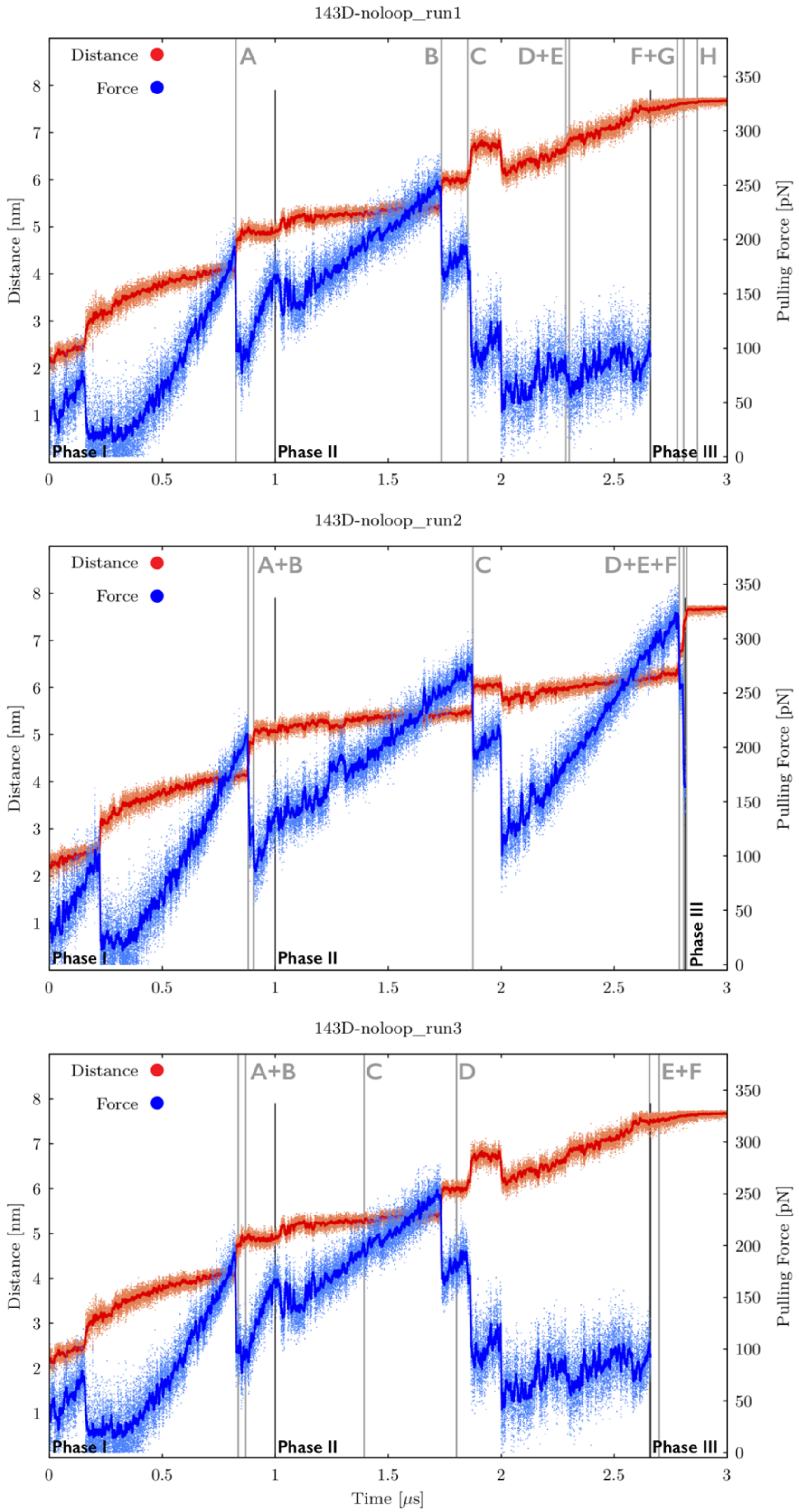

Figure S22A: Time evolution of distance between pulling centers and pulling force during three independent *very slow zig-zag pulling* simulations of 143D$_{noloop}$ GQ system (see legend of Figure

S17A for more details). See Figures S22B-S22D for inspection of structures corresponding to main structural events.

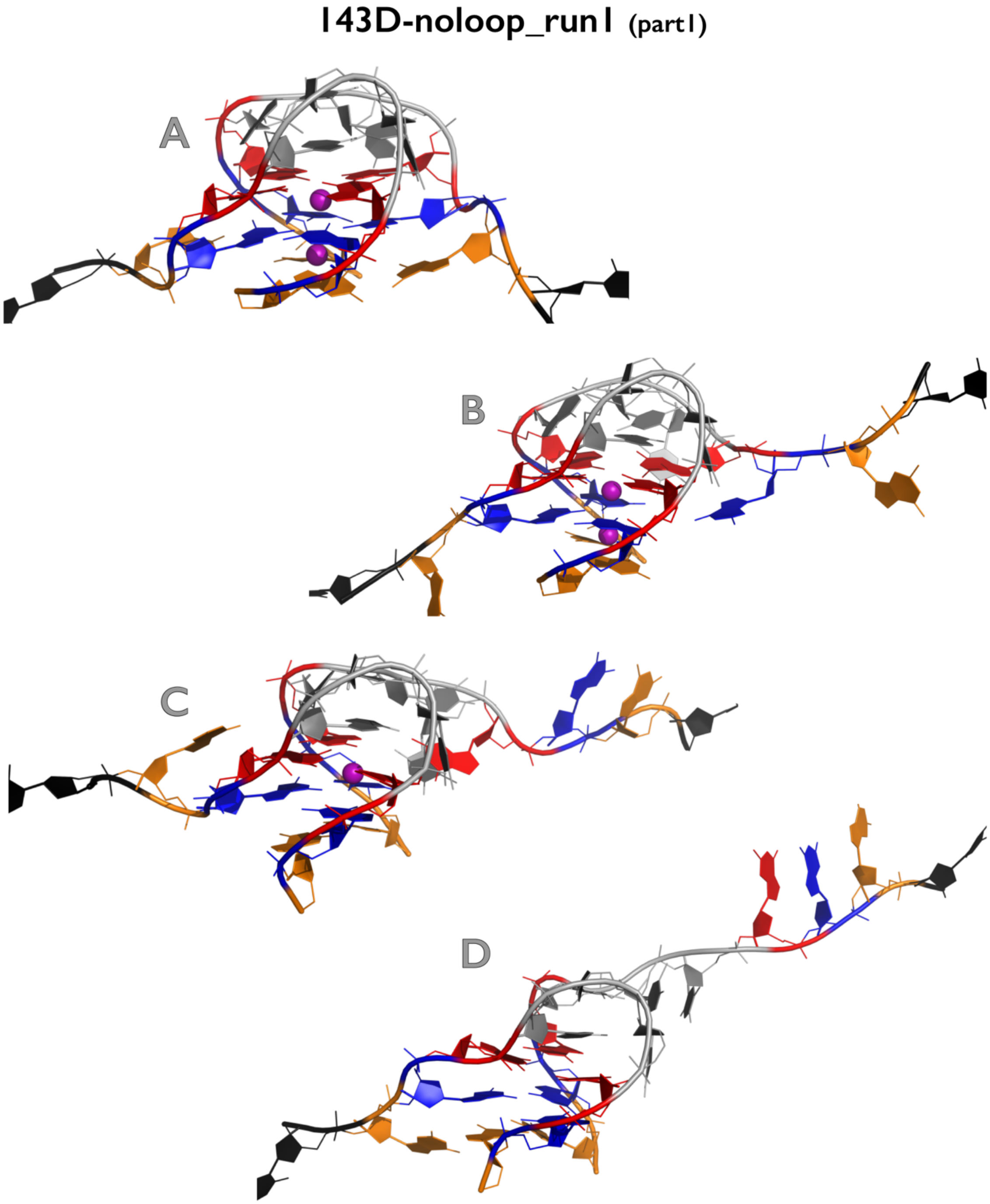

Figure continuing on the next page

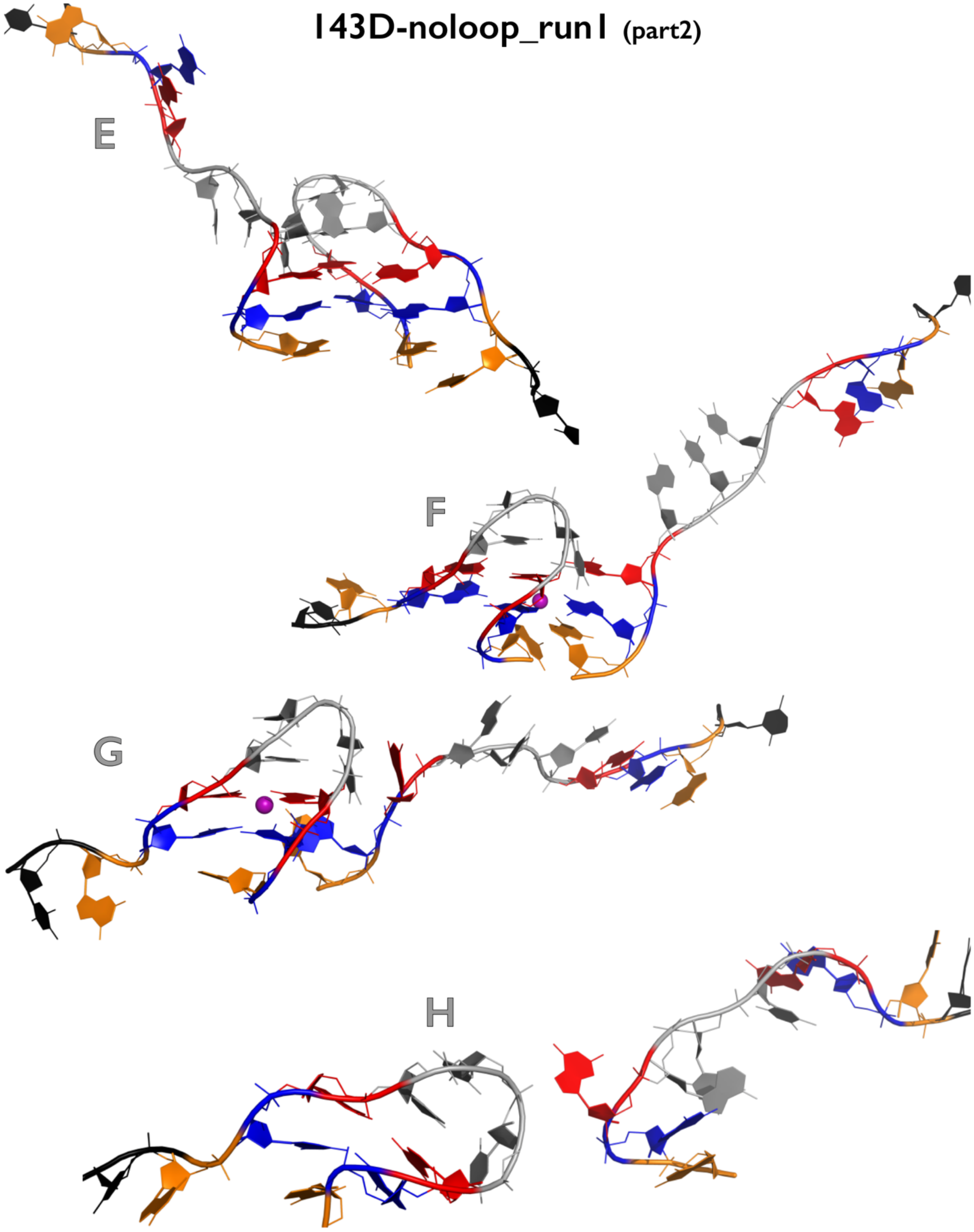

Figure S22B: Most important structural events during first independent *very slow zig-zag pulling* simulation of 143D$_{noloop}$ GQ system. See legend of Figure S1B for more details.

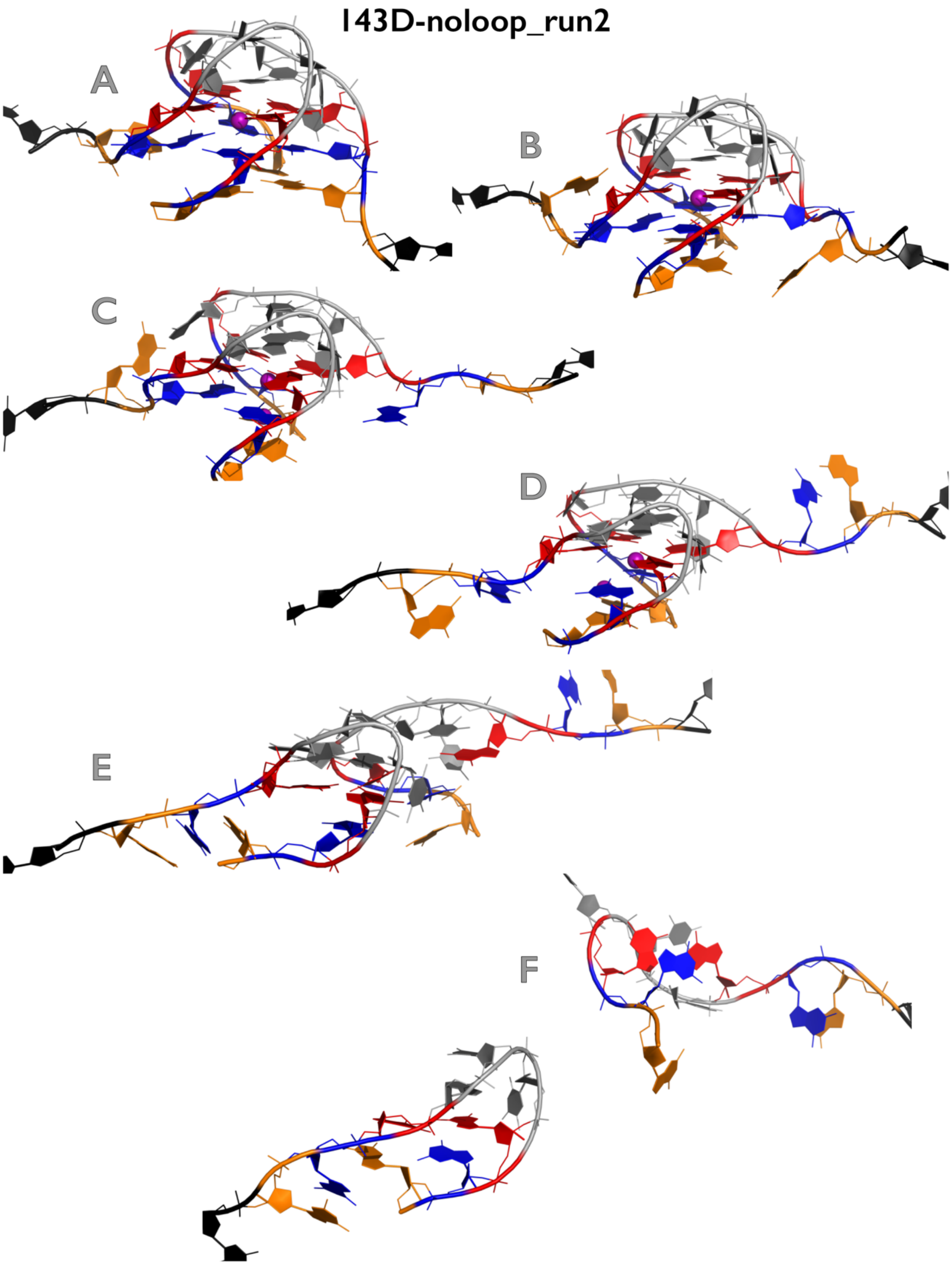

Figure S22C: Most important structural events during second independent *very slow zig-zag pulling* simulation of $143D_{noloop}$ GQ system. See legend of Figure S1B for more details.

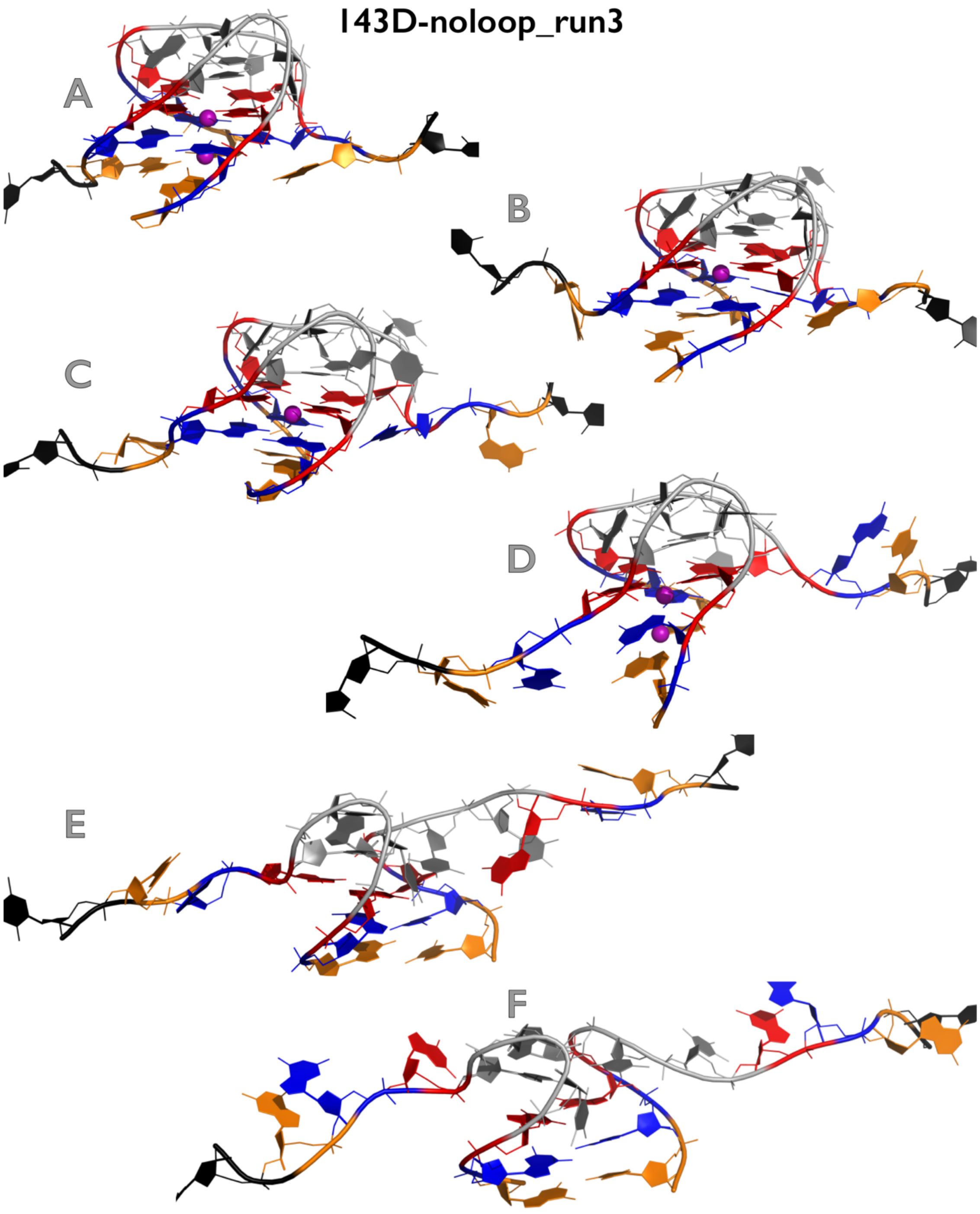

Figure S22D: Most important structural events during third independent *very slow zig-zag pulling* simulation of 143D$_{noloop}$ GQ system. See legend of Figure S1B for more details.

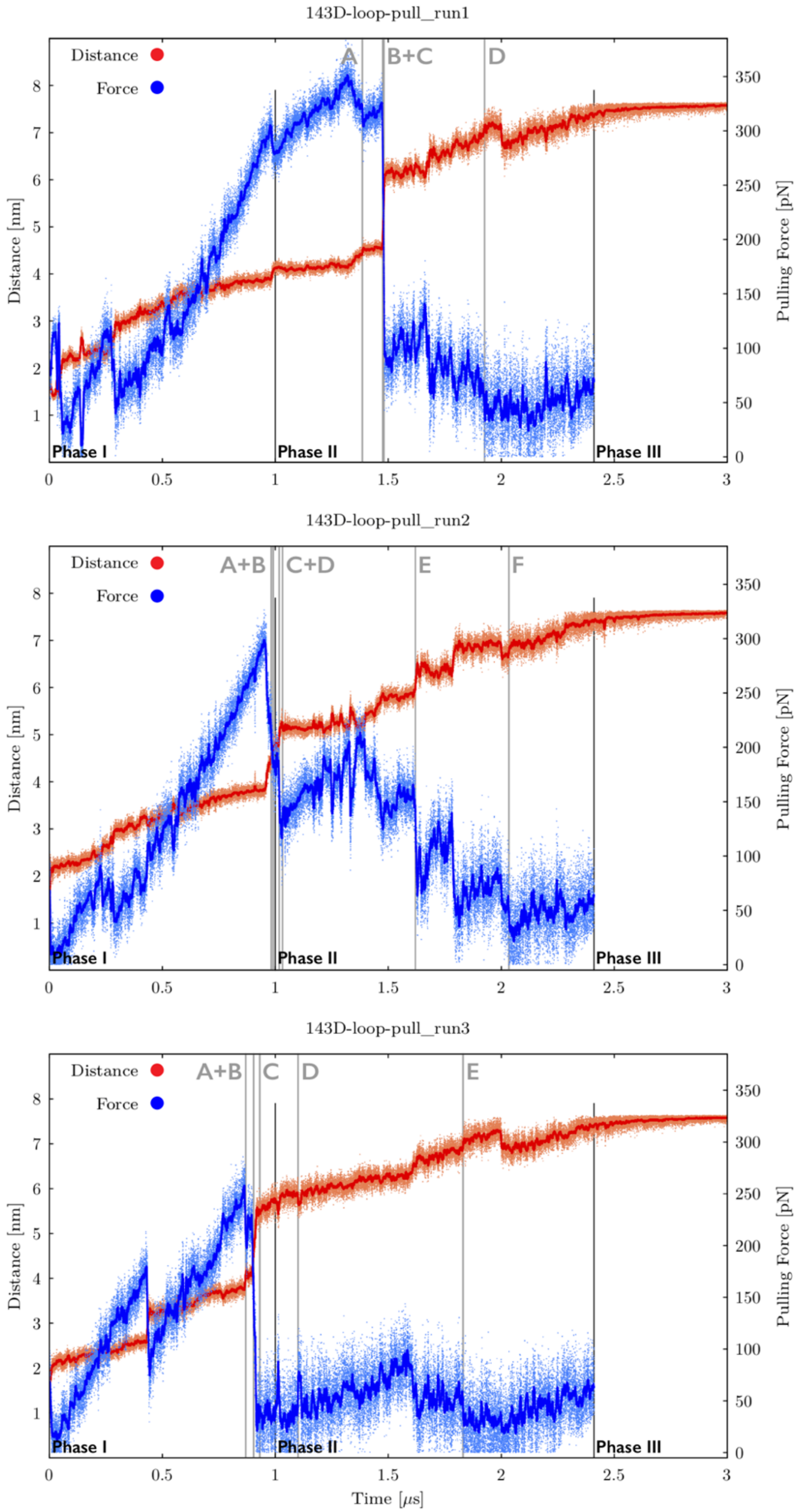

Figure S23A: Time evolution of distance between pulling centers and pulling force during three independent *very slow zig-zag pulling* simulations of 143D$_{loop\text{-}pull}$ GQ system (see legend of Figure

S17A for more details). See Figures S23B-S23D for inspection of structures corresponding to main structural events.

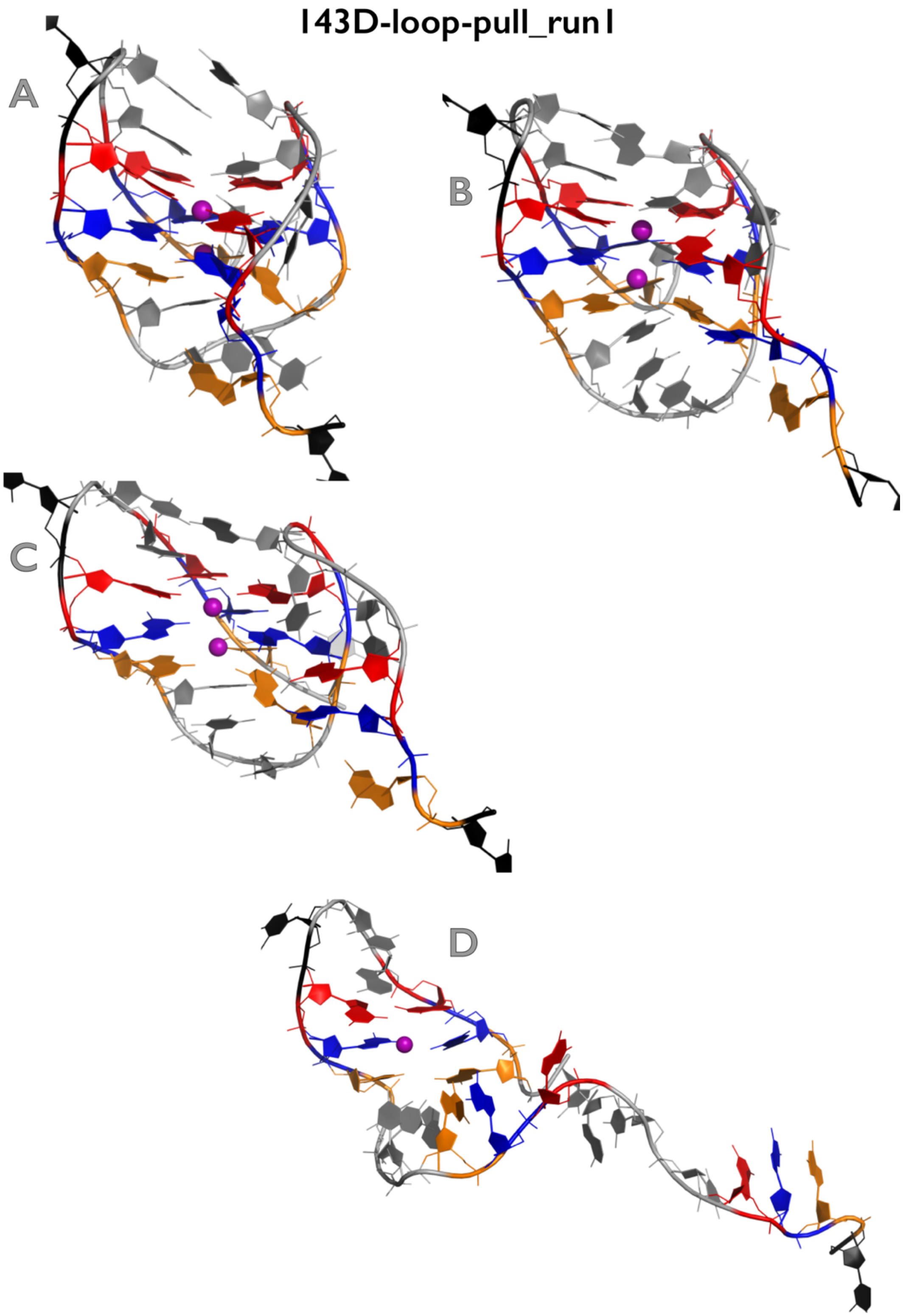

Figure S23B: Most important structural events during first independent *very slow zig-zag pulling* simulation of $143D_{loop\text{-}pull}$ GQ system. See legend of Figure S1B for more details.

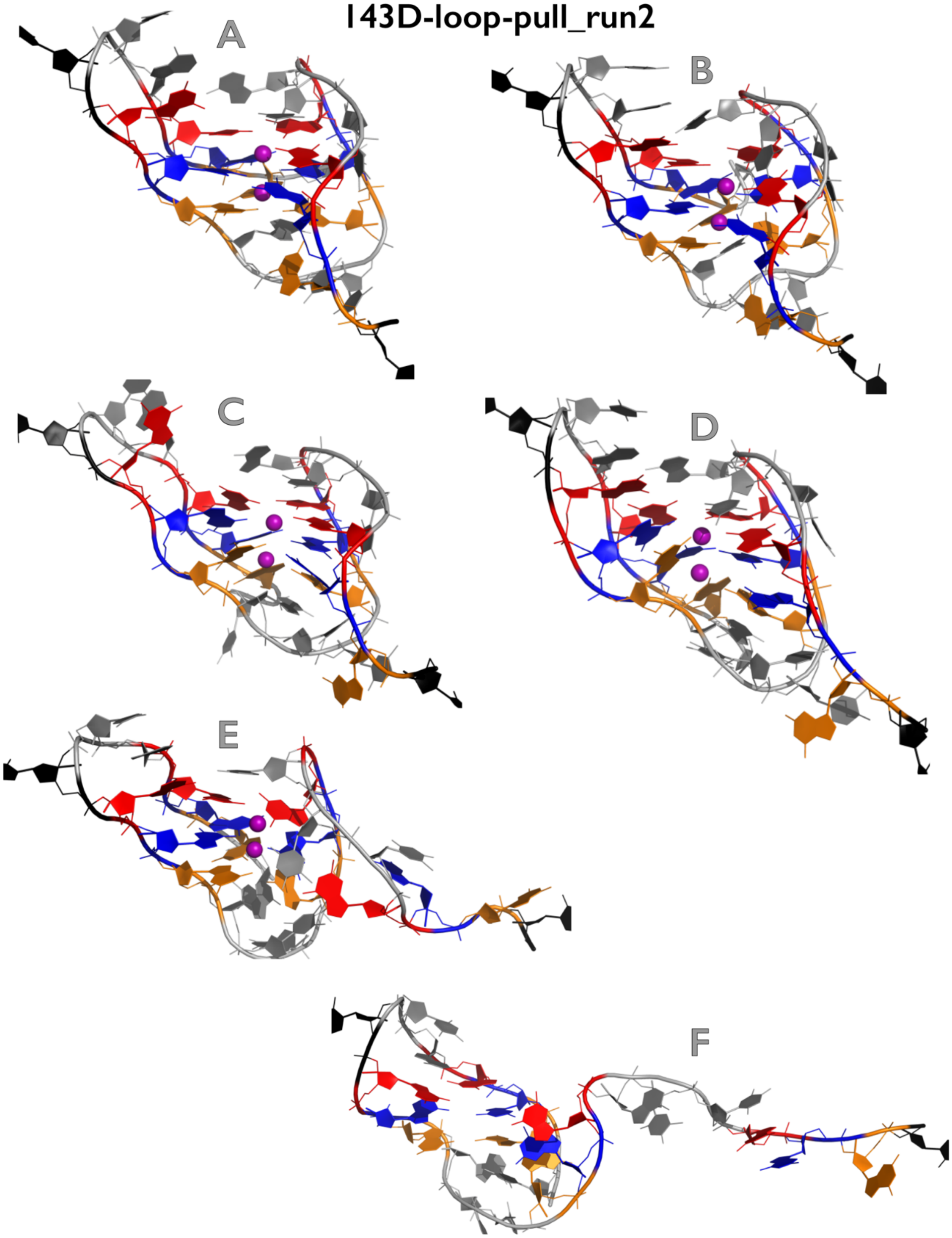

Figure S23C: Most important structural events during second independent *very slow zig-zag pulling* simulation of 143D$_{loop-pull}$ GQ system. See legend of Figure S1B for more details.

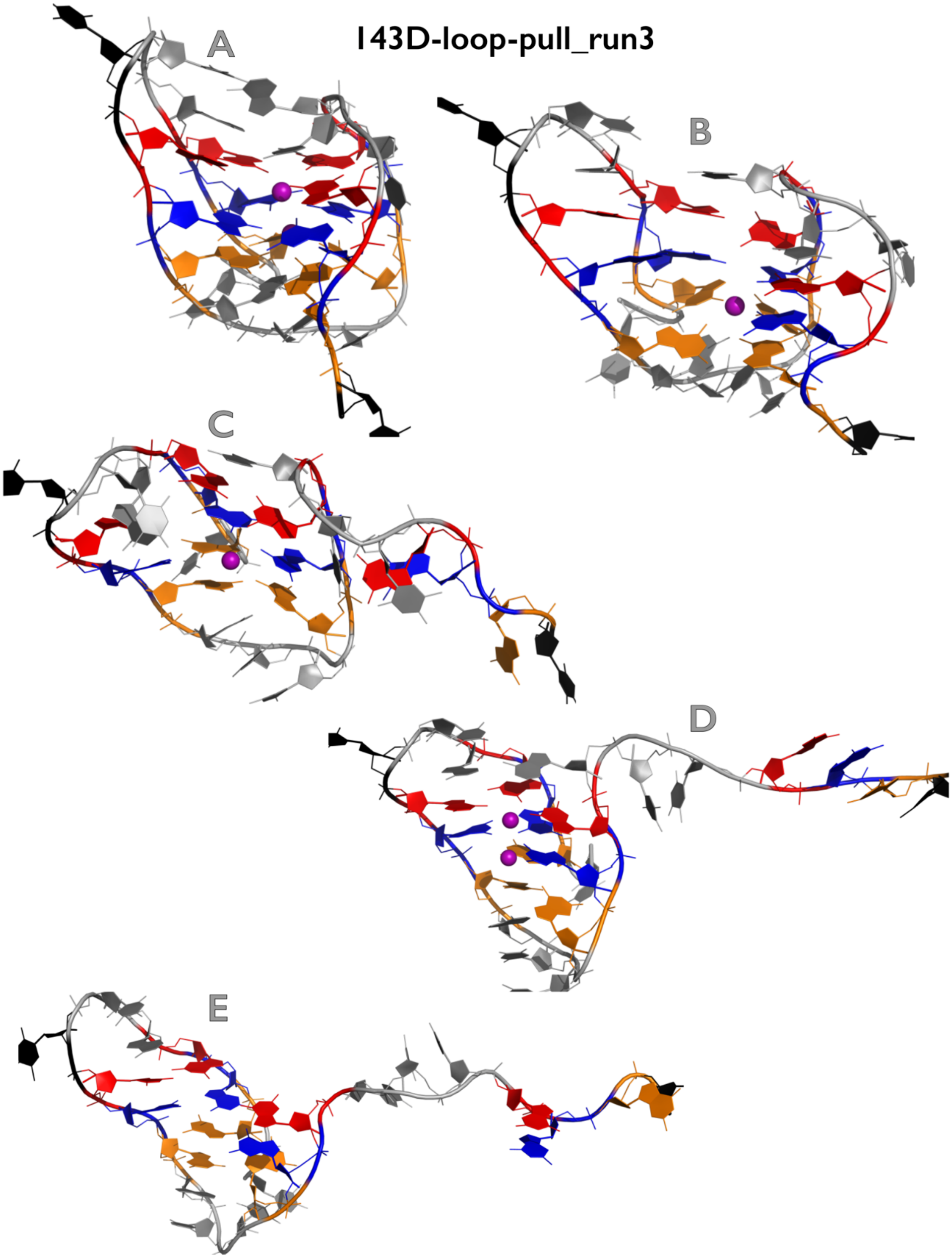

Figure S23D: Most important structural events during third independent *very slow zig-zag pulling* simulation of $143D_{loop\text{-}pull}$ GQ system. See legend of Figure S1B for more details.

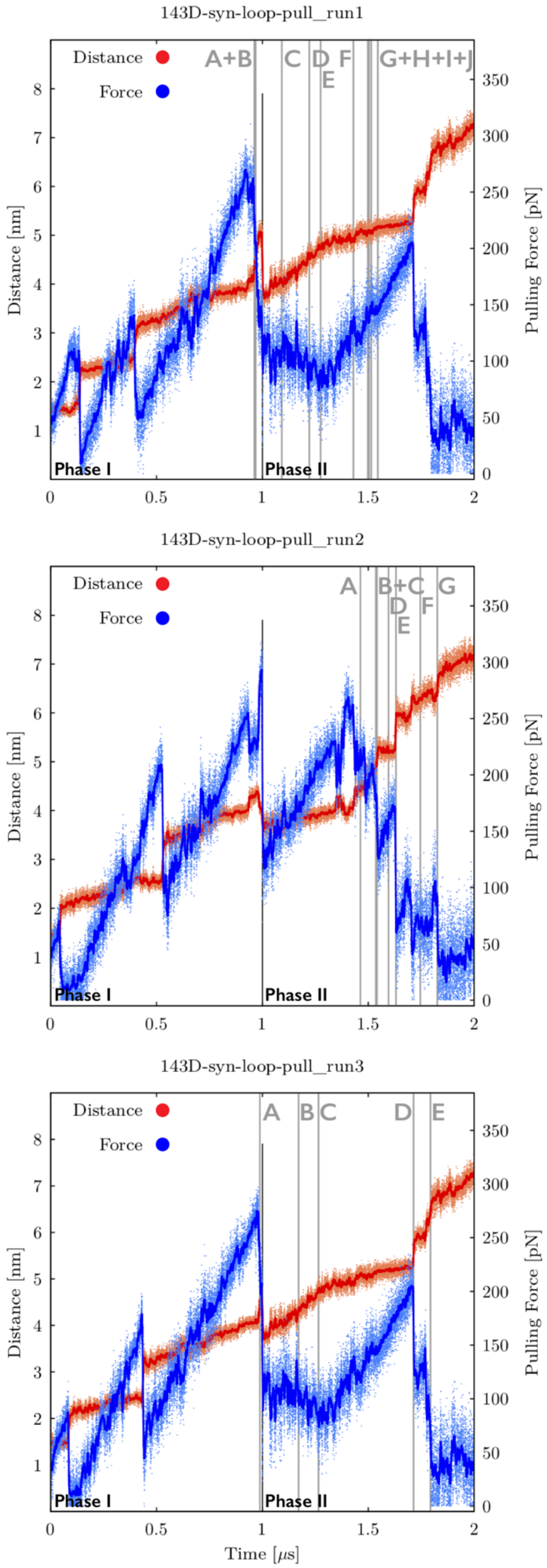

Figure S24A: Time evolution of distance between pulling centers and pulling force during three independent *very slow zig-zag pulling* simulations of 143D$_{syn_loop\text{-}pull}$ GQ system (see legend of

Figure S17A for more details). See Figures S24B-S24D for inspection of structures corresponding to main structural events.

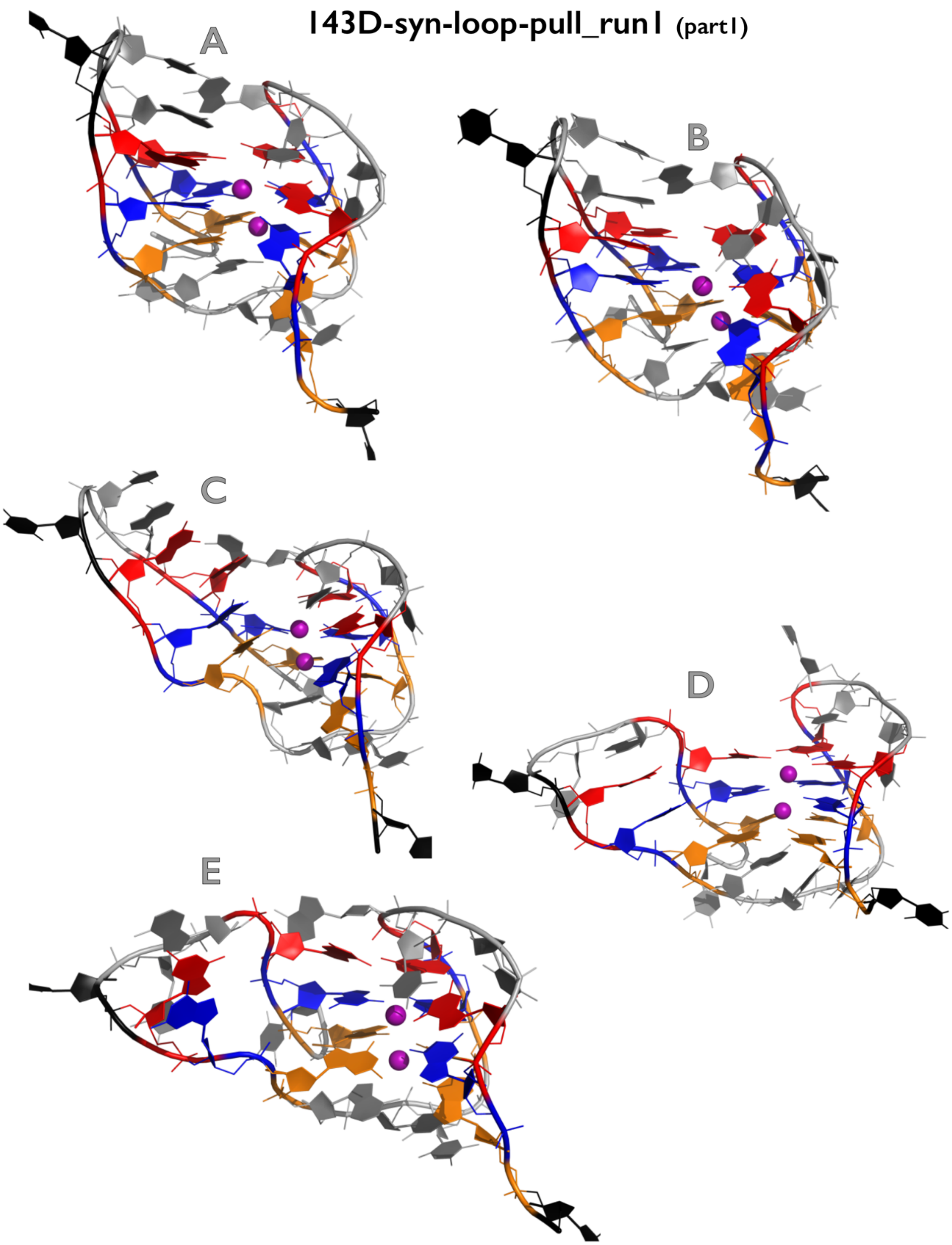

Figure continuing on the next page

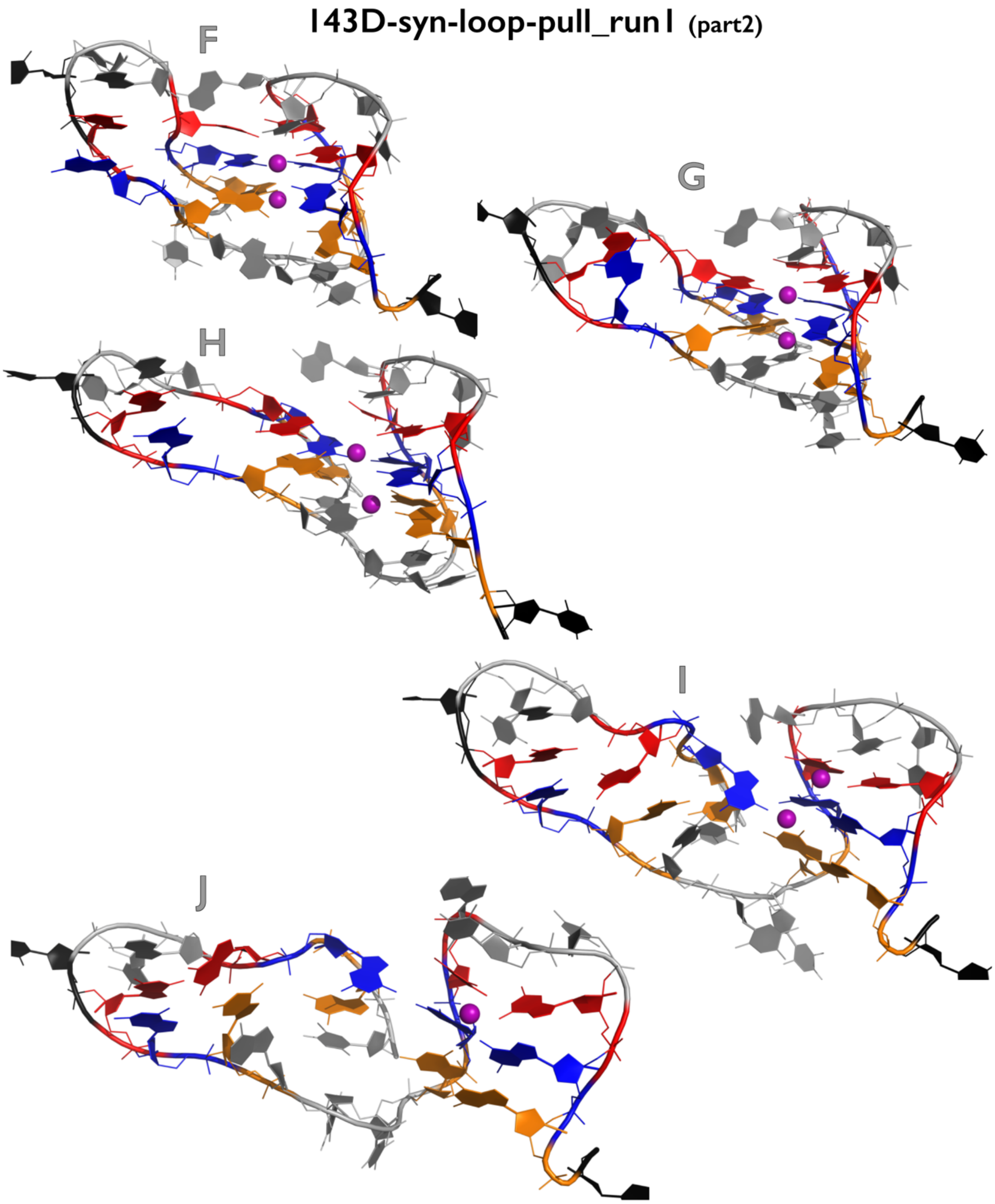

Figure S24B: Most important structural events during first independent *very slow zig-zag pulling* simulation of 143D$_{syn_loop-pull}$ GQ system. See legend of Figure S1B for more details.

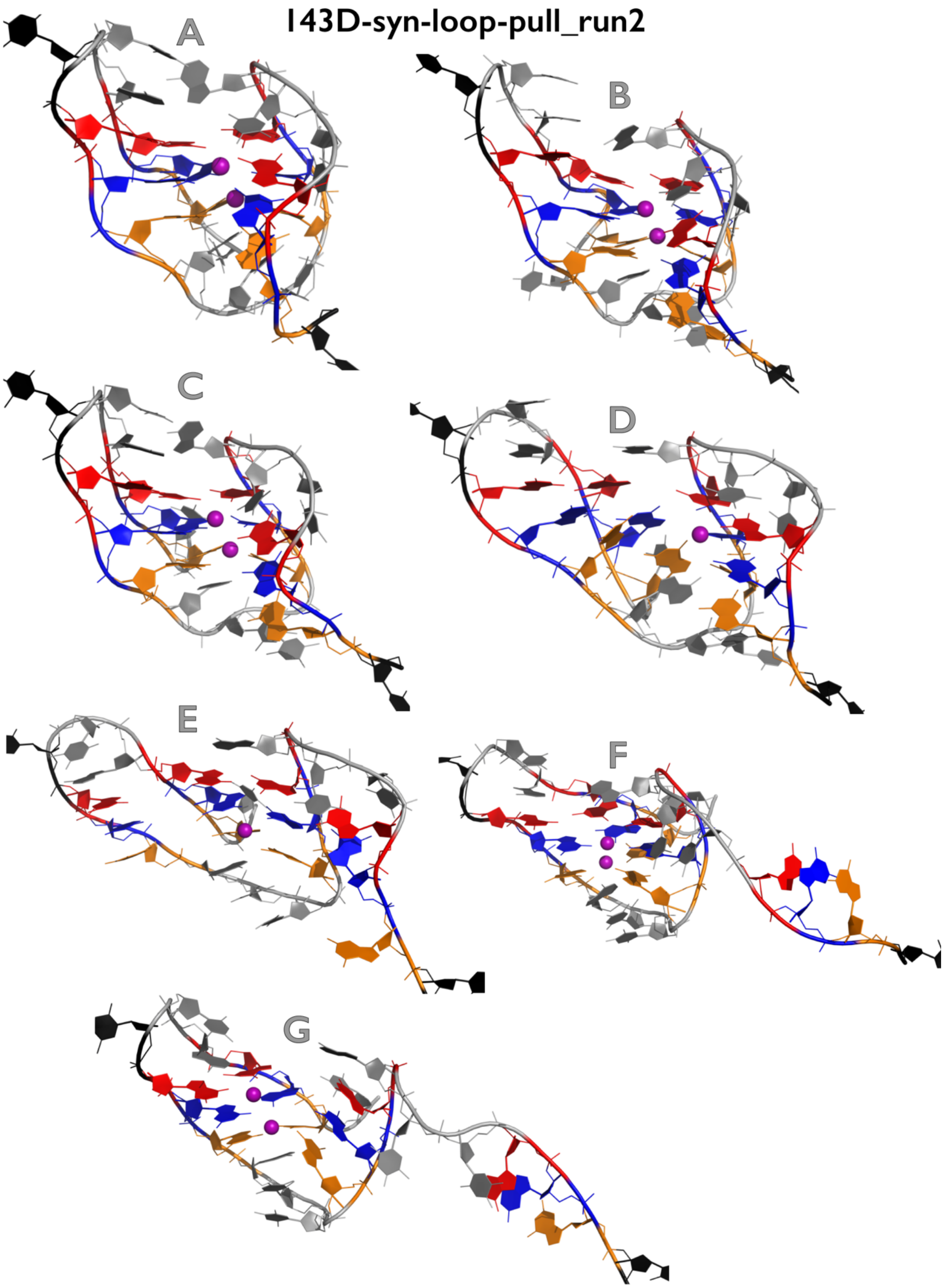

Figure S24C: Most important structural events during second independent *very slow zig-zag pulling* simulation of $143D_{syn\_loop\text{-}pull}$ GQ system. See legend of Figure S1B for more details.

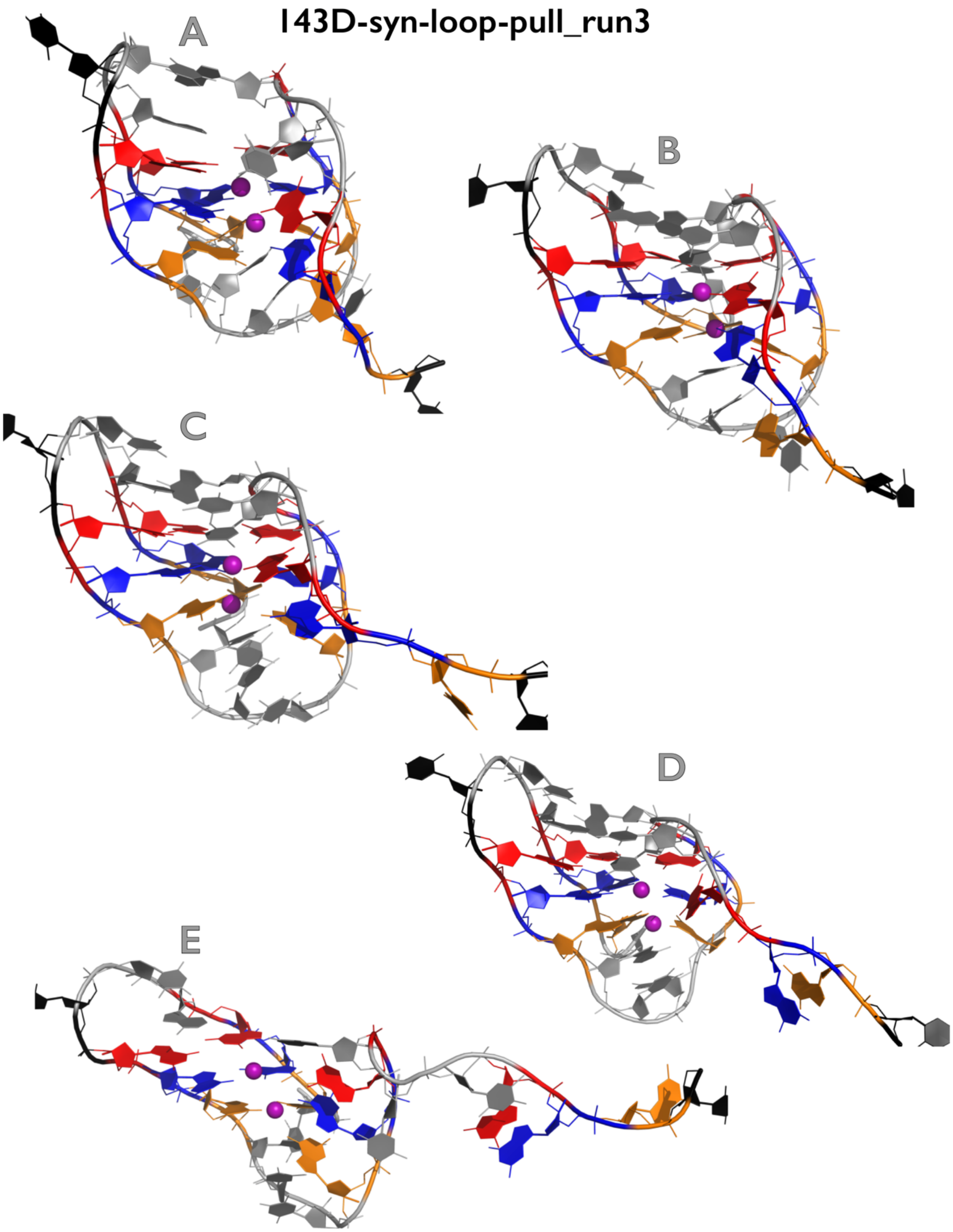

Figure S24D: Most important structural events during third independent *very slow zig-zag pulling* simulation of $143D_{syn\_loop\text{-}pull}$ GQ system. See legend of Figure S1B for more details.

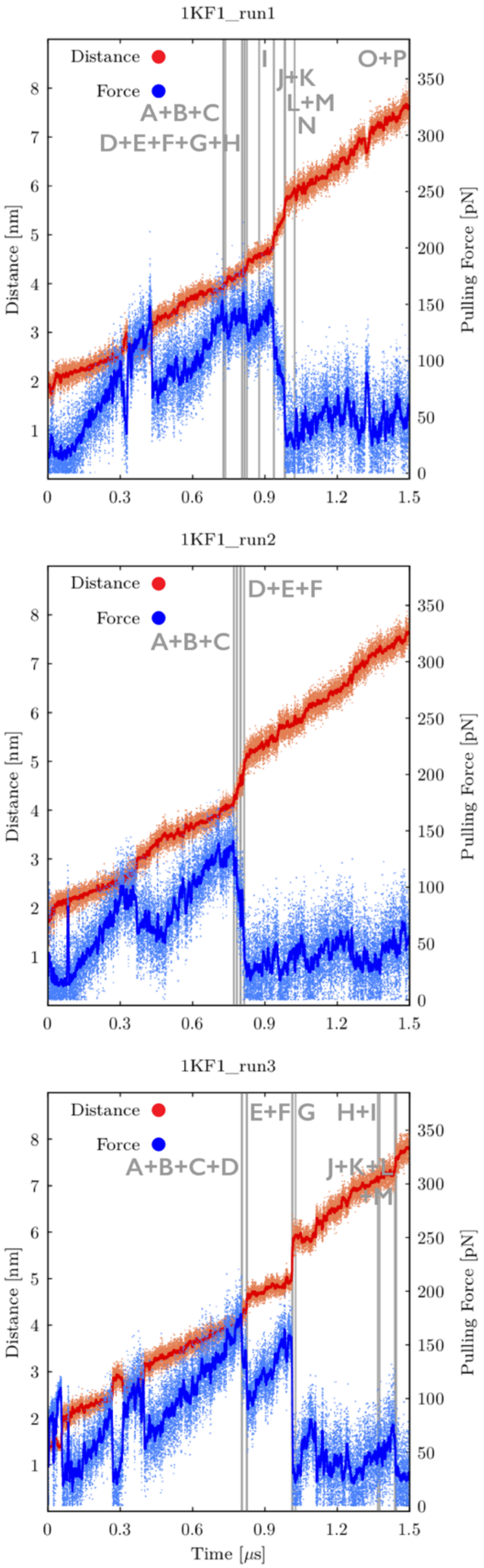

Figure S25A: Time evolution of distance between pulling centers and pulling force during three independent *very slow pulling* simulations of 1KF1 GQ system. Snapshots were saved every 50 ps and plots are showing both instantaneous values (orange and light-blue dots for distance and force,

respectively) and smoothing, i.e., averaging over 100 consecutive snapshots (red and blue lines for distance and force, respectively). Main structural events are highlighted as grey vertical lines with labels (capital letters). See Figures S25B-S25D for inspection of structures corresponding to main structural events. Note that first major drops of the pulling force before the GQ unfolding event "A" (and notable prolongation of end-to-end distances) are connected with repositioning of terminal T residues.

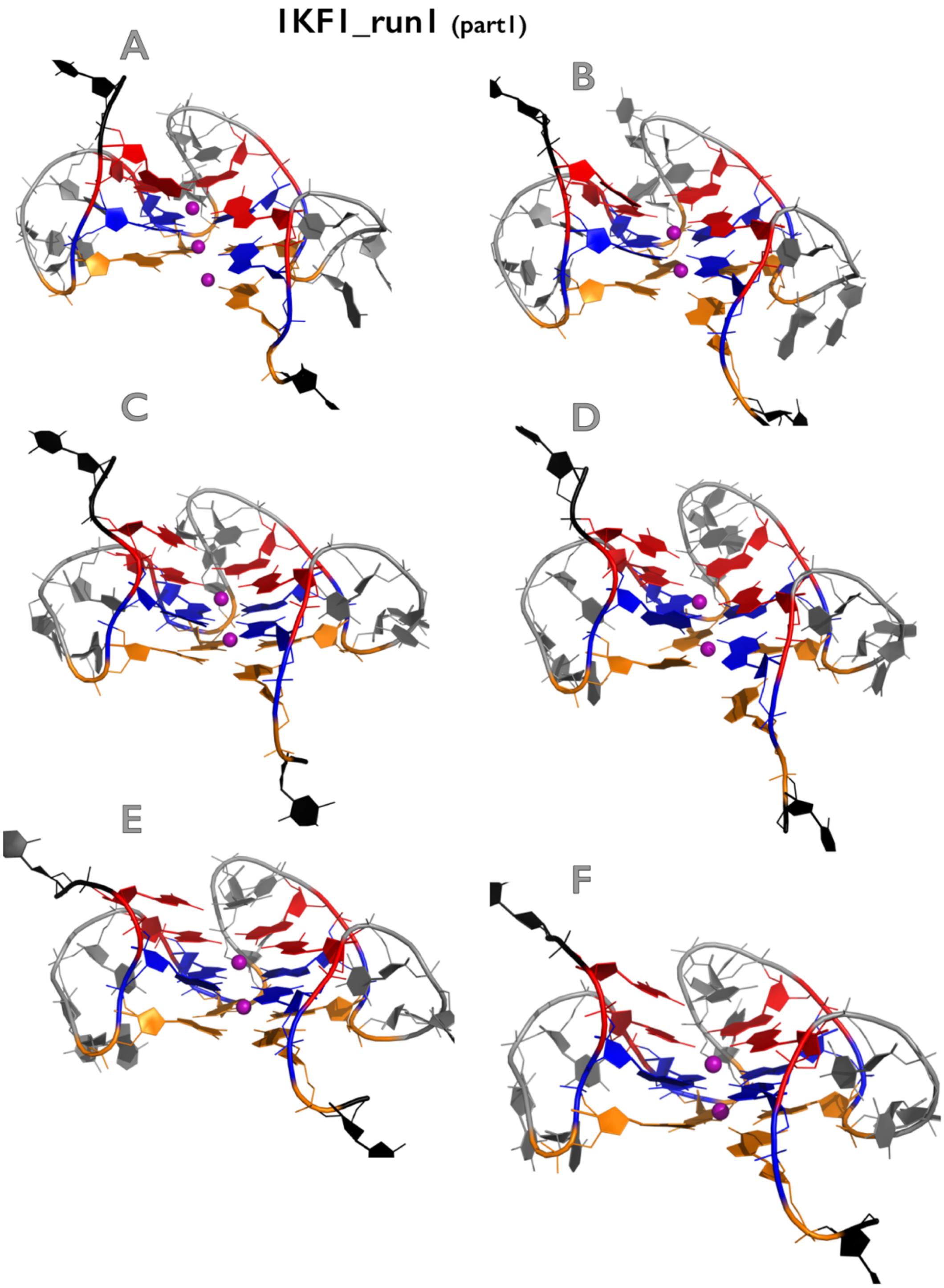

Figure continuing on the next page

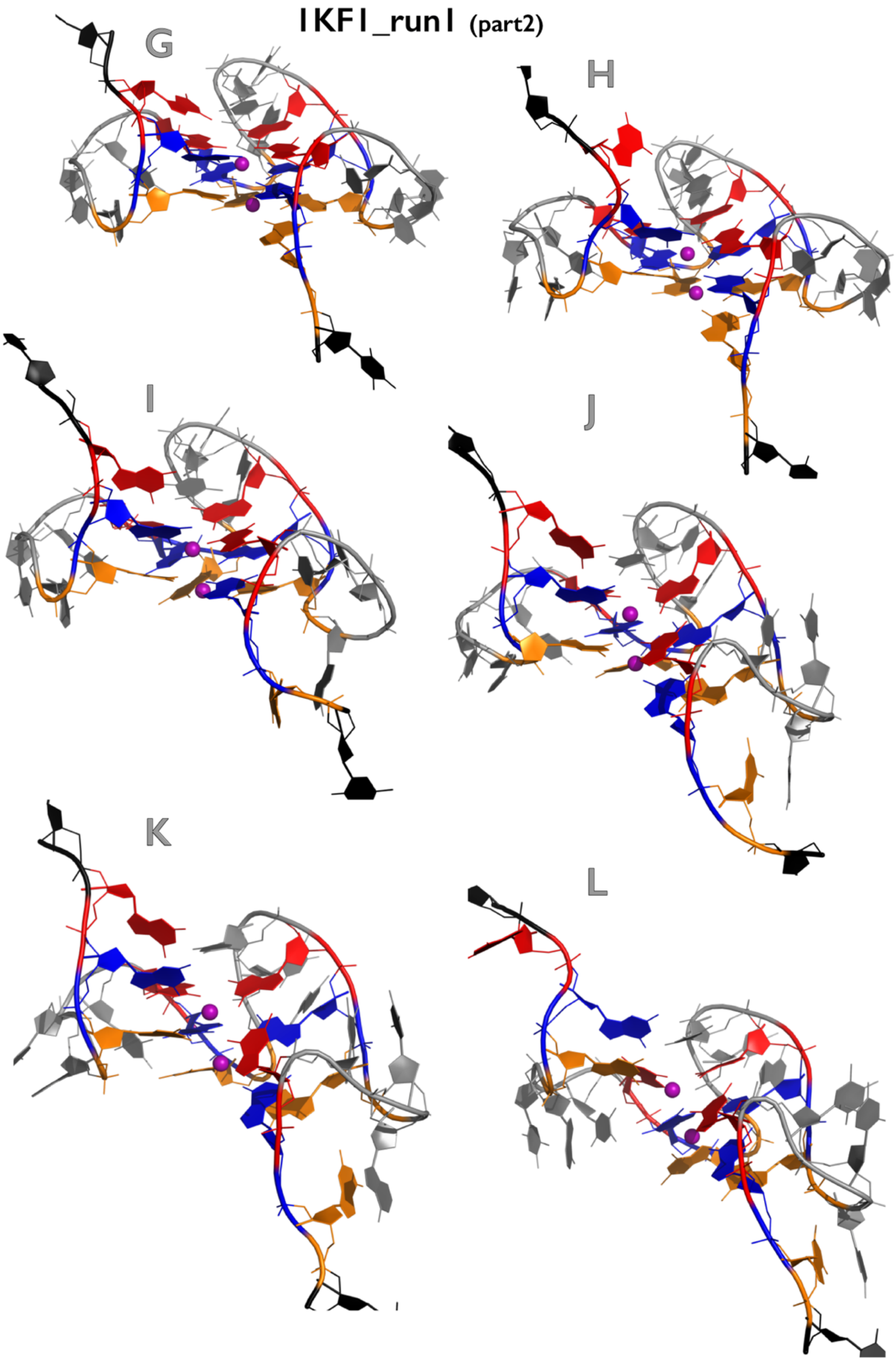

Figure continuing on the next page

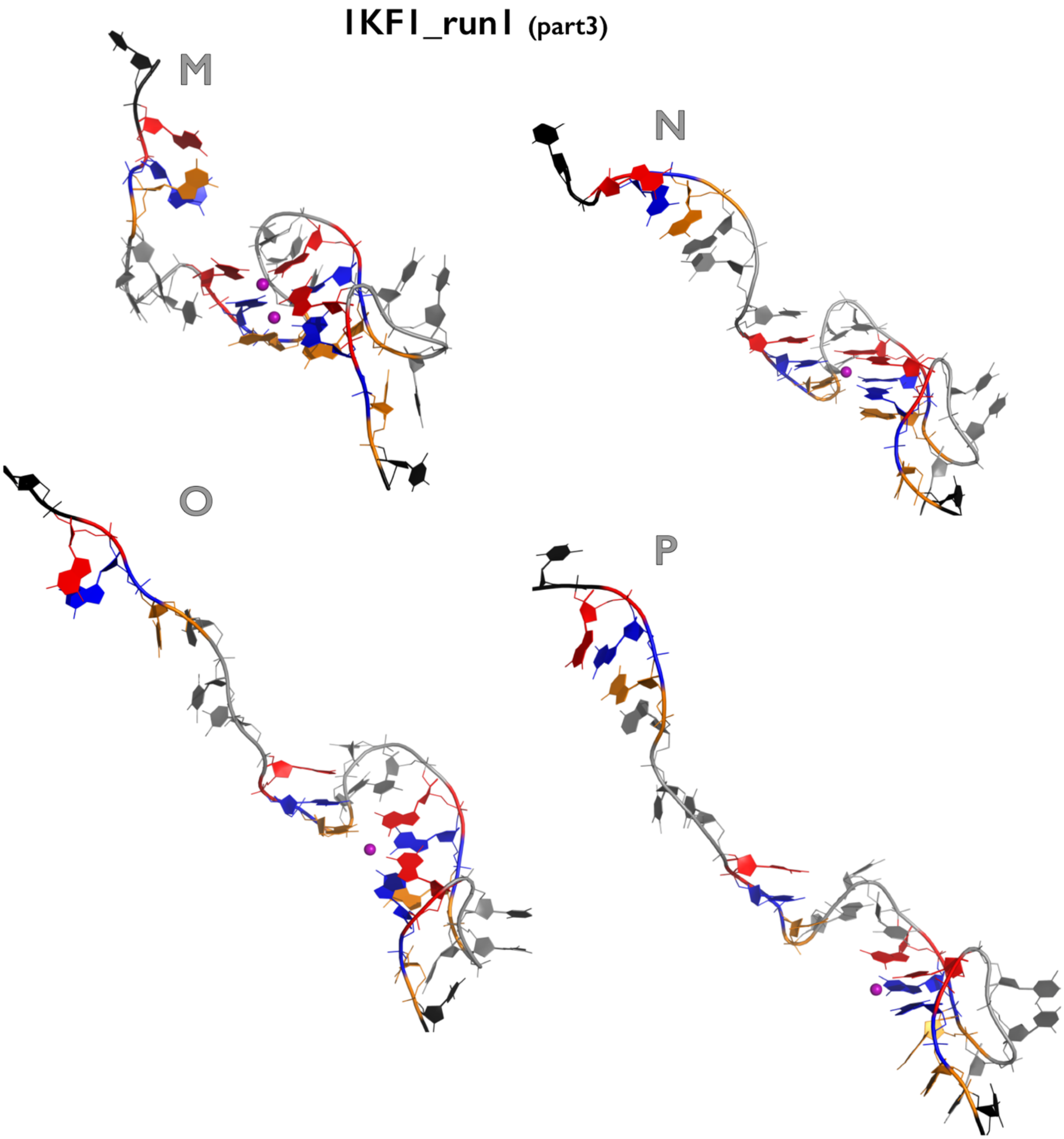

Figure S25B: Most important structural events during first independent *very slow pulling* simulation of 1KF1 GQ system. See legend of Figure S1B for more details.

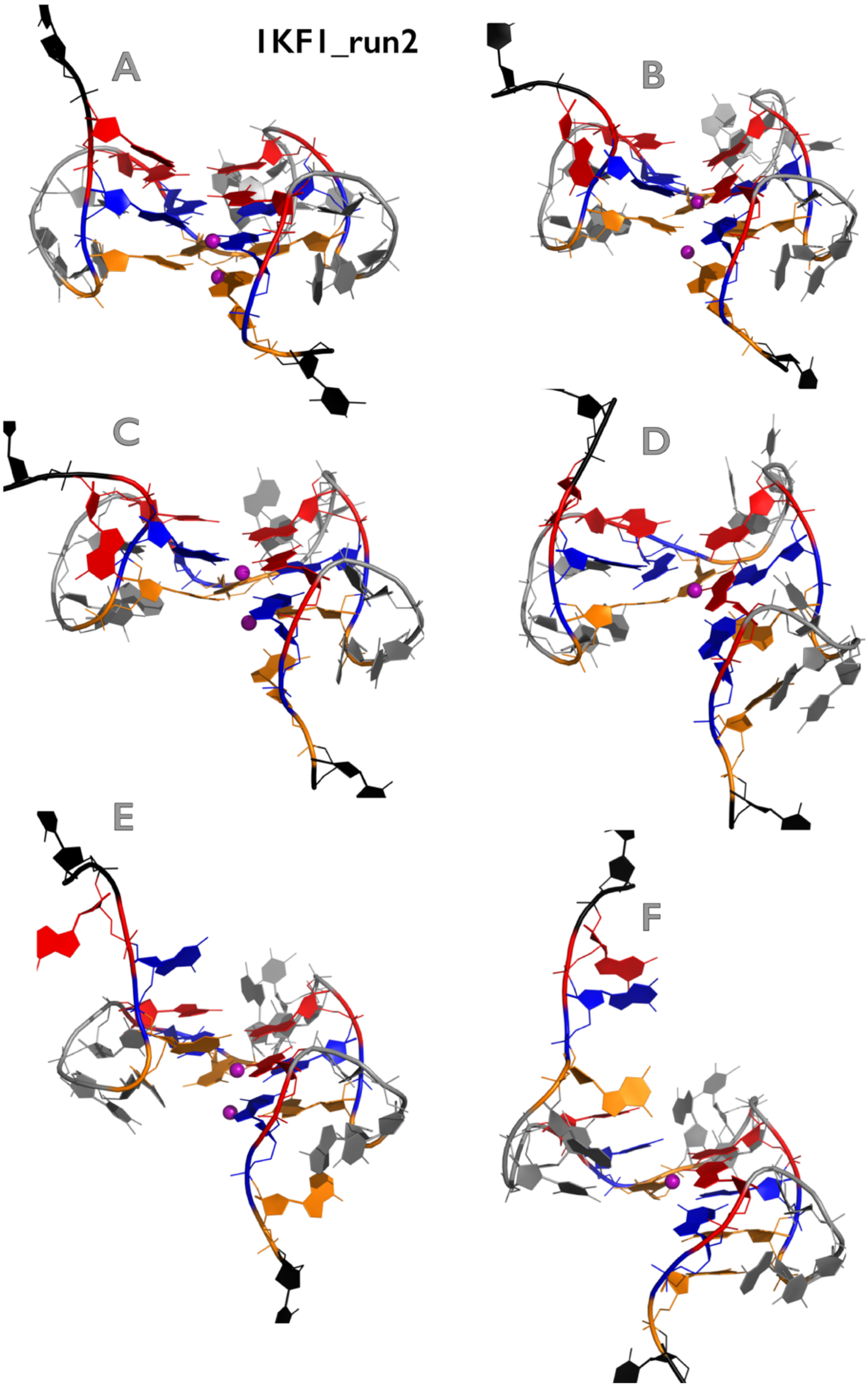

Figure S25C: Most important structural events during second independent *very slow pulling* simulation of 1KF1 GQ system. See legend of Figure S1B for more details.

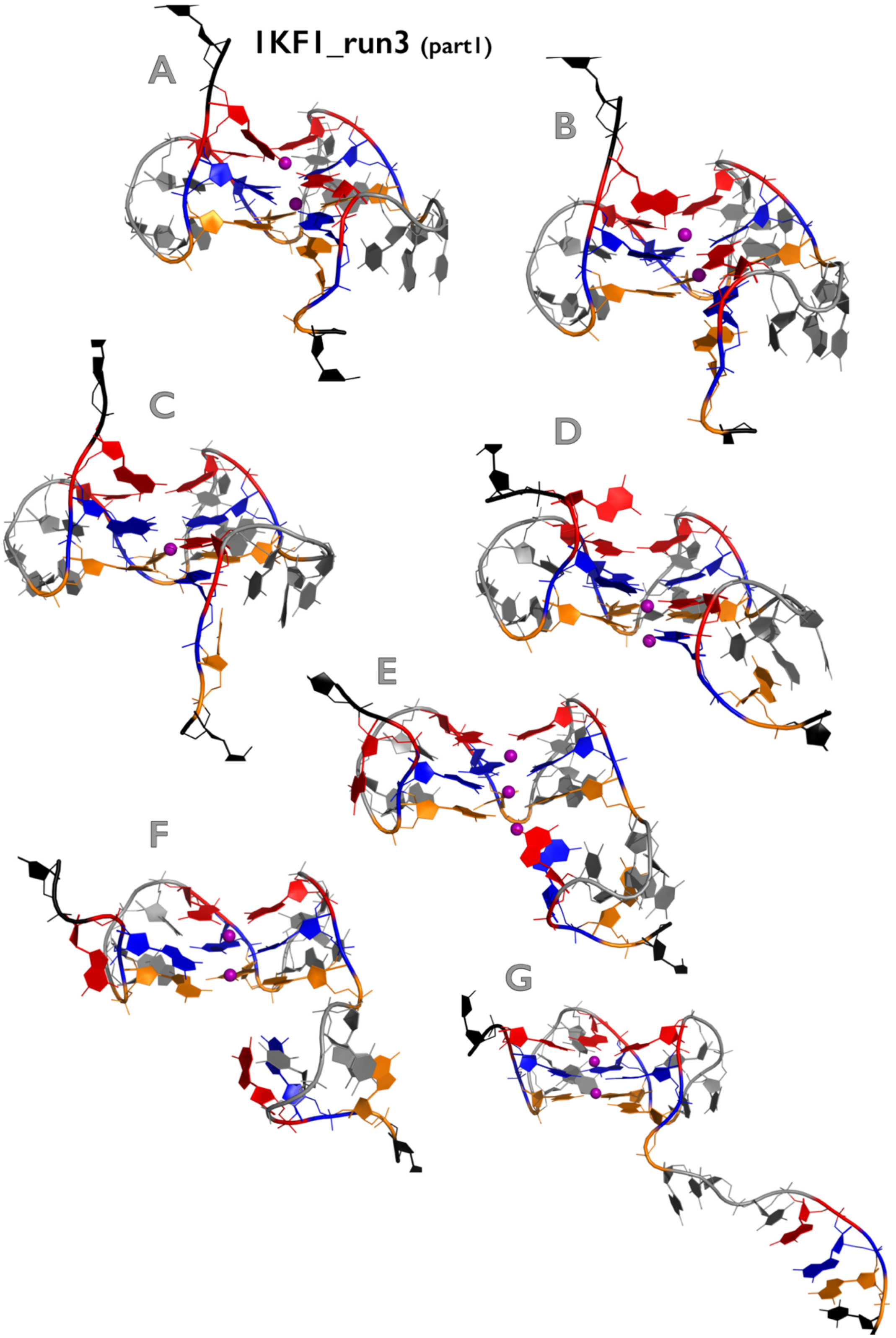

Figure continuing on the next page

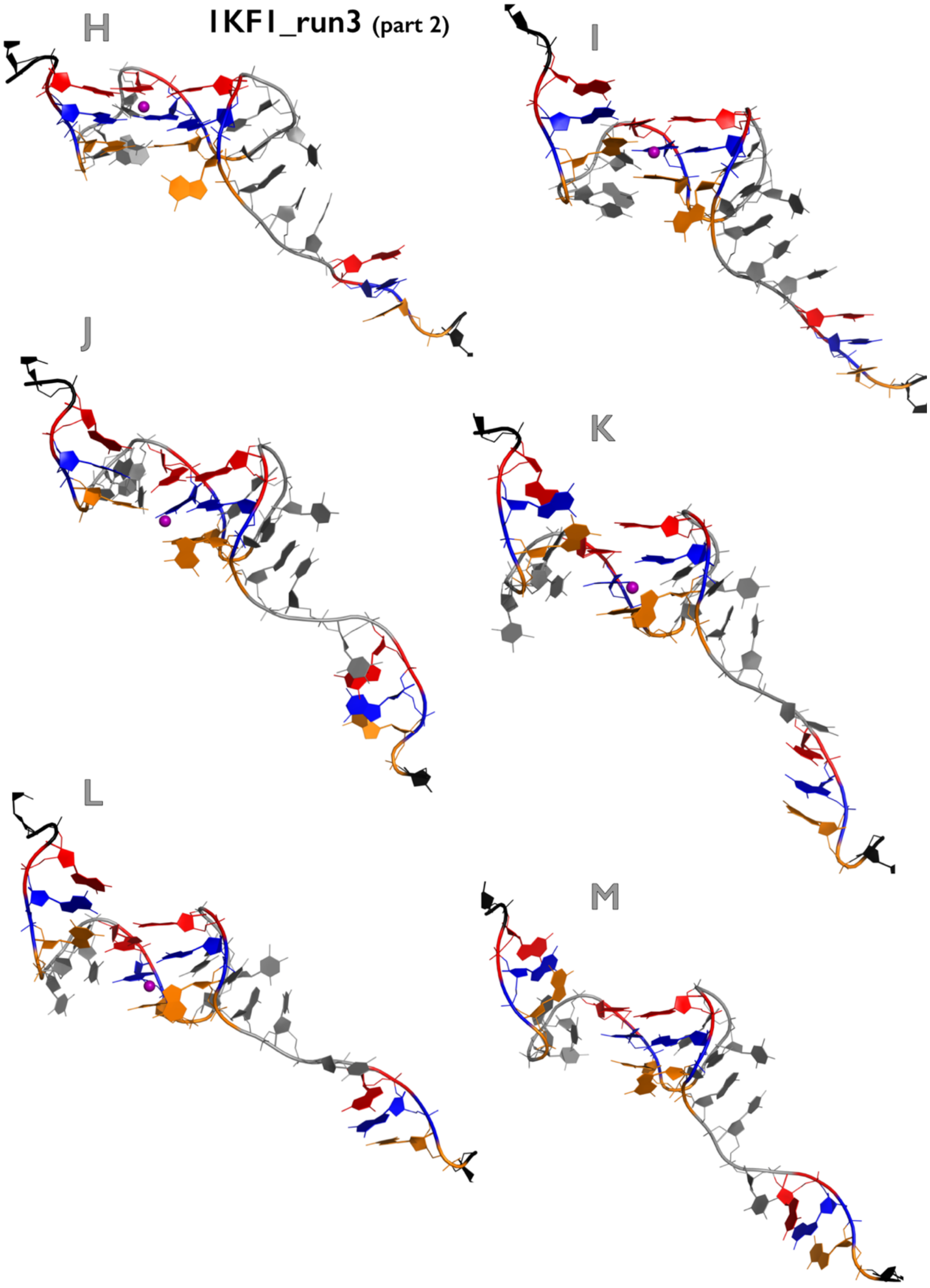

Figure S25D: Most important structural events during third independent *very slow pulling* simulation of 1KF1 GQ system. See legend of Figure S1B for more details.

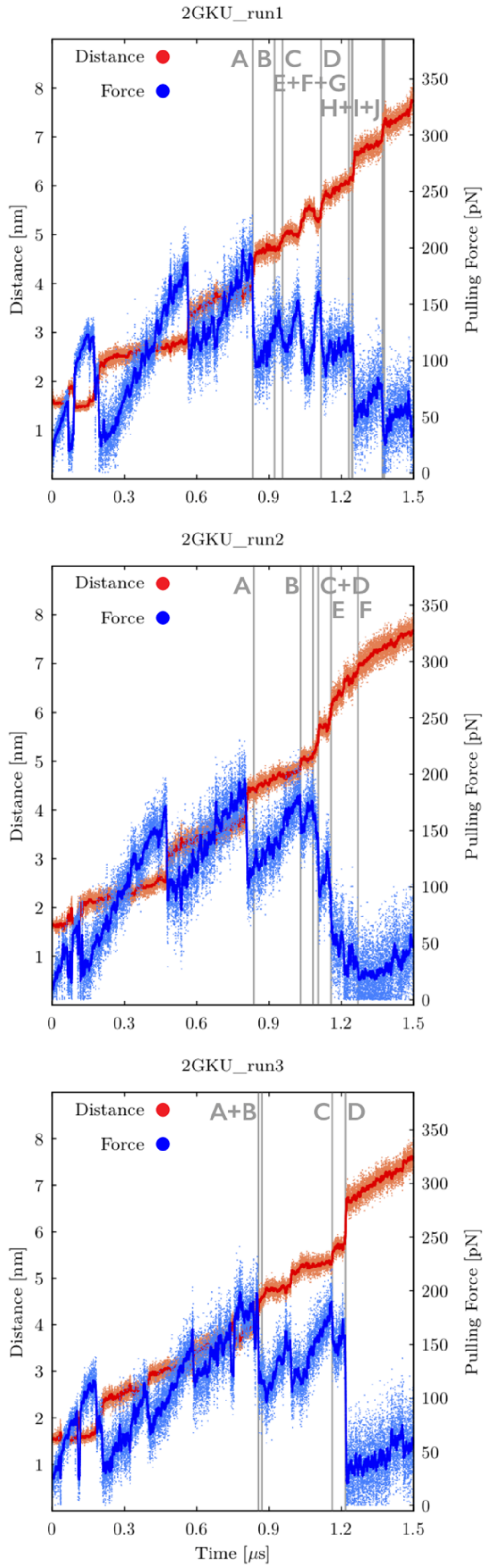

Figure S26A: Time evolution of distance between pulling centers and pulling force during three independent *very slow pulling* simulations of 2GKU GQ system (see legend of Figure S25A for

more details). See Figures S26B-S26D for inspection of structures corresponding to main structural events.

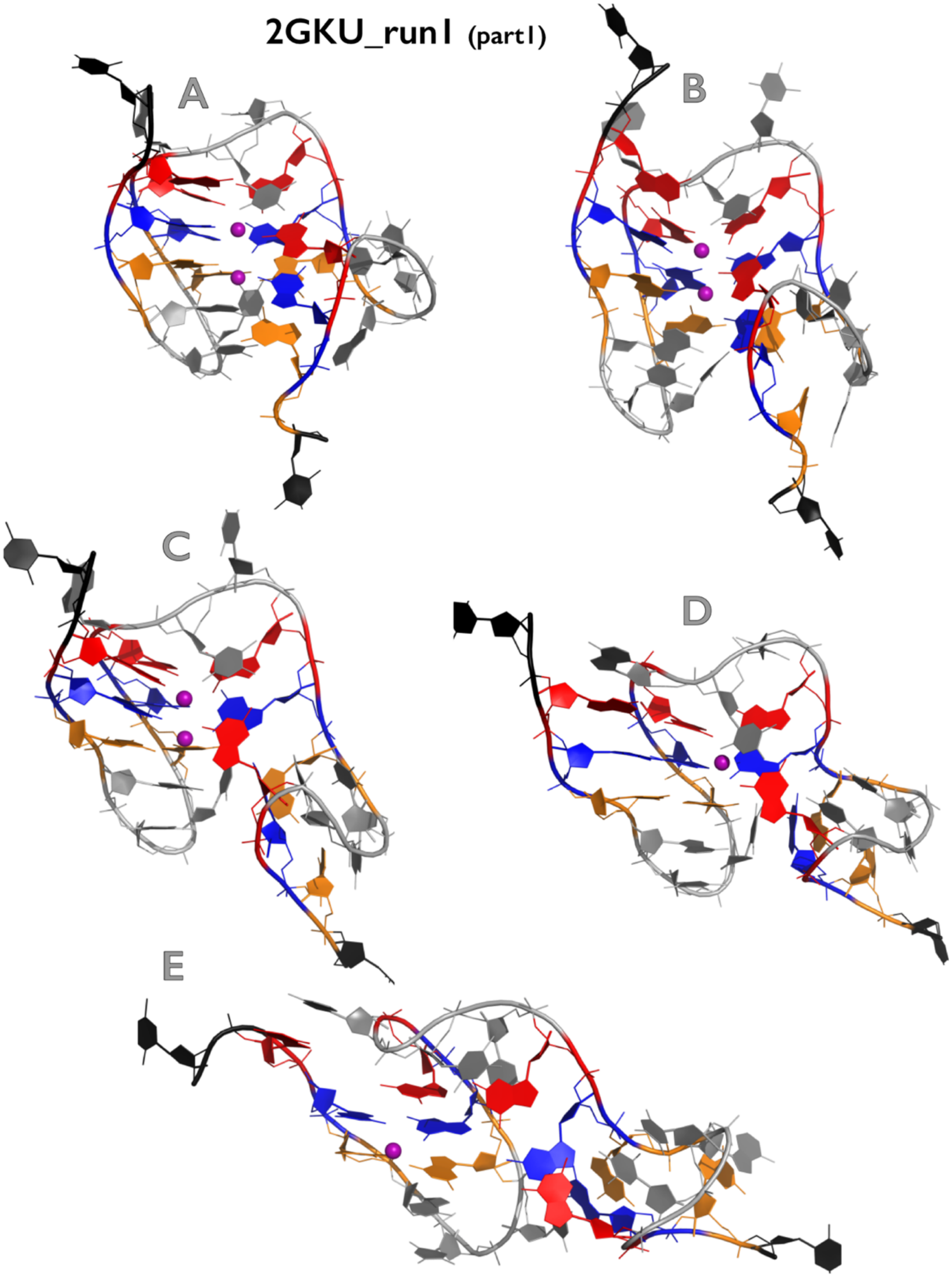

Figure continuing on the next page

2GKU_run1 (part2)

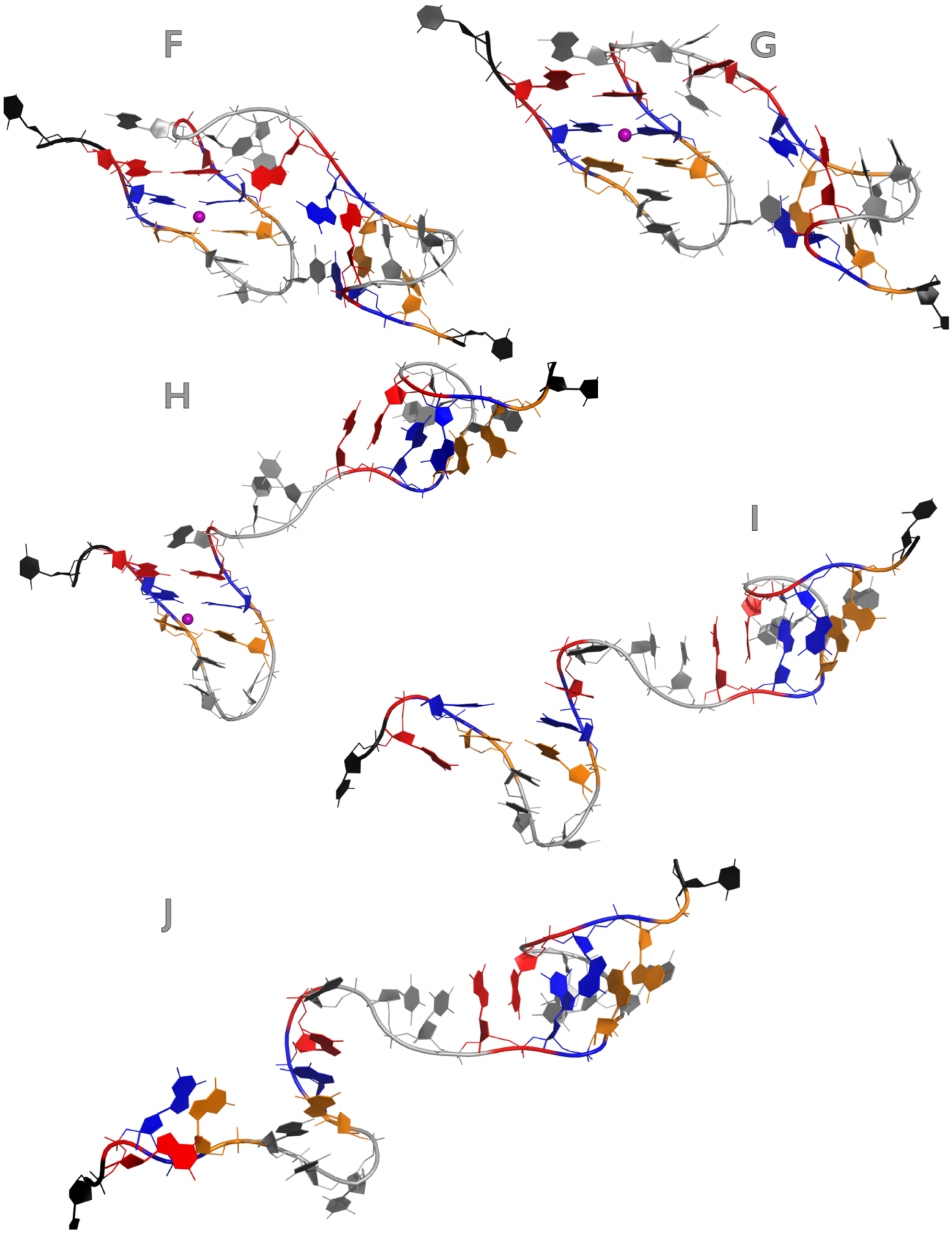

Figure S26B: Most important structural events during first independent *very slow pulling* simulation of 2GKU GQ system. See legend of Figure S1B for more details.

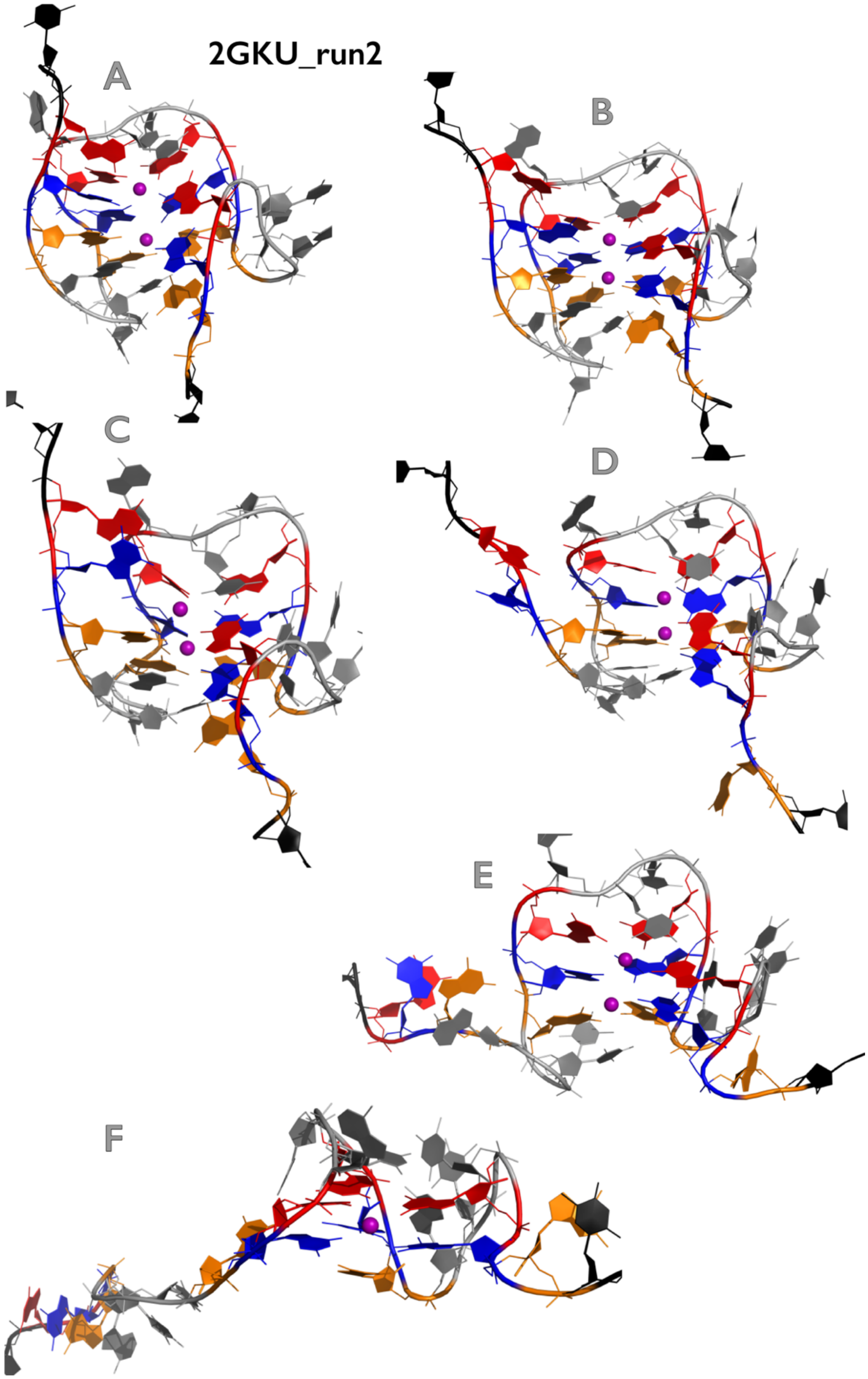

Figure S26C: Most important structural events during second independent *very slow pulling* simulation of 2GKU GQ system. See legend of Figure S1B for more details.

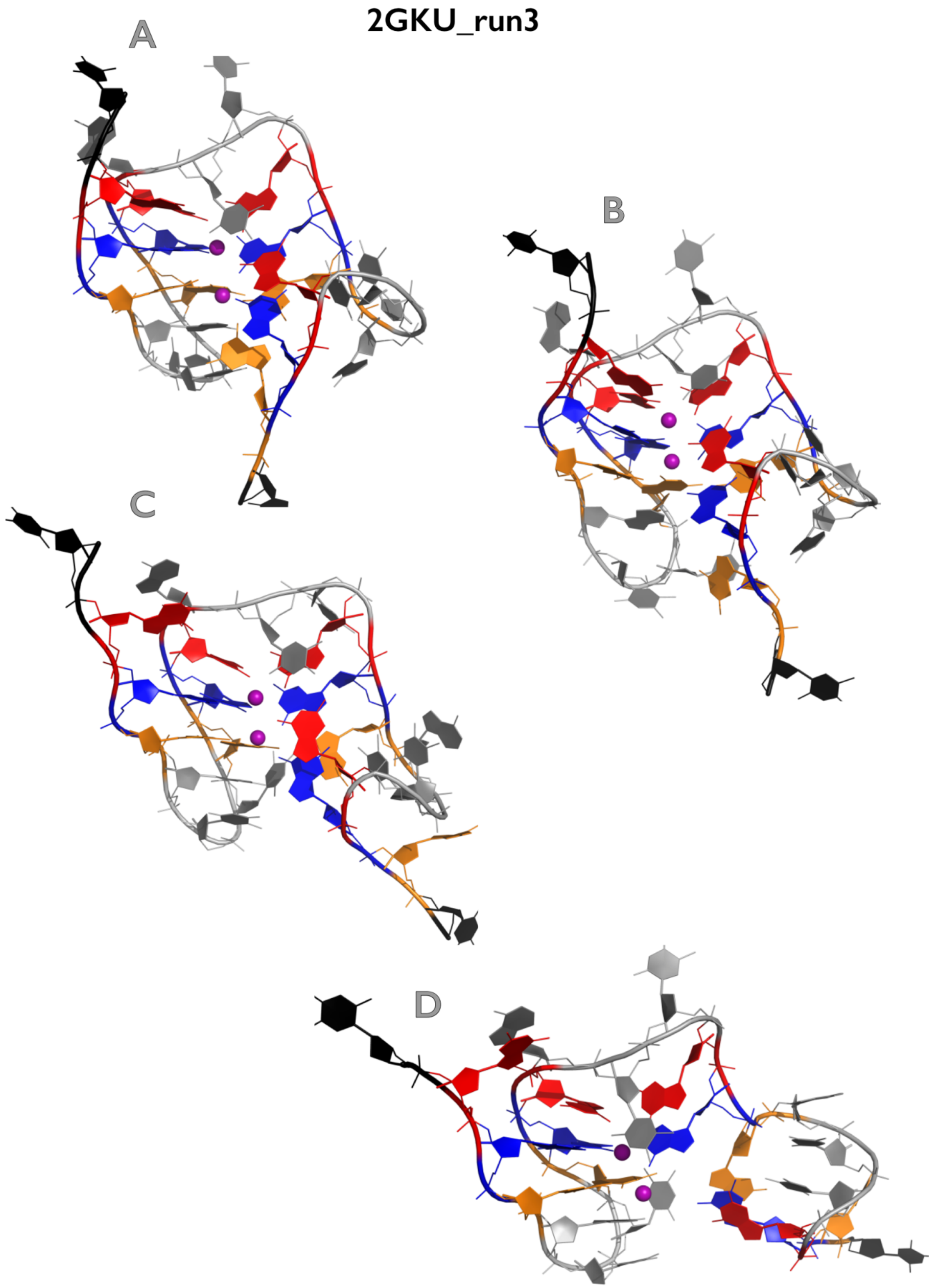

Figure S26D: Most important structural events during third independent *very slow pulling* simulation of 2GKU GQ system. See legend of Figure S1B for more details.

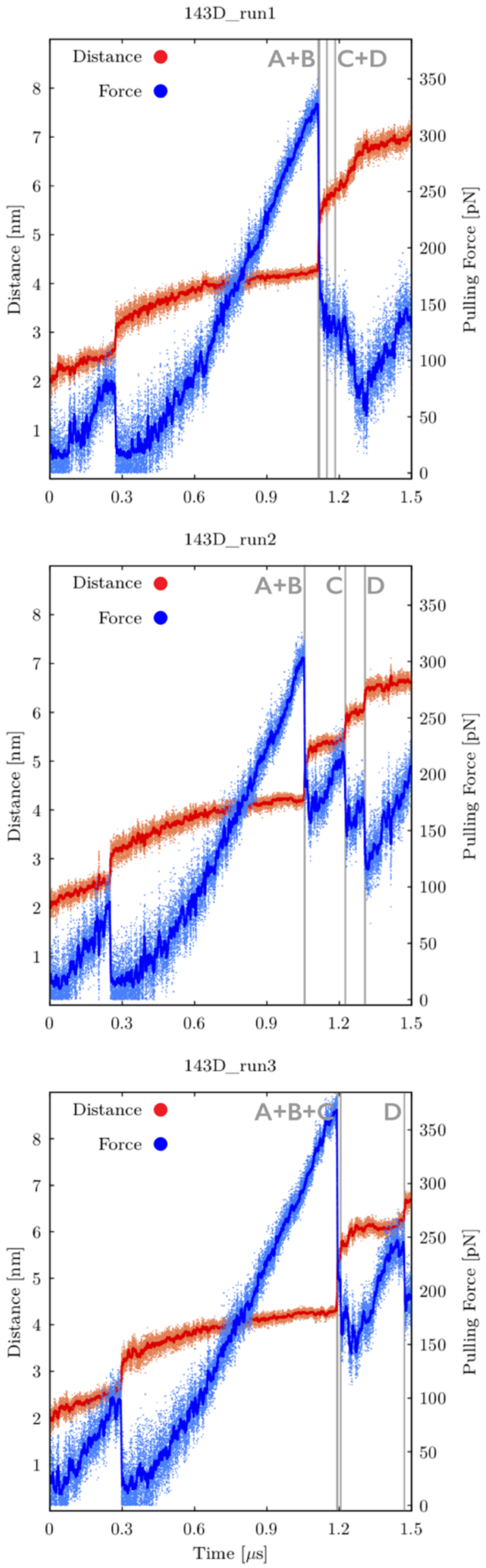

Figure S27A: Time evolution of distance between pulling centers and pulling force during three independent *very slow pulling* simulations of 143D GQ system (see legend of Figure S25A for

more details). See Figures S27B-S27D for inspection of structures corresponding to main structural events.

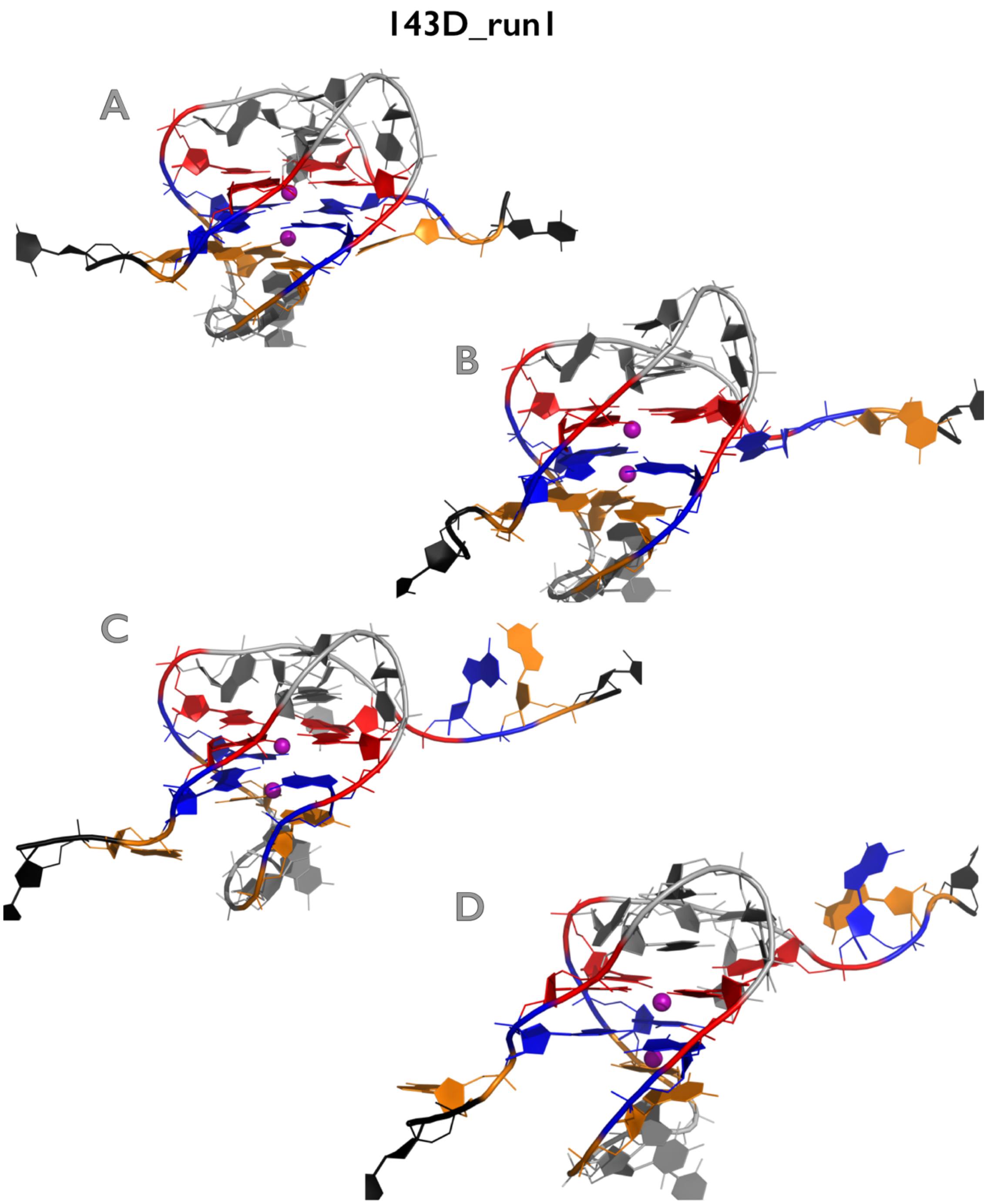

Figure S27B: Most important structural events during first independent *very slow pulling* simulation of 143D GQ system. See legend of Figure S1B for more details.

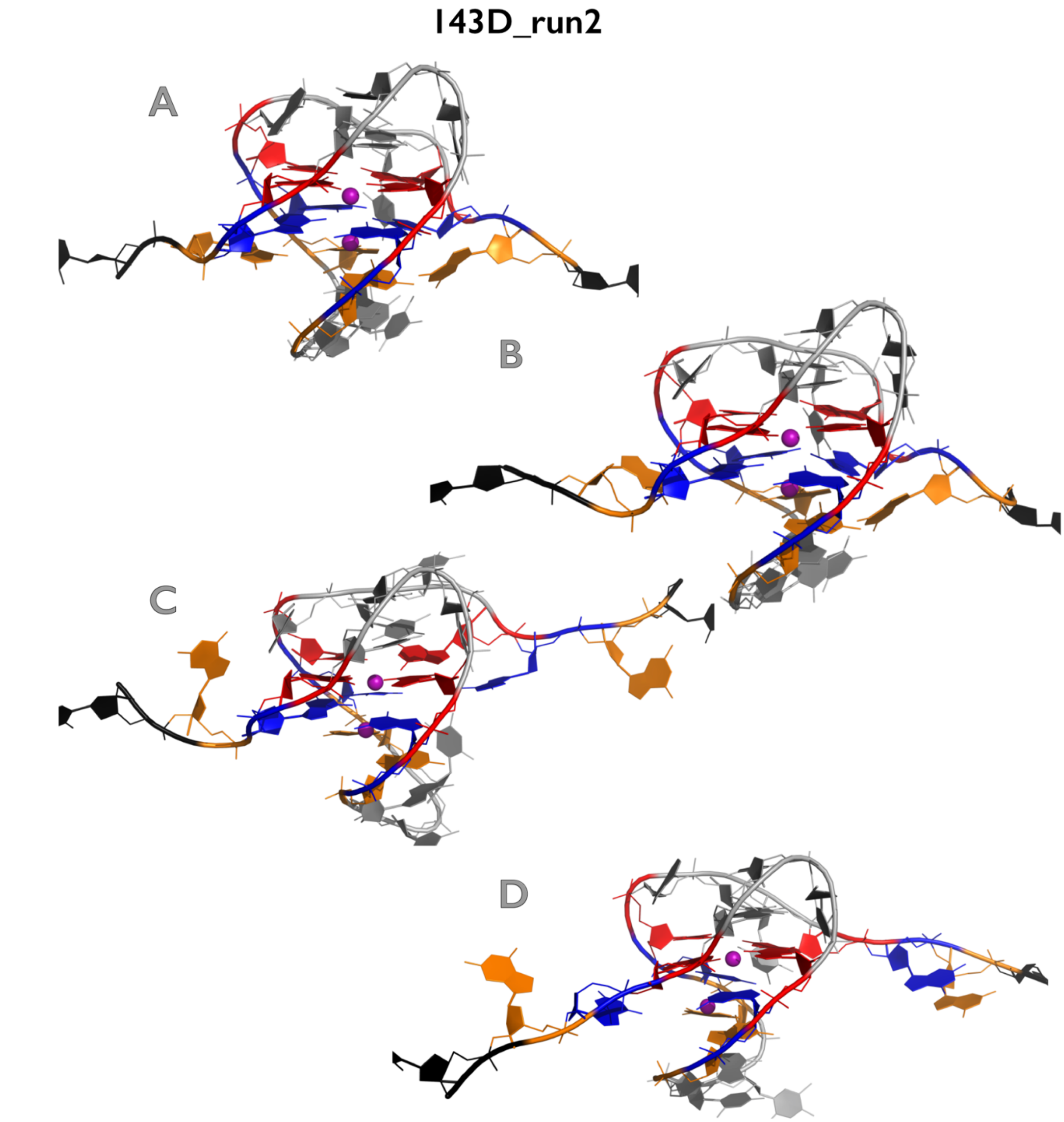

Figure S27C: Most important structural events during second independent *very slow pulling* simulation of 143D GQ system. See legend of Figure S1B for more details.

143D_run3

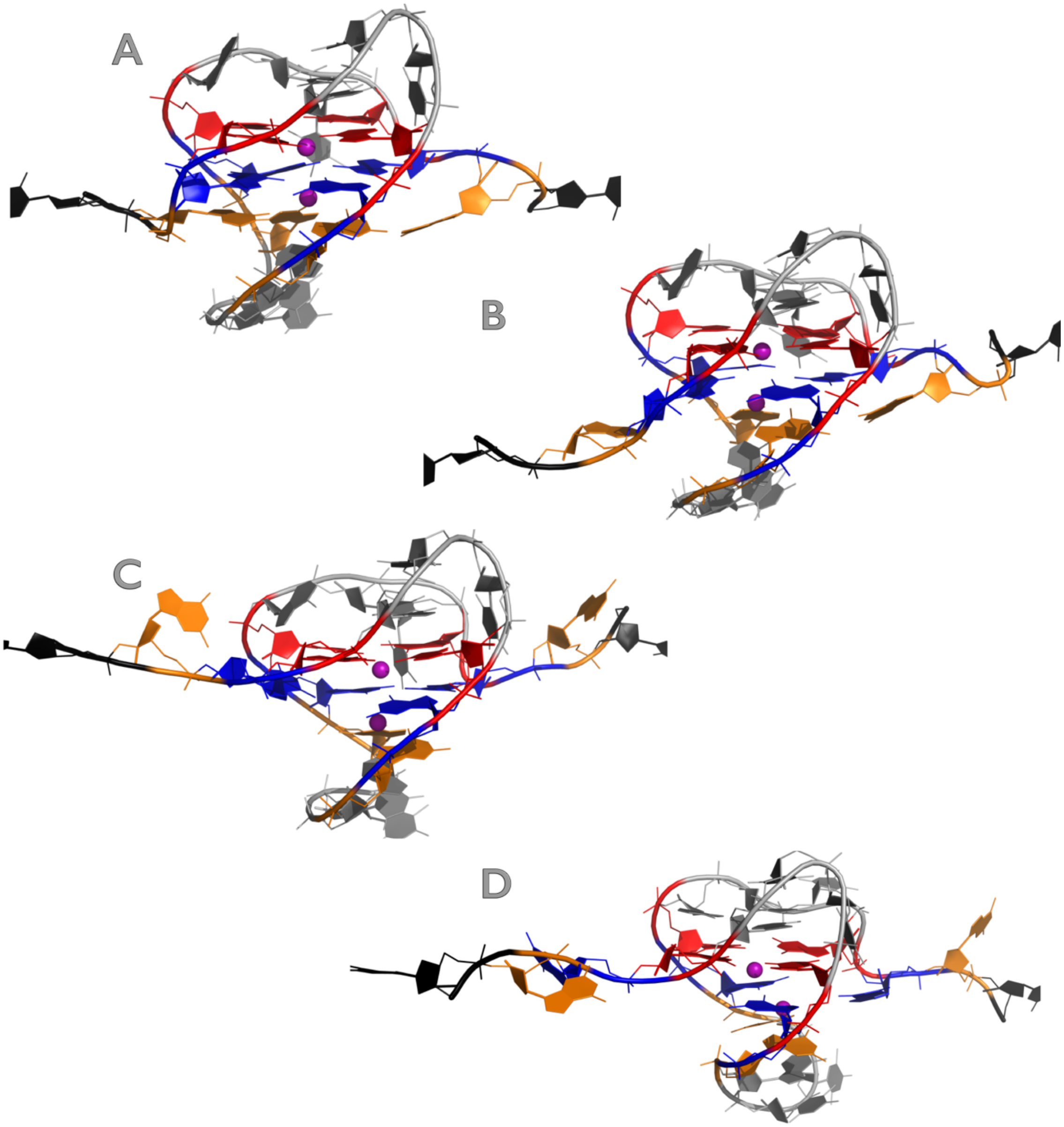

Figure S27D: Most important structural events during third independent *very slow pulling* simulation of 143D GQ system. See legend of Figure S1B for more details.

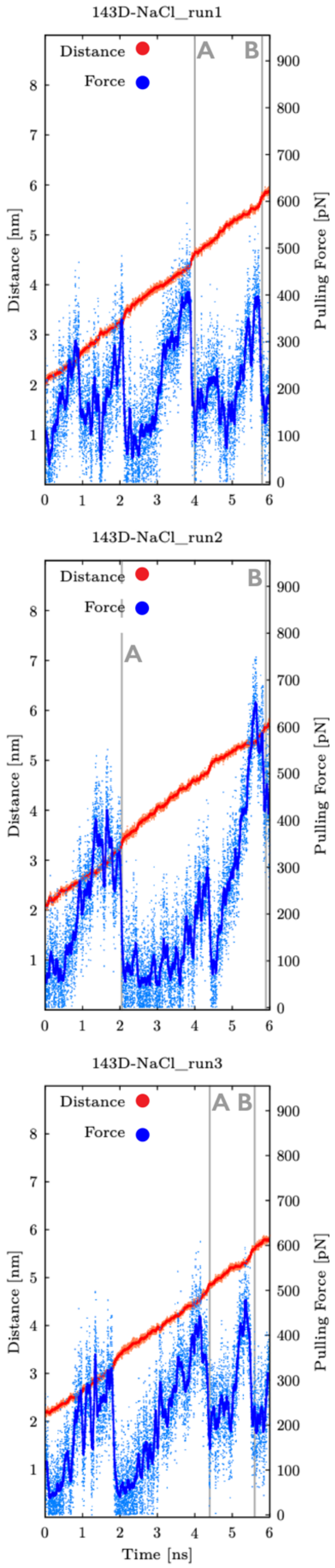

Figure S28A: Time evolution of distance between pulling centers and pulling force during three independent *fast pulling* simulations of 143D$_{NaCl}$ GQ system (see legend of Figure S1A for more details). See Figure S28B for inspection of structures corresponding to main structural events.

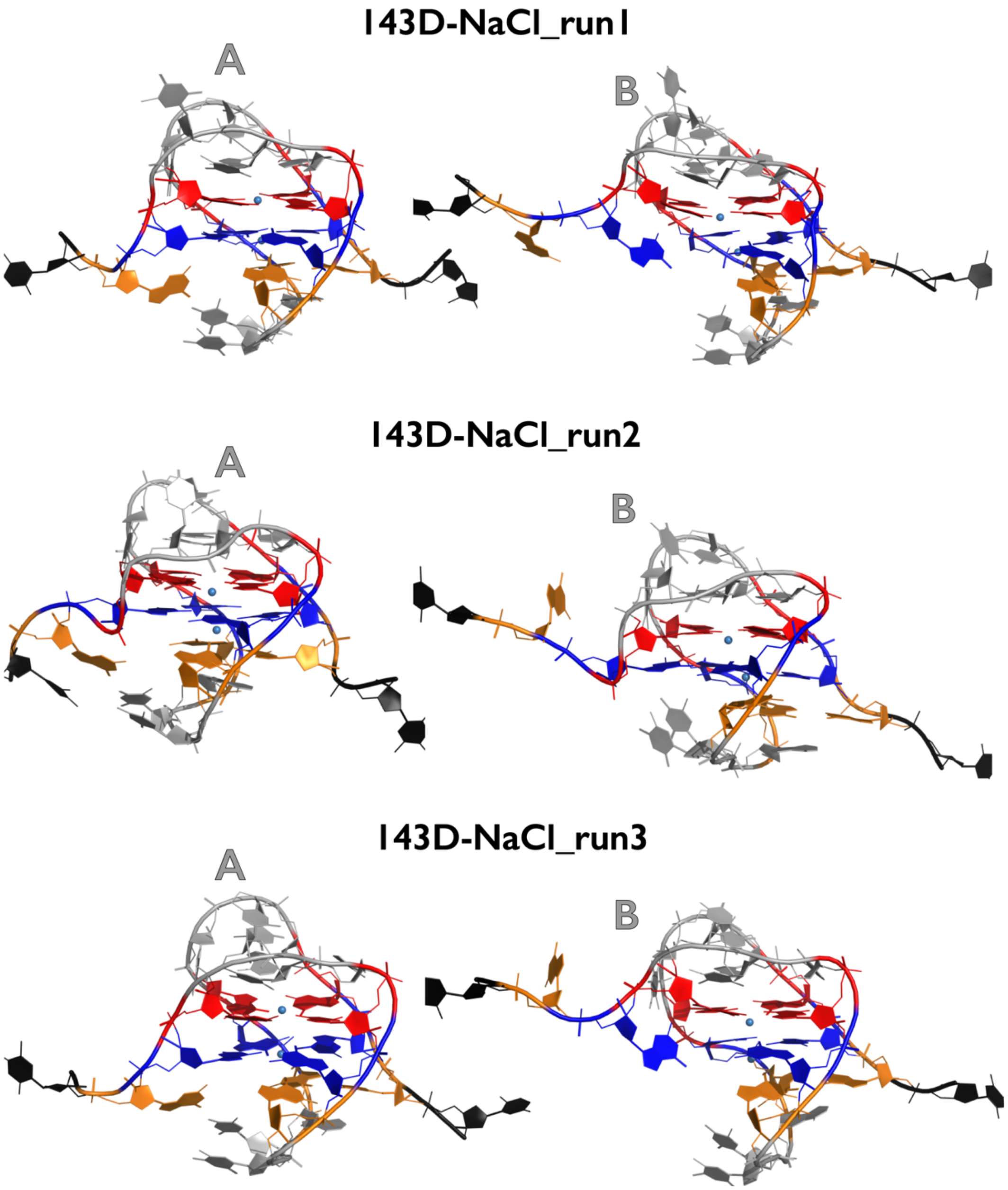

Figure S28B: Most important structural events during first, second and third independent *fast pulling* simulations of 143D$_{NaCl}$ GQ system. G residues from first (5'-end), second and third quartet are highlighted in orange, blue and red, respectively. Pulling centers, i.e., both terminal T residues, are shown in black. Remaining DNA residues are in gray and channel Na^+ ions are shown as purple spheres. Additional Na^+ ions, Cl^- ions, H-atoms and water molecules are not shown for clarity.

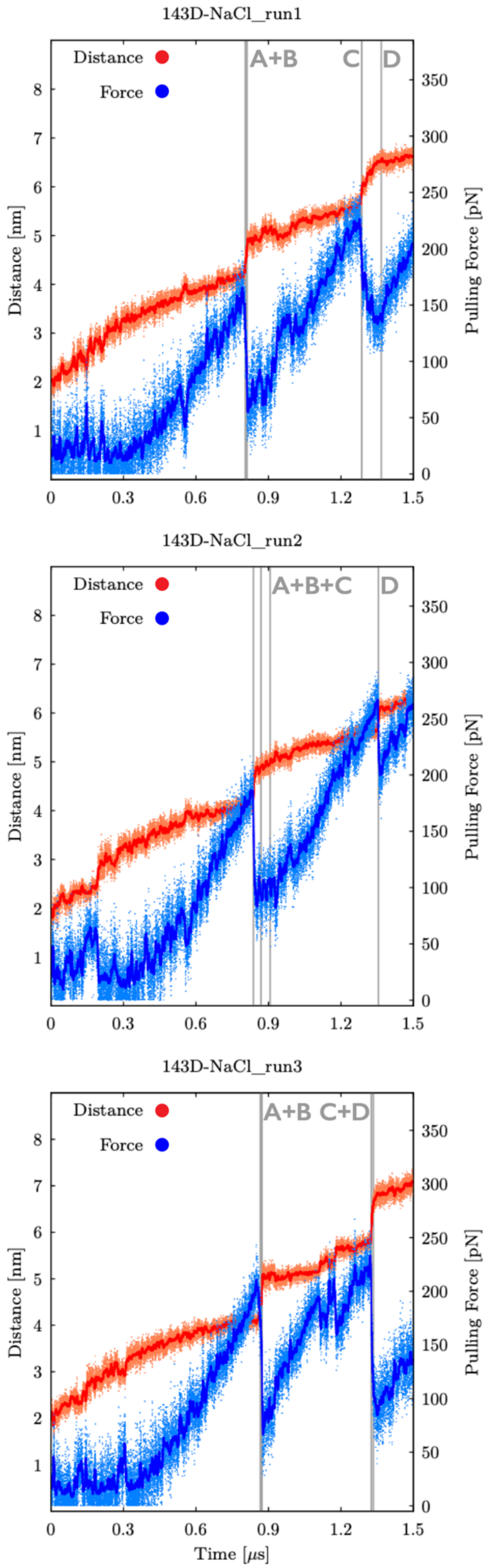

Figure S29A: Time evolution of distance between pulling centers and pulling force during three independent *very slow pulling* simulations of 143D$_{NaCl}$ GQ system (see legend of Figure S25A for

more details). See Figures S29B-S29D for inspection of structures corresponding to main structural events.

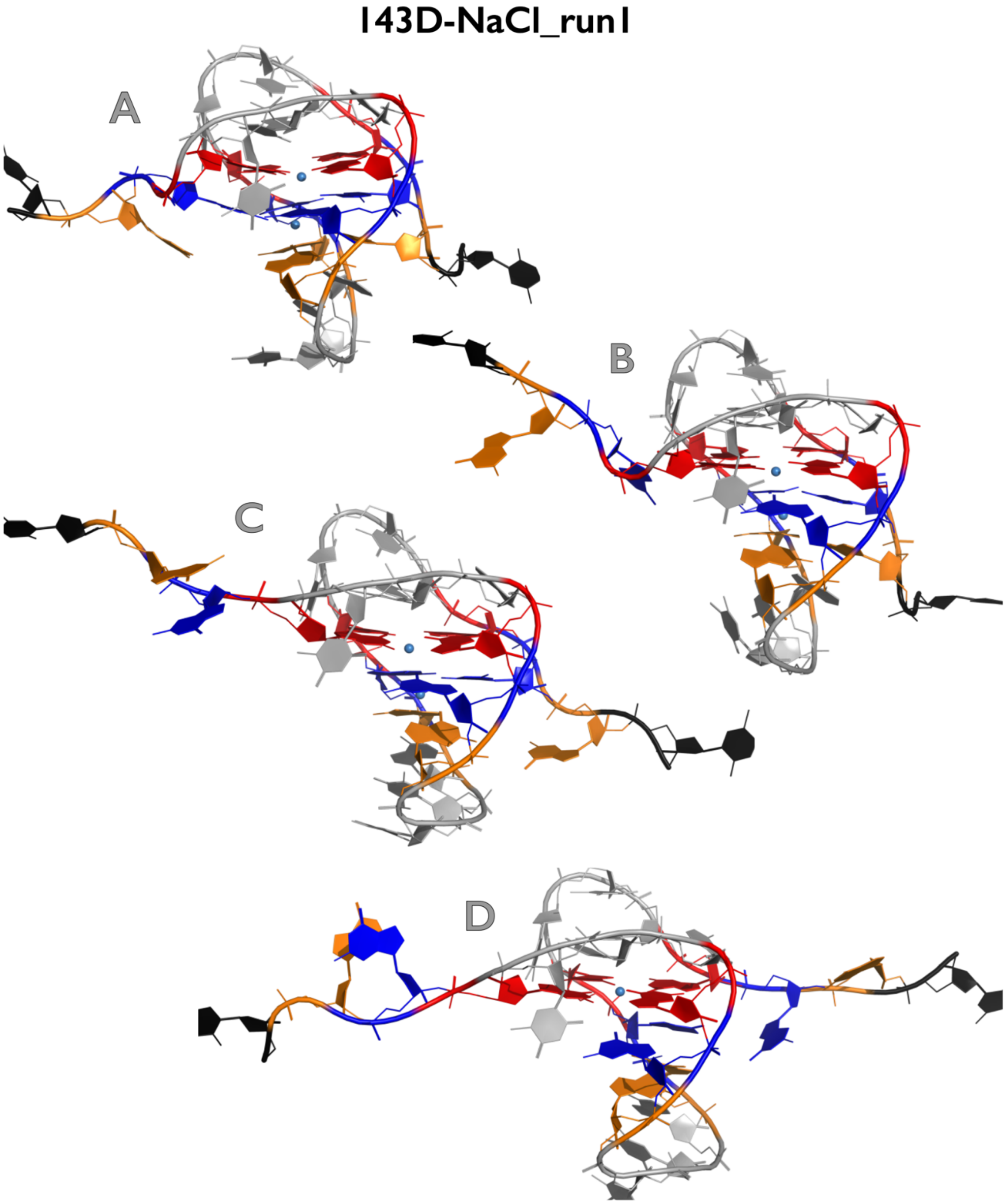

Figure S29B: Most important structural events during first independent *very slow pulling* simulation of $143D_{NaCl}$ GQ system. See legend of Figure S28B for more details.

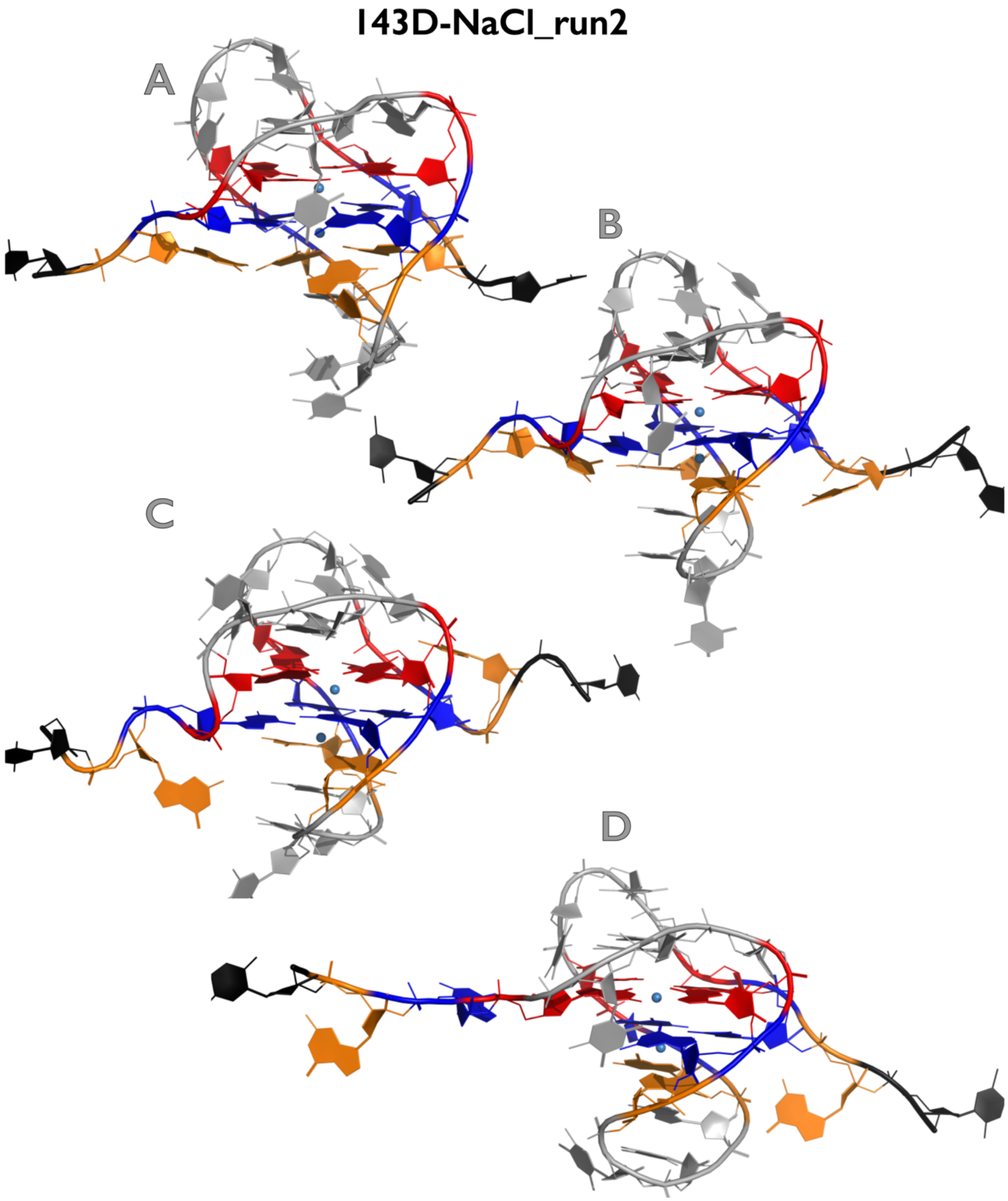

Figure S29C: Most important structural events during second independent *very slow pulling* simulation of 143D$_{NaCl}$ GQ system. See legend of Figure S28B for more details.

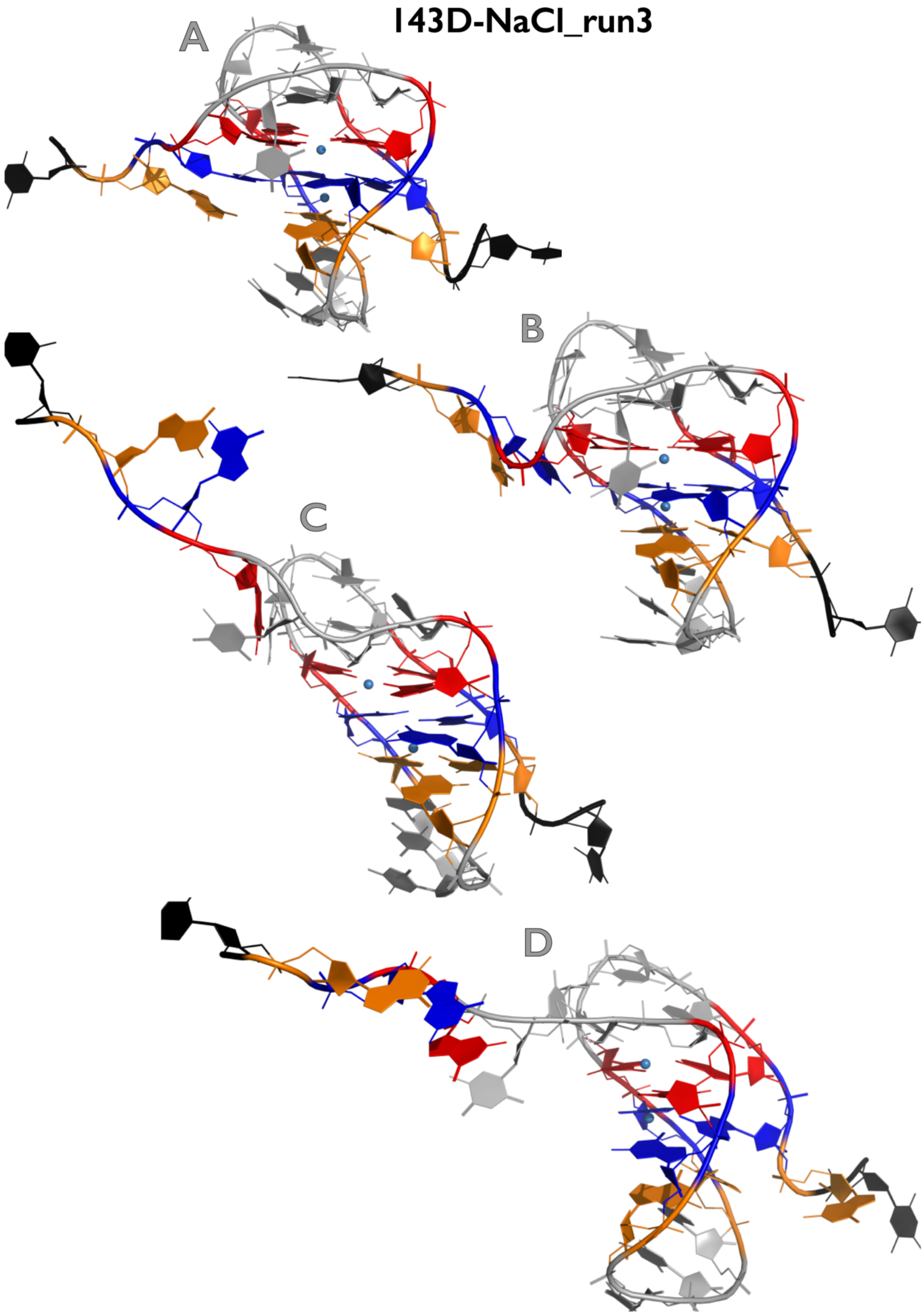

Figure S29D: Most important structural events during third independent *very slow pulling* simulation of $143D_{NaCl}$ GQ system. See legend of Figure S28B for more details.

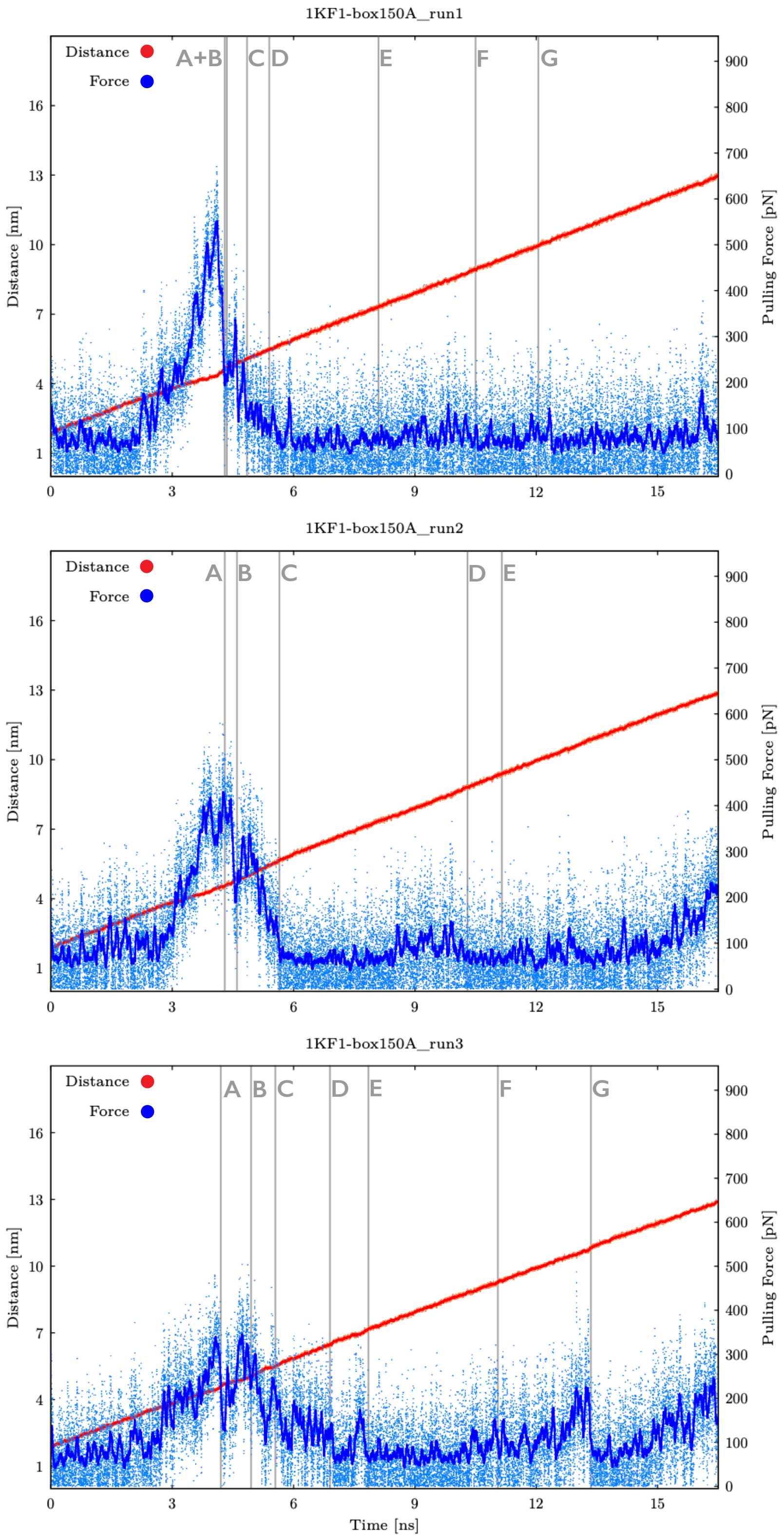

Figure S30A: Time evolution of distance between pulling centers and pulling force during three independent *fast pulling* simulations of 1KF1$_{box150Å}$ GQ system (see legend of Figure S1A for more details). See Figures S30B-S30D for inspection of structures corresponding to main structural events.

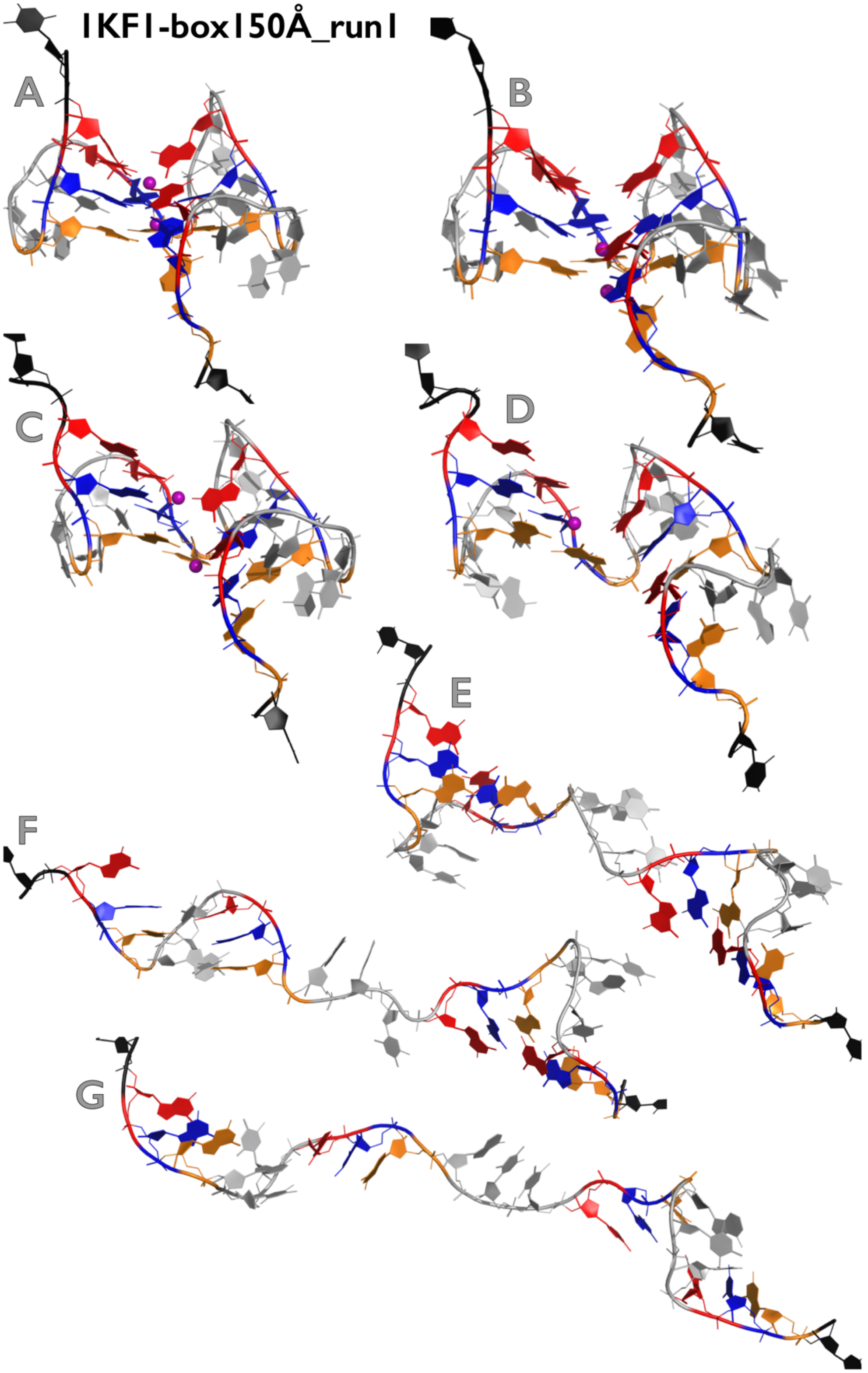

Figure S30B: Most important structural events during first independent *fast pulling* simulation of $1KF1_{box150Å}$ GQ system. See legend of Figure S1B for more details.

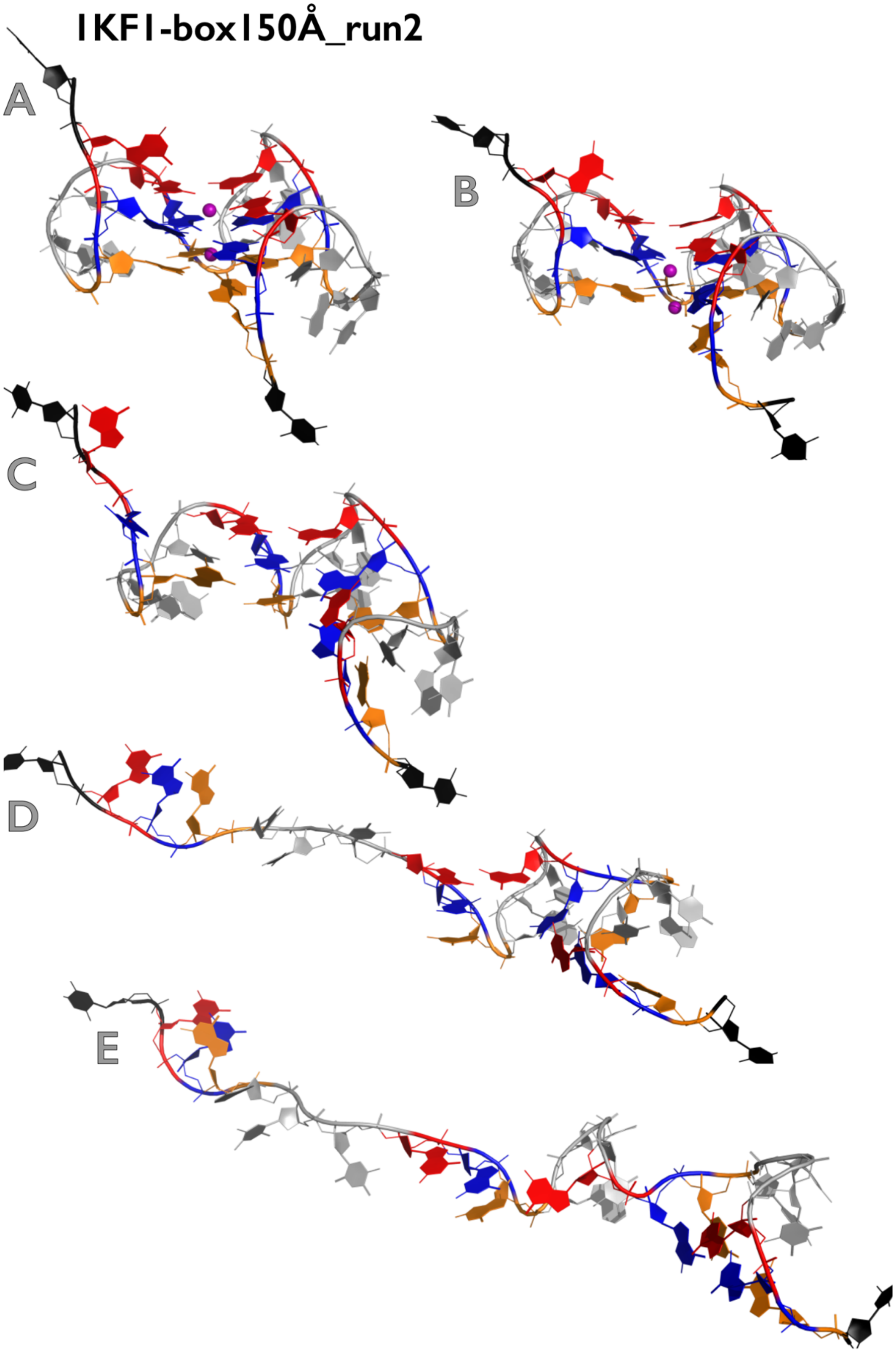

Figure S30C: Most important structural events during second independent *fast pulling* simulation of 1KF1$_{box150Å}$ GQ system. See legend of Figure S1B for more details.

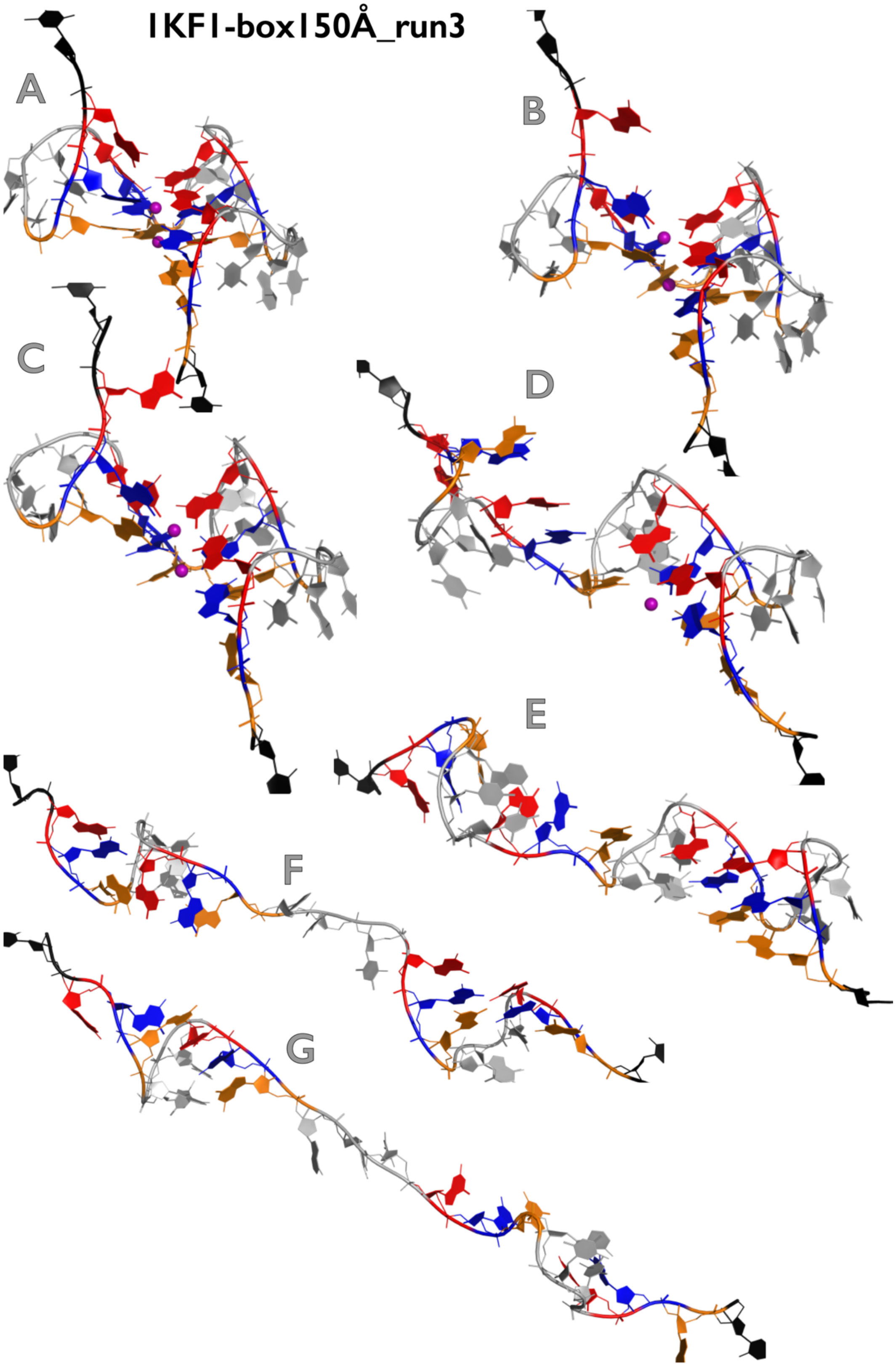

Figure S30D: Most important structural events during third independent *fast pulling* simulation of 1KF1$_{box150Å}$ GQ system. See legend of Figure S1B for more details.

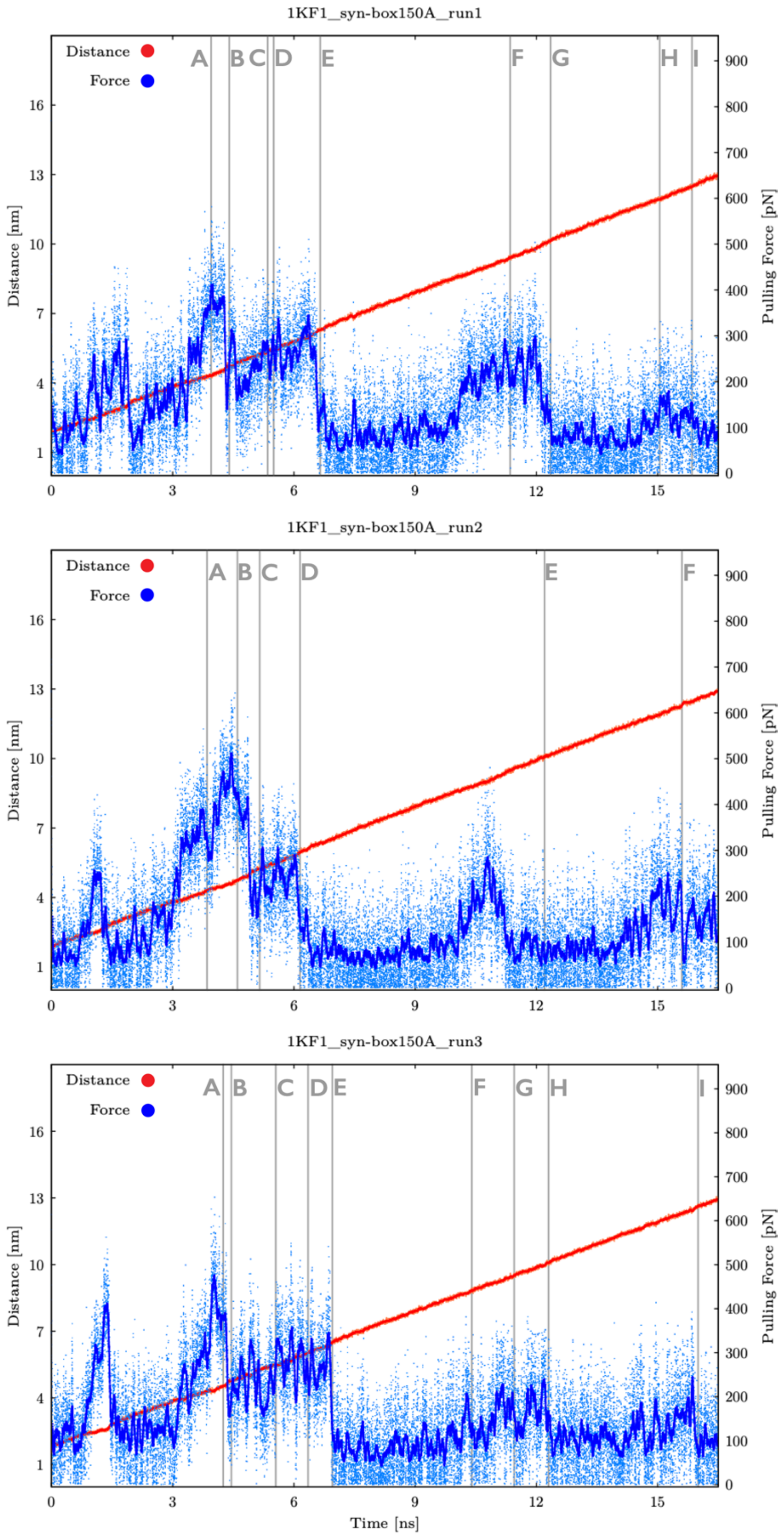

Figure S31A: Time evolution of distance between pulling centers and pulling force during three independent *fast pulling* simulations of 1KF1$_{\text{syn-box150Å}}$ GQ system (see legend of Figure S1A for more details). See Figures S31B-S31D for inspection of structures corresponding to main structural events.

1KF1_syn-box150Å_run1 (part 1)

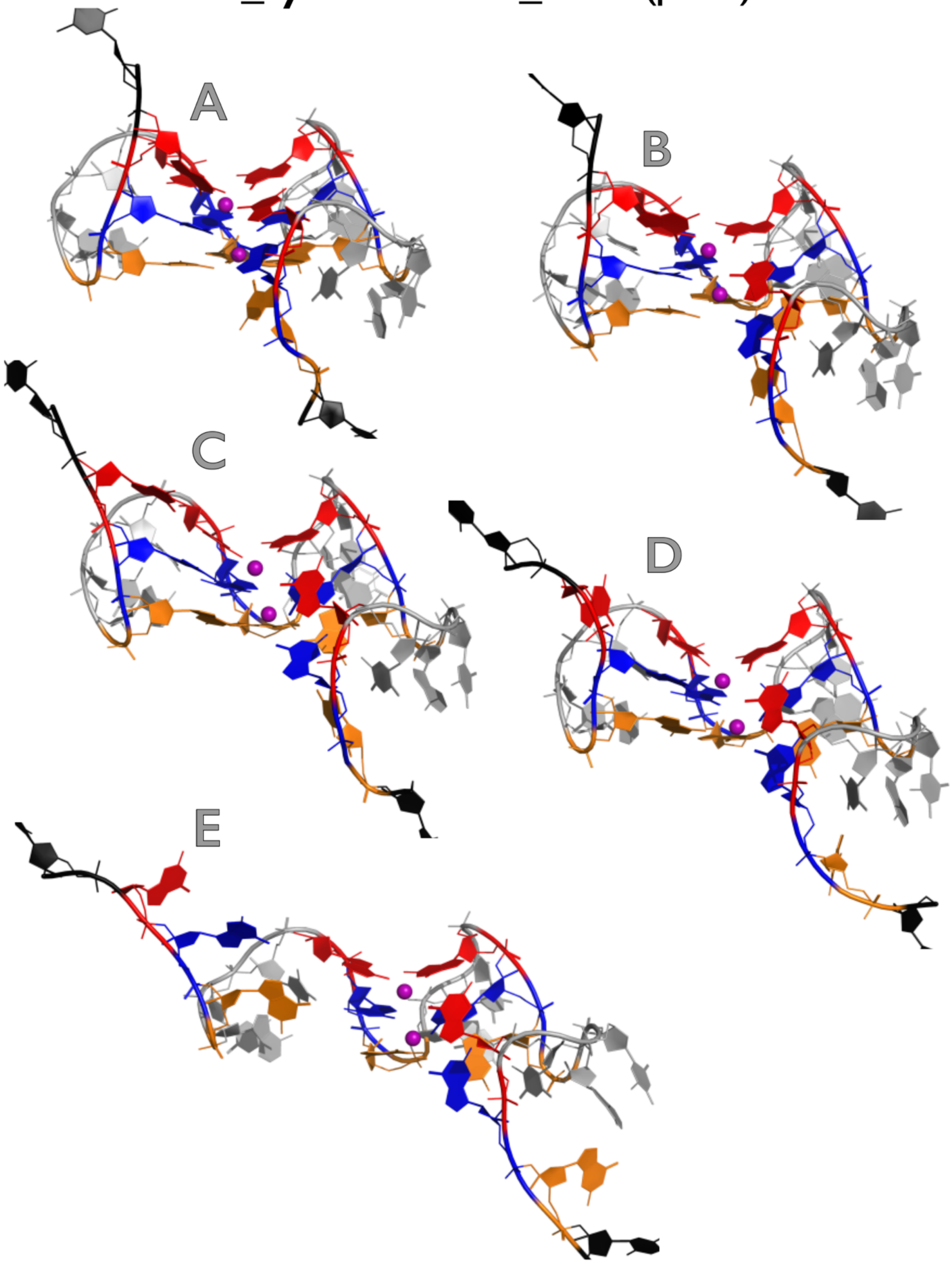

Figure continuing on the next page

1KF1_syn-box150Å_run1 (part 2)

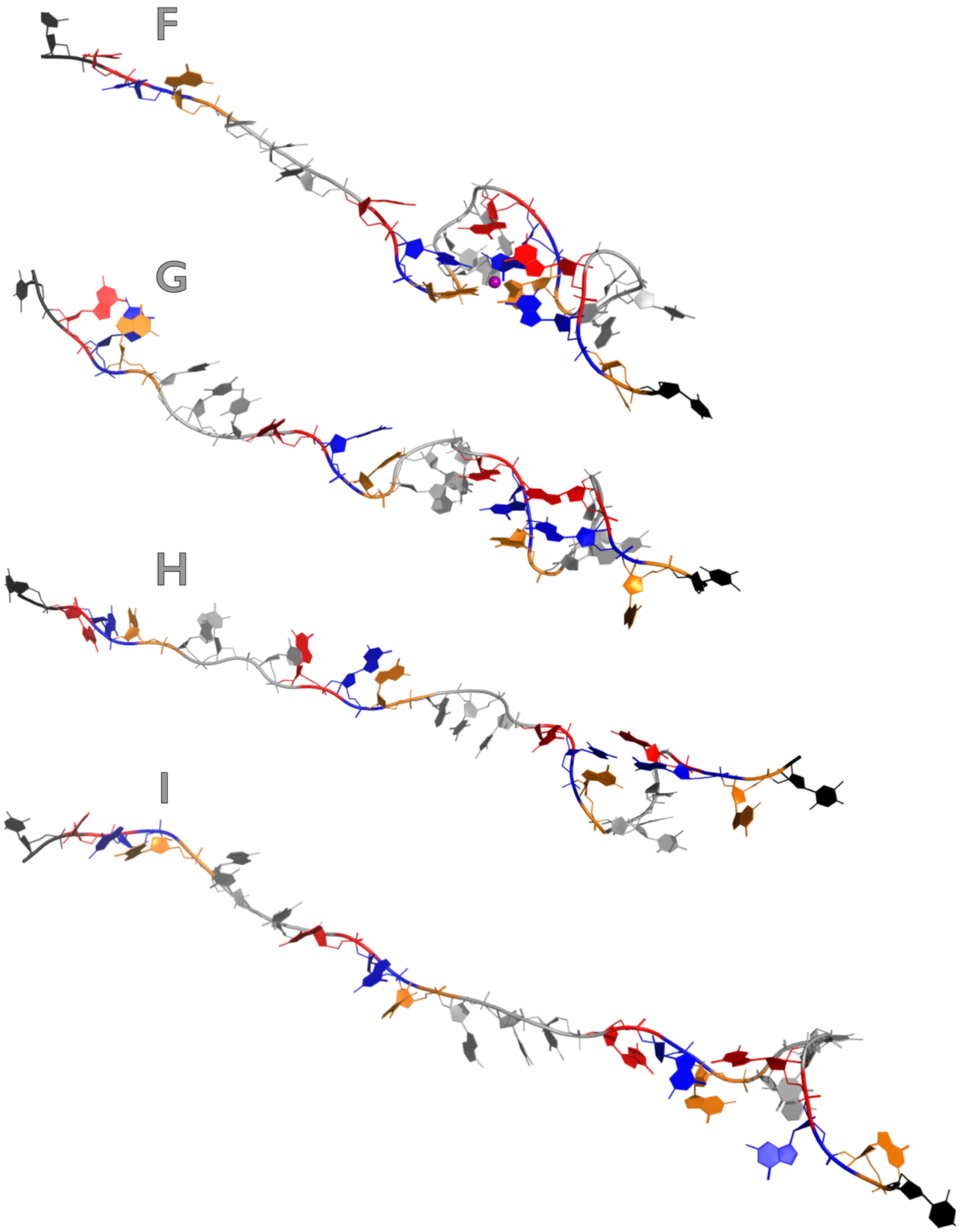

Figure S31B: Most important structural events during first independent *fast pulling* simulation of 1KF1$_{syn\text{-}box150Å}$ GQ system. See legend of Figure S1B for more details.

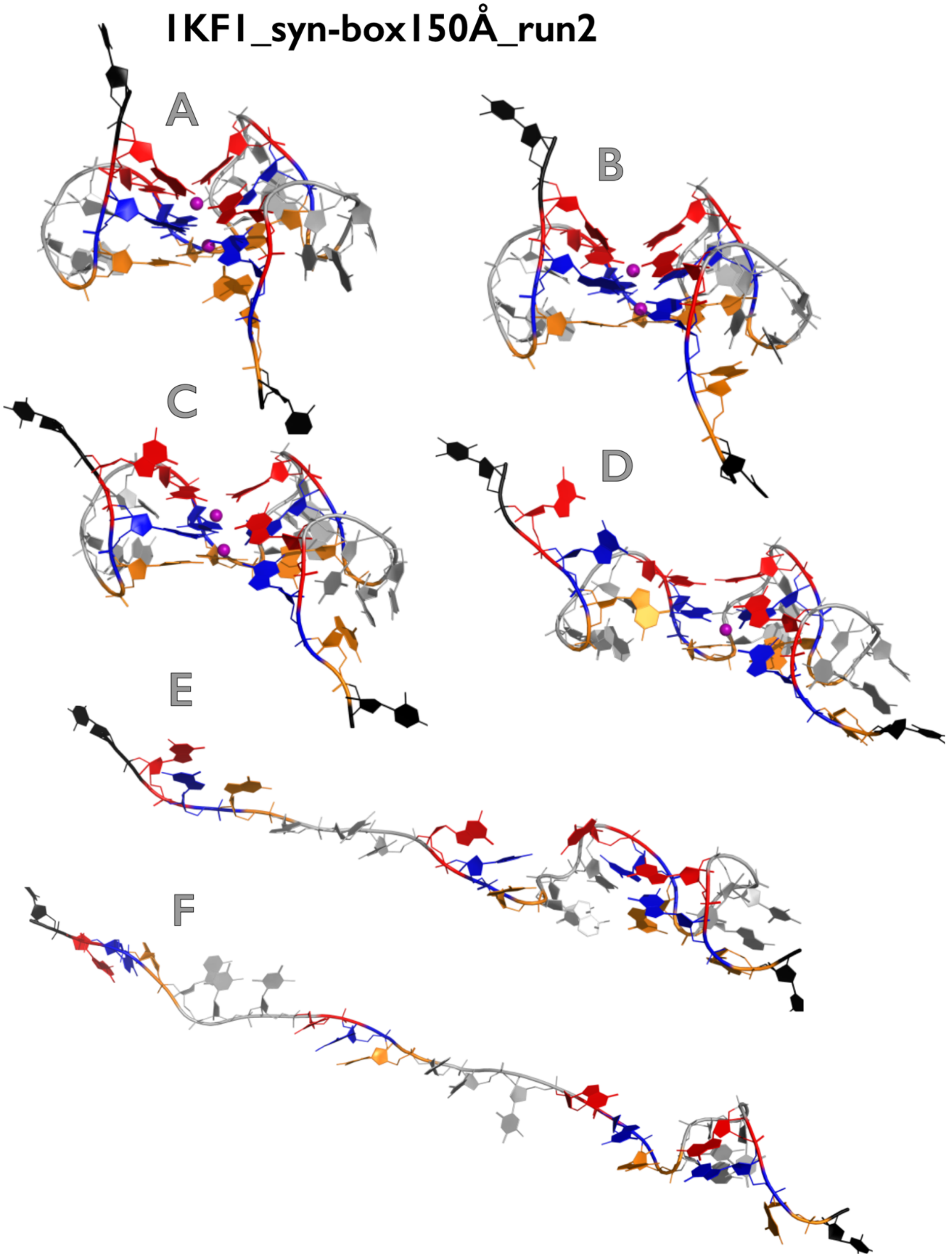

Figure S31C: Most important structural events during second independent *fast pulling* simulation of $1KF1_{syn\text{-}box150Å}$ GQ system. See legend of Figure S1B for more details.

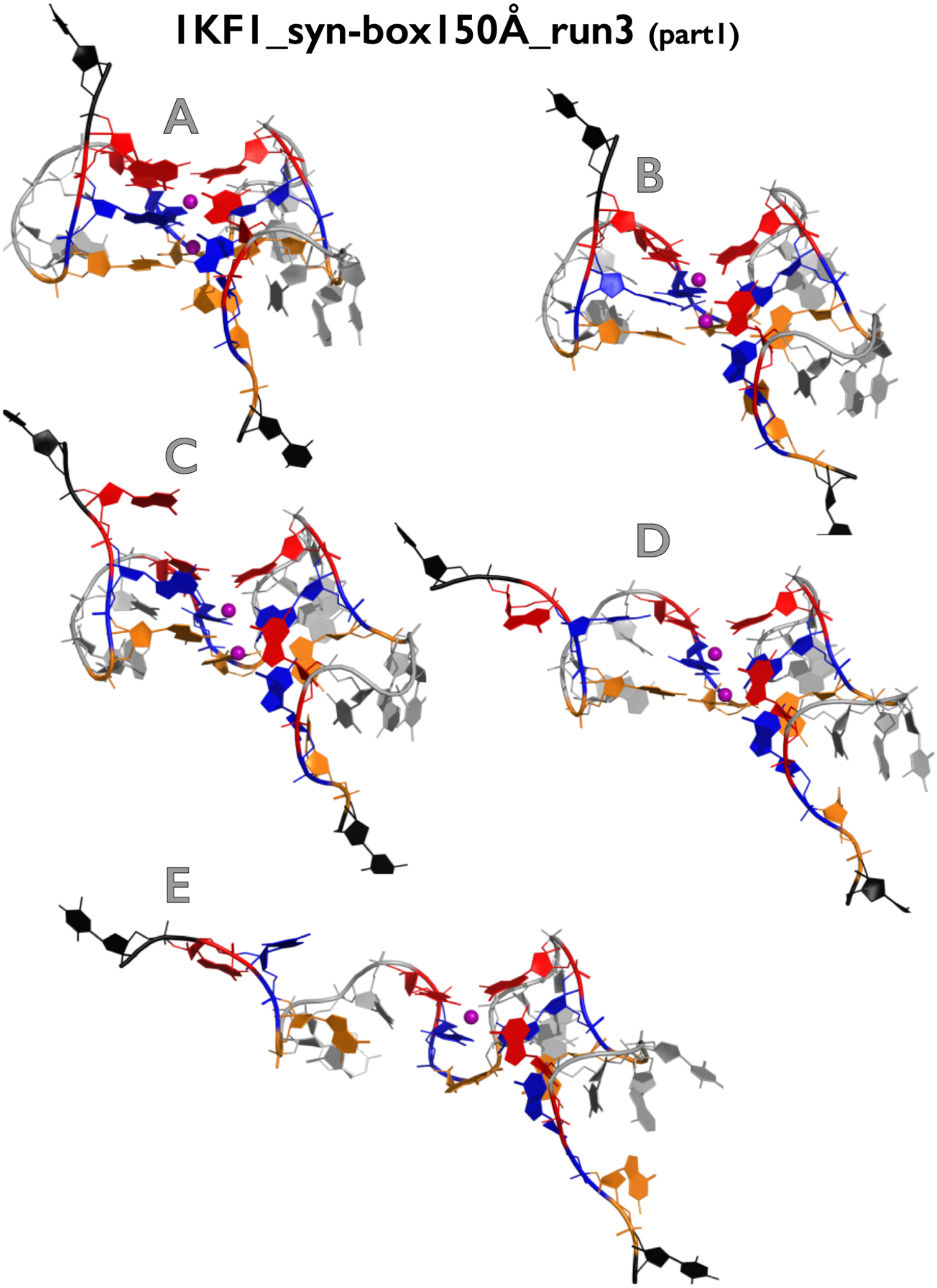

Figure continuing on the next page

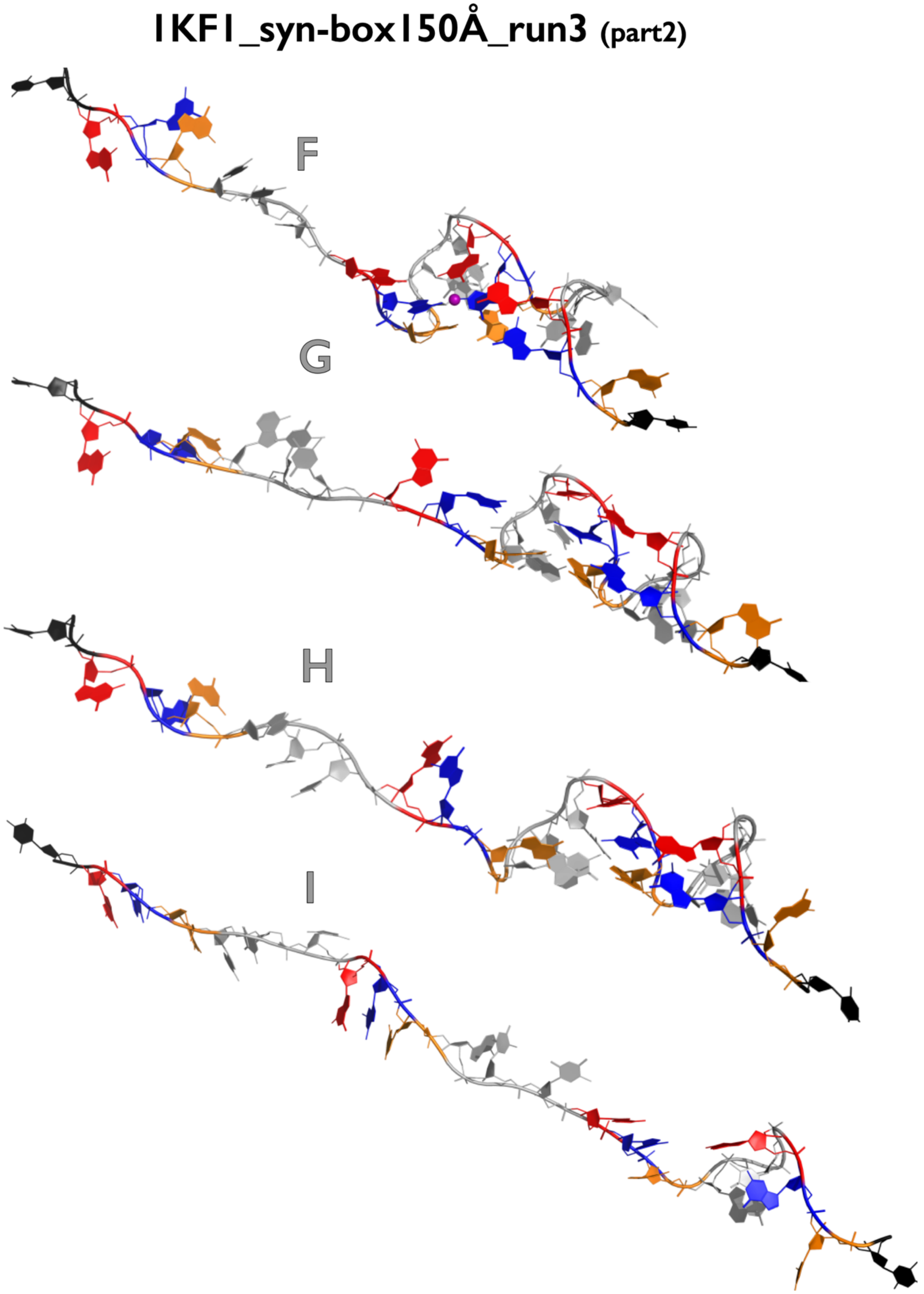

Figure S31D: Most important structural events during third independent *fast pulling* simulation of 1KF1$_{\text{syn-box150Å}}$ GQ system. See legend of Figure S1B for more details.

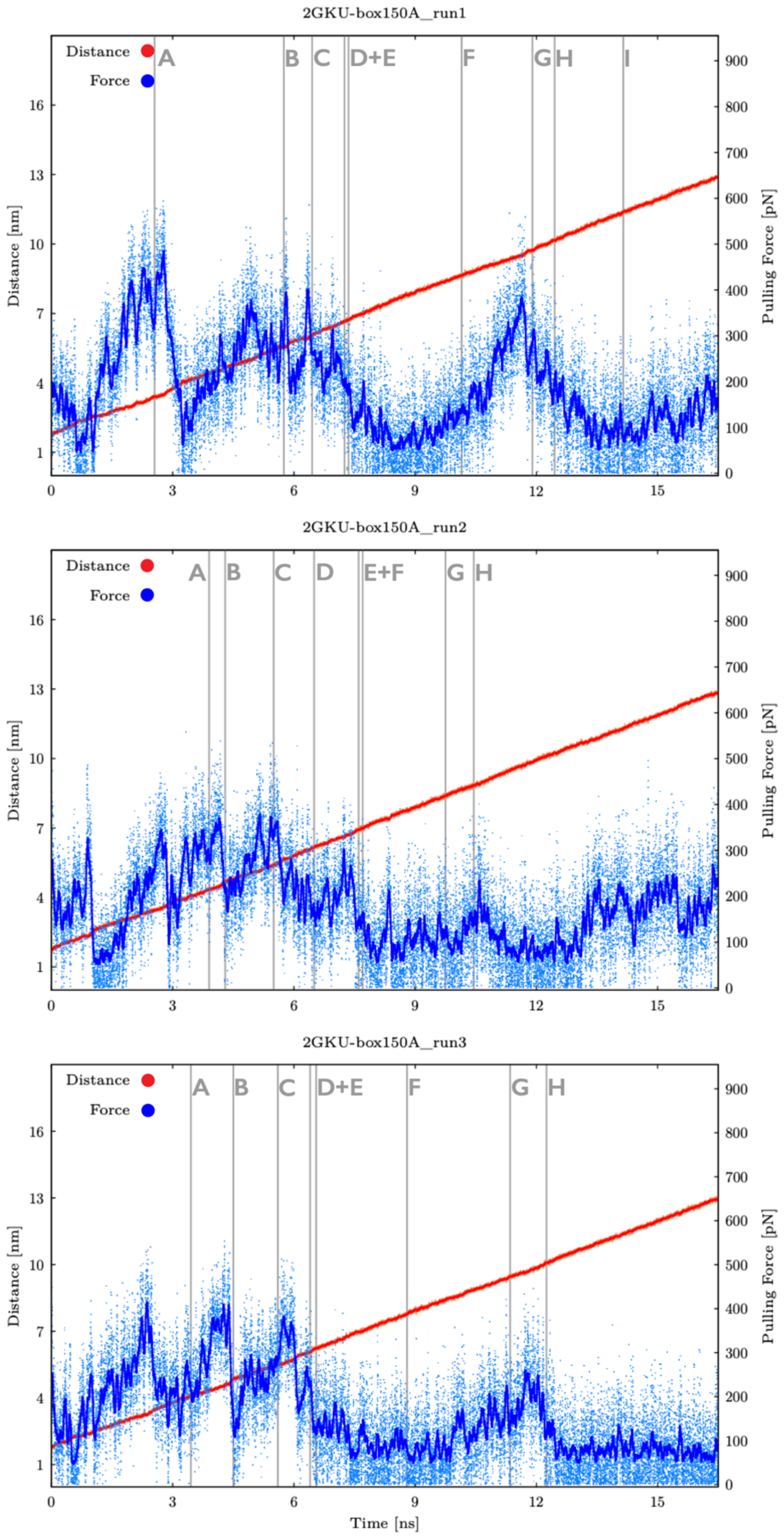

Figure S32A: Time evolution of distance between pulling centers and pulling force during three independent *fast pulling* simulations of 2GKU$_{box150Å}$ GQ system (see legend of Figure S1A for more details). See Figures S32B-S32D for inspection of structures corresponding to main structural events.

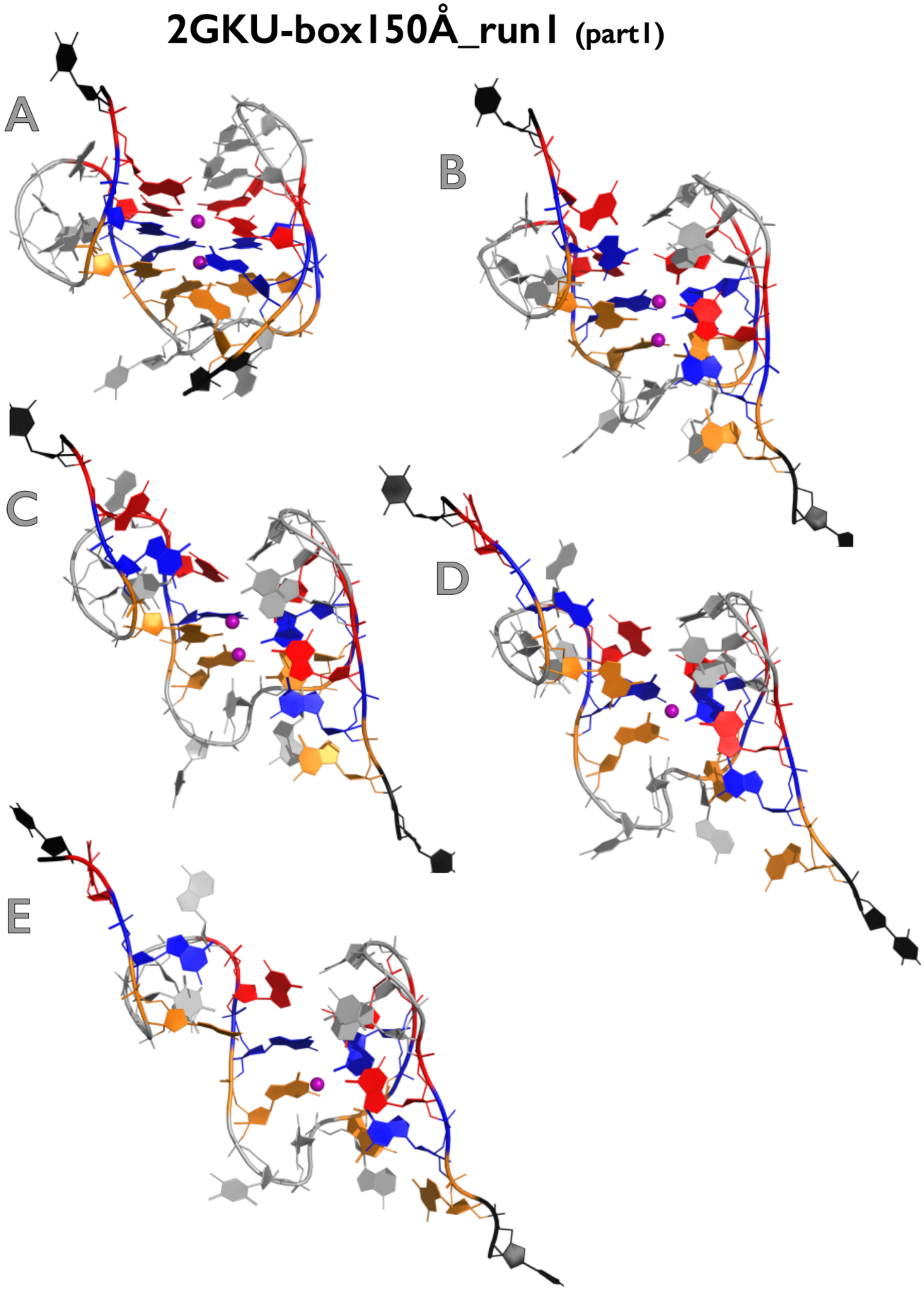

Figure continuing on the next page

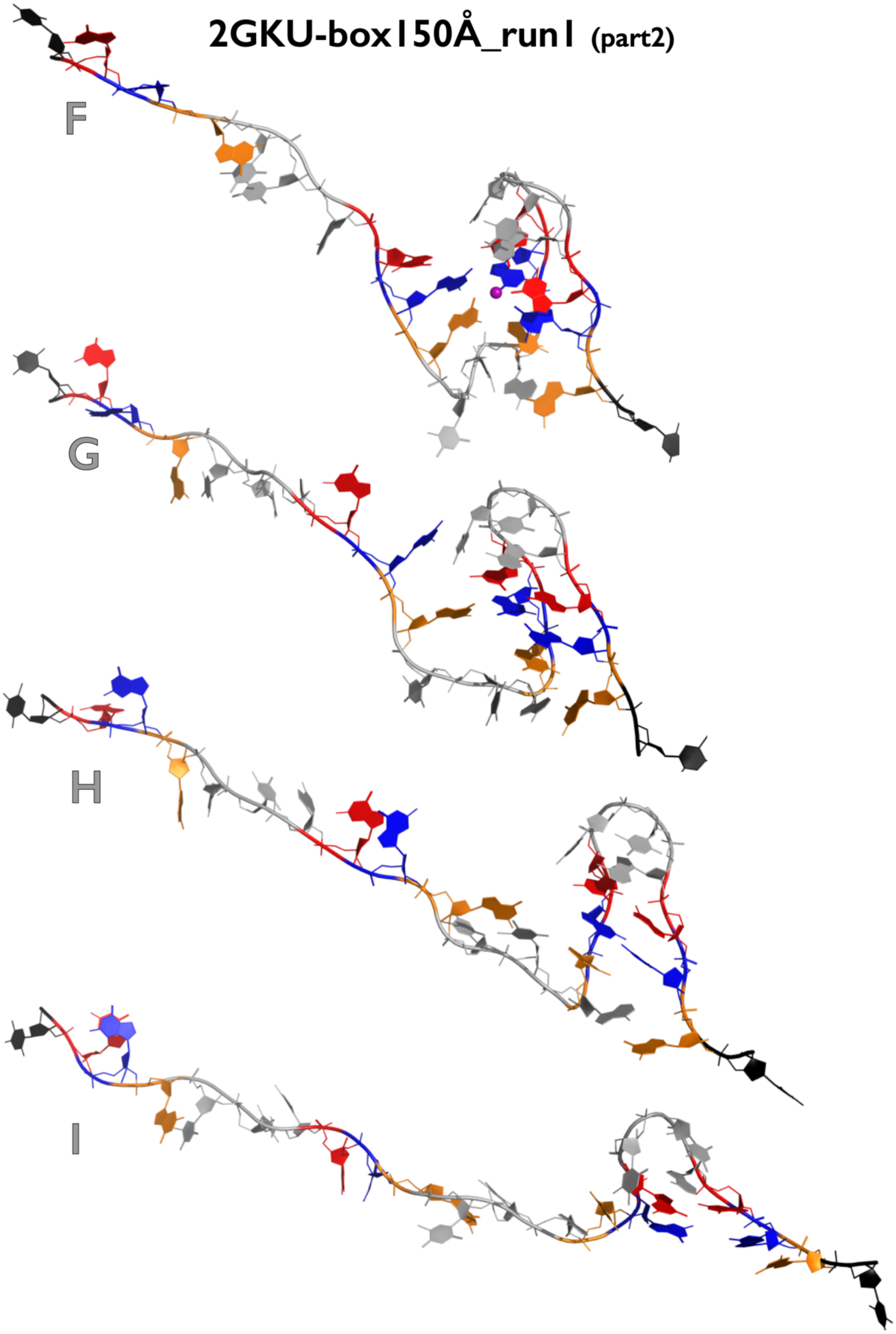

Figure S32B: Most important structural events during first independent *fast pulling* simulation of $2GKU_{box150Å}$ GQ system. See legend of Figure S1B for more details.

2GKU-box150Å_run2 (part1)

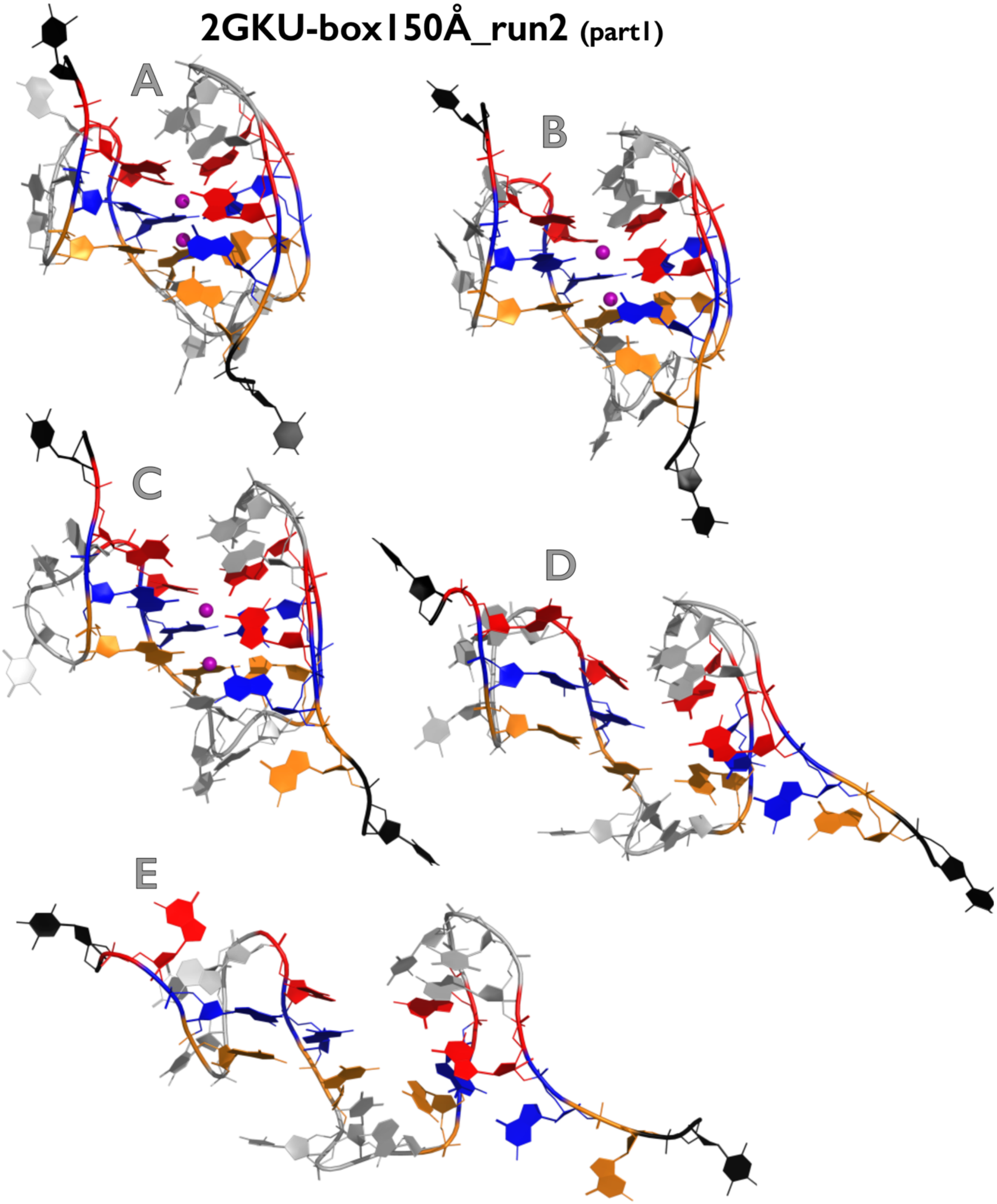

Figure continuing on the next page

2GKU-box150Å_run2 (part2)

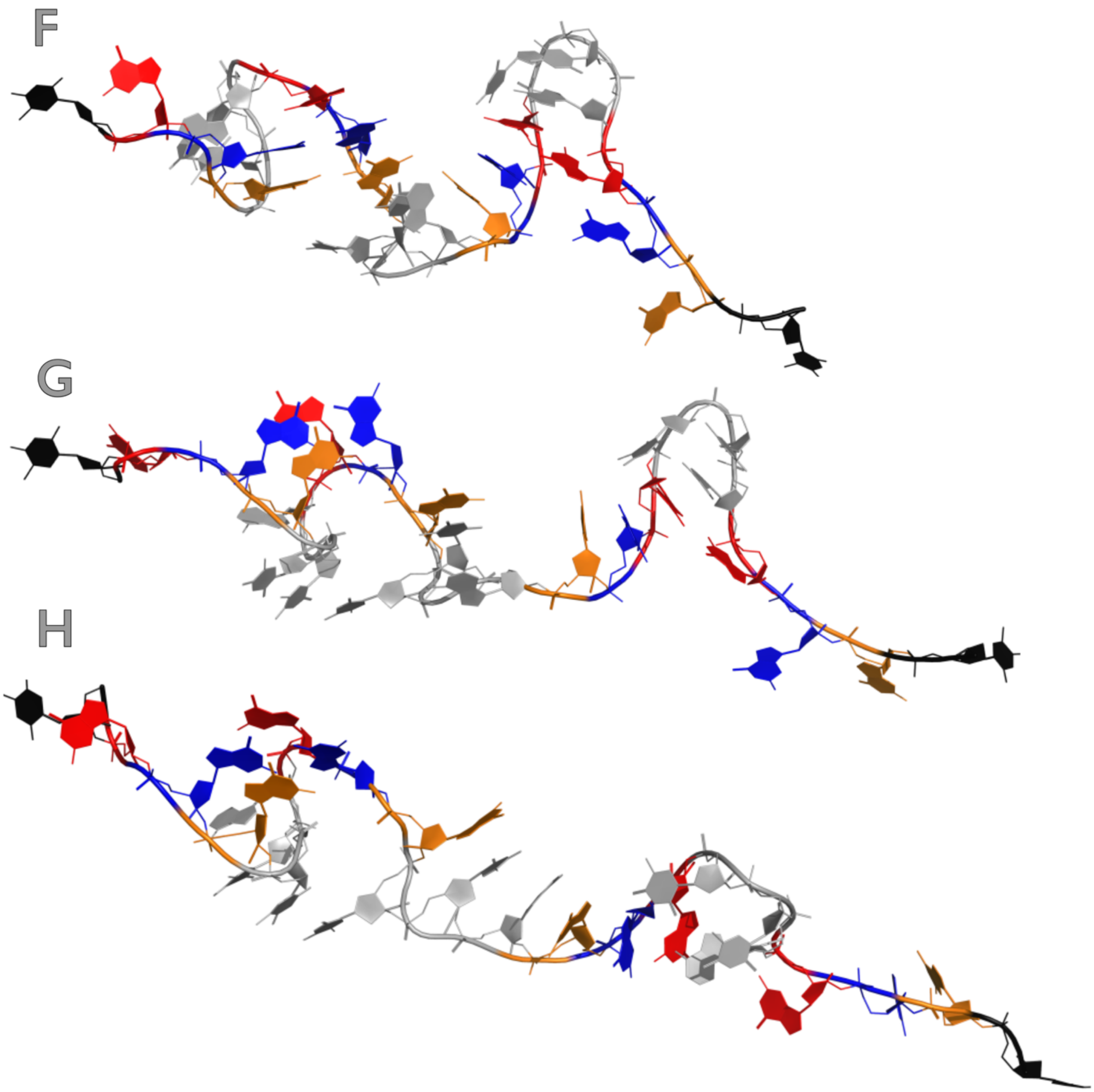

Figure S32C: Most important structural events during second independent *fast pulling* simulation of $2GKU_{box150Å}$ GQ system. See legend of Figure S1B for more details.

2GKU-box150Å_run3 (part1)

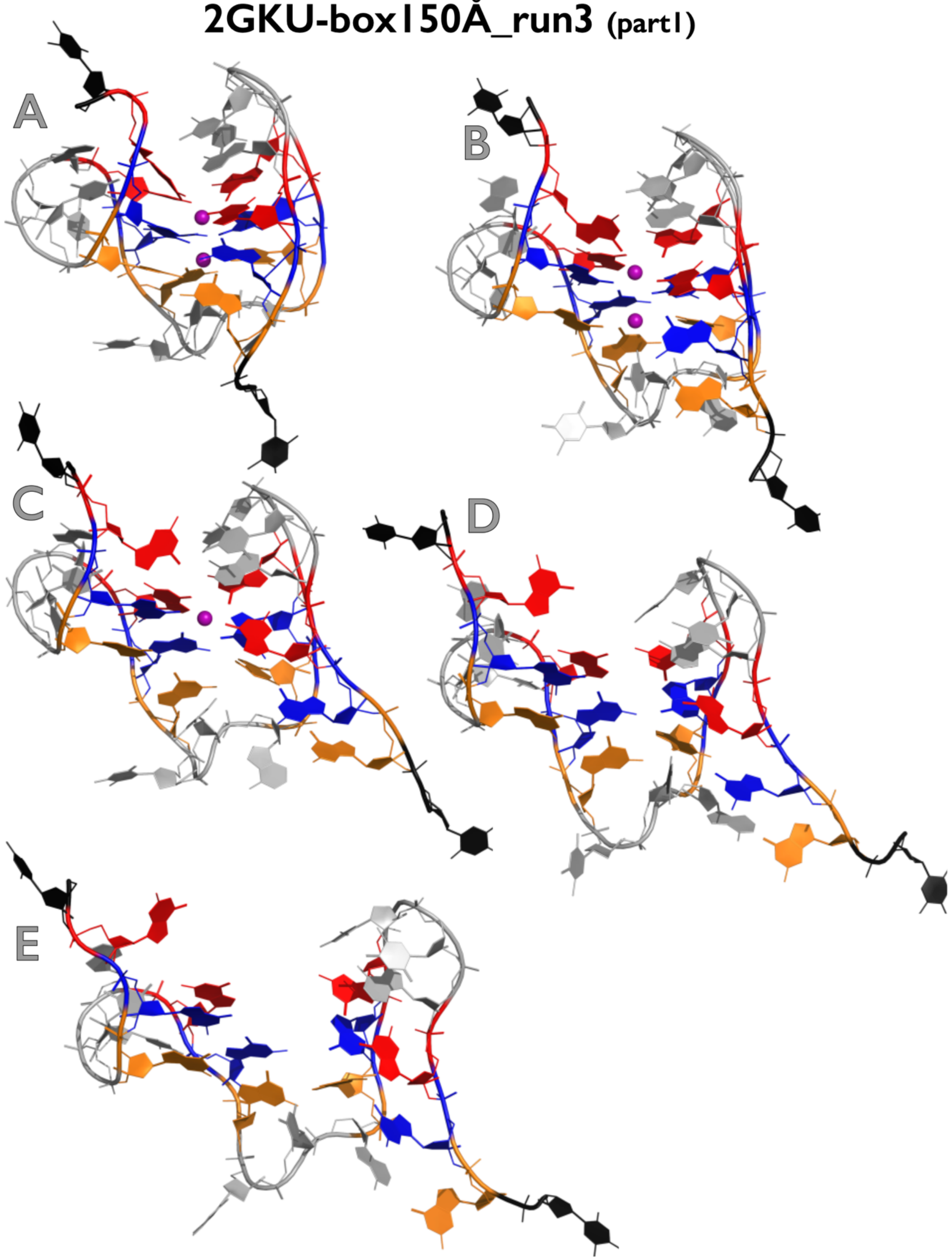

Figure continuing on the next page

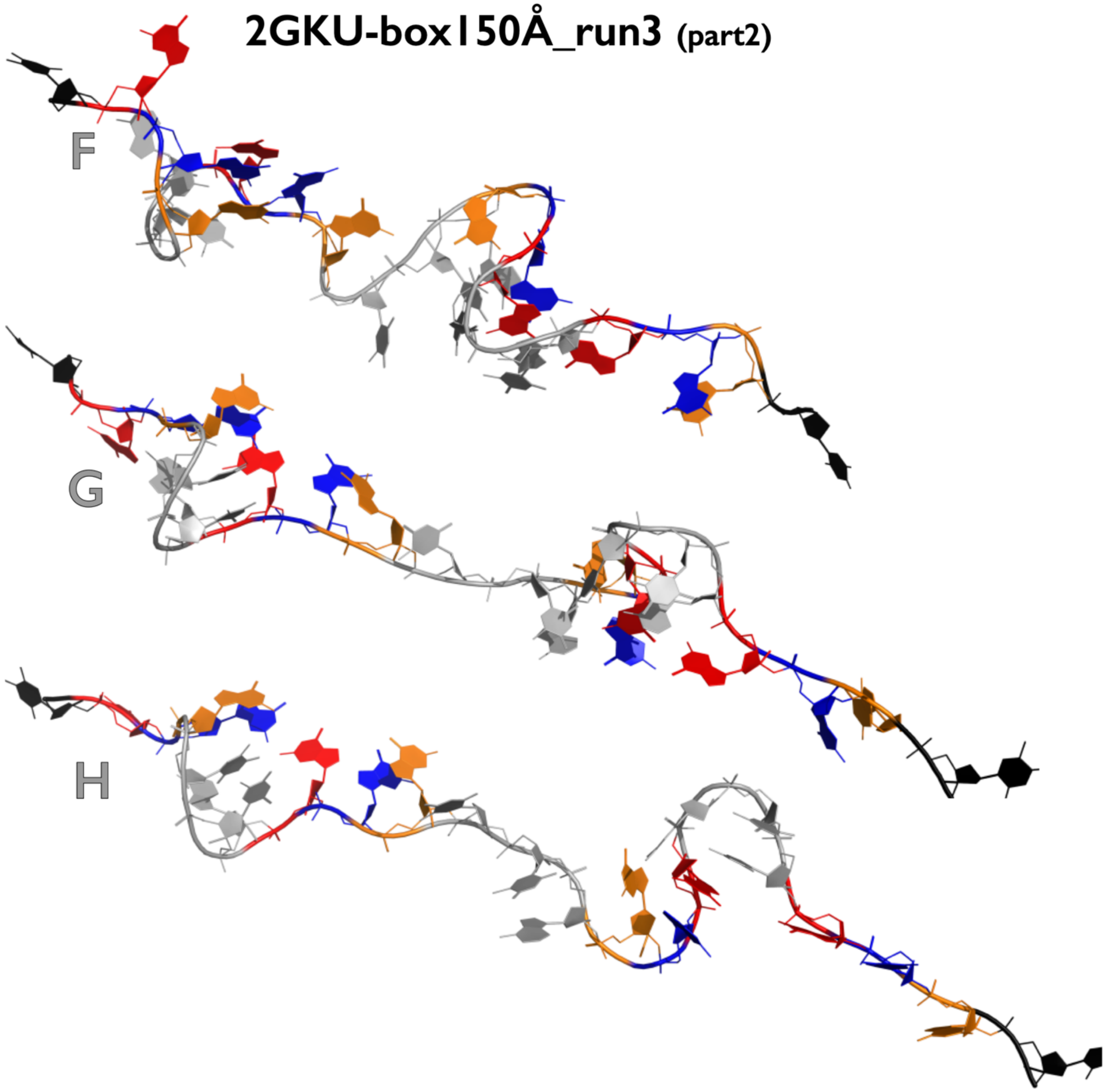

Figure S32D: Most important structural events during third independent *fast pulling* simulation of 2GKU$_{box150Å}$ GQ system. See legend of Figure S1B for more details.

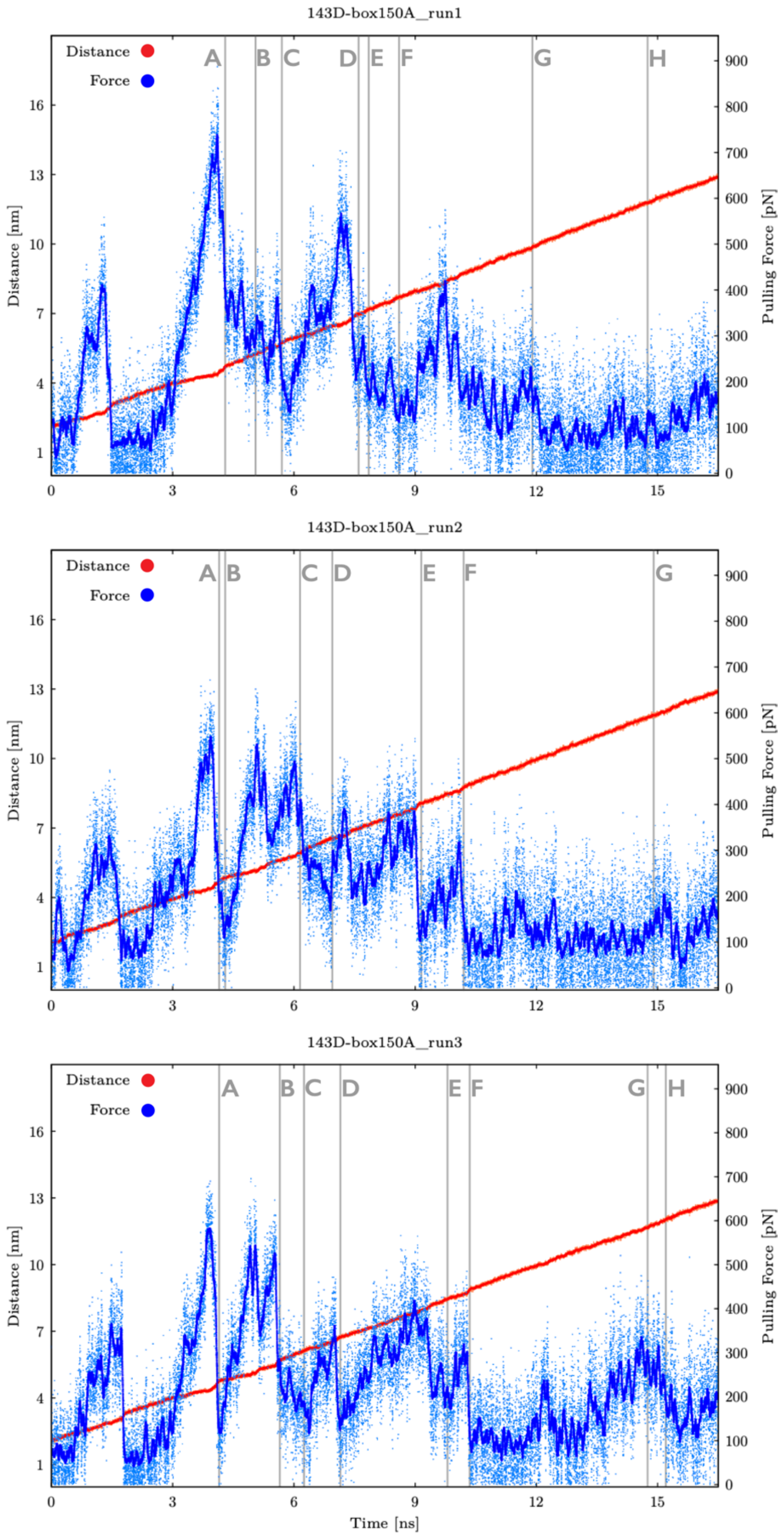

Figure S33A: Time evolution of distance between pulling centers and pulling force during three independent *fast pulling* simulations of 143D$_{box150Å}$ GQ system (see legend of Figure S1A for more details). See Figures S33B-S33D for inspection of structures corresponding to main structural events.

143D-box150Å_run1 (part1)

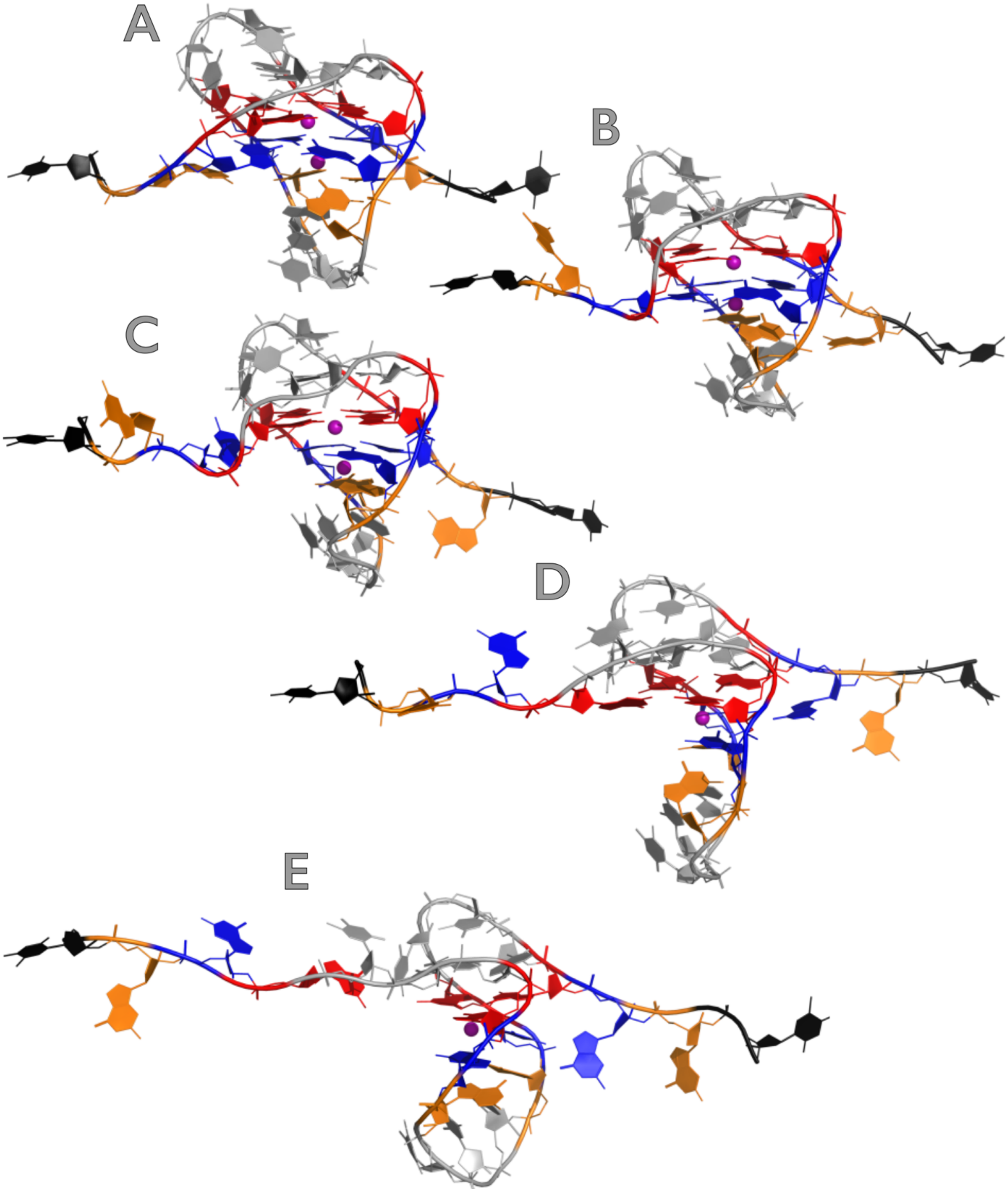

Figure continuing on the next page

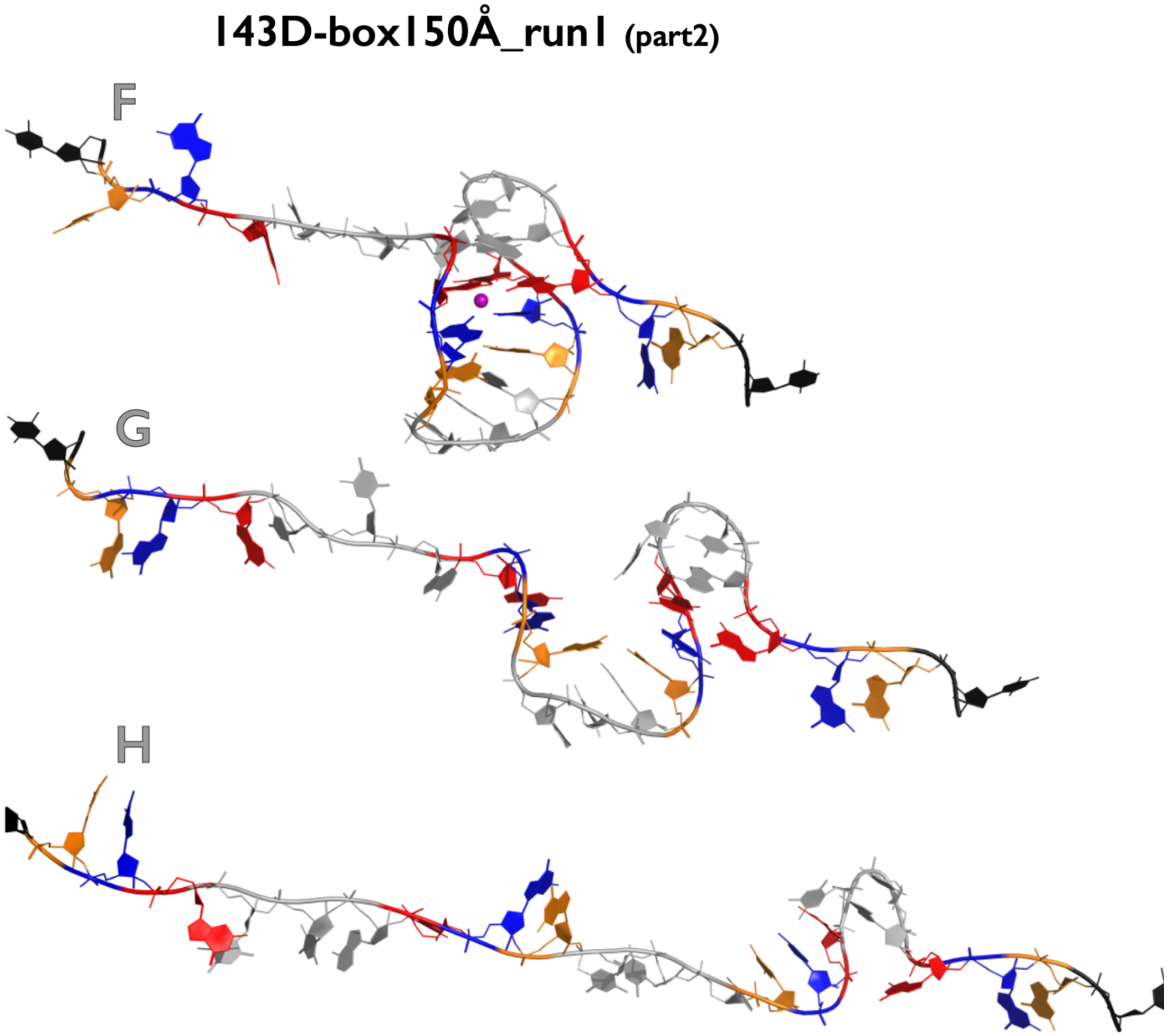

Figure S33B: Most important structural events during first independent *fast pulling* simulation of $143\mathrm{D}_{\mathrm{box150Å}}$ GQ system. See legend of Figure S1B for more details.

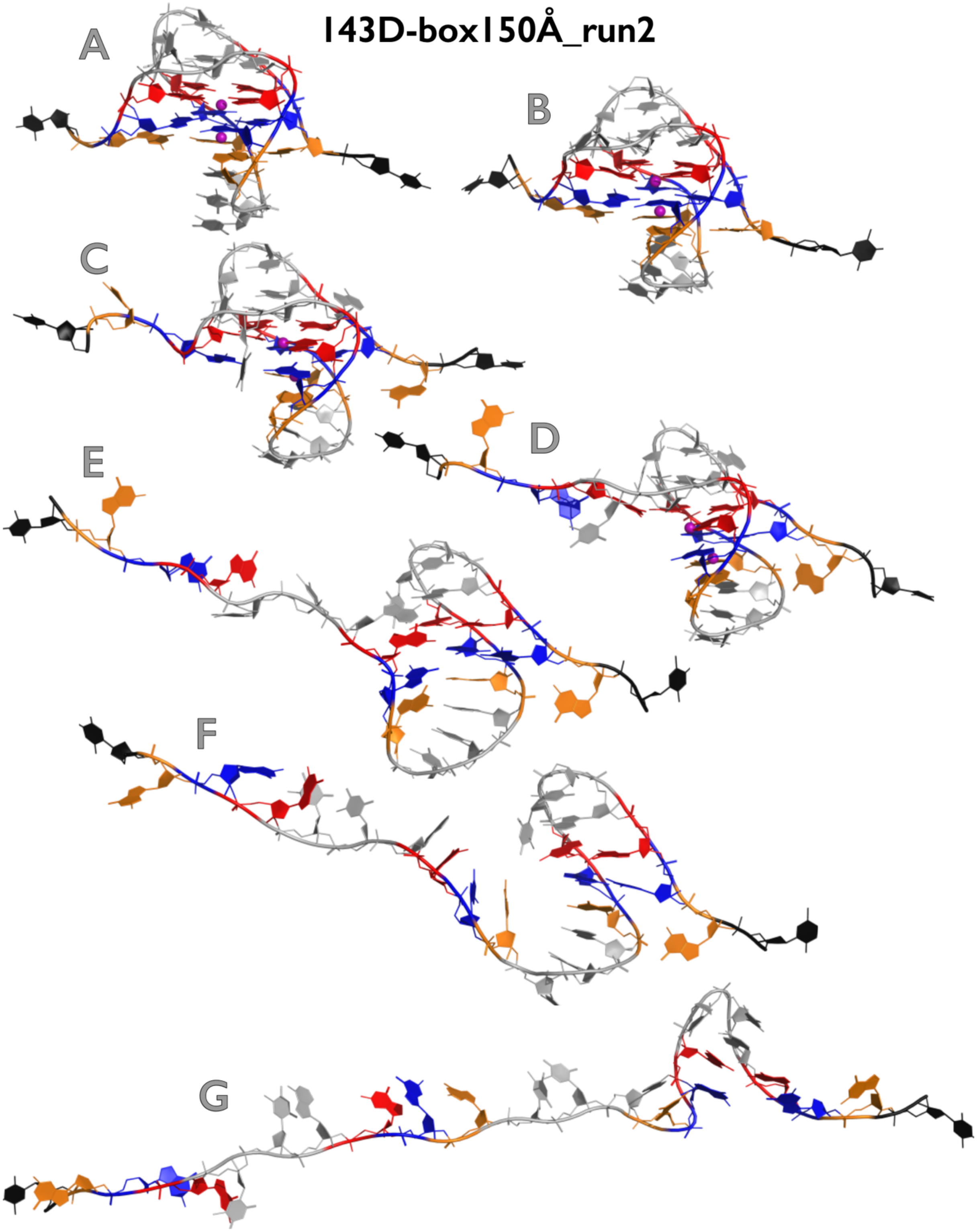

Figure S33C: Most important structural events during second independent *fast pulling* simulation of 143D$_{box150Å}$ GQ system. See legend of Figure S1B for more details.

143D-box150Å_run3 (part 1)

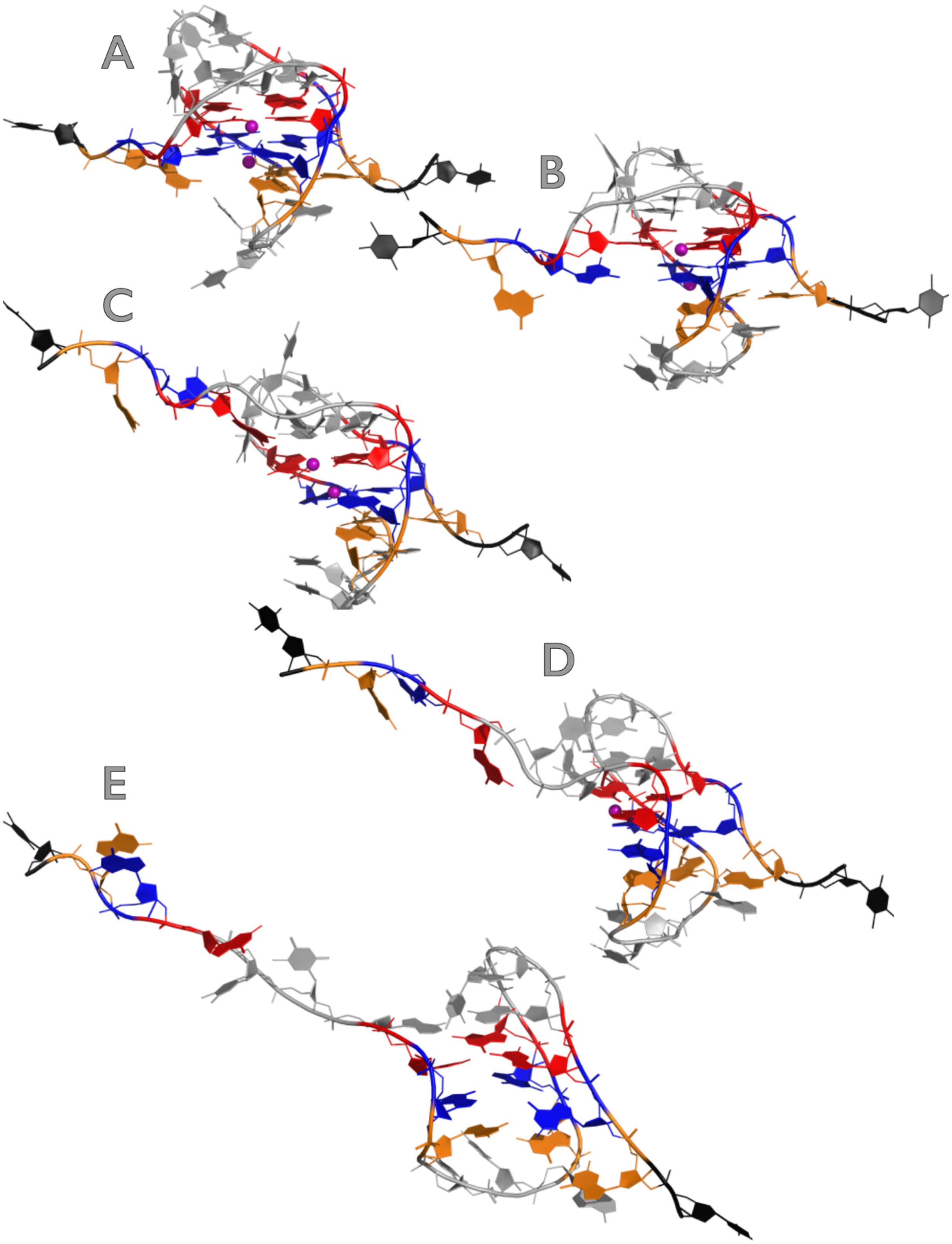

Figure continuing on the next page

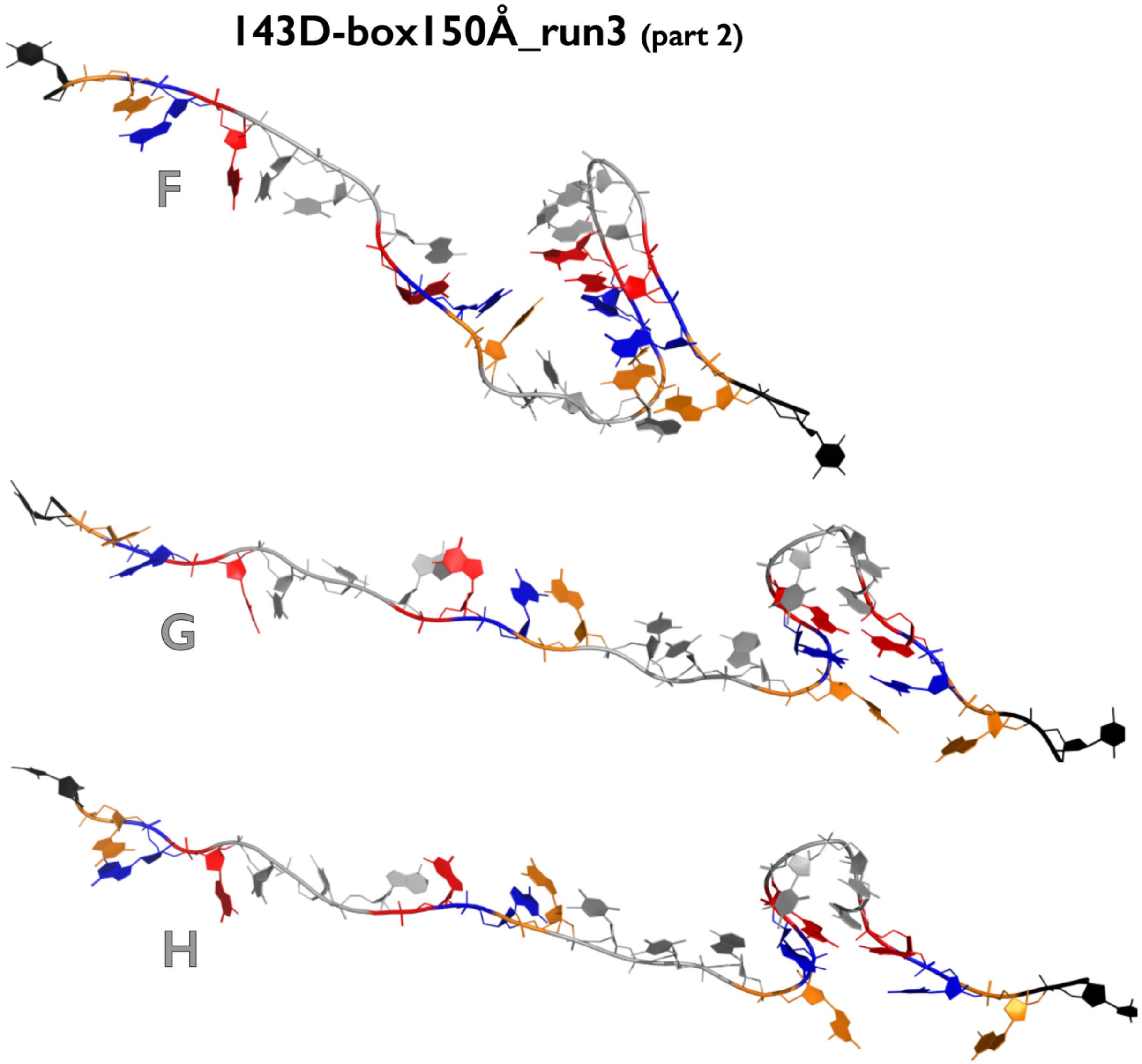

Figure S33D: Most important structural events during third independent *fast pulling* simulation of 143D$_{box150Å}$ GQ system. See legend of Figure S1B for more details.

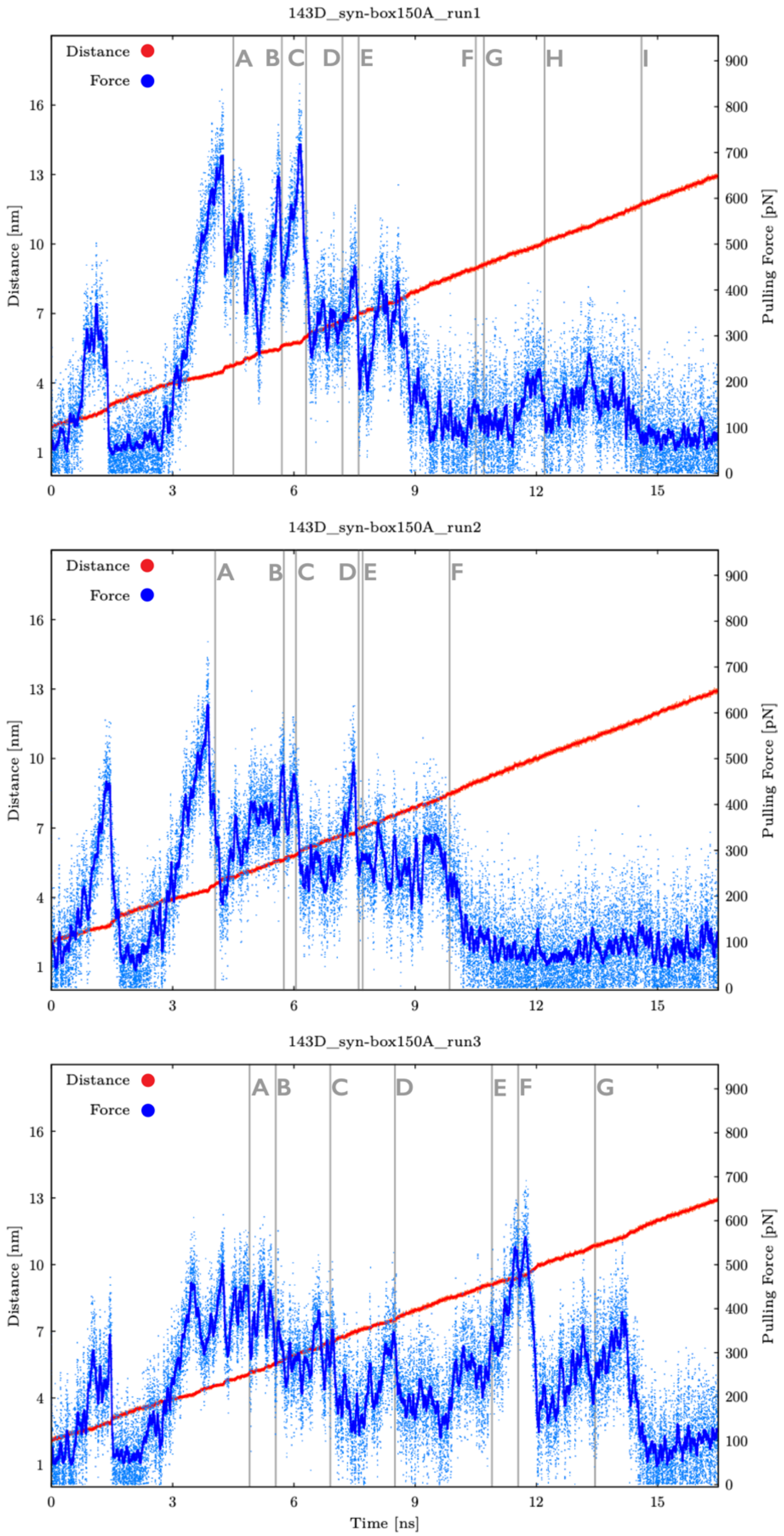

Figure S34A: Time evolution of distance between pulling centers and pulling force during three independent *fast pulling* simulations of 143D$_{syn\text{-box150Å}}$ GQ system (see legend of Figure S1A for more details). See Figures S34B-S34D for inspection of structures corresponding to main structural events.

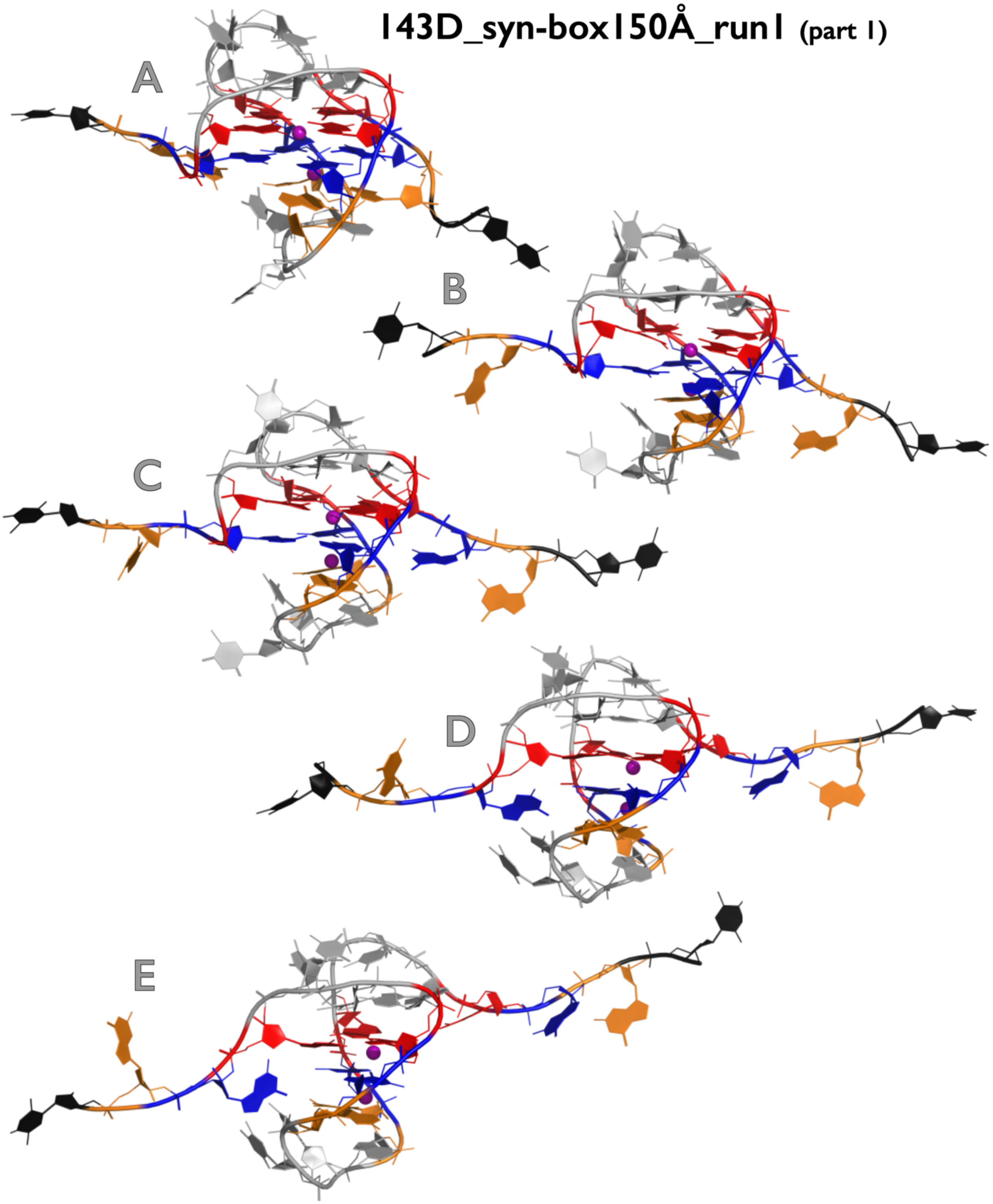

Figure continuing on the next page

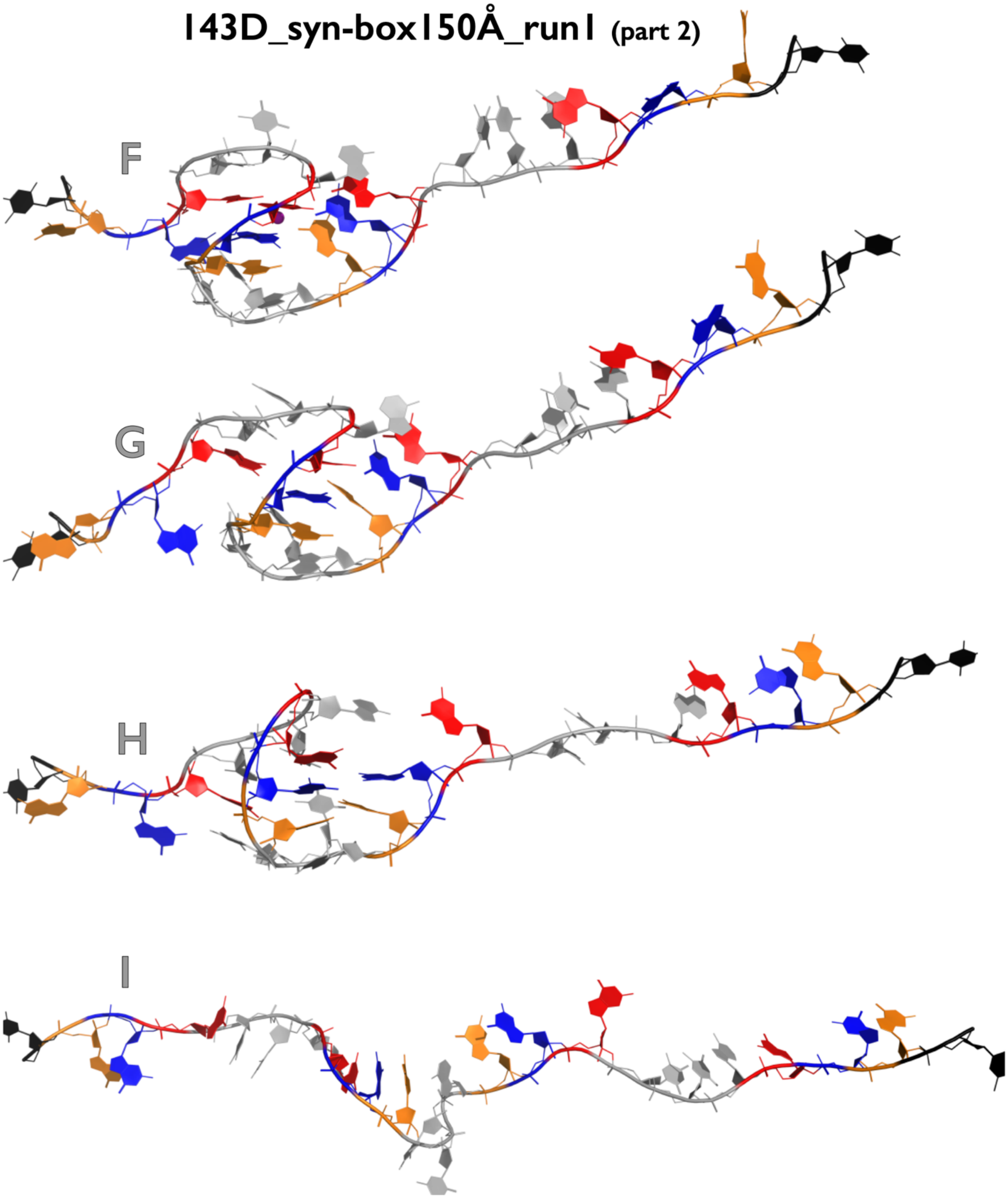

Figure S34B: Most important structural events during first independent *fast pulling* simulation of 143D$_{syn\text{-box150Å}}$ GQ system. See legend of Figure S1B for more details.

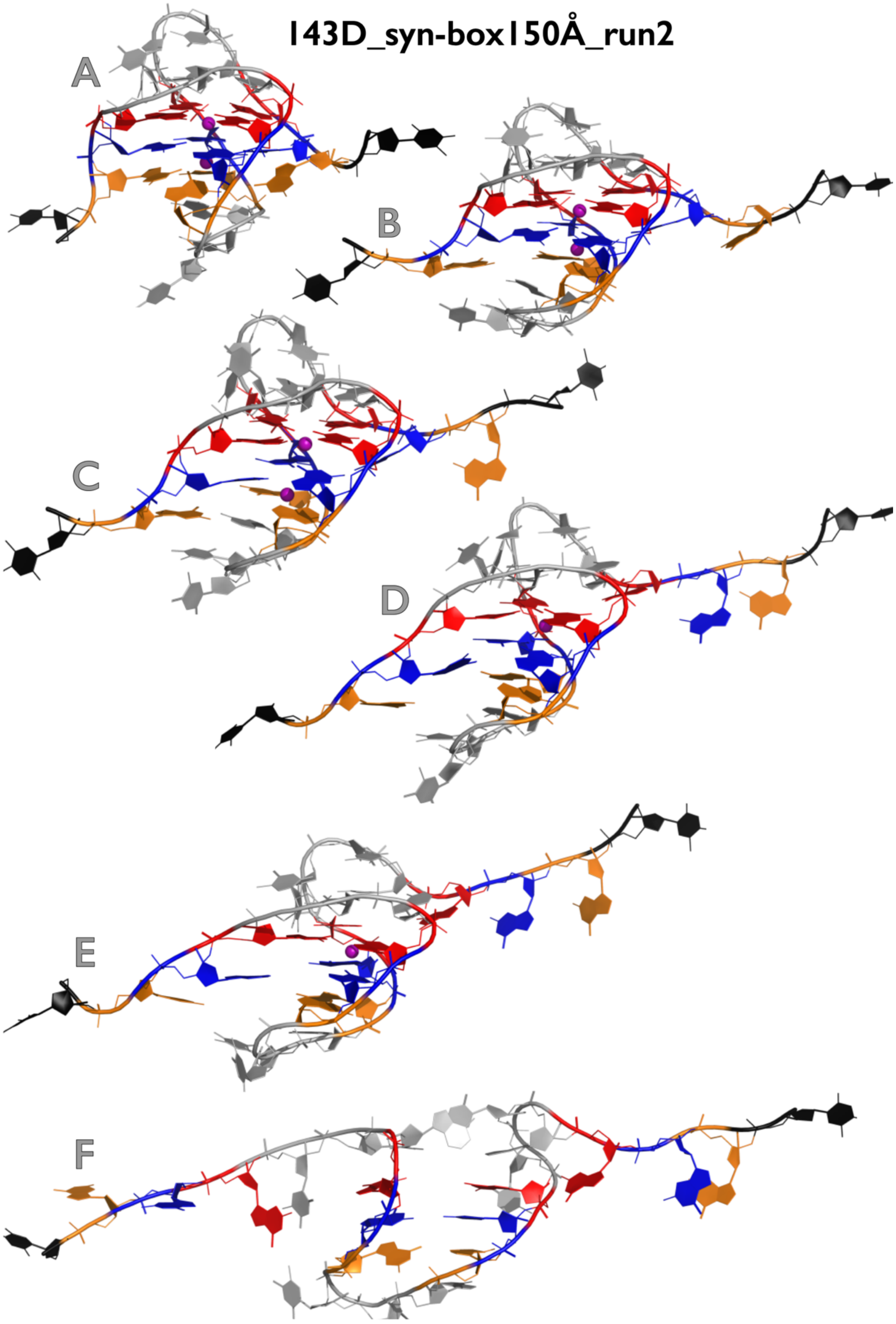

Figure S34C: Most important structural events during second independent *fast pulling* simulation of 143D$_{syn\text{-}box150Å}$ GQ system. See legend of Figure S1B for more details.

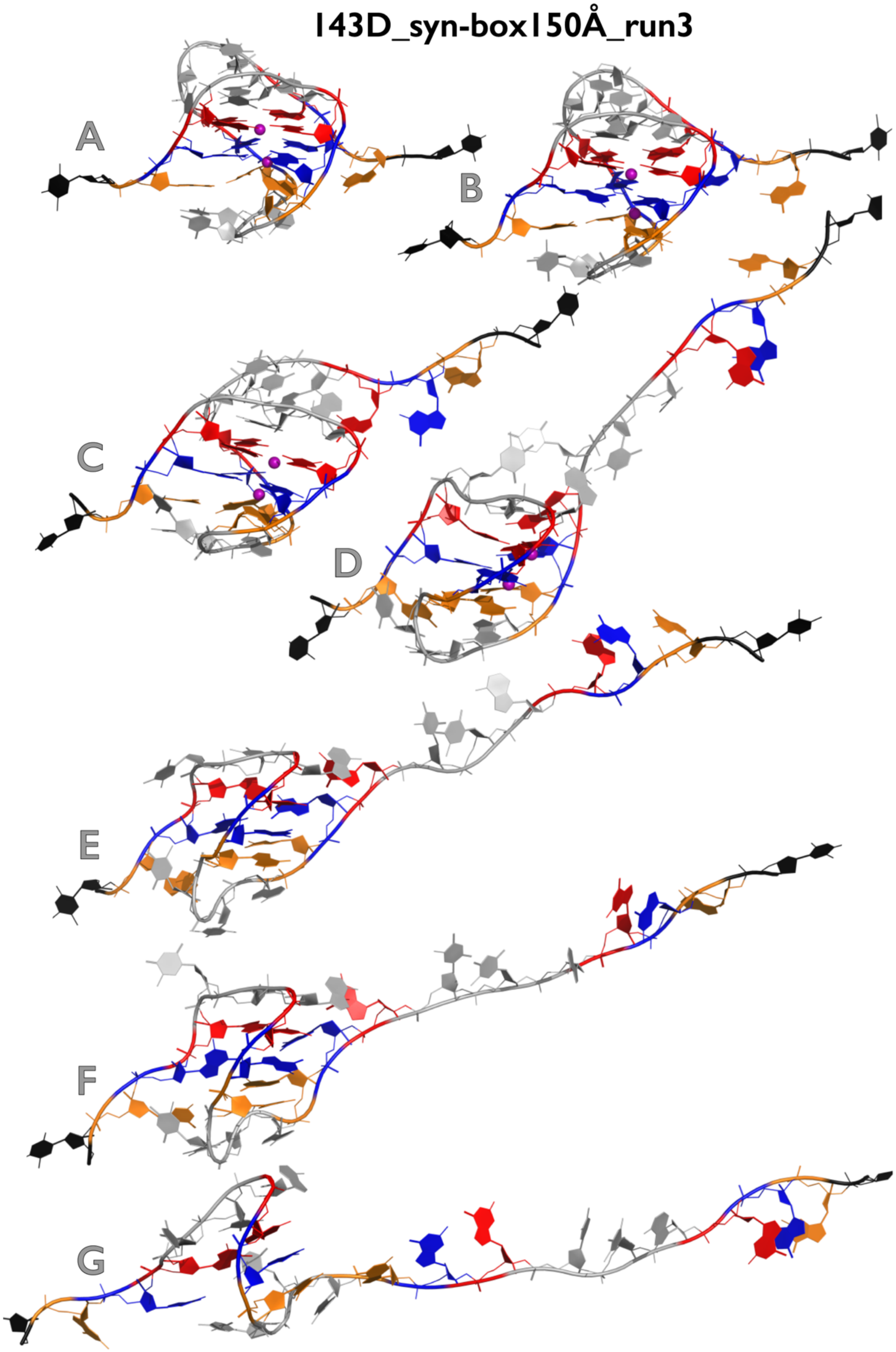

Figure S34D: Most important structural events during third independent *fast pulling* simulation of 143D$_{syn\text{-}box150Å}$ GQ system. See legend of Figure S1B for more details.

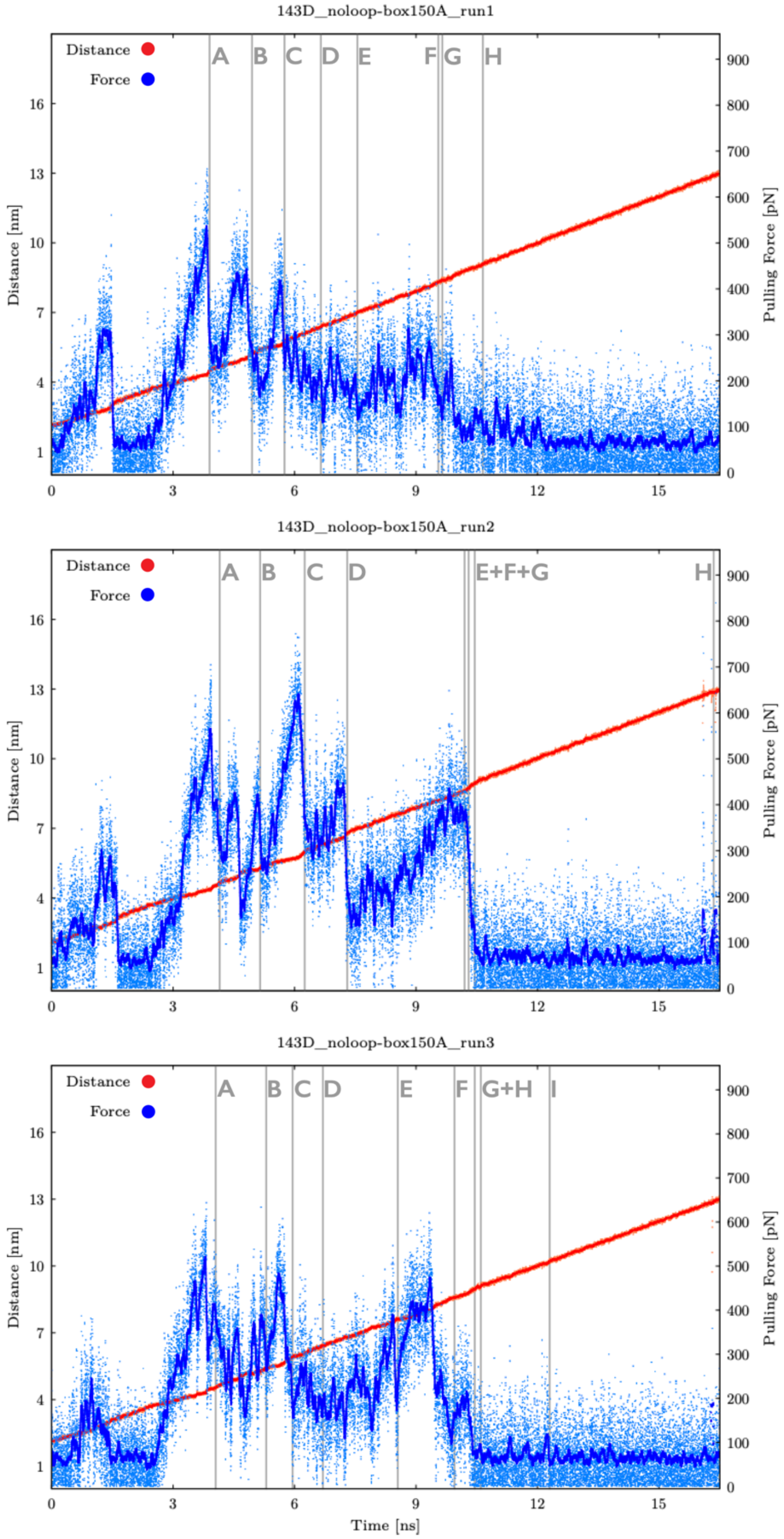

Figure S35A: Time evolution of distance between pulling centers and pulling force during three independent *fast pulling* simulations of 143D$_{\text{noloop-box150Å}}$ GQ system (see legend of Figure S1A for more details). See Figures S35B-S35D for inspection of structures corresponding to main structural events.

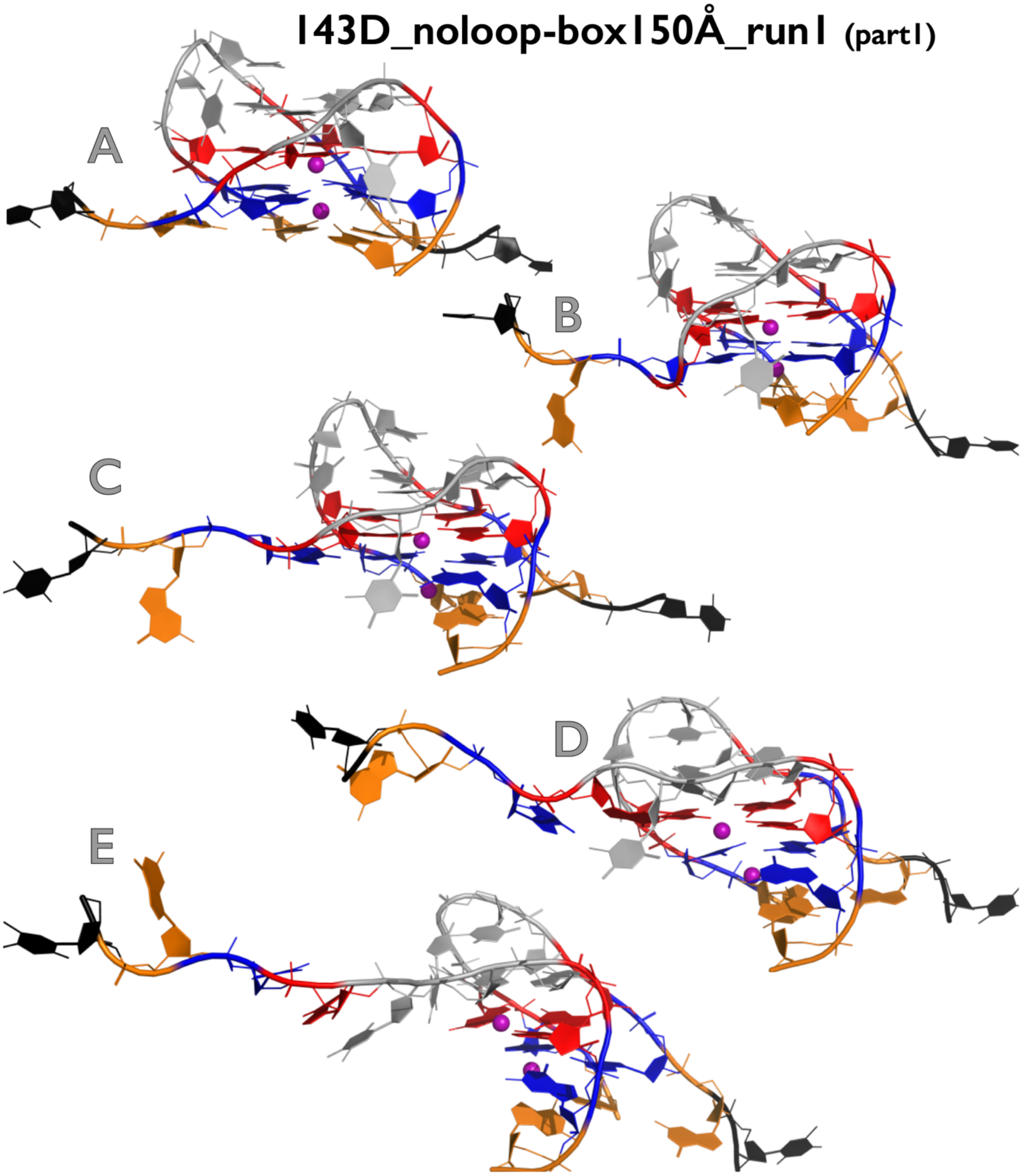

Figure continuing on the next page

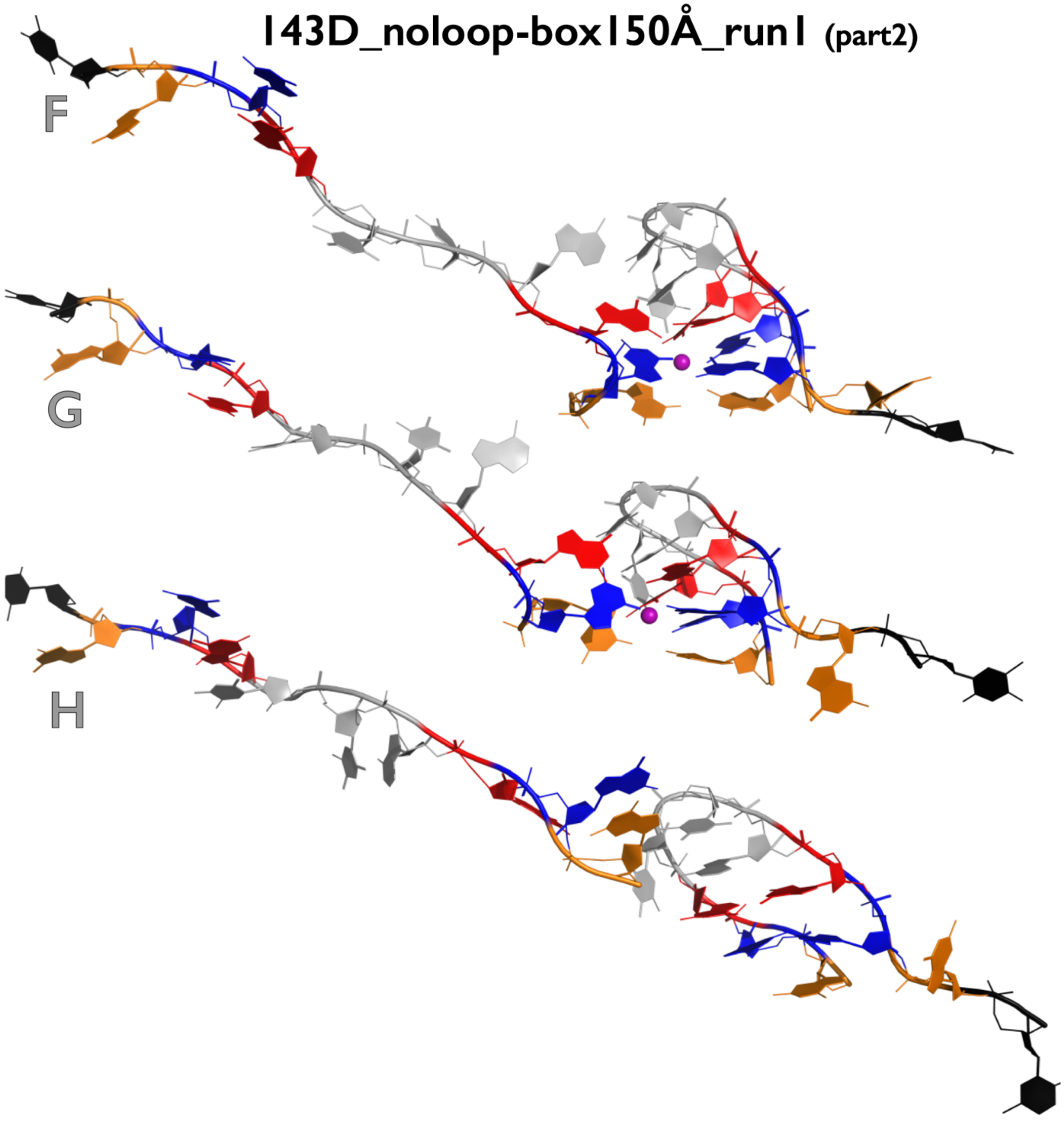

Figure S35B: Most important structural events during first independent *fast pulling* simulation of $143D_{noloop\text{-}box150Å}$ GQ system. See legend of Figure S1B for more details.

143D_noloop-box150Å_run2 (part1)

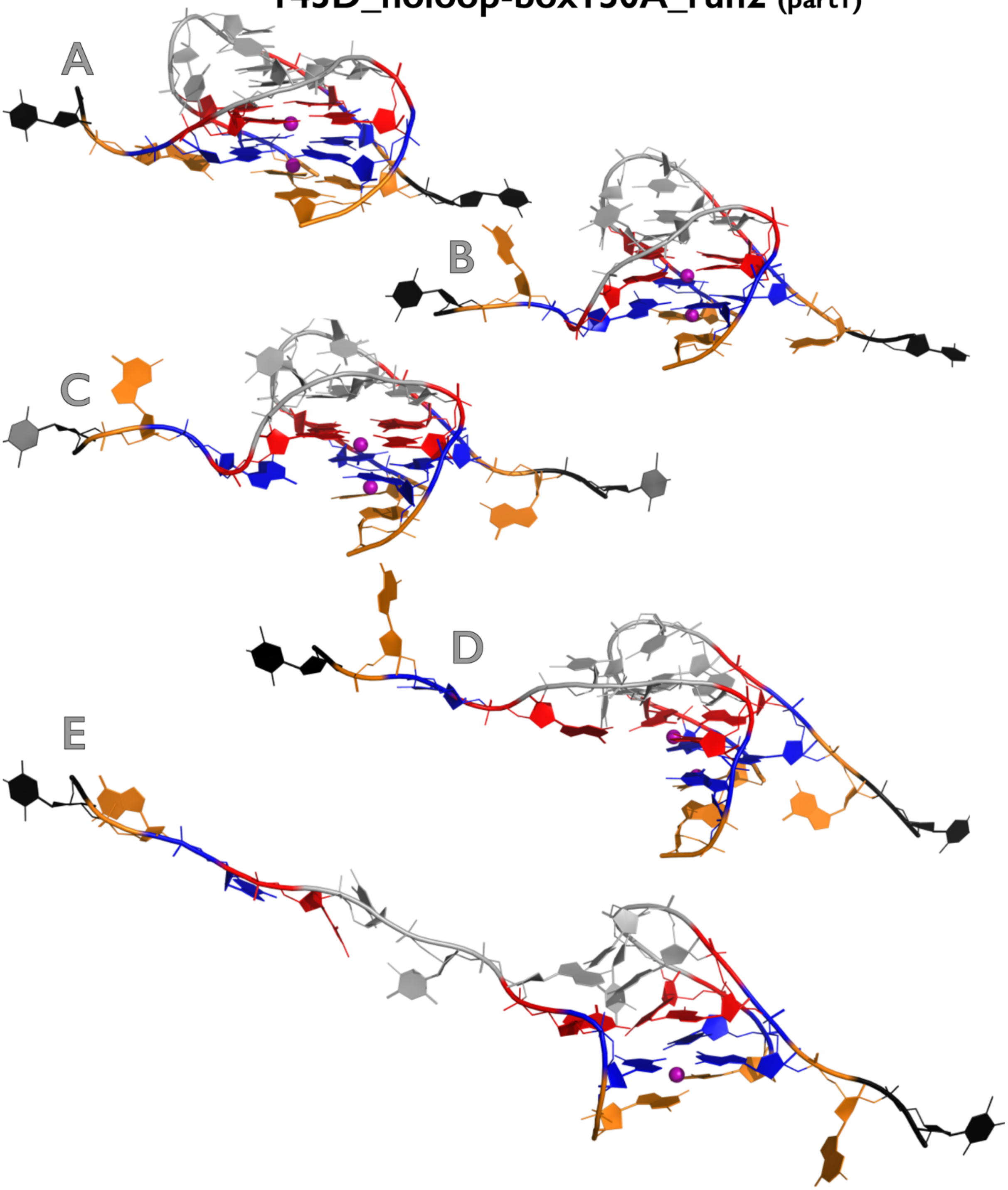

Figure continuing on the next page

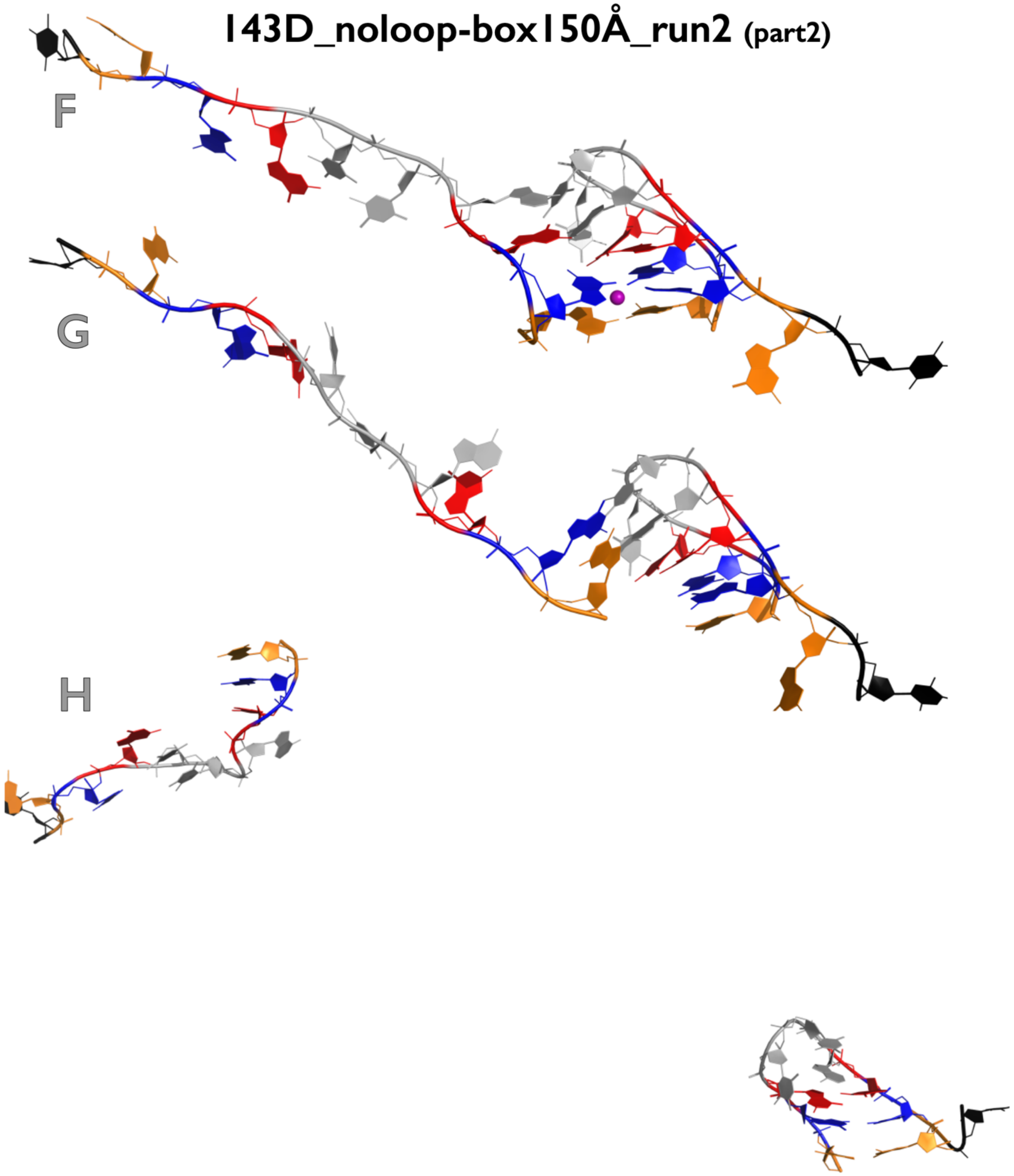

Figure S35C: Most important structural events during second independent *fast pulling* simulation of 143D$_{noloop\text{-}box150Å}$ GQ system. See legend of Figure S1B for more details.

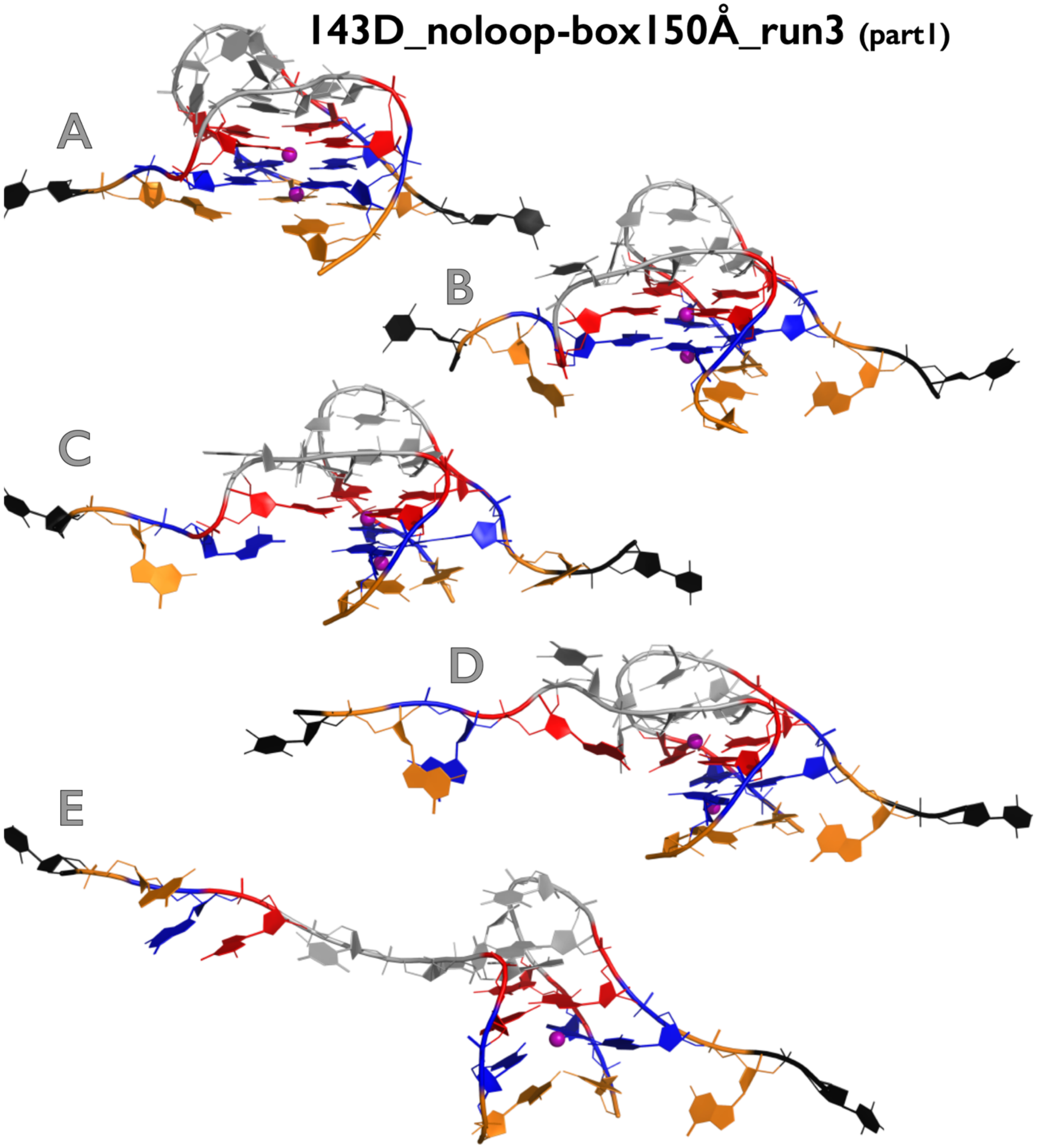

Figure continuing on the next page

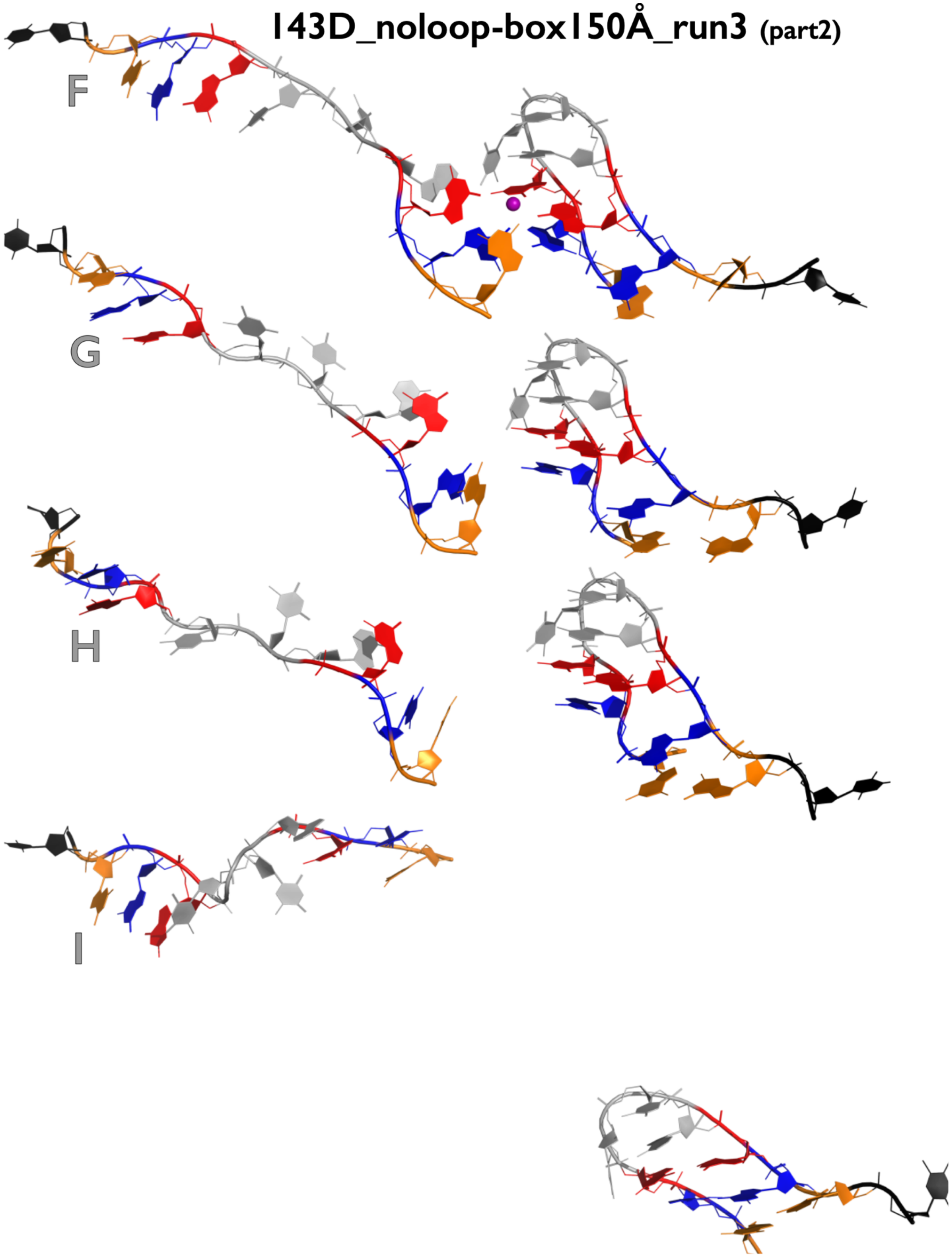

Figure S35D: Most important structural events during third independent *fast pulling* simulation of 143D$_{\text{noloop-box150Å}}$ GQ system. See legend of Figure S1B for more details.

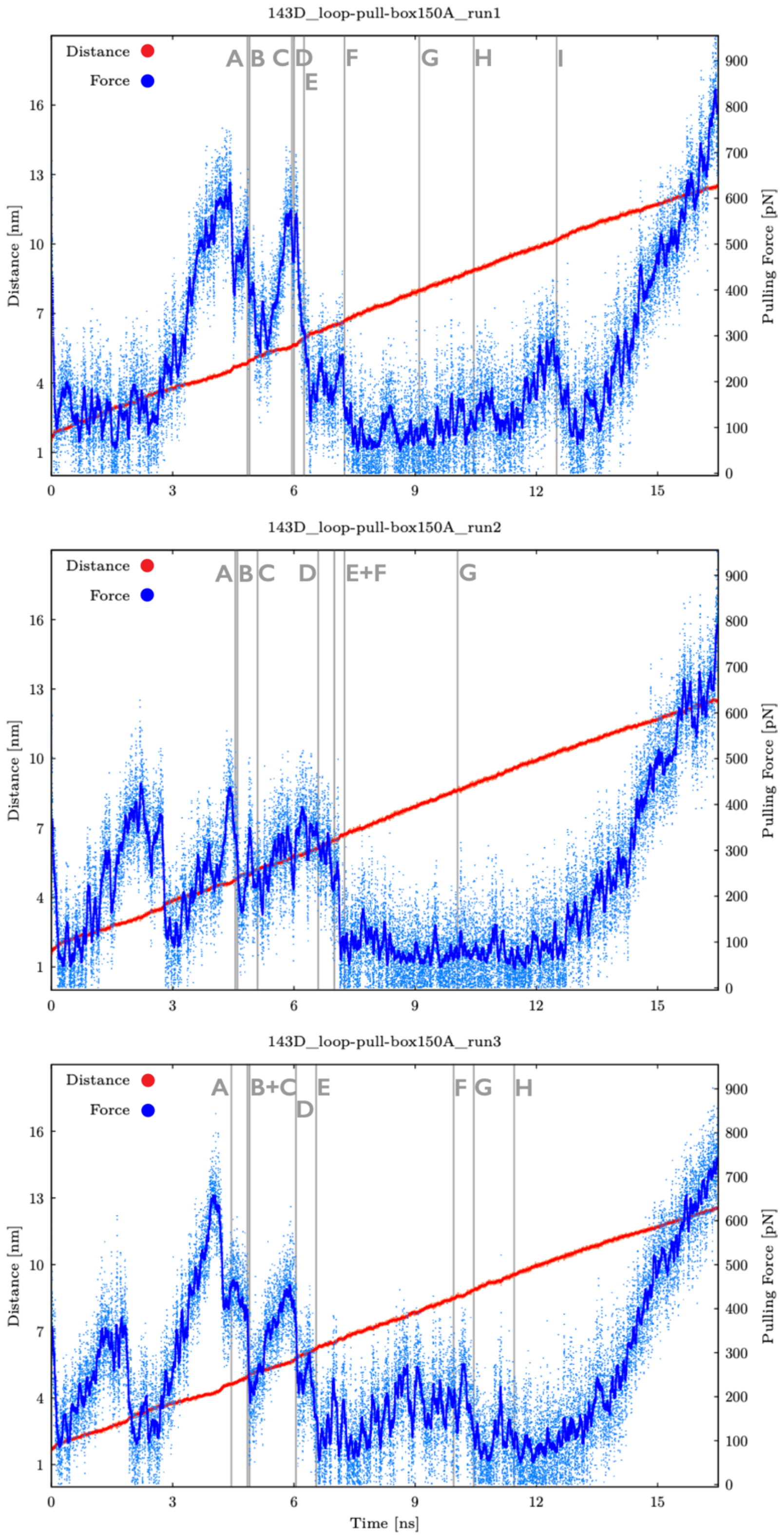

Figure S36A: Time evolution of distance between pulling centers and pulling force during three independent *fast pulling* simulations of $143D_{\text{loop-pull-box150Å}}$ GQ system (see legend of Figure S1A for more details). The pulling setup reached maximum allowed extension for the $143D_{\text{loop-pull-box150Å}}$ system and huge increase of the force towards the end of each simulation corresponds to

excessive stretching of the backbone. See Figures S36B-S36D for inspection of structures corresponding to main structural events.

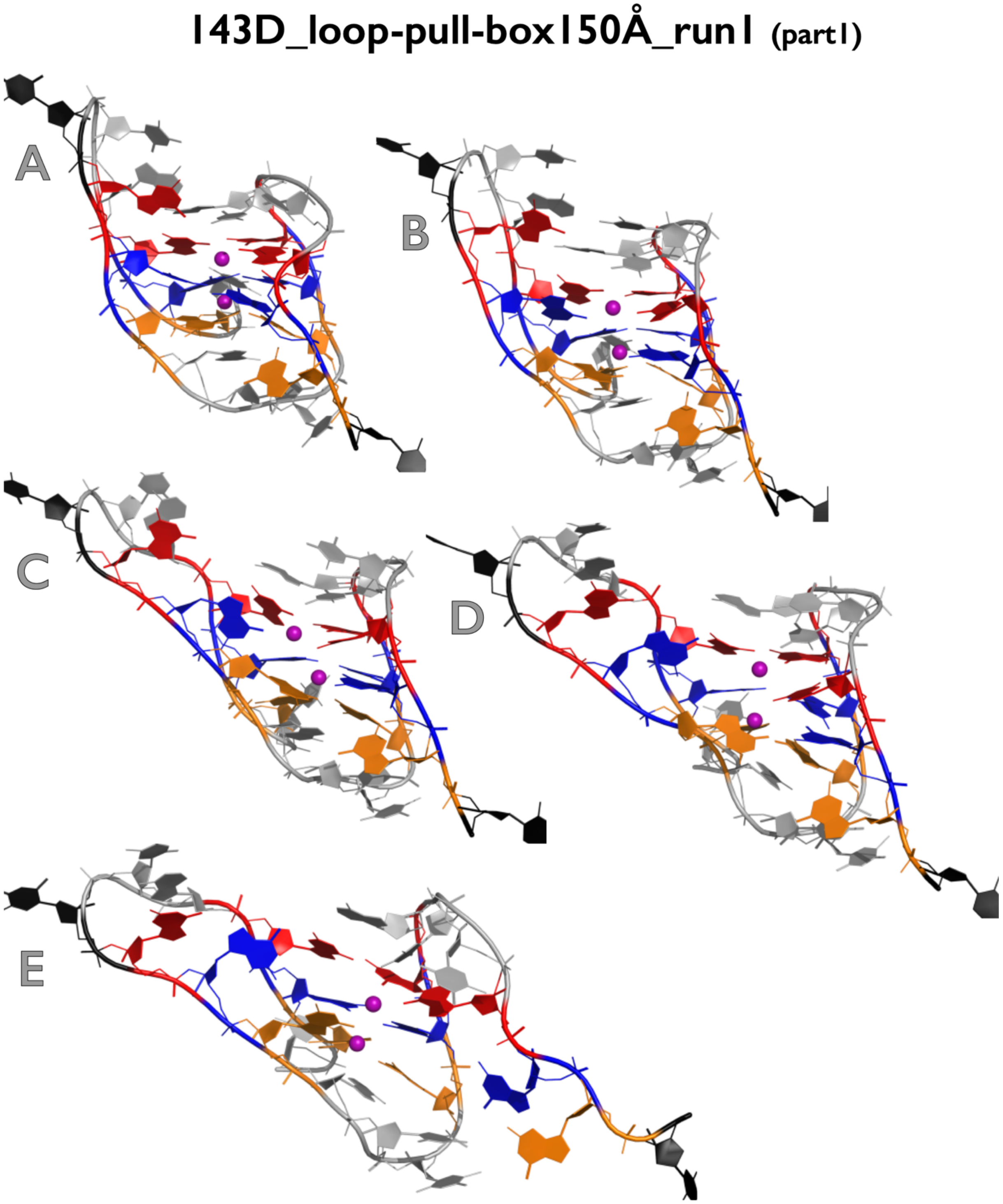

Figure continuing on the next page

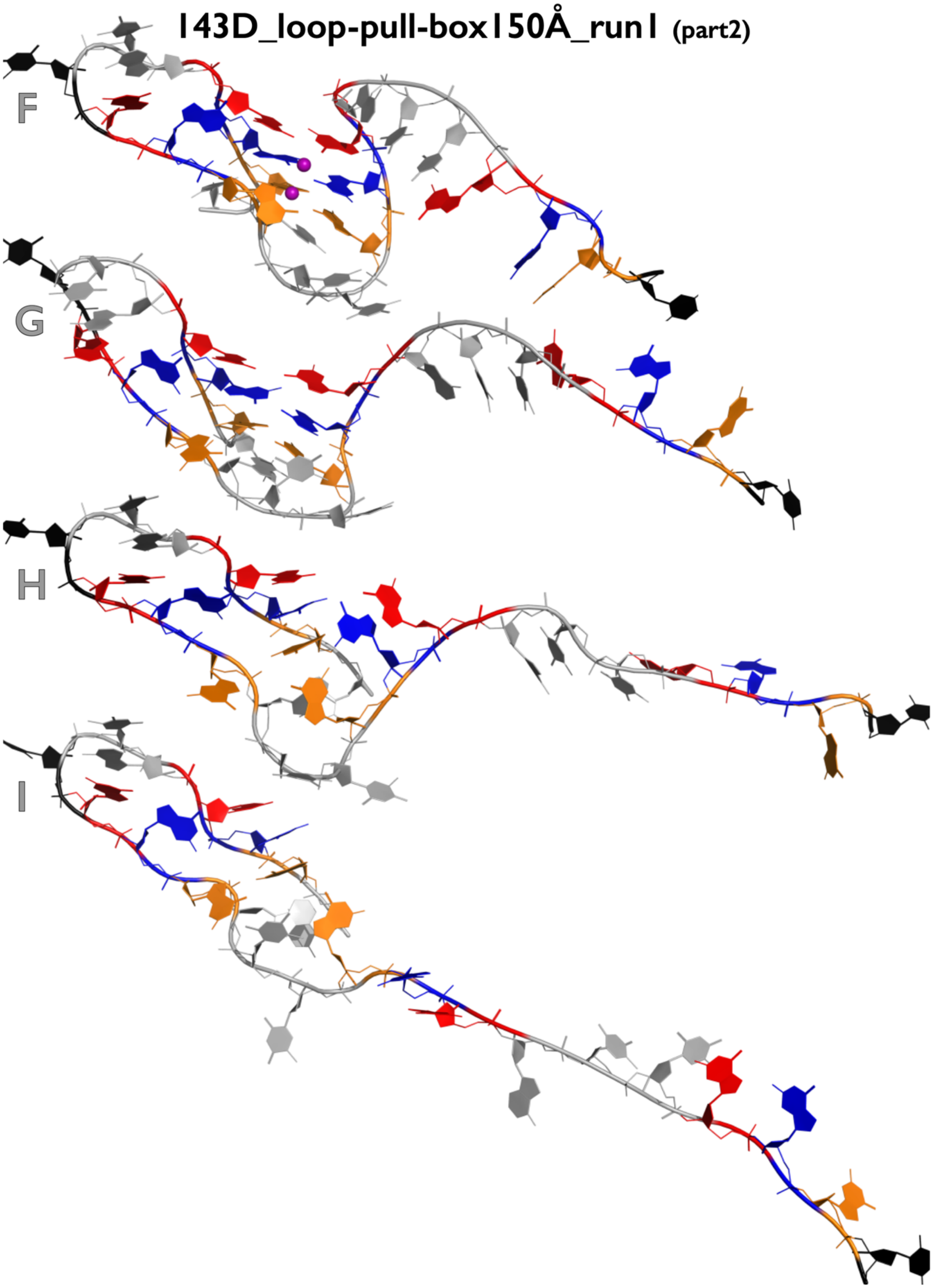

Figure S36B: Most important structural events during first independent *fast pulling* simulation of $143D_{loop\text{-}pull\text{-}box150Å}$ GQ system. See legend of Figure S1B for more details.

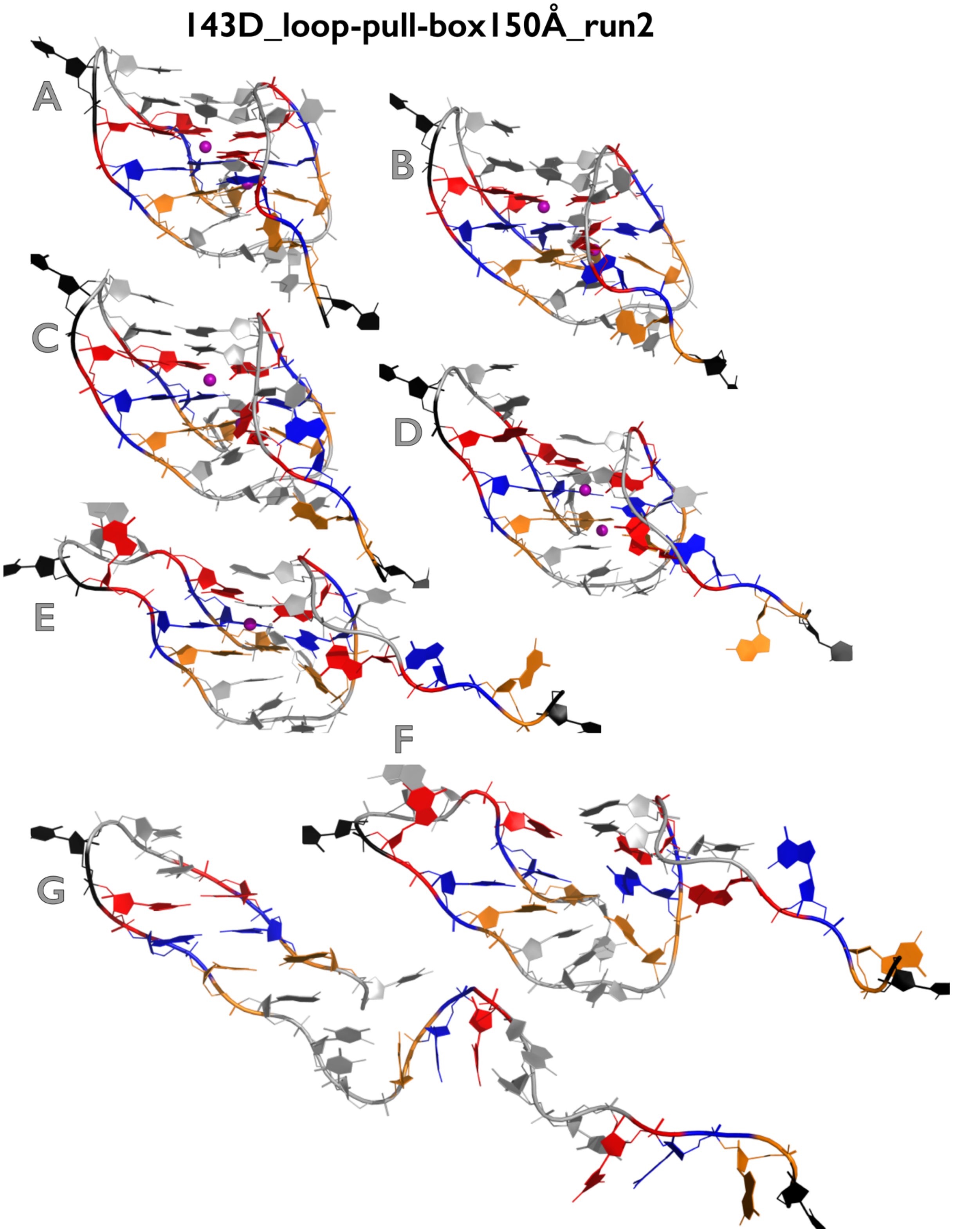

Figure S36C: Most important structural events during second independent *fast pulling* simulation of $143D_{\text{loop-pull-box150Å}}$ GQ system. See legend of Figure S1B for more details.

143D_loop-pull-box150Å_run3 (part1)

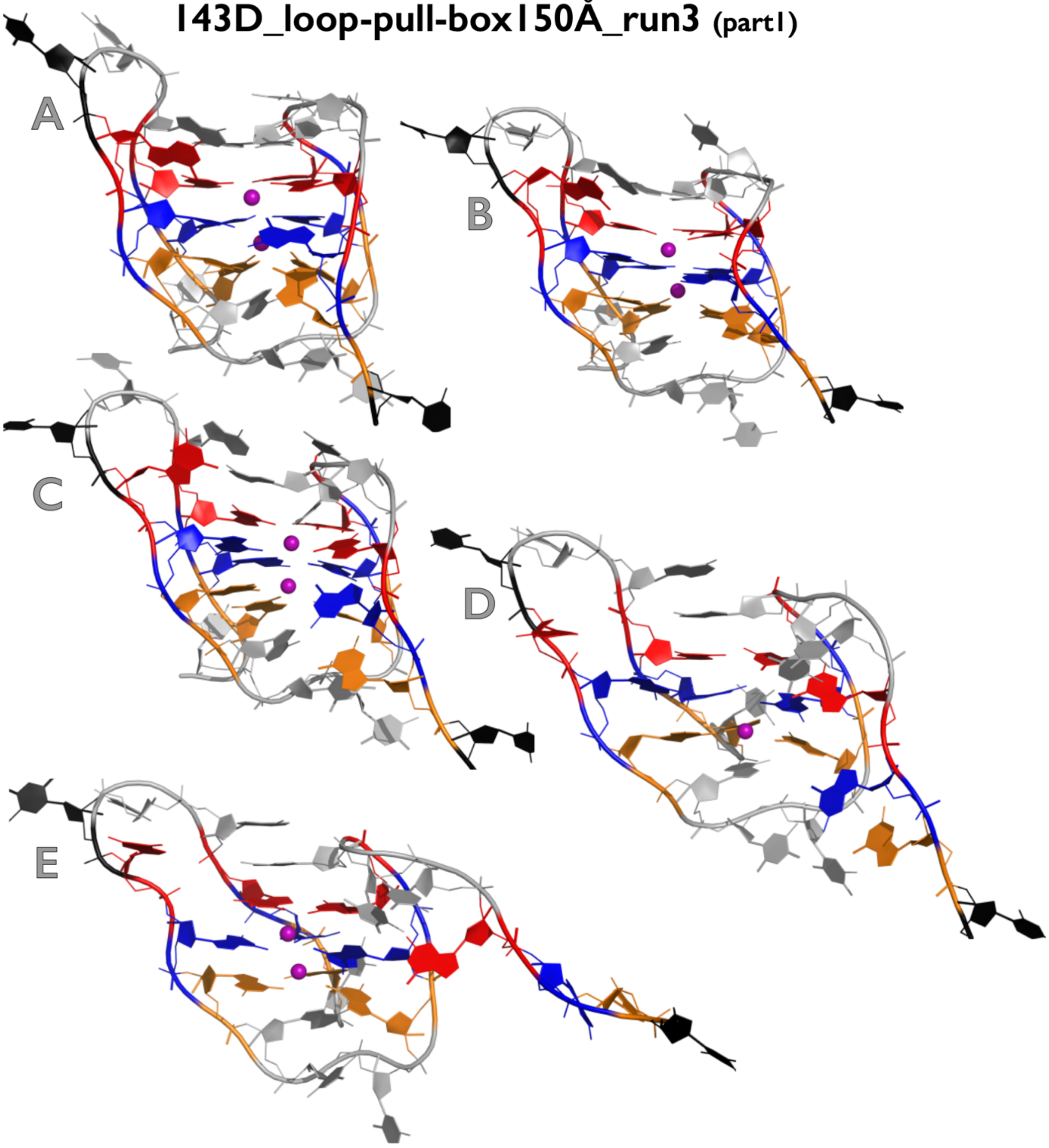

Figure continuing on the next page

143D_loop-pull-box150Å_run3 (part2)

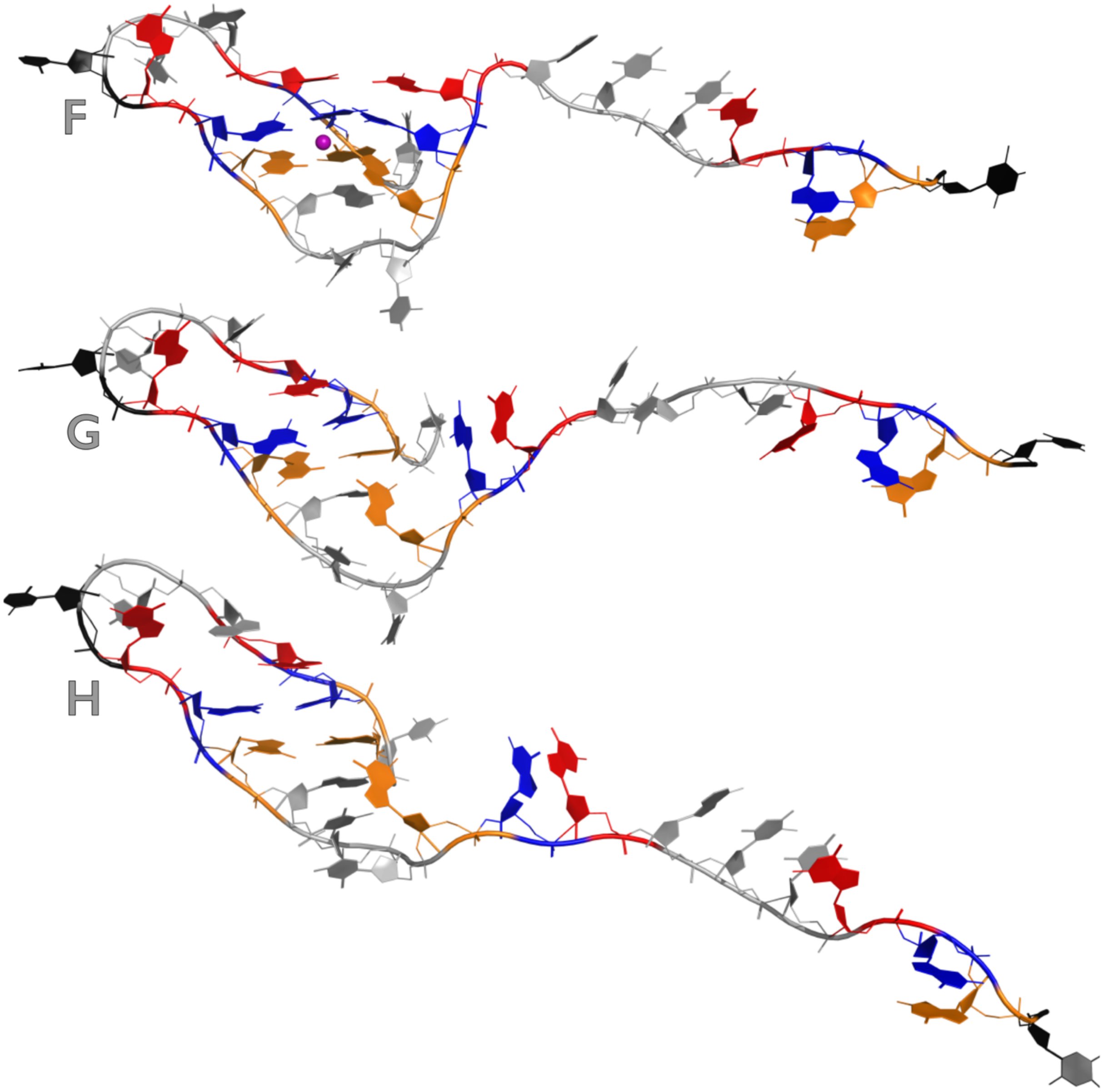

Figure S36D: Most important structural events during third independent *fast pulling* simulation of 143D$_{loop\text{-}pull\text{-}box150Å}$ GQ system. See legend of Figure S1B for more details.

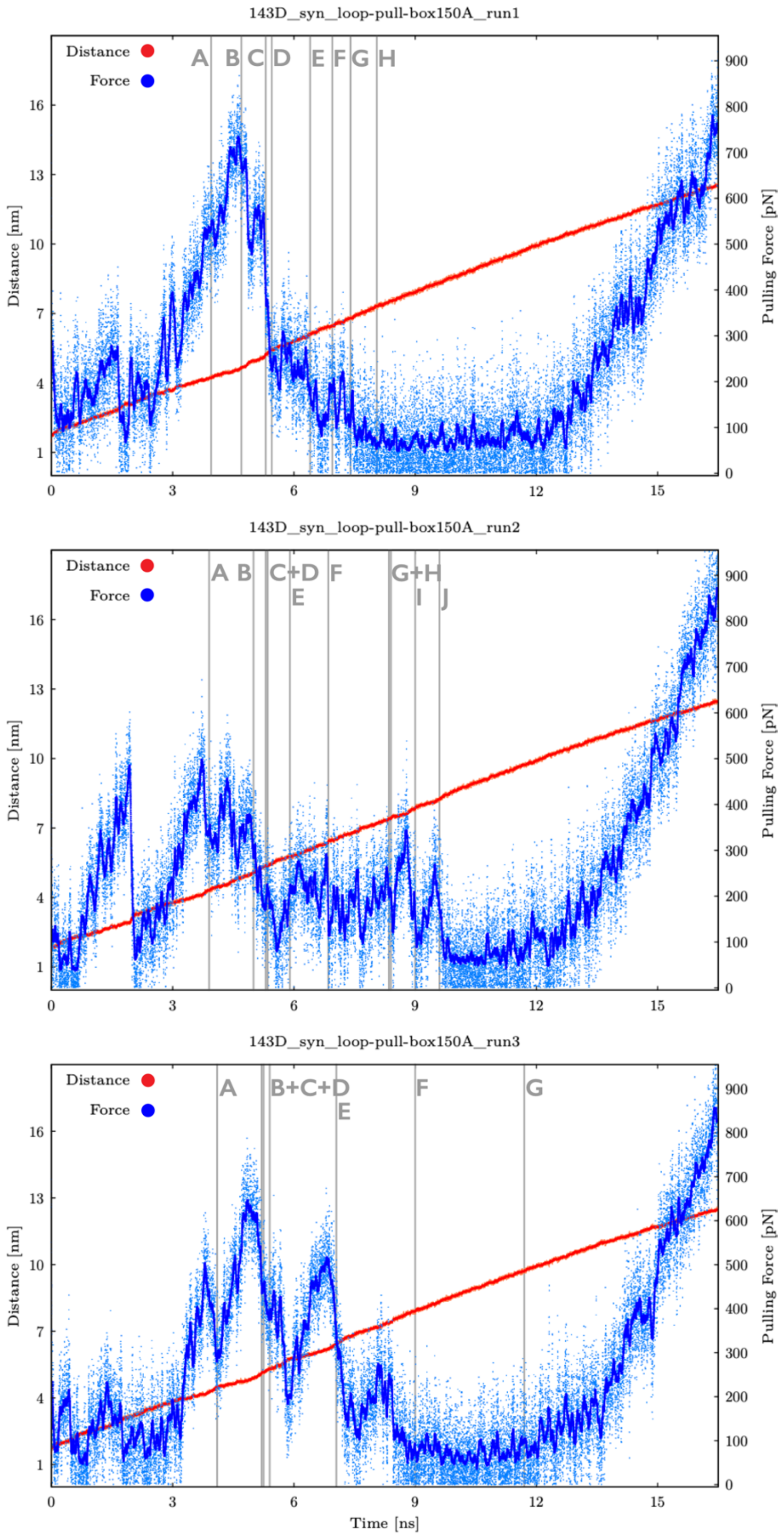

Figure S37A: Time evolution of distance between pulling centers and pulling force during three independent *fast pulling* simulations of 143D$_{\mathit{syn}\text{_loop-pull-box150Å}}$ GQ system (see legend of Figure S1A for more details). The pulling setup reached maximum allowed extension for the 143D$_{\text{loop-pull-box150Å}}$ system and huge increase of the force towards the end of each simulation corresponds to

excessive stretching of the backbone. See Figures S37B-S37D for inspection of structures corresponding to main structural events.

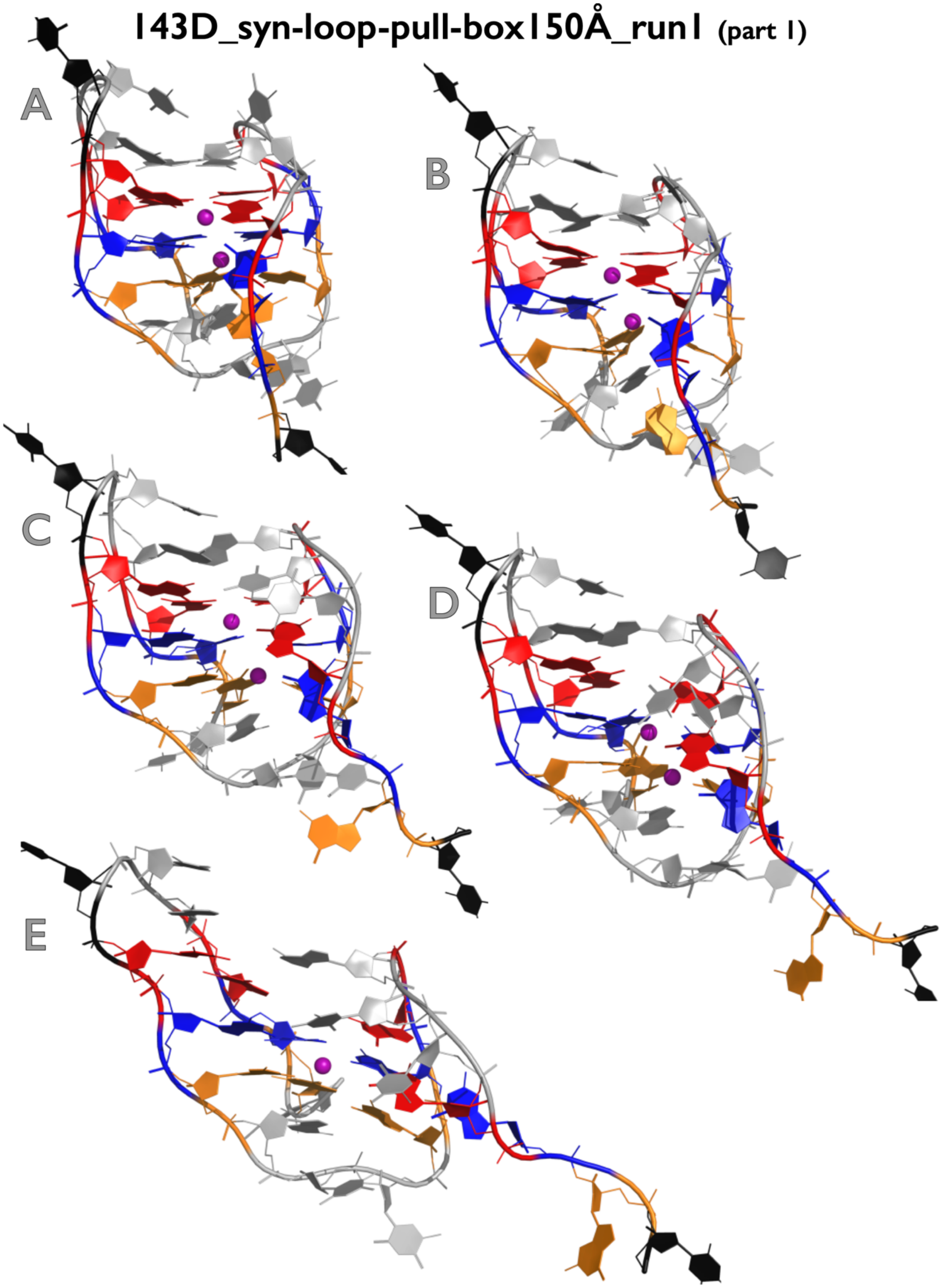

Figure continuing on the next page

143D_syn-loop-pull-box150Å_run1 (part 2)

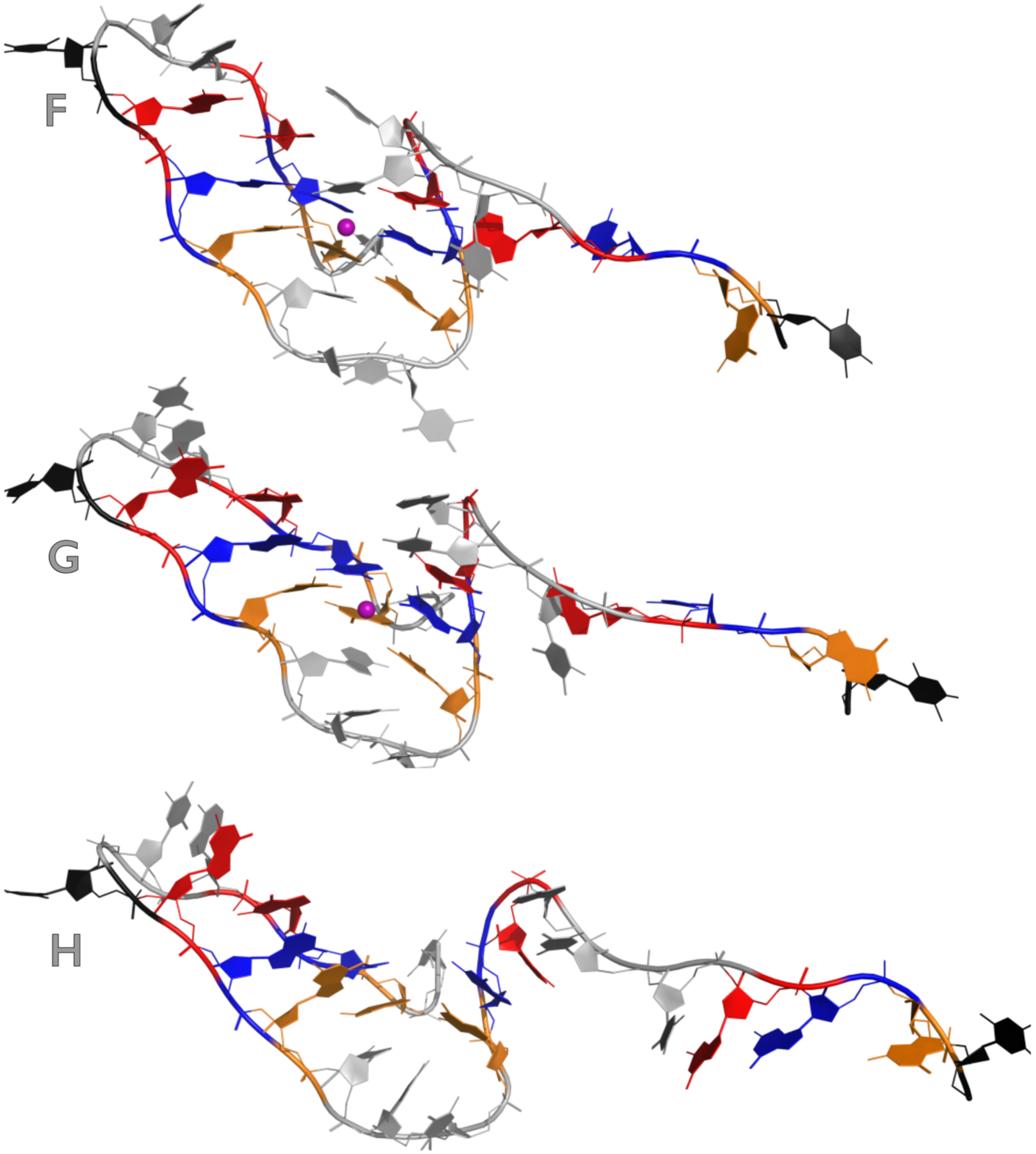

Figure S37B: Most important structural events during first independent *fast pulling* simulation of 143D$_{syn_\text{loop-pull-box150Å}}$ GQ system. See legend of Figure S1B for more details.

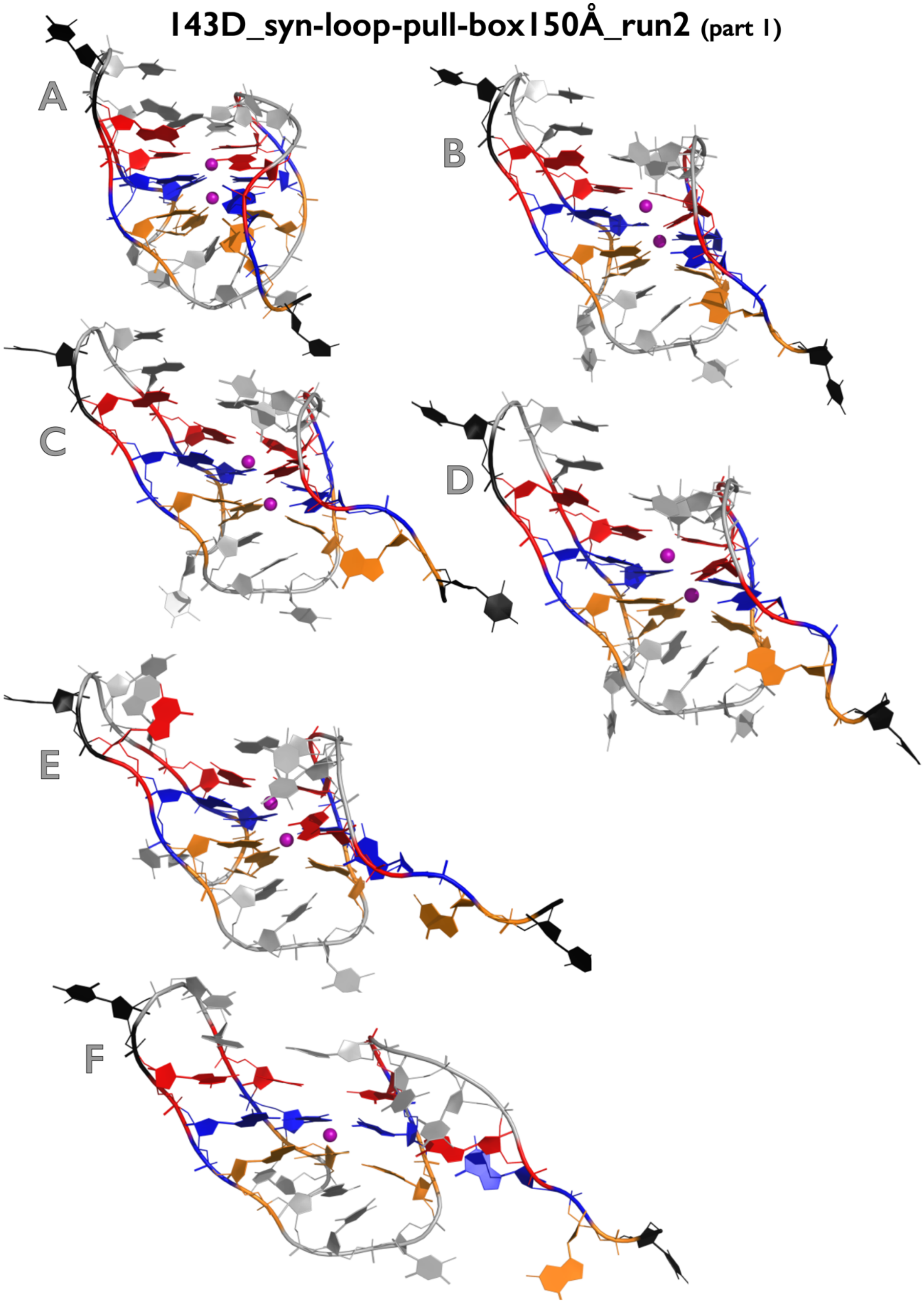

Figure continuing on the next page

143D_syn-loop-pull-box150Å_run2 (part 2)

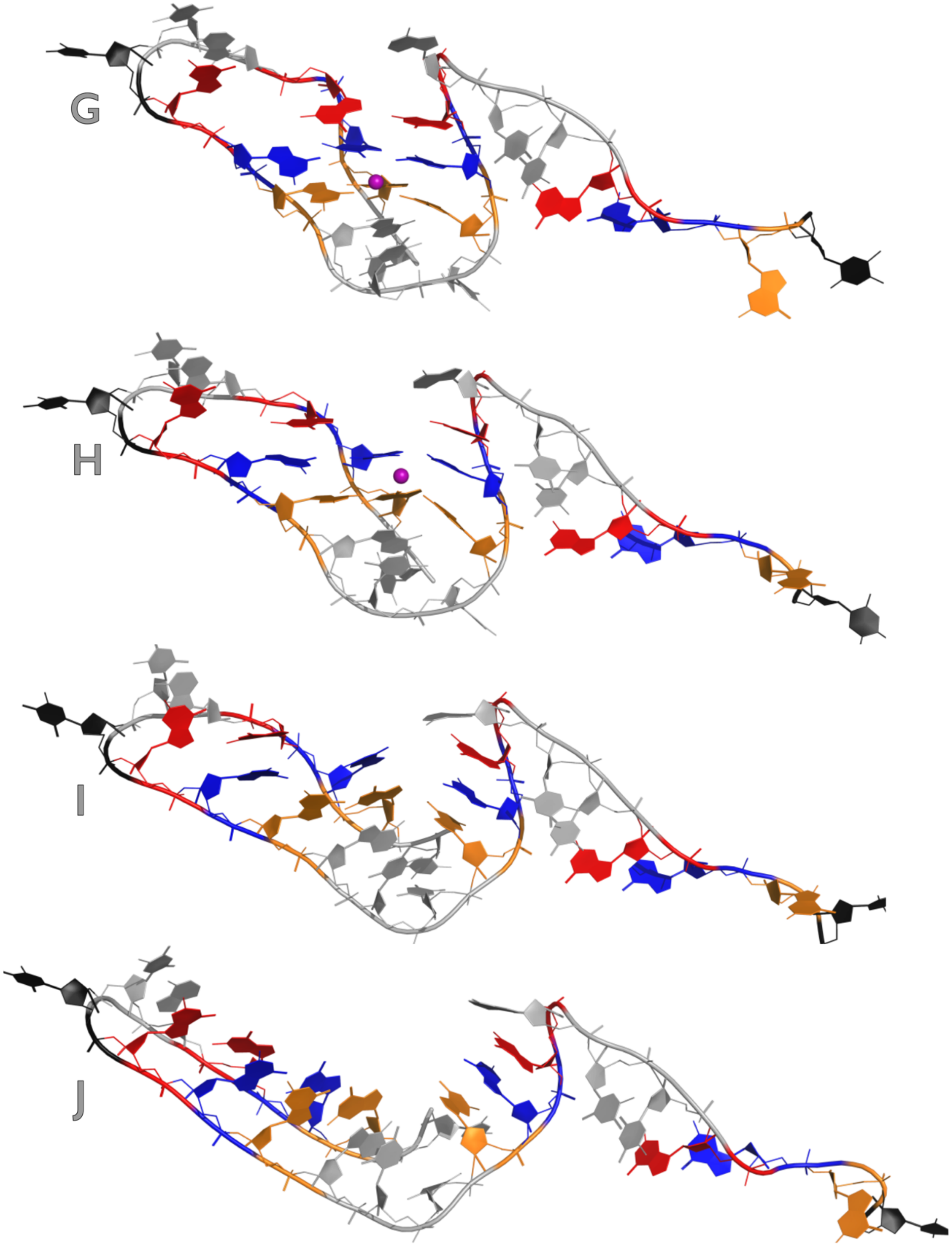

Figure S37C: Most important structural events during second independent *fast pulling* simulation of 143D$_{syn_\mathrm{loop\text{-}pull\text{-}box150Å}}$ GQ system. See legend of Figure S1B for more details.

143D_syn-loop-pull-box150Å_run3 (part 1)

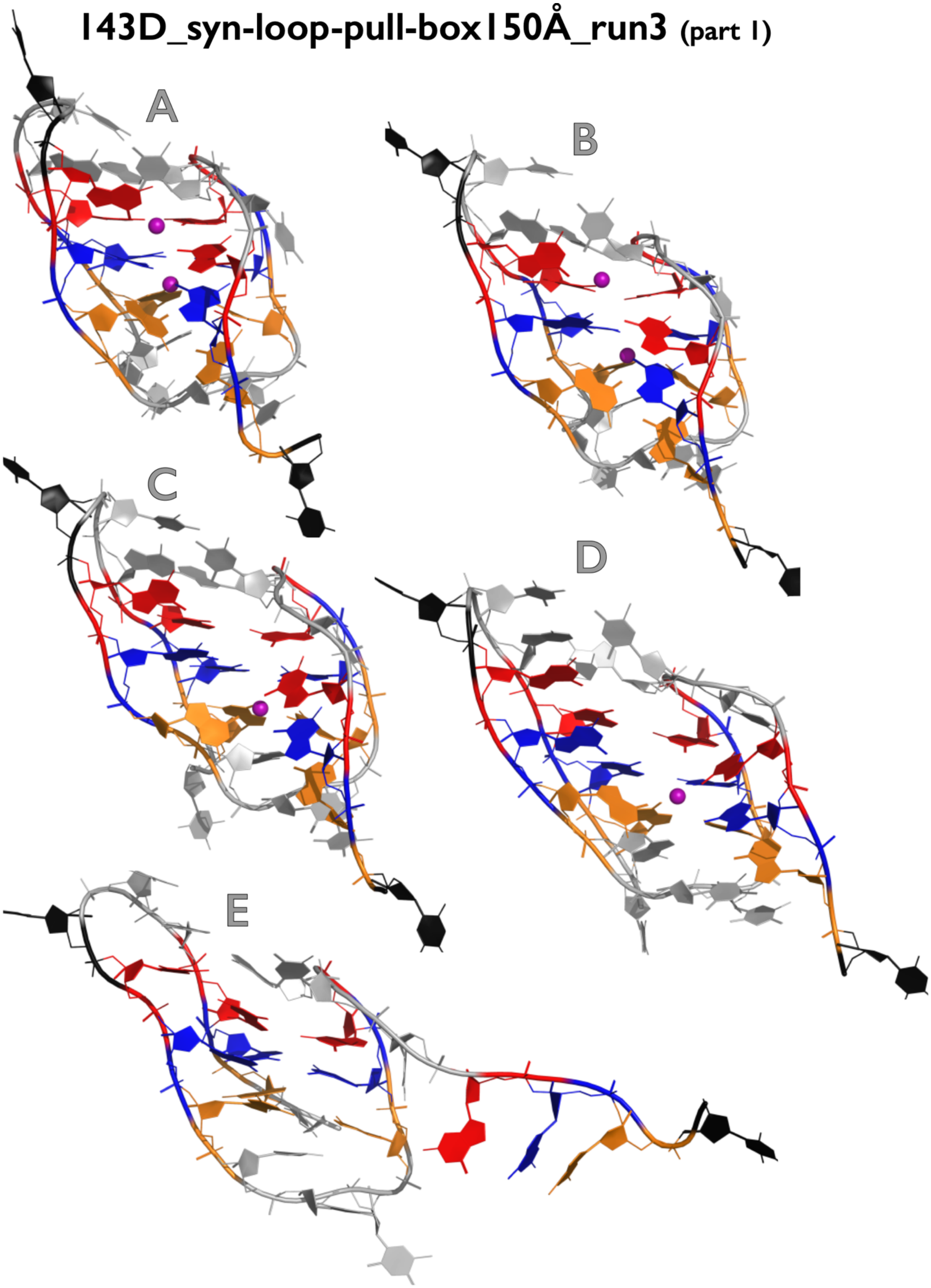

Figure continuing on the next page

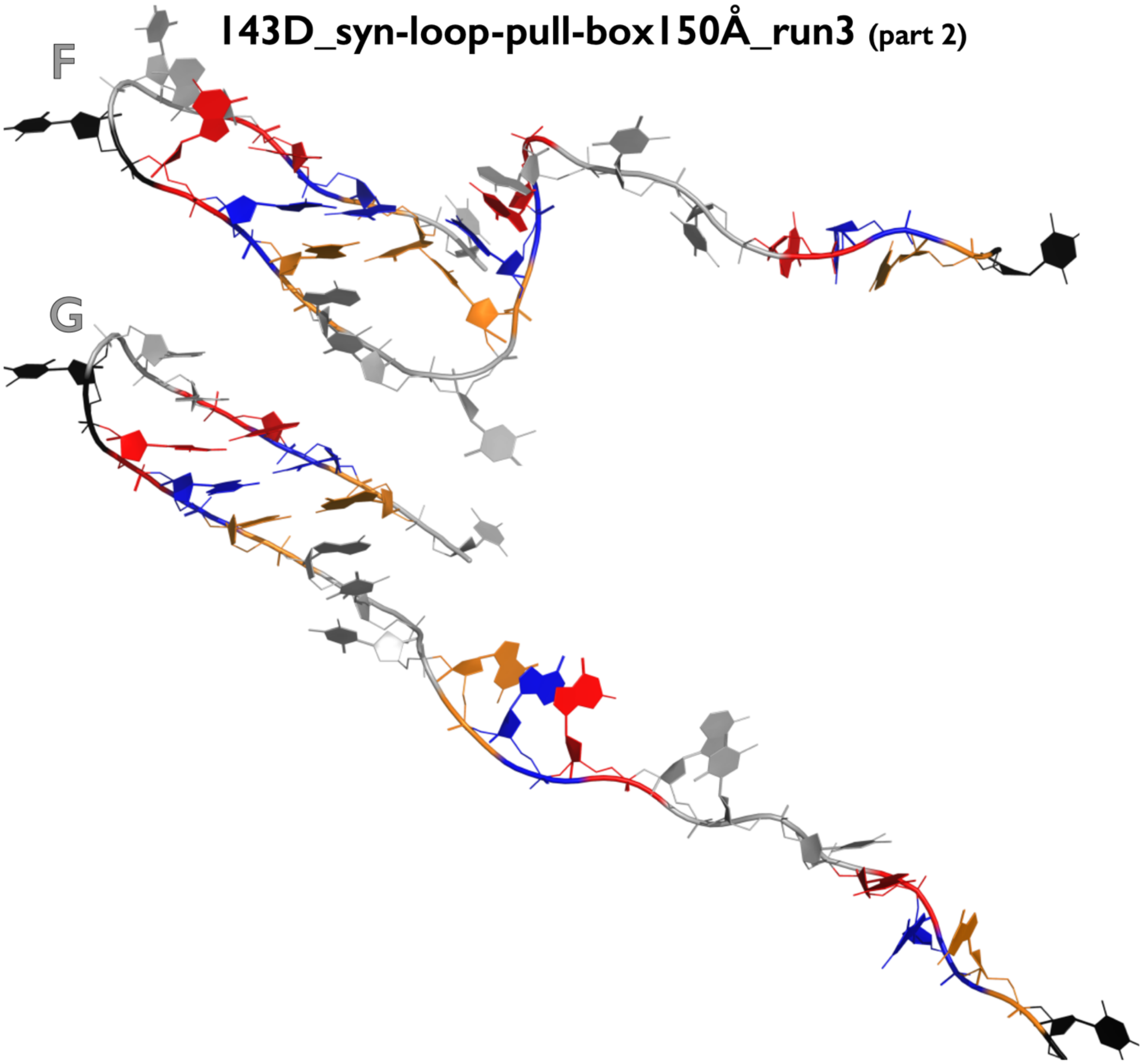

Figure S37D: Most important structural events during third independent *fast pulling* simulation of 143D$_{syn_loop\text{-}pull\text{-}box150Å}$ GQ system. See legend of Figure S1B for more details.

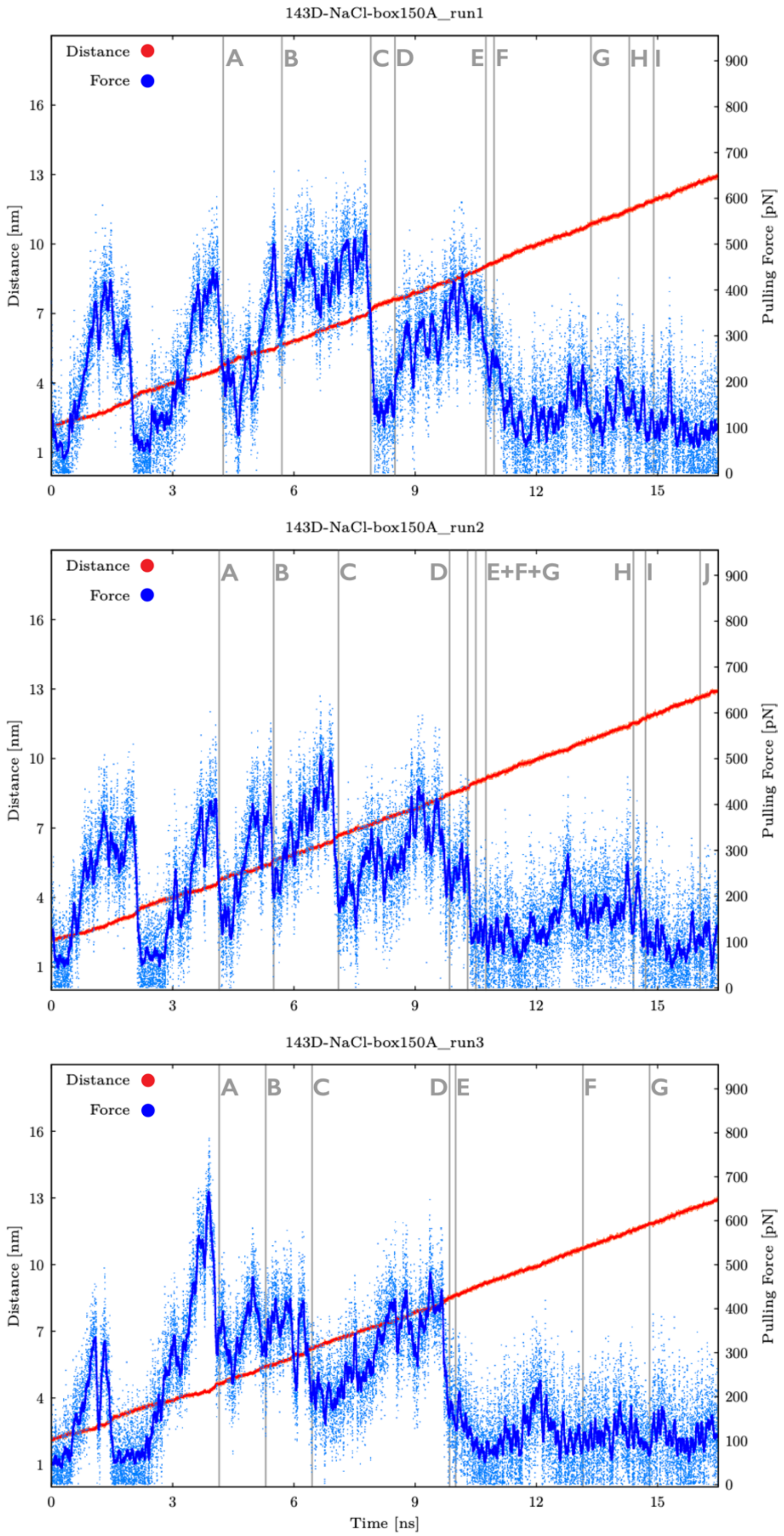

Figure S38A: Time evolution of distance between pulling centers and pulling force during three independent *fast pulling* simulations of 143D$_{\text{NaCl-box150Å}}$ GQ system (see legend of Figure S1A for more details). See Figure S38B-S38D for inspection of structures corresponding to main structural events.

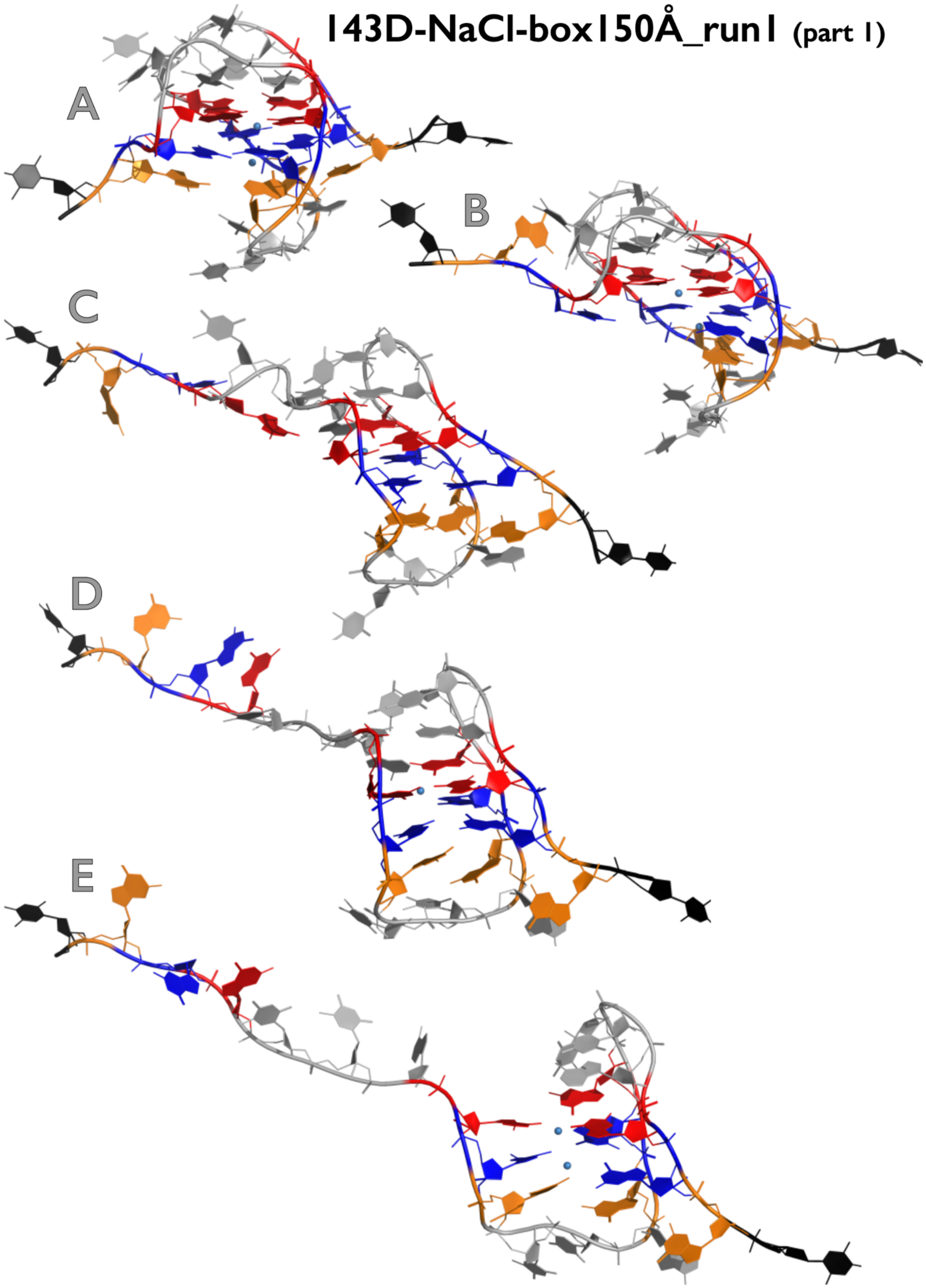

Figure continuing on the next page

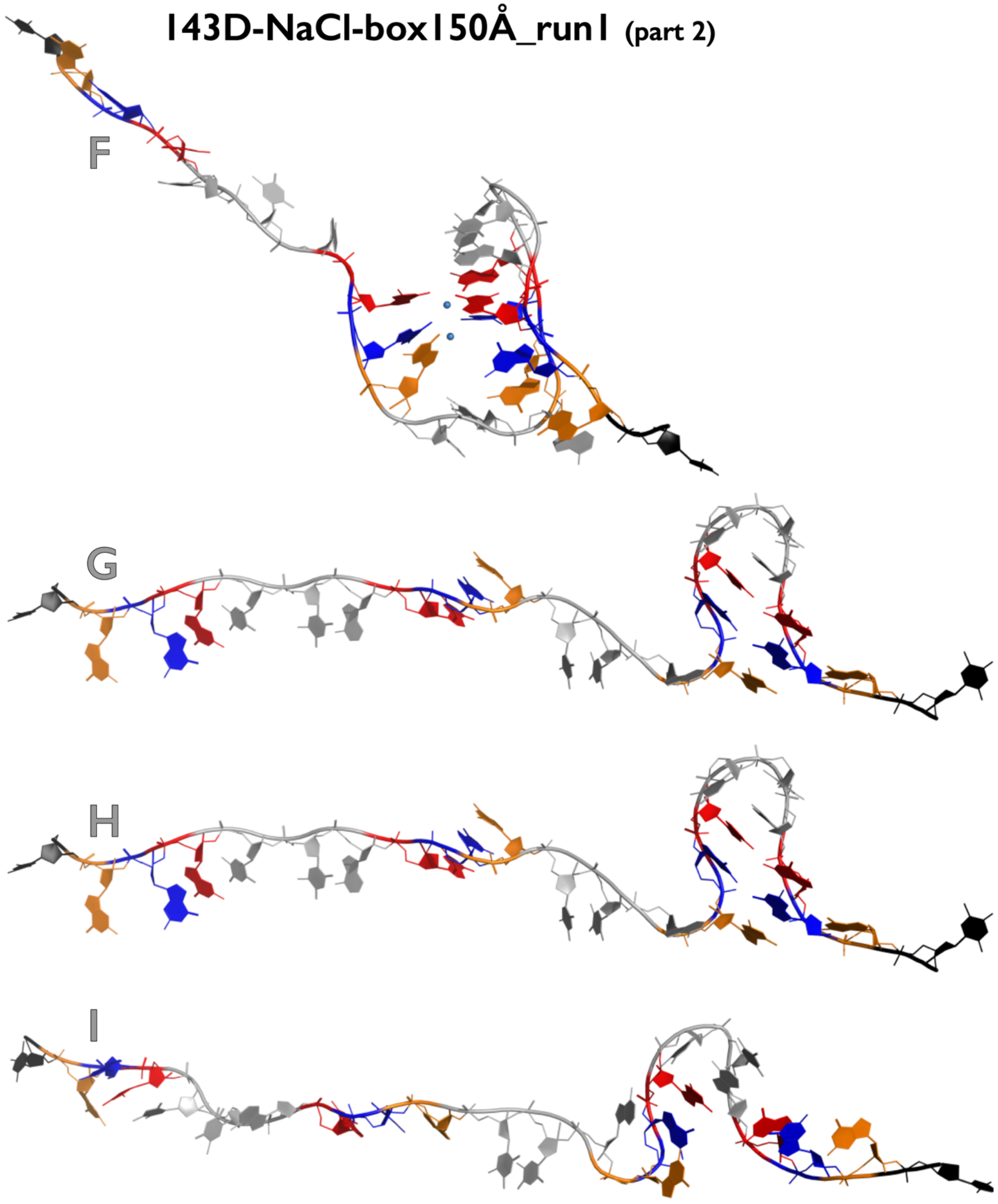

Figure S38B: Most important structural events during first independent *fast pulling* simulation of 143D$_{NaCl\text{-}box150Å}$ GQ system. See legend of Figure S28B for more details.

143D-NaCl-box150Å_run2 (part 1)

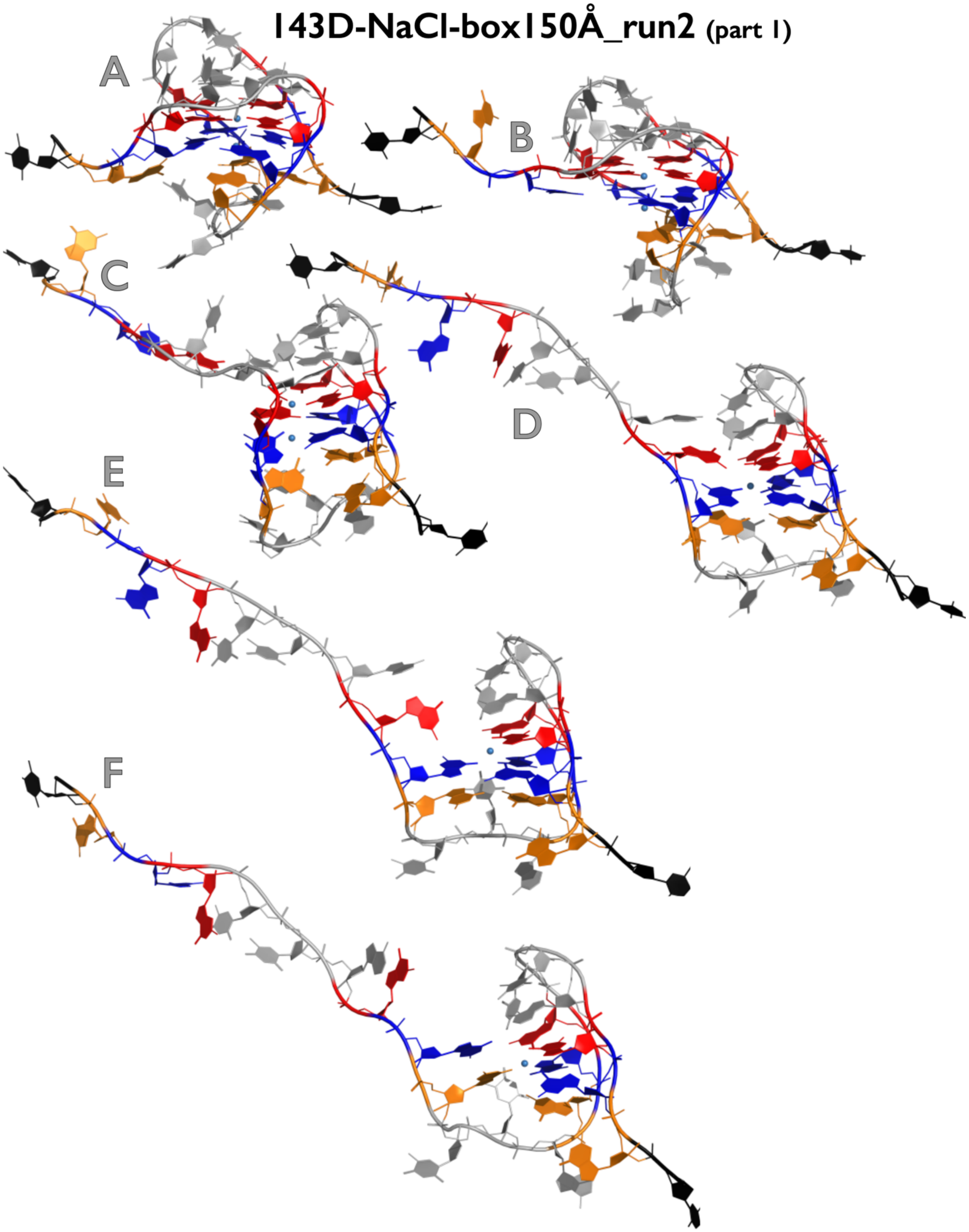

Figure continuing on the next page

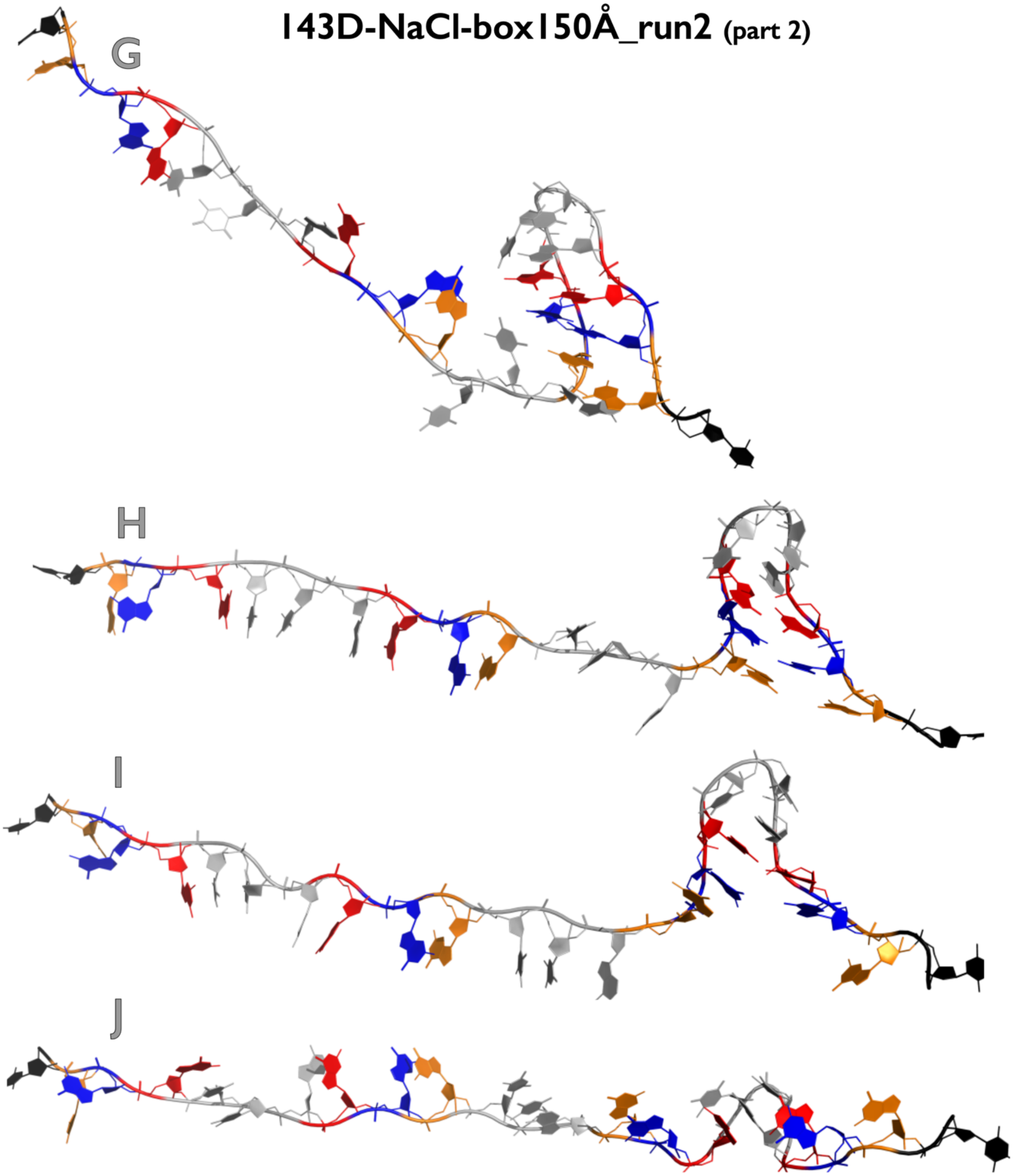

Figure S38C: Most important structural events during second independent *fast pulling* simulation of 143D$_{NaCl\text{-}box150Å}$ GQ system. See legend of Figure S28B for more details.

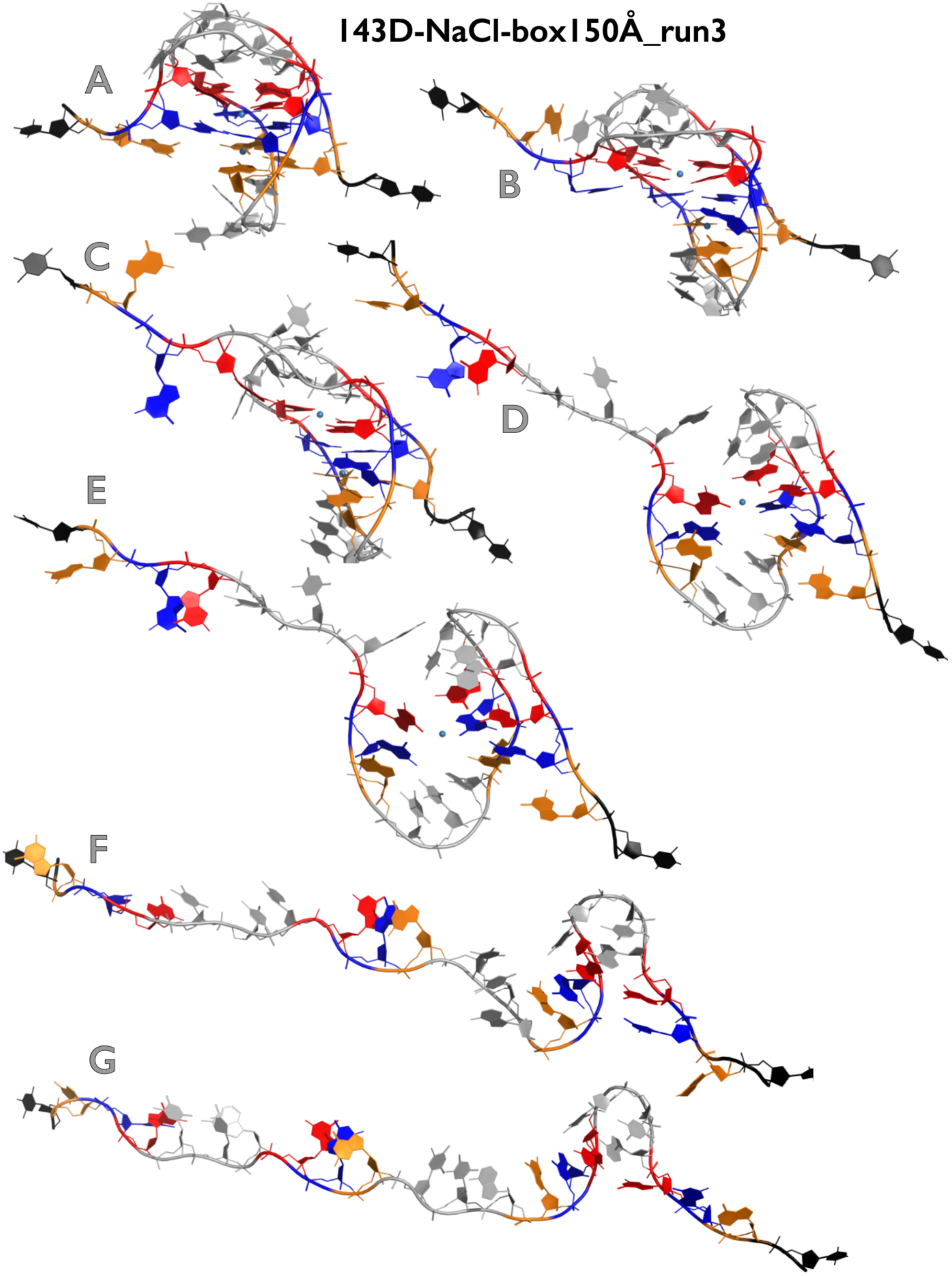

Figure S38D: Most important structural events during third independent *fast pulling* simulation of 143D$_{\text{NaCl-box150Å}}$ GQ system. See legend of Figure S28B for more details.

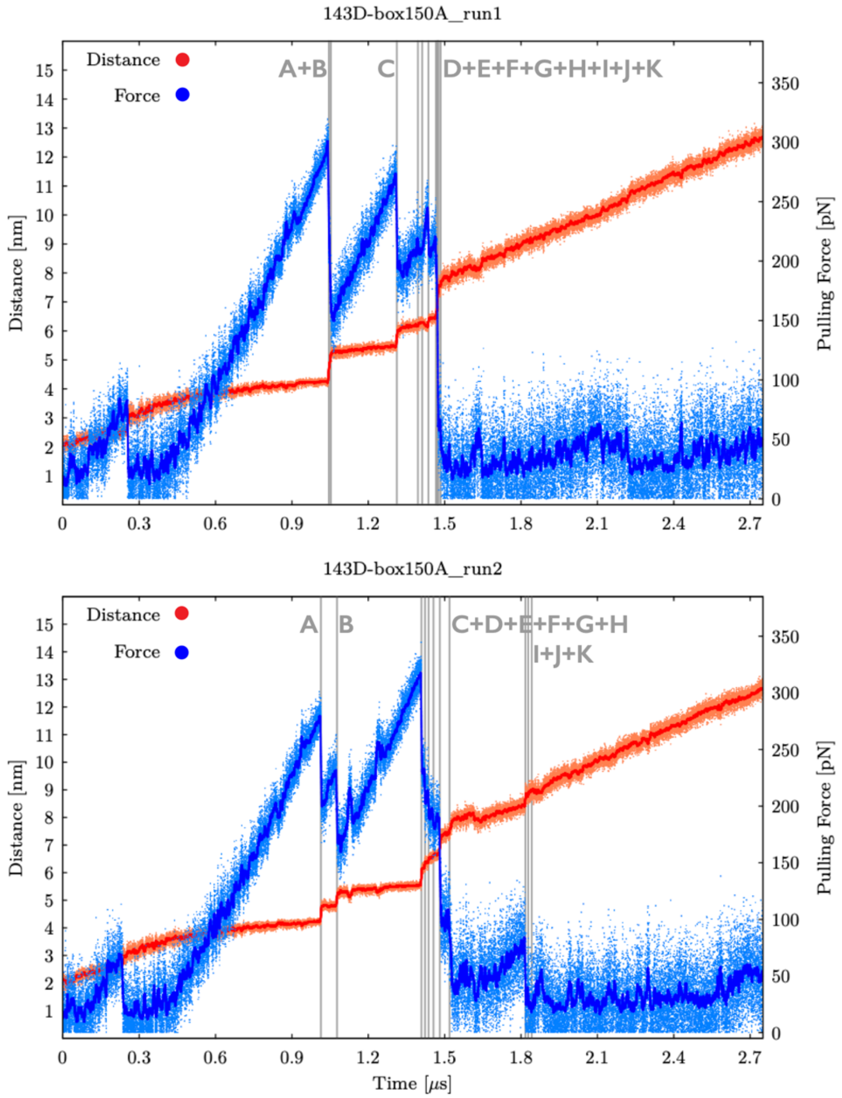

Figure S39A: Time evolution of distance between pulling centers and pulling force during two independent *very slow pulling* simulations of 143D$_{box150Å}$ GQ system (see legend of Figure S25A for more details). See Figures S39B and S39C for inspection of structures corresponding to main structural events.

143D-box150Å_run1 (part1)

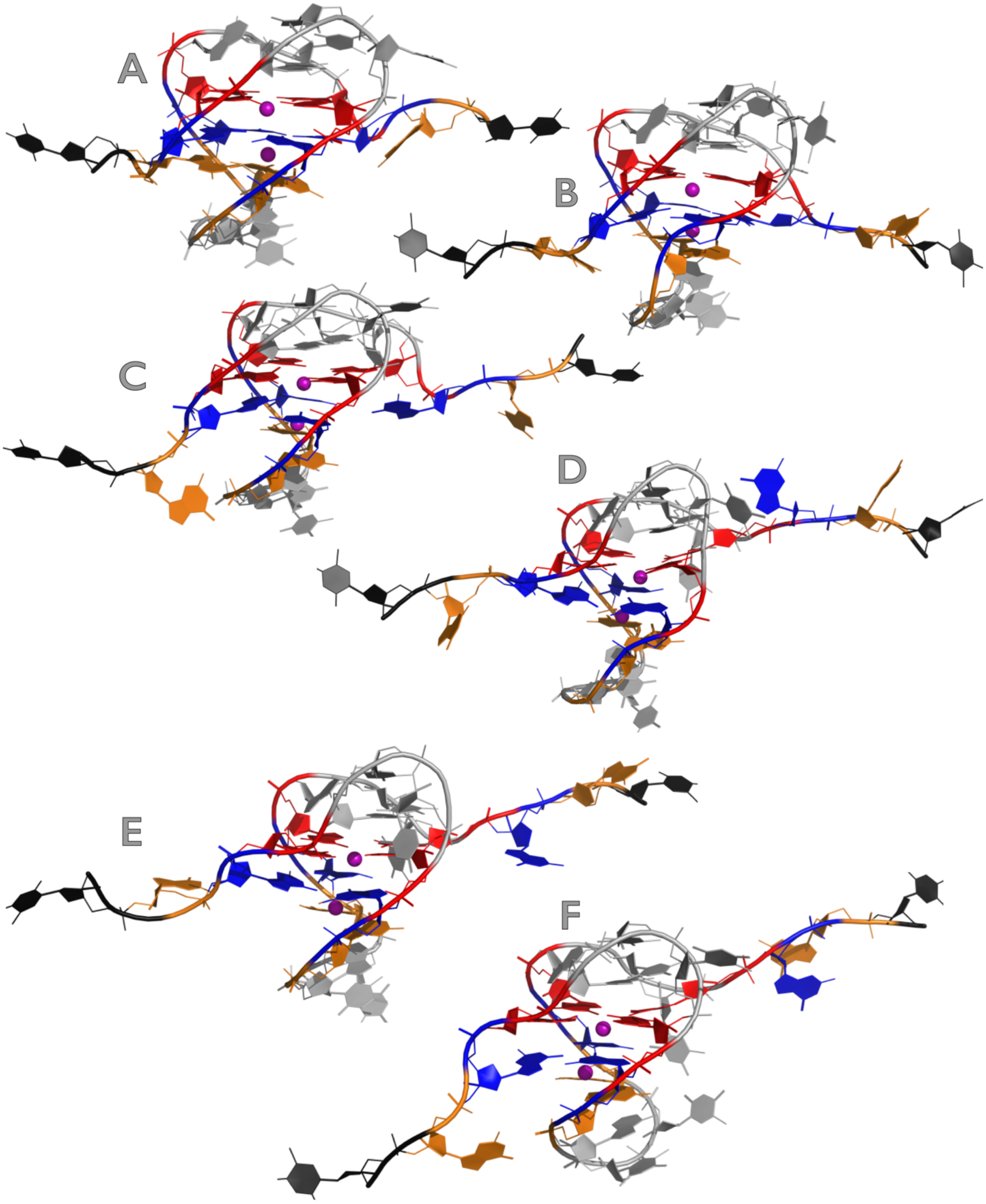

Figure continuing on the next page

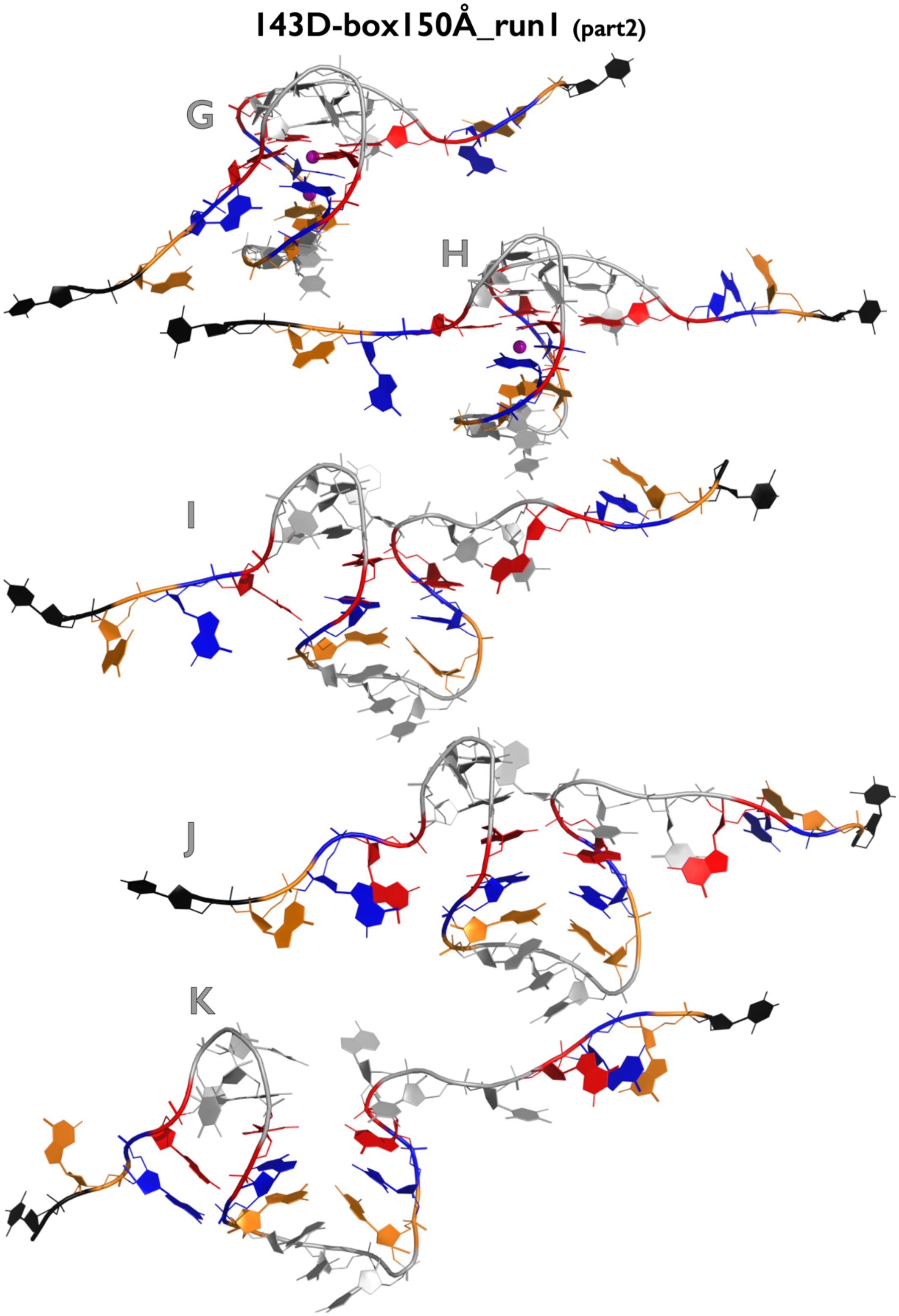

Figure S39B: Most important structural events during first independent *very slow pulling* simulation of 143D$_{box150Å}$ GQ system. See legend of Figure S1B for more details.

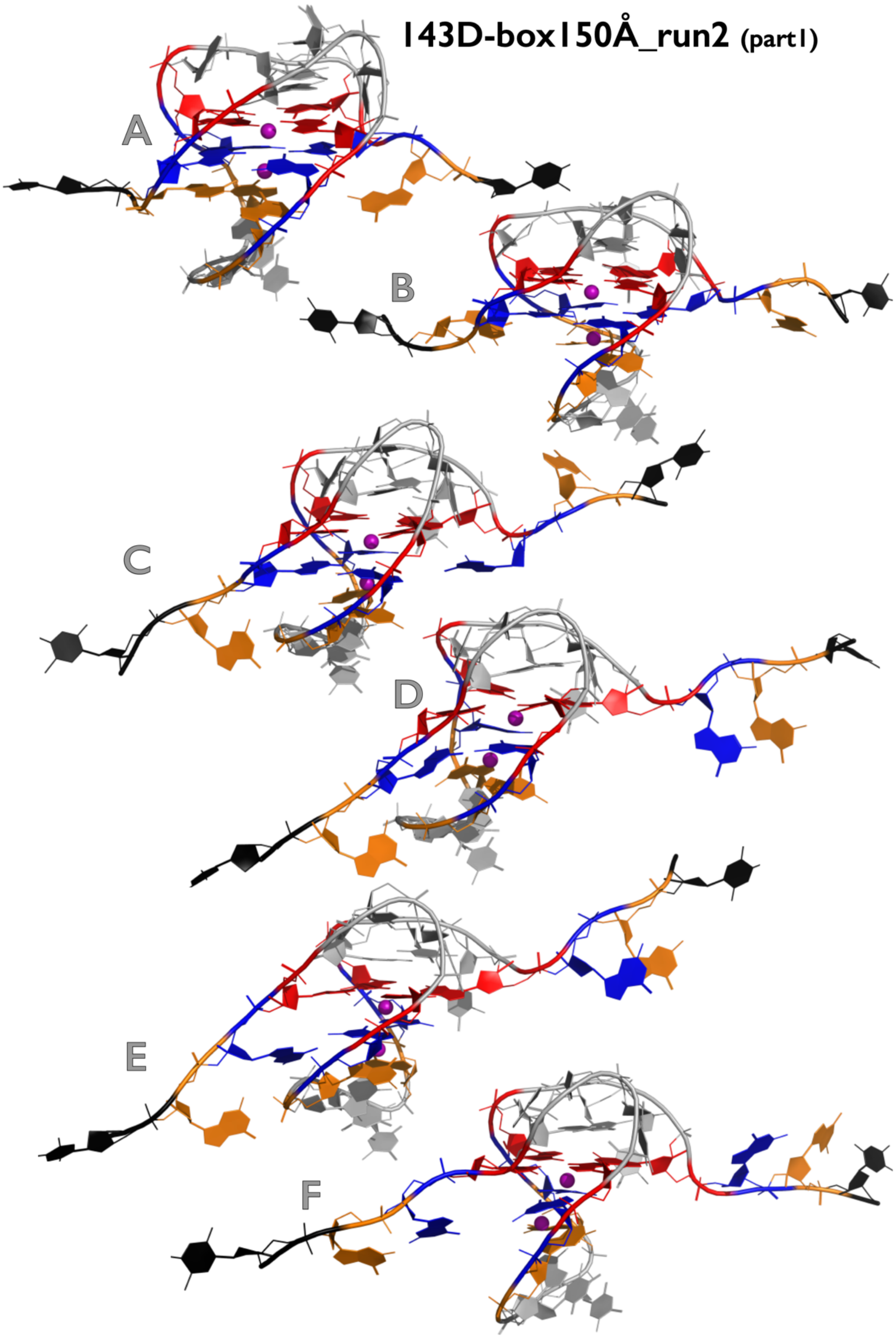

Figure continuing on the next page

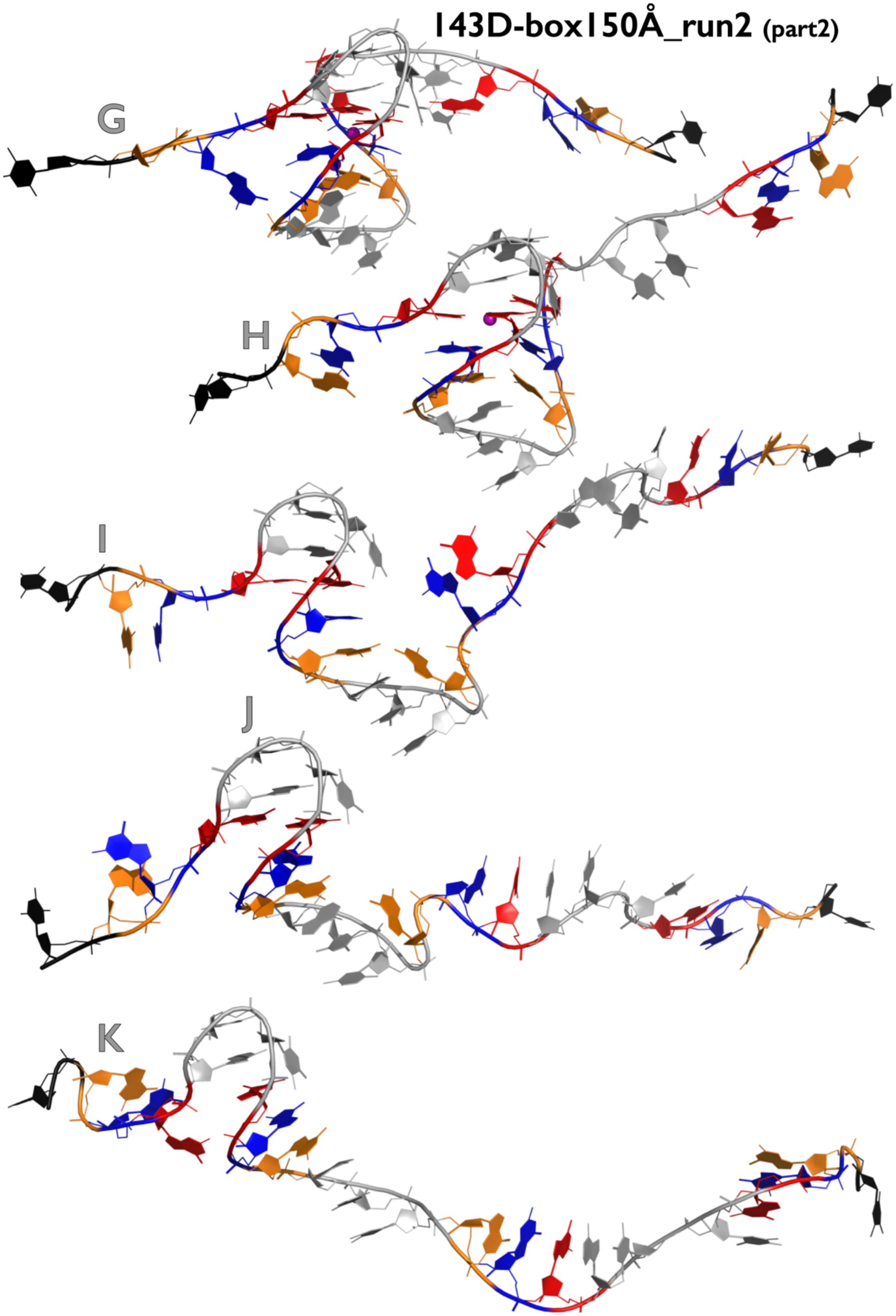

Figure S39C: Most important structural events during second independent *very slow pulling* simulation of 143D$_{box150Å}$ GQ system. See legend of Figure S1B for more details.

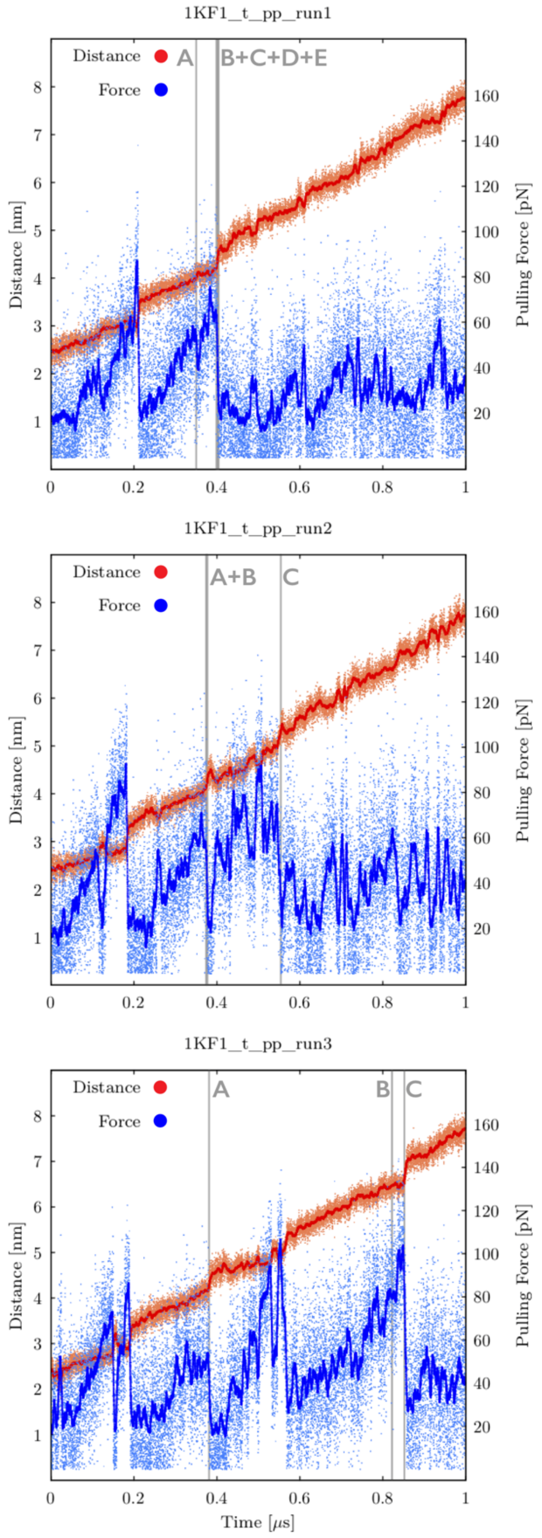

Figure S40A: Time evolution of distance between pulling centers and pulling force during three independent *very slow pulling* simulations of 1KF1_t_pp G-triplex system. Snapshots were saved every 50 ps and plots are showing both instantaneous values (orange and light-blue dots for distance and force, respectively) and smoothing, i.e., averaging over 100 consecutive snapshots (red and blue lines for distance and force, respectively). Main structural events are highlighted as

grey vertical lines with labels (capital letters). See Figures S40B and S40C for inspection of structures corresponding to main structural events. Note that first major drops of the pulling force before the unfolding event "A" (and notable prolongation of end-to-end distances) are connected with repositioning of terminal T residues.

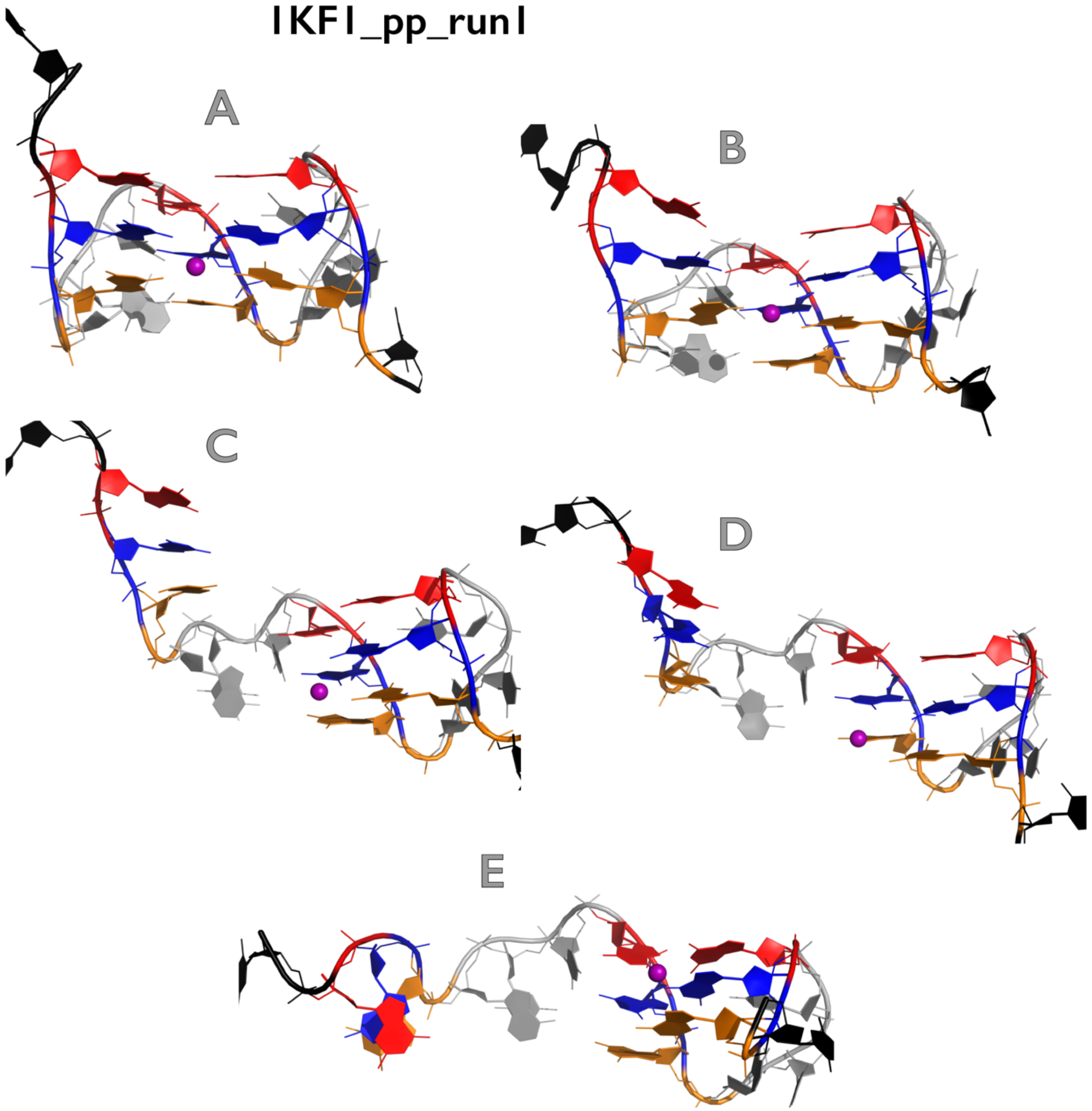

Figure S40B: Most important structural events during first independent *very slow pulling* simulation of 1KF1_t_pp G-triplex system. See legend of Figure S1B for more details.

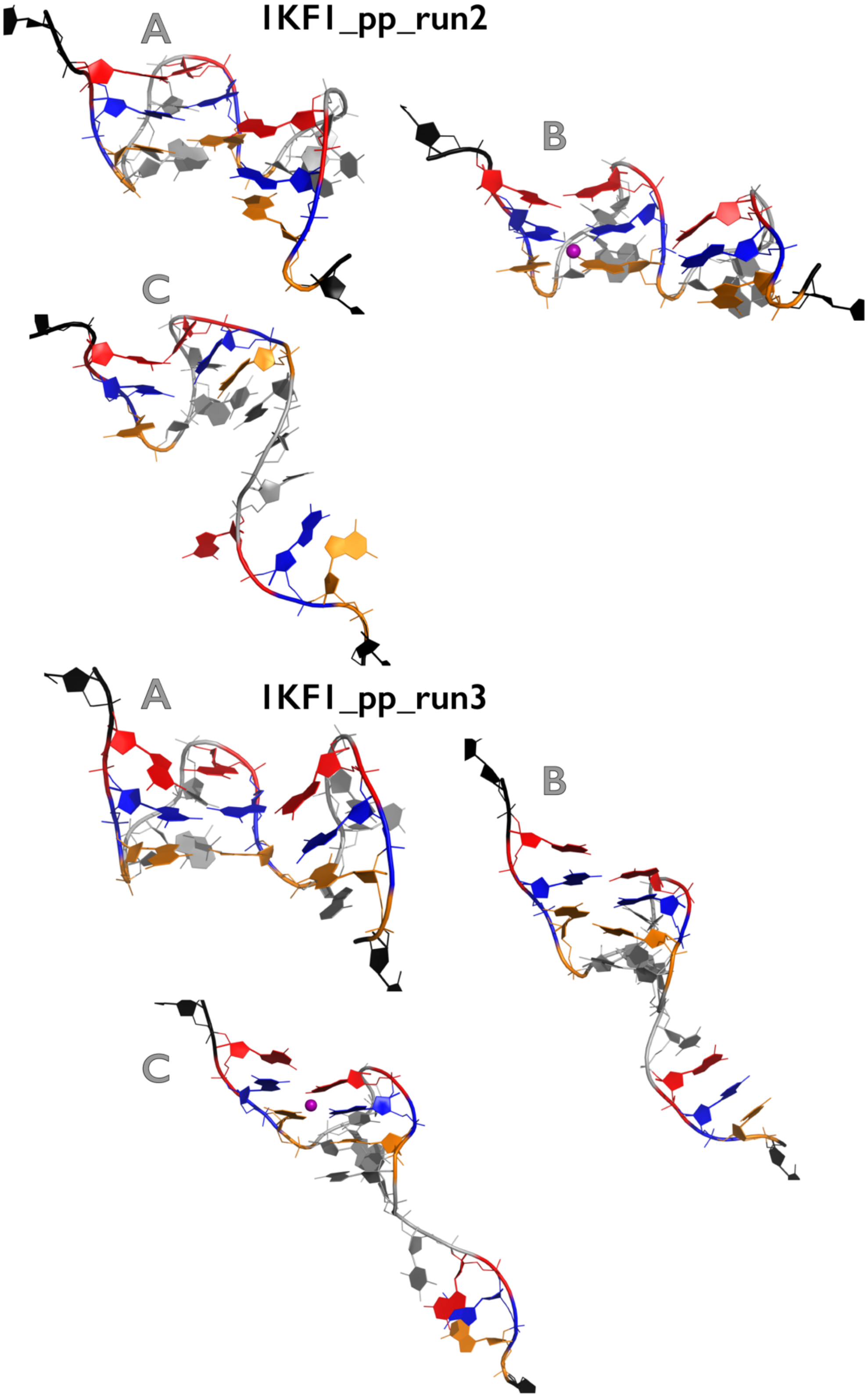

Figure S40C: Most important structural events during second and third independent *very slow pulling* simulation of 1KF1_t_pp G-triplex system. See legend of Figure S1B for more details.

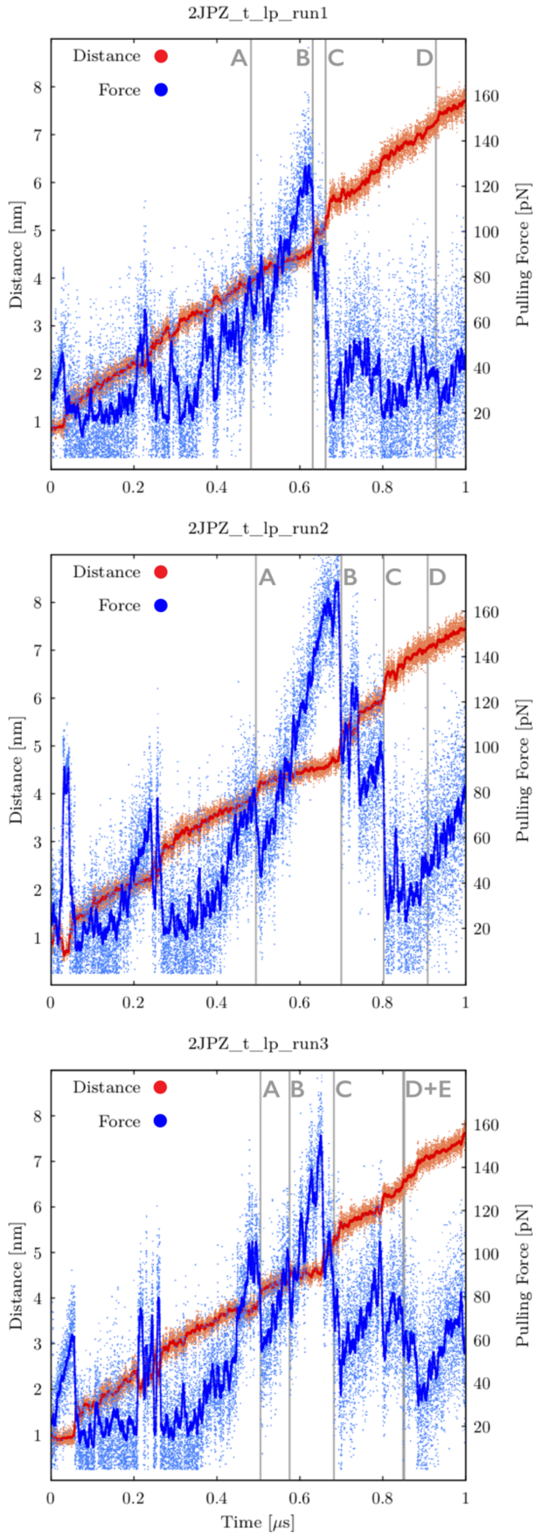

Figure S41A: Time evolution of distance between pulling centers and pulling force during three independent *very slow pulling* simulations of 2JPZ_t_l$_{n}$p G-triplex system (see legend of Figure S40A for more details). See Figures S41B-S41D for inspection of structures corresponding to main structural events.

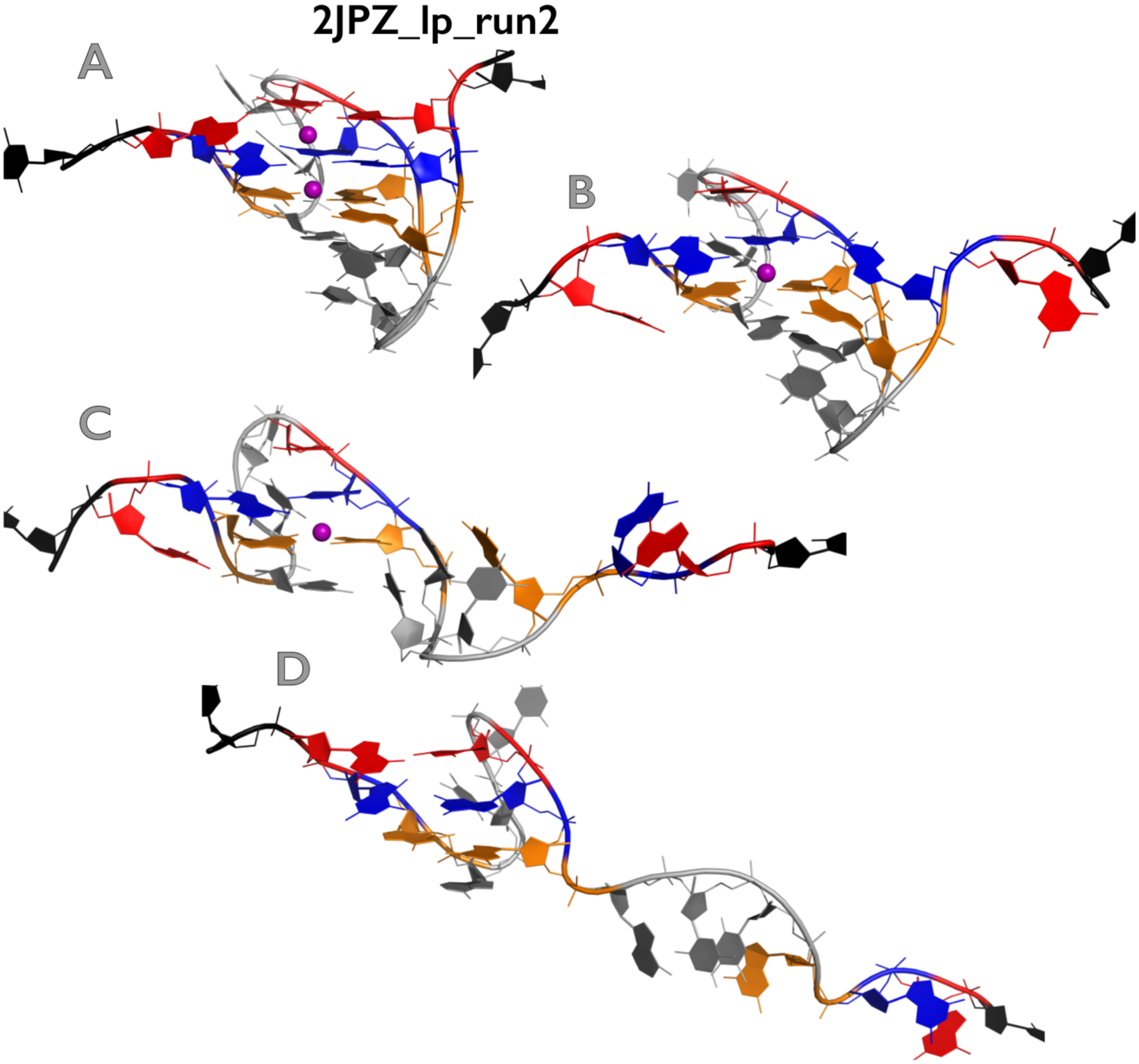

Figure S41B: Most important structural events during first independent *very slow pulling* simulation of 2JPZ_t_l_np G-triplex system. See legend of Figure S1B for more details.

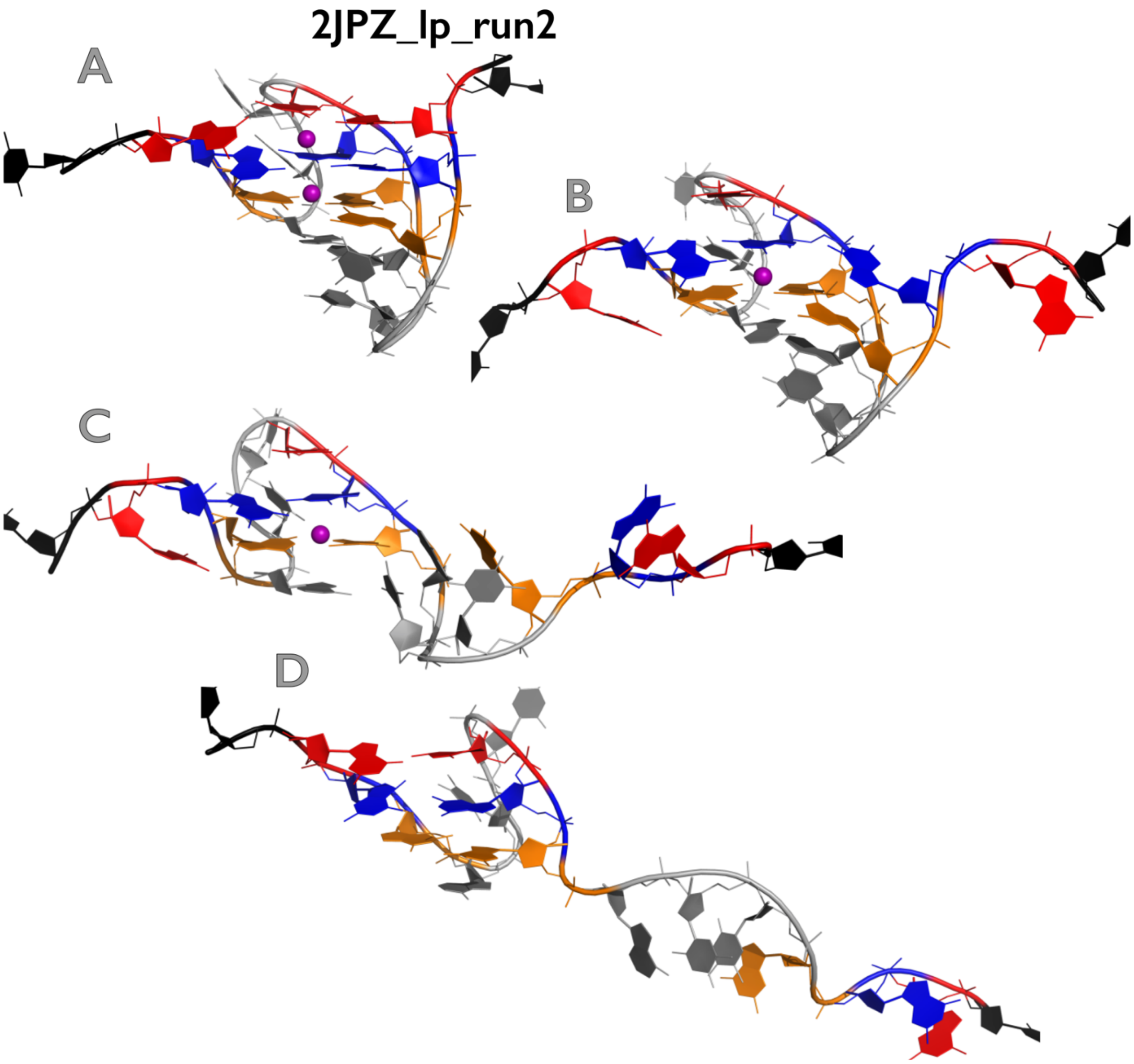

Figure S41C: Most important structural events during second independent *very slow pulling* simulation of 2JPZ_t_l$_n$p G-triplex system. See legend of Figure S1B for more details.

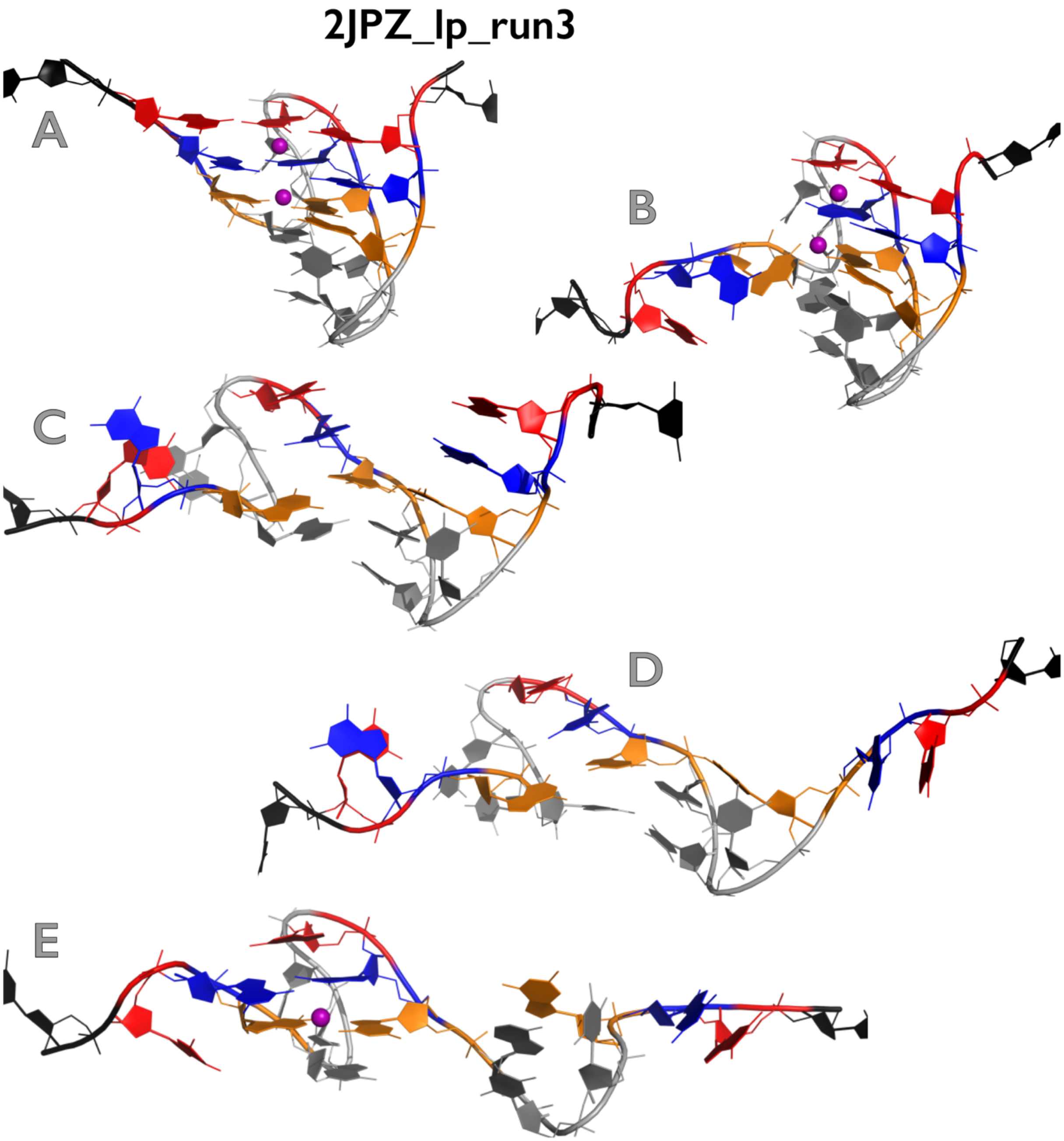

Figure S41D: Most important structural events during third independent *very slow pulling* simulation of 2JPZ_t_l_np G-triplex system. See legend of Figure S1B for more details.

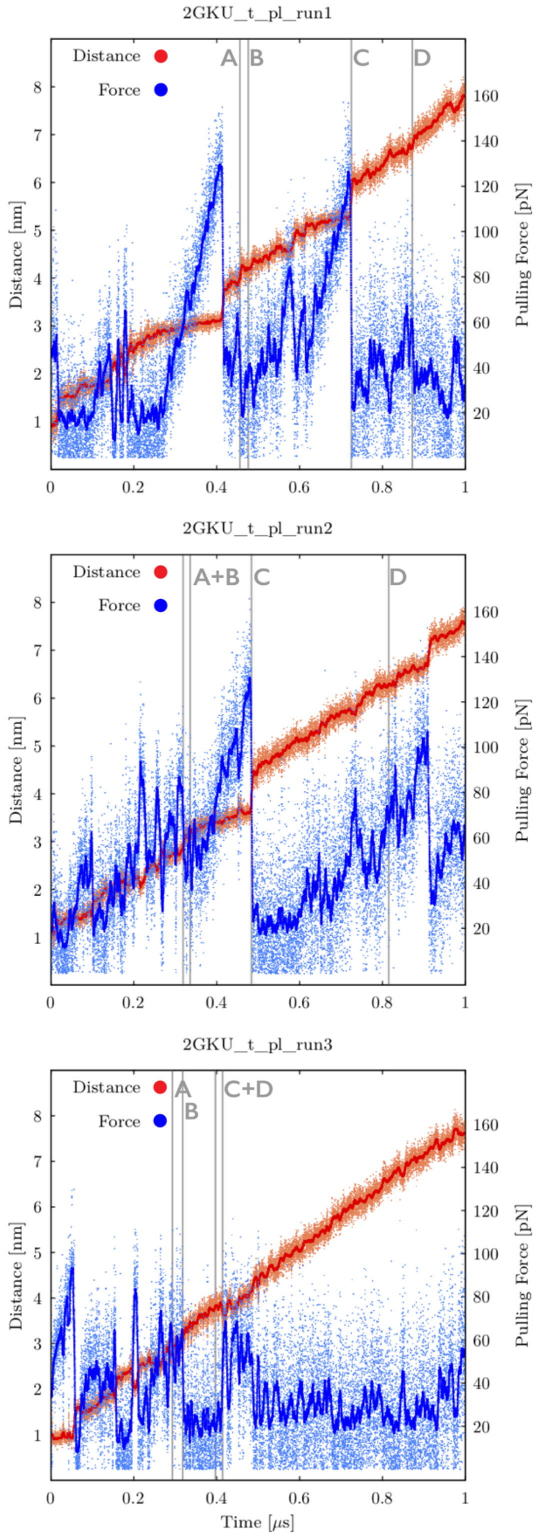

Figure S42A: Time evolution of distance between pulling centers and pulling force during three independent *very slow pulling* simulations of 2GKU_t_pl$_w$ G-triplex system (see legend of Figure S40A for more details). See Figures S42B-S42D for inspection of structures corresponding to main structural events.

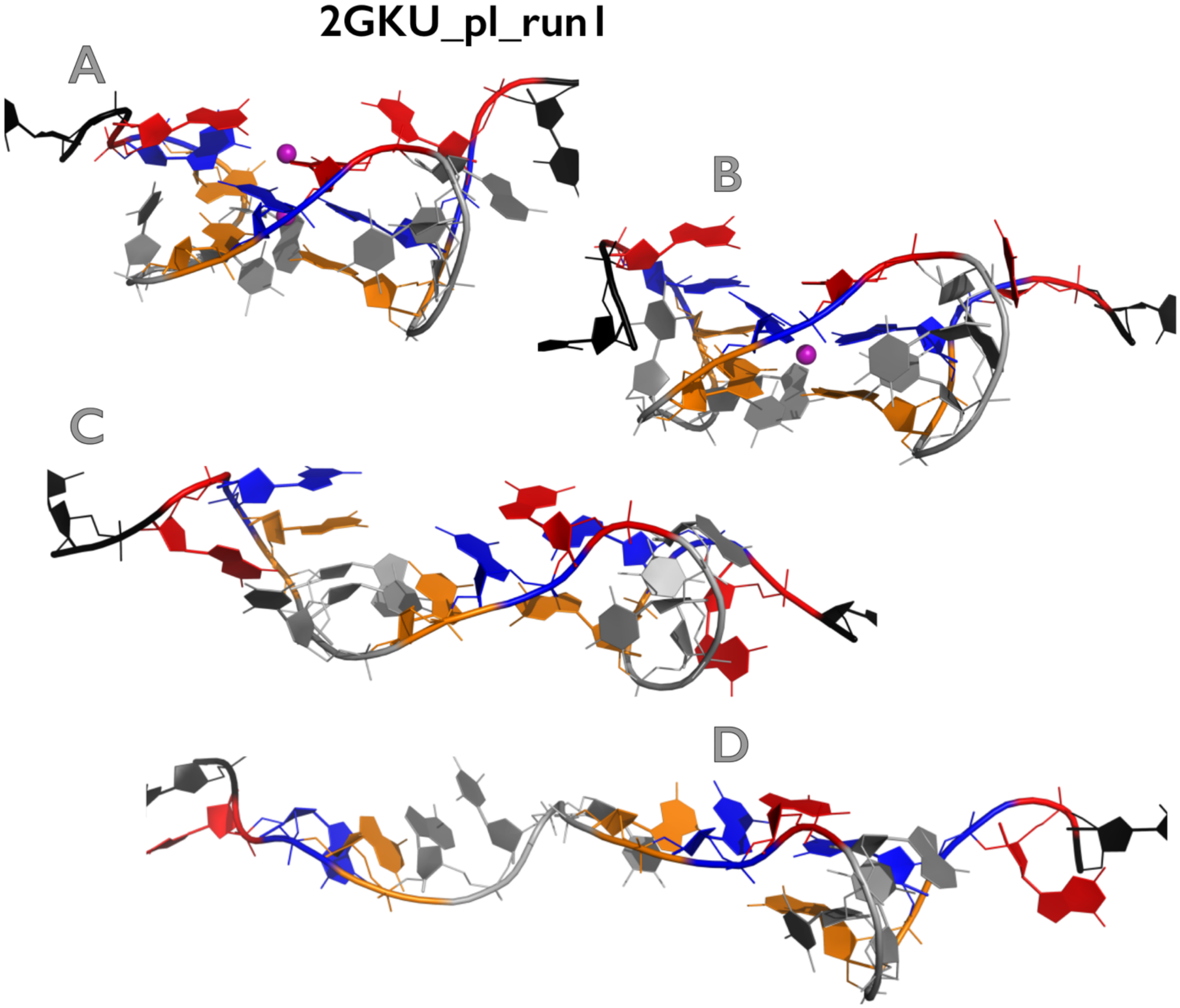

Figure S42B: Most important structural events during first independent *very slow pulling* simulation of 2GKU_t_pl$_w$ G-triplex system. See legend of Figure S1B for more details.

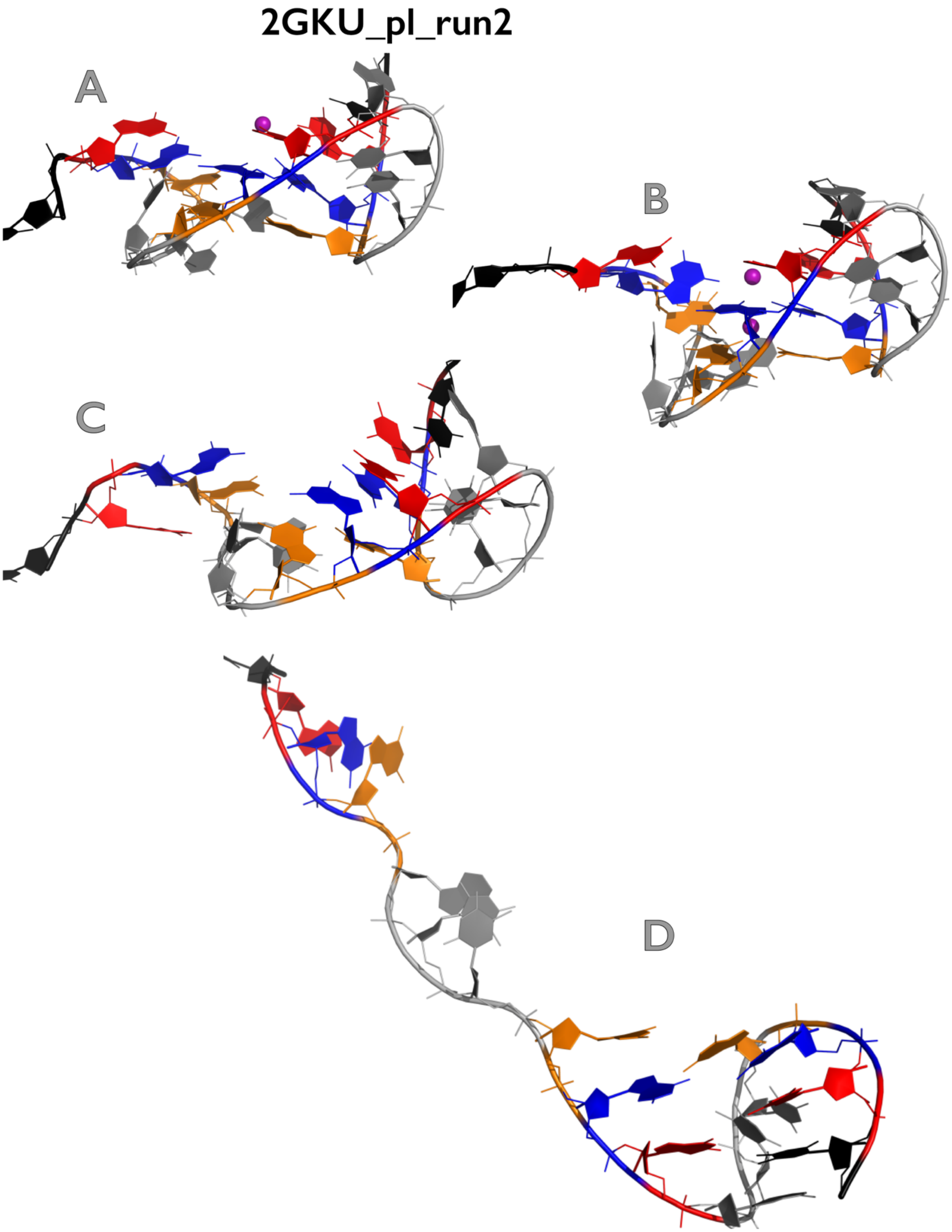

Figure S42C: Most important structural events during second independent *very slow pulling* simulation of 2GKU_t_pl$_w$ G-triplex system. See legend of Figure S1B for more details.

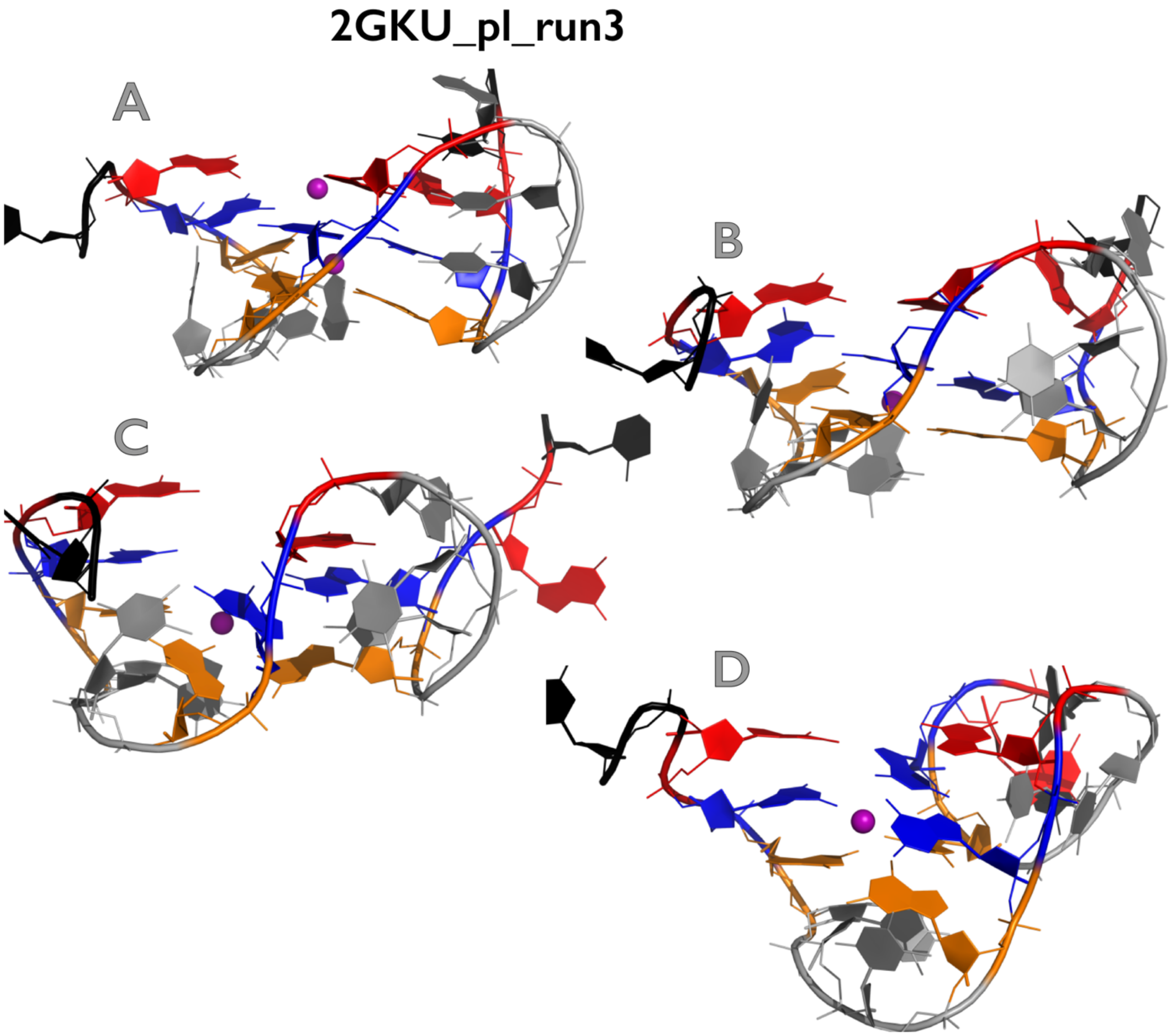

Figure S42D: Most important structural events during third independent *very slow pulling* simulation of 2GKU_t_pl$_w$ G-triplex system. See legend of Figure S1B for more details.

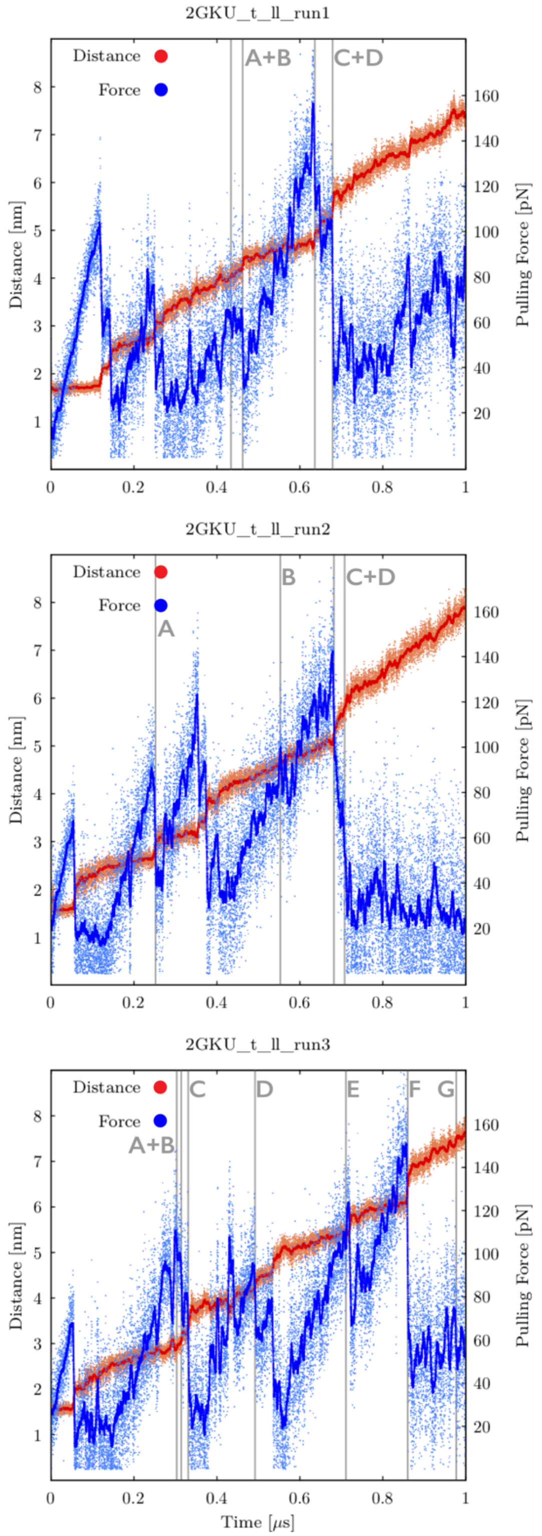

Figure S43A: Time evolution of distance between pulling centers and pulling force during three independent *very slow pulling* simulations of 2GKU_t_l$_w$l$_n$ G-triplex system (see legend of Figure S40A for more details). See Figures S43B-S43D for inspection of structures corresponding to main structural events.

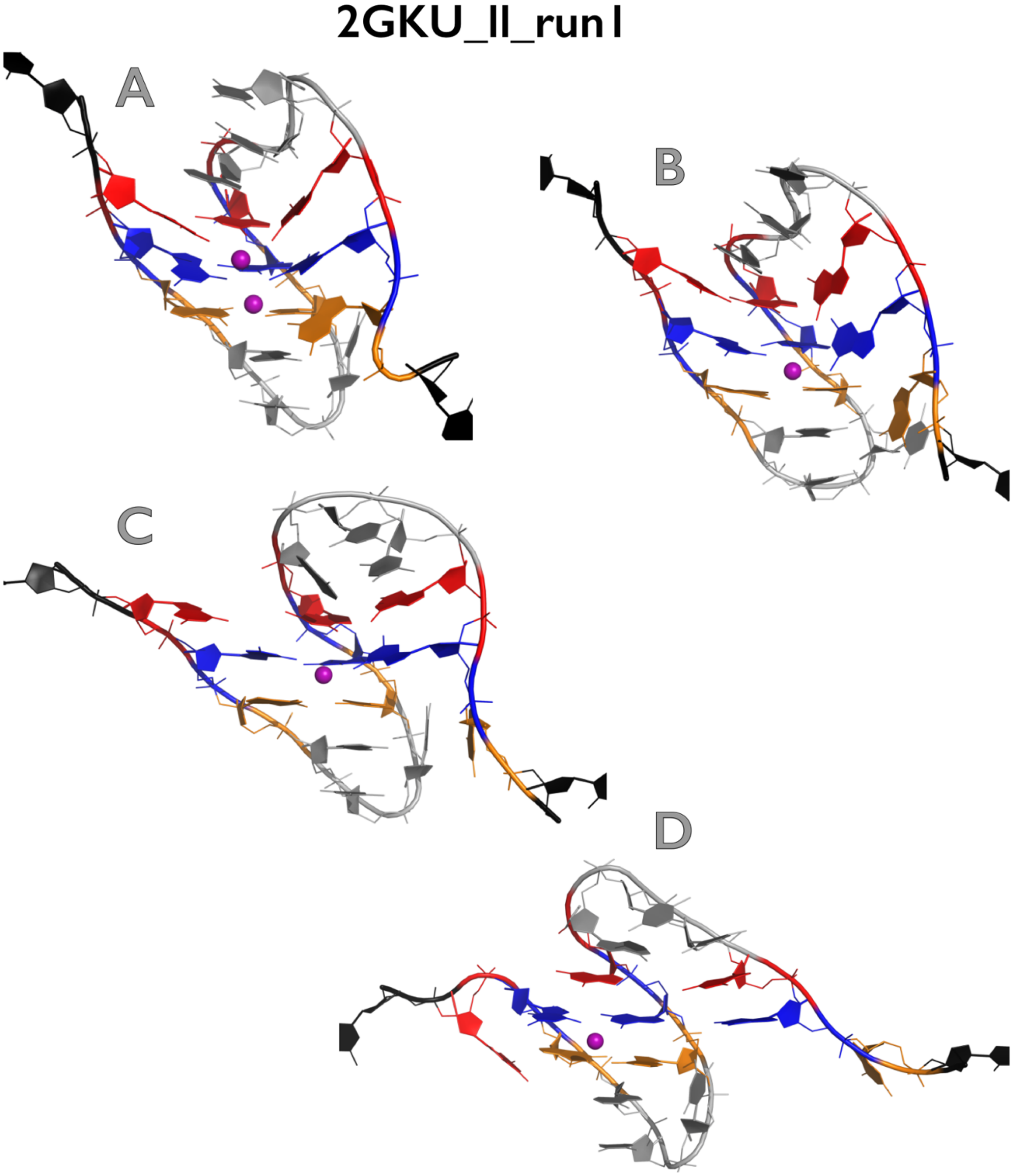

Figure S43B: Most important structural events during first independent *very slow pulling* simulation of 2GKU_t_$l_w l_n$ G-triplex system. See legend of Figure S1B for more details.

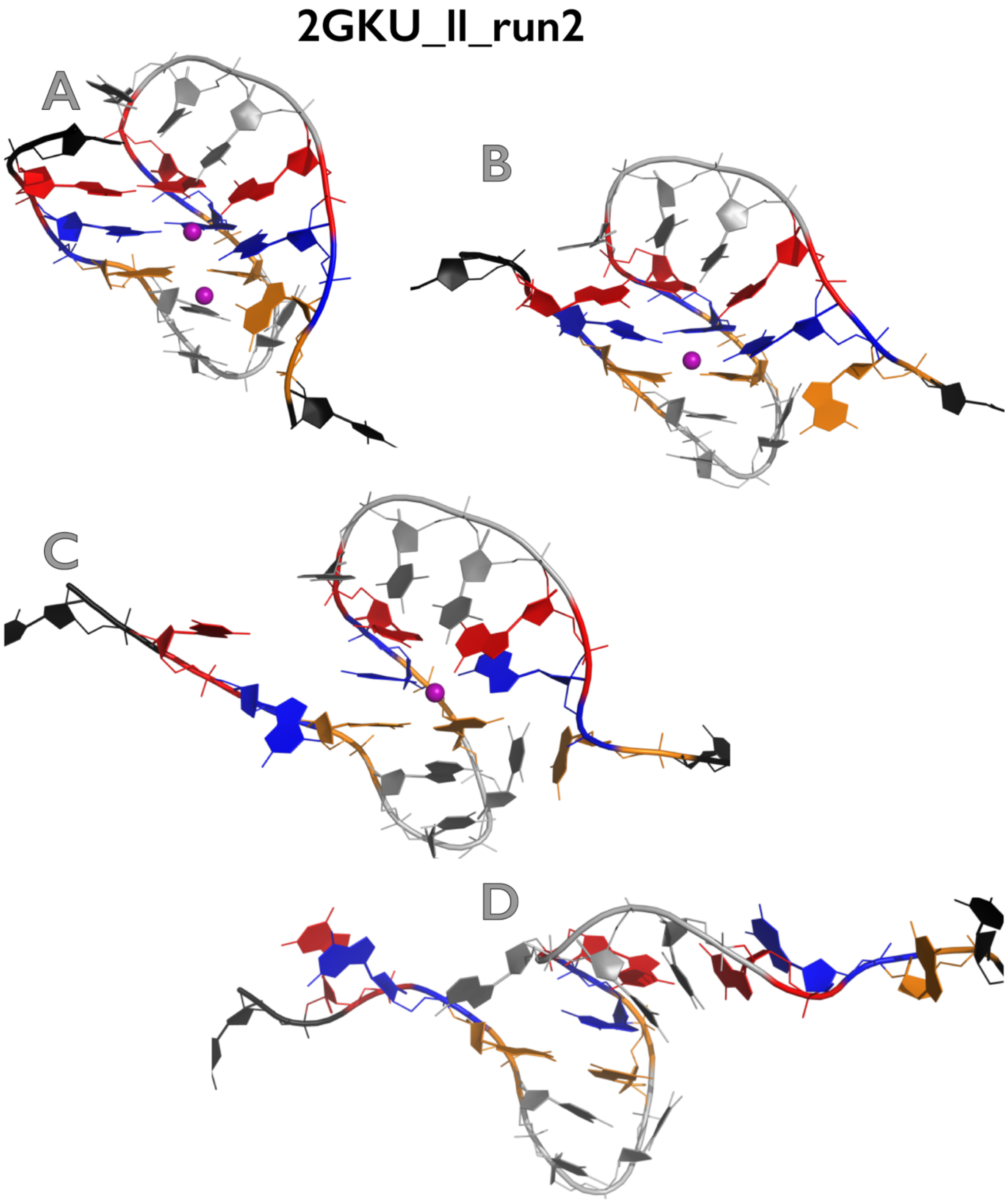

Figure S43C: Most important structural events during second independent *very slow pulling* simulation of 2GKU_t_$l_w l_n$ G-triplex system. See legend of Figure S1B for more details.

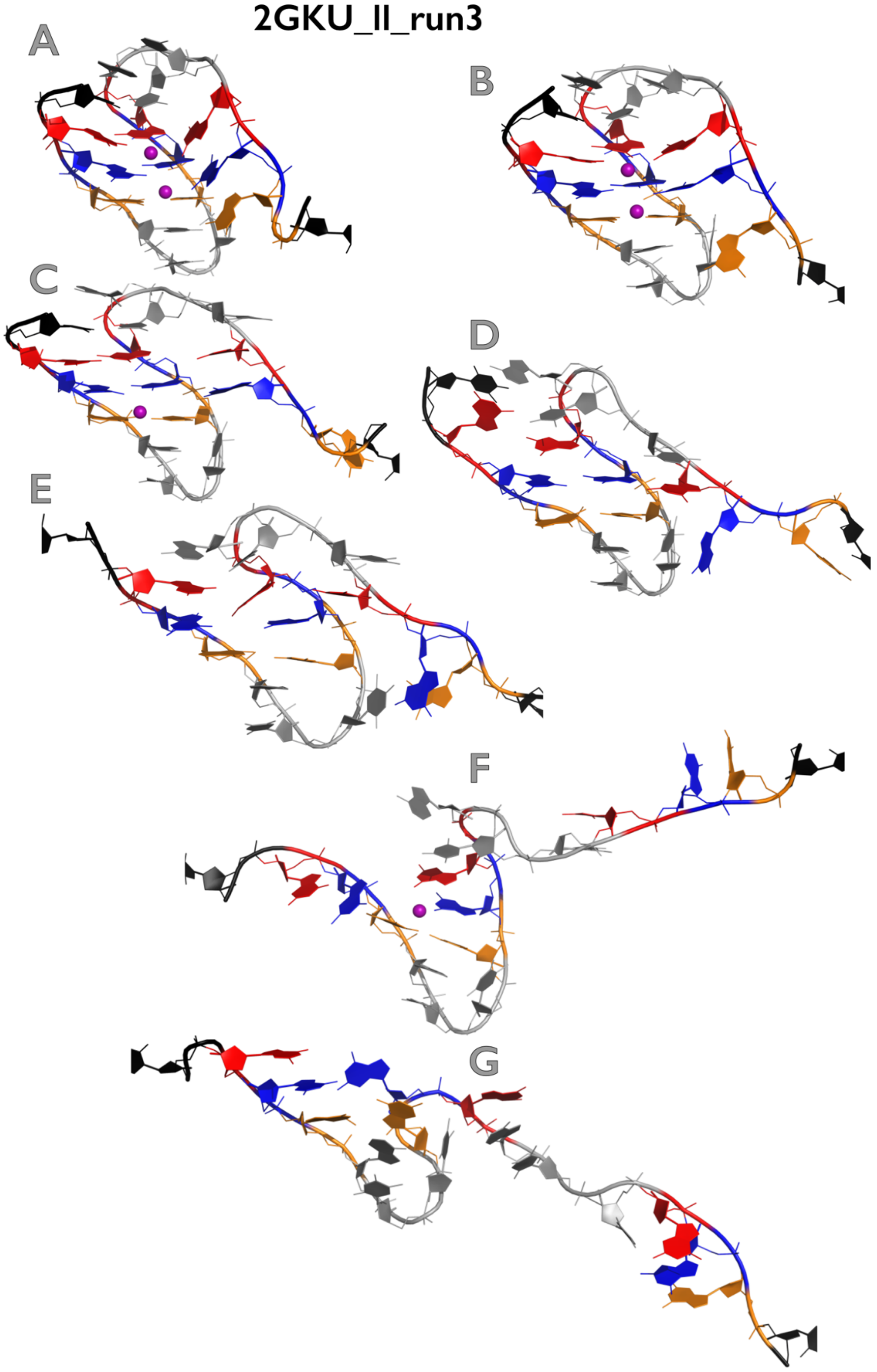

Figure S43D: Most important structural events during third independent *very slow pulling* simulation of 2GKU_t_l_wl_n G-triplex system. See legend of Figure S1B for more details.

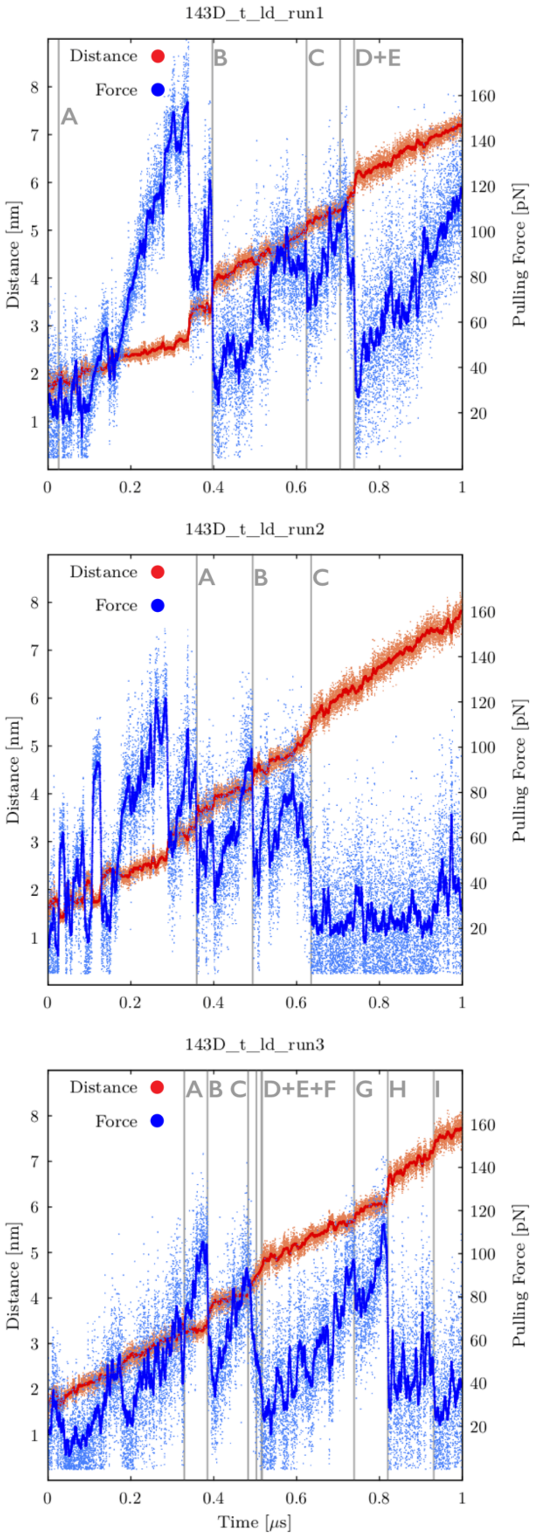

Figure S44A: Time evolution of distance between pulling centers and pulling force during three independent *very slow pulling* simulations of 143D_t_l$_{w}$d G-triplex system (see legend of Figure S40A for more details). See Figures S44B-S44D for inspection of structures corresponding to main structural events.

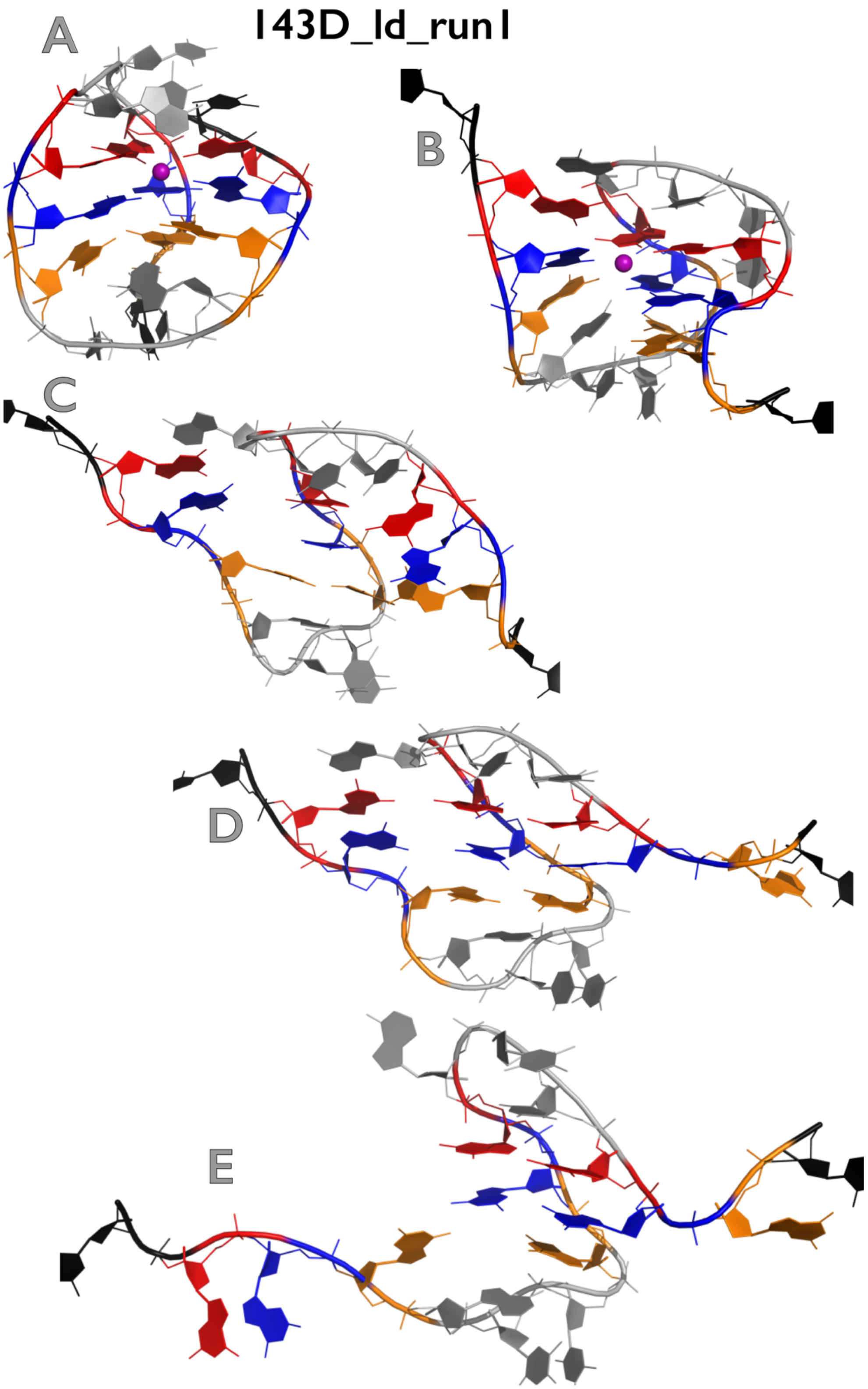

Figure S44B: Most important structural events during first independent *very slow pulling* simulation of 143D_t_l_wd G-triplex system. See legend of Figure S1B for more details.

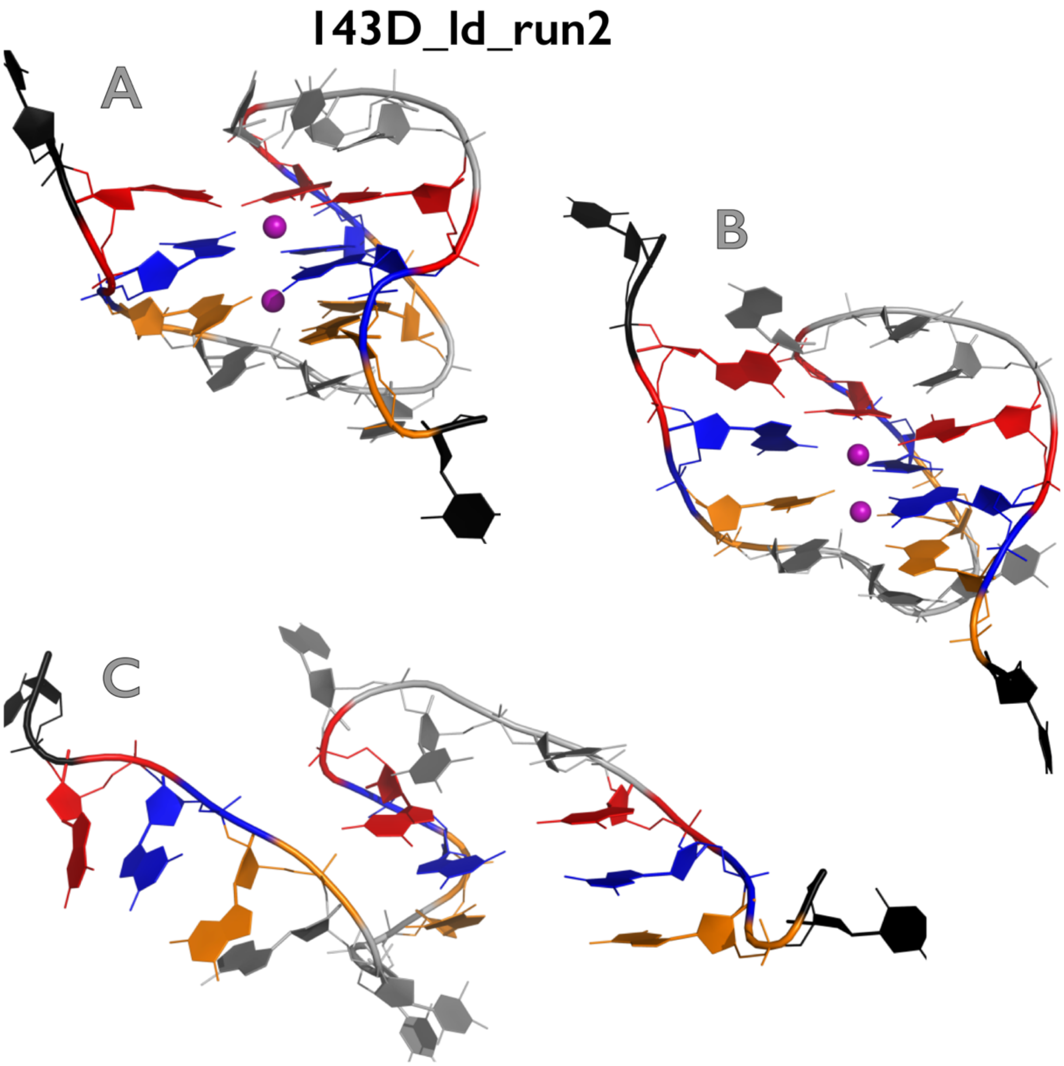

Figure S44C: Most important structural events during second independent *very slow pulling* simulation of 143D_t_l_wd G-triplex system. See legend of Figure S1B for more details.

143D_ld_run3 (part 1)

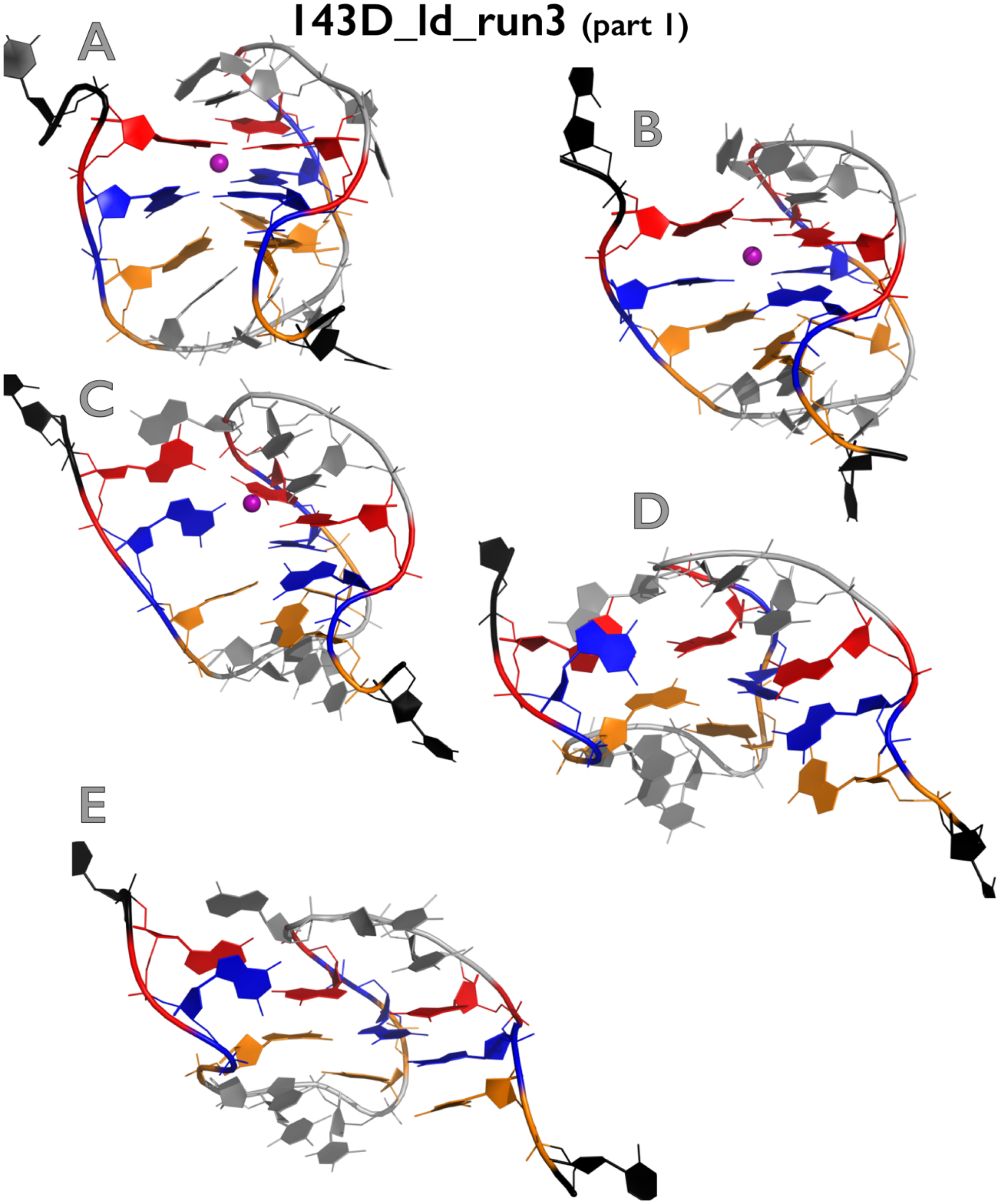

Figure continuing on the next page

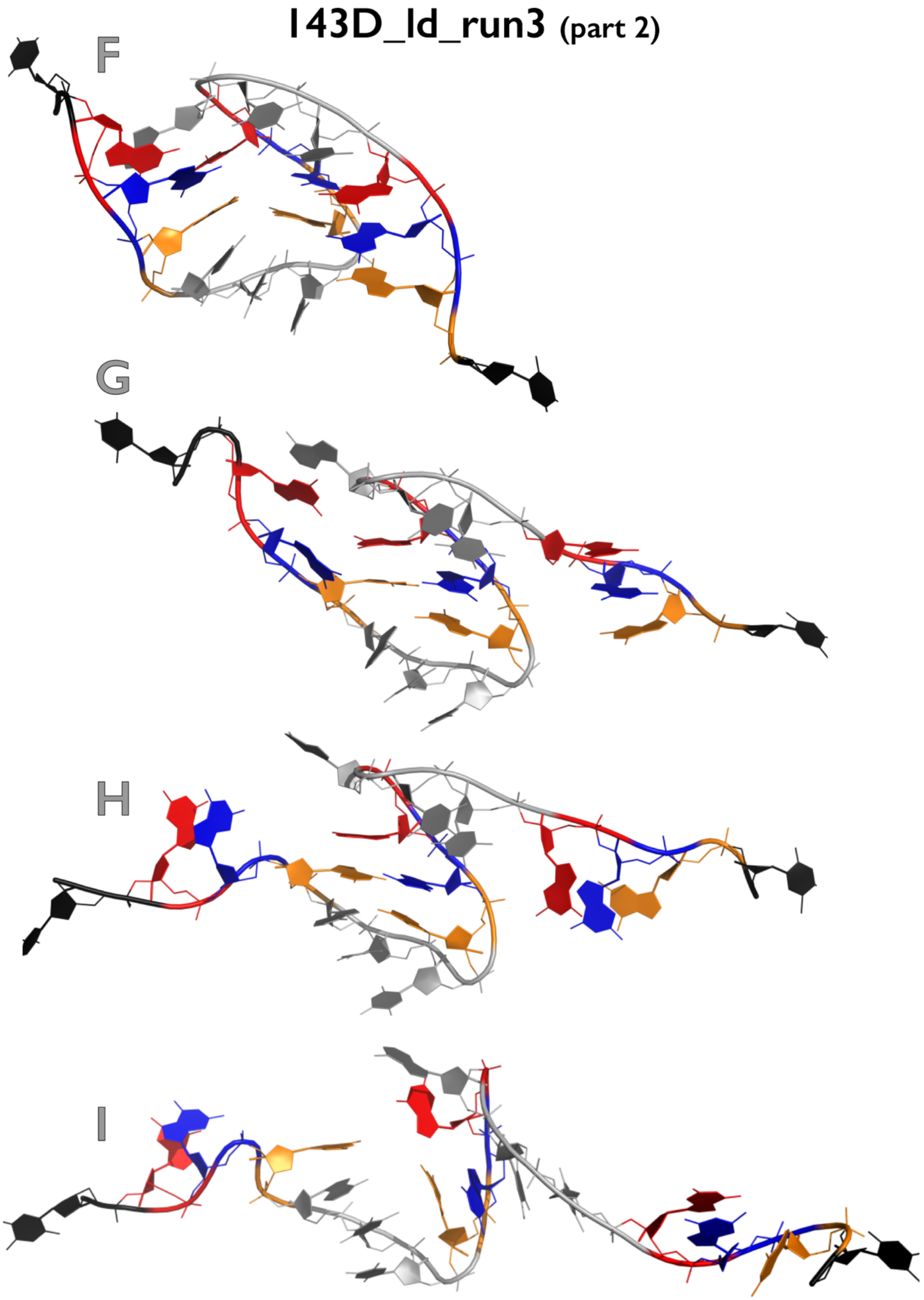

Figure S44D: Most important structural events during third independent *very slow pulling* simulation of 143D_t_l$_{W}$d G-triplex system. See legend of Figure S1B for more details.

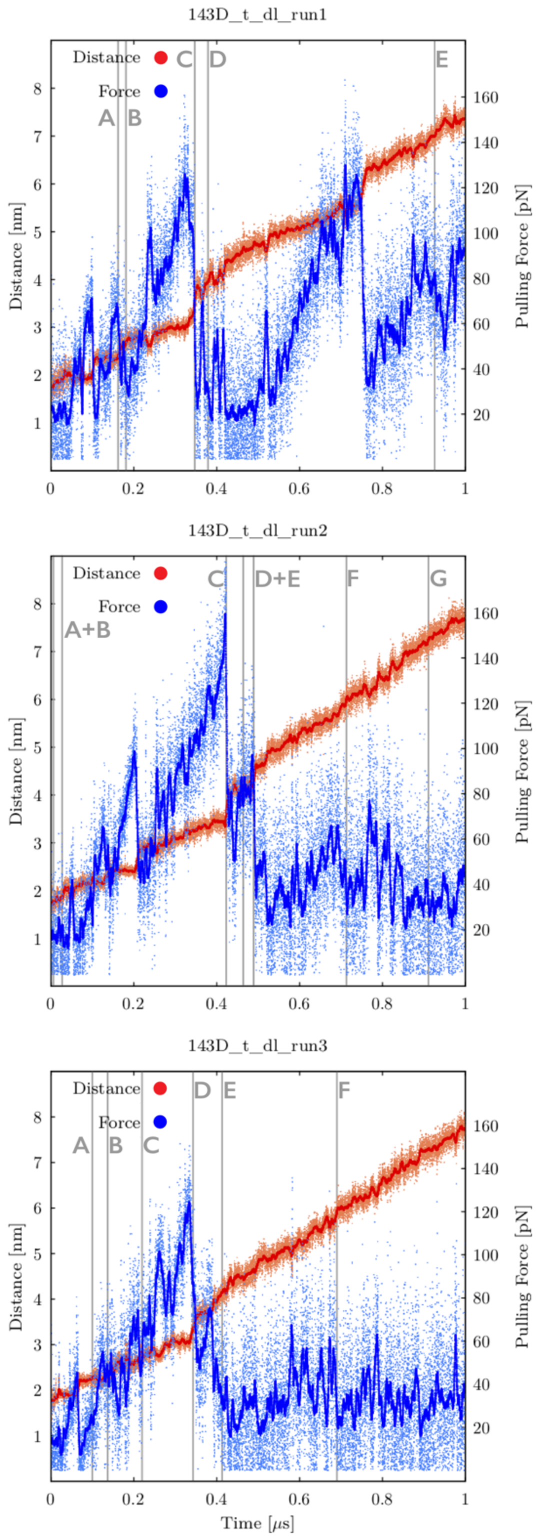

Figure S45A: Time evolution of distance between pulling centers and pulling force during three independent *very slow pulling* simulations of 143D_t_dl$_n$ G-triplex system (see legend of Figure S40A for more details). See Figures S45B-S45D for inspection of structures corresponding to main structural events.

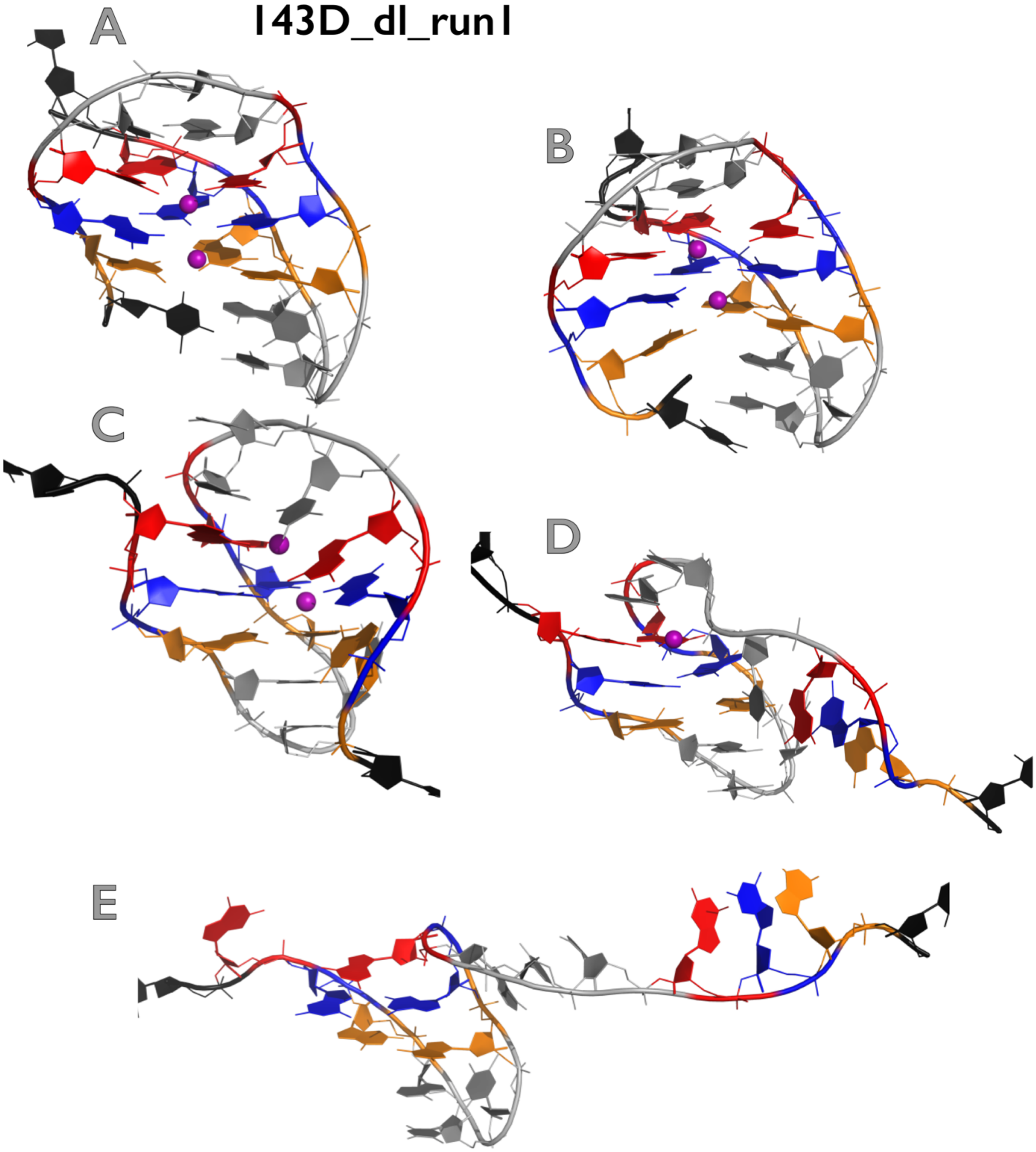

Figure S45B: Most important structural events during first independent *very slow pulling* simulation of 143D_t_dl$_n$ G-triplex system. See legend of Figure S1B for more details.

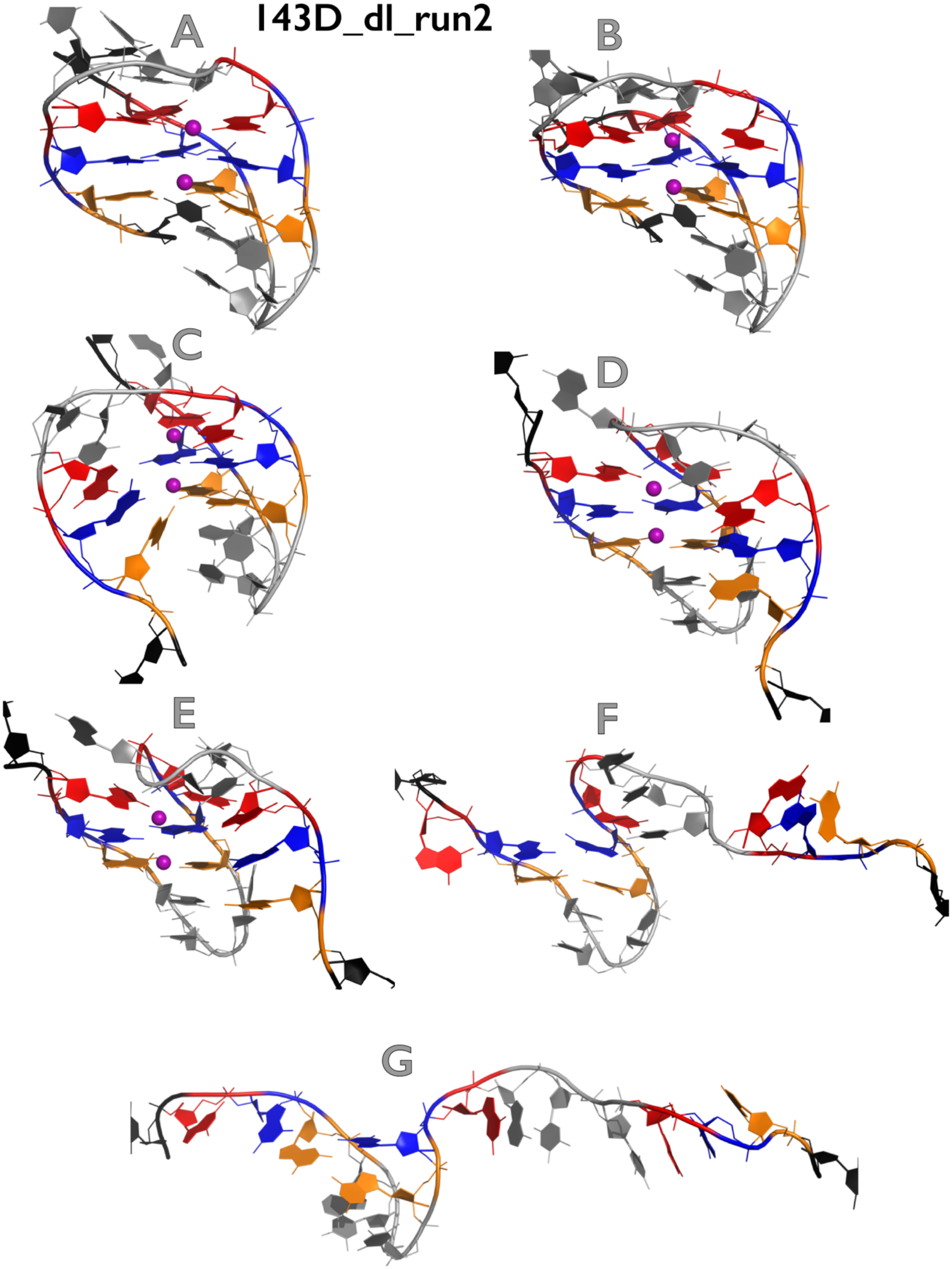

Figure S45C: Most important structural events during second independent *very slow pulling* simulation of 143D_t_dl$_n$ G-triplex system. See legend of Figure S1B for more details.

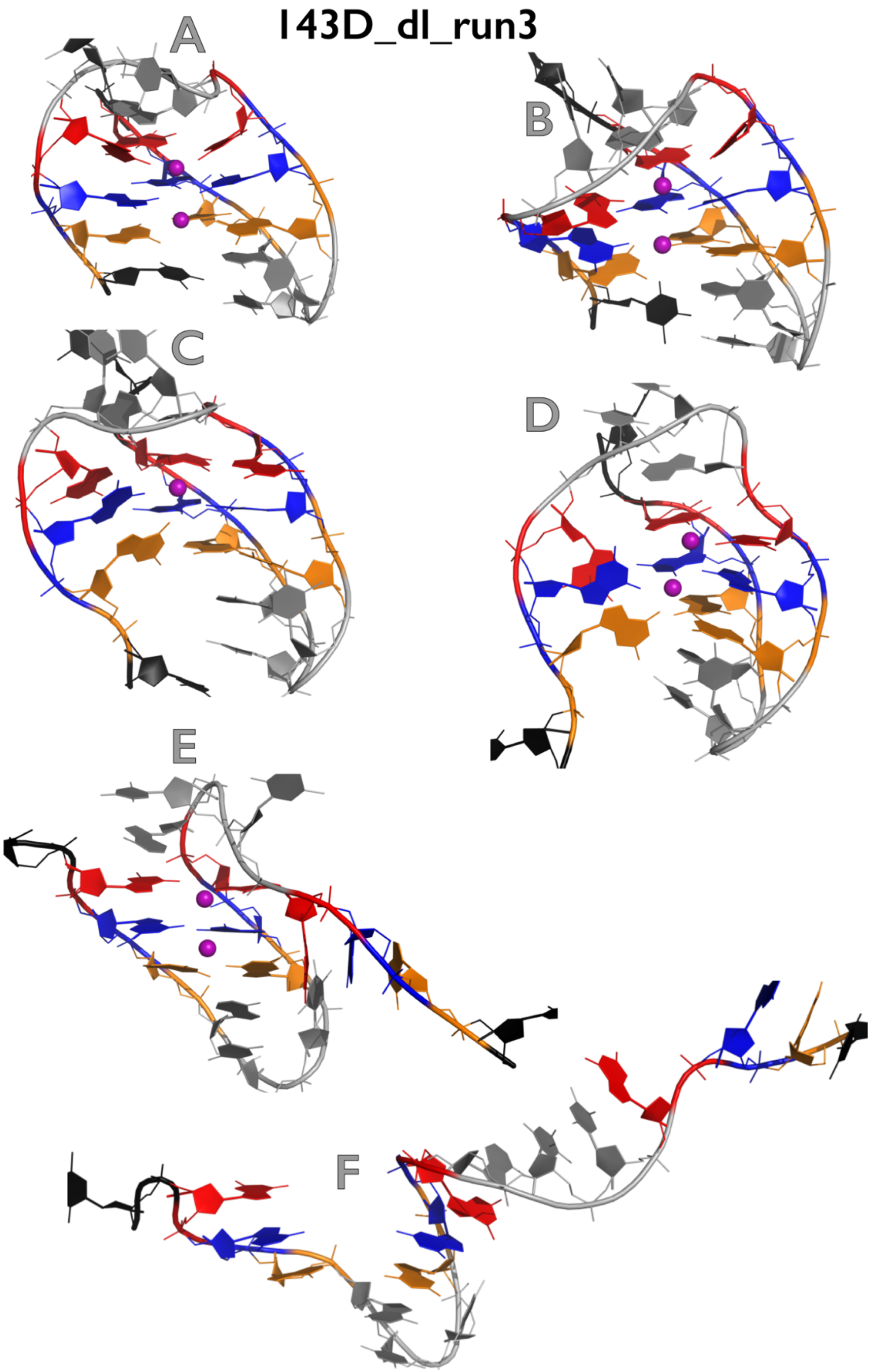

Figure S45D: Most important structural events during third independent *very slow pulling* simulation of 143D_t_dl$_n$ G-triplex system. See legend of Figure S1B for more details.

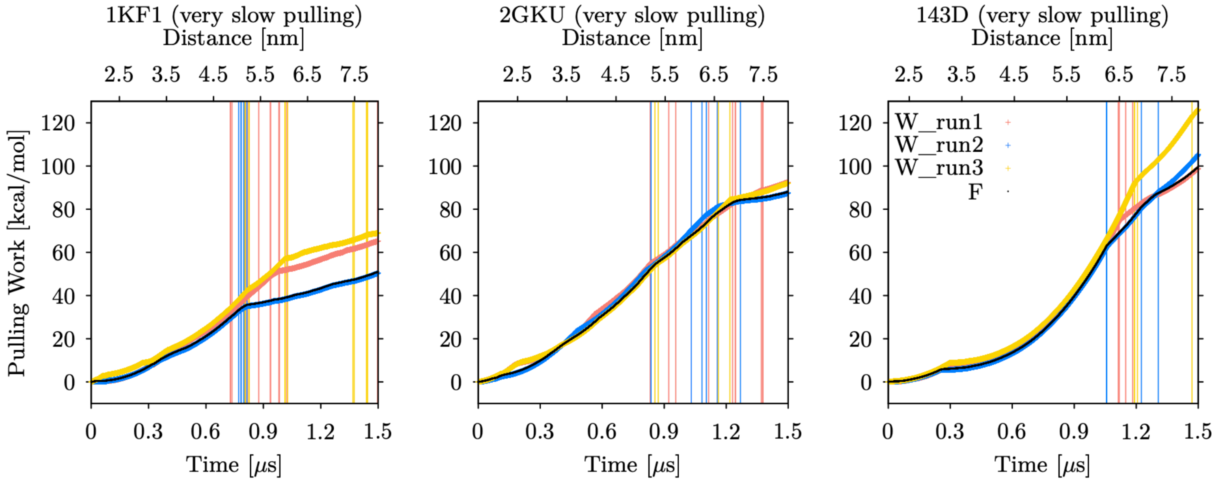

Figure S46: Pulling works required to unfold 1KF1, 2GKU, and 143D GQ systems during *very slow pulling* simulations. Individual pulling work (W) for each from three independent simulations is show in different colors, i.e., salmon, blue and gold for first, second and third simulation, respectively. Their exponential average (F, black line) obtained via Jarzynski equation is also plotted. Vertical lines indicate positions of main structural events for each simulation, i.e., those shown in corresponding Figures S25A, S26A, and S27A.

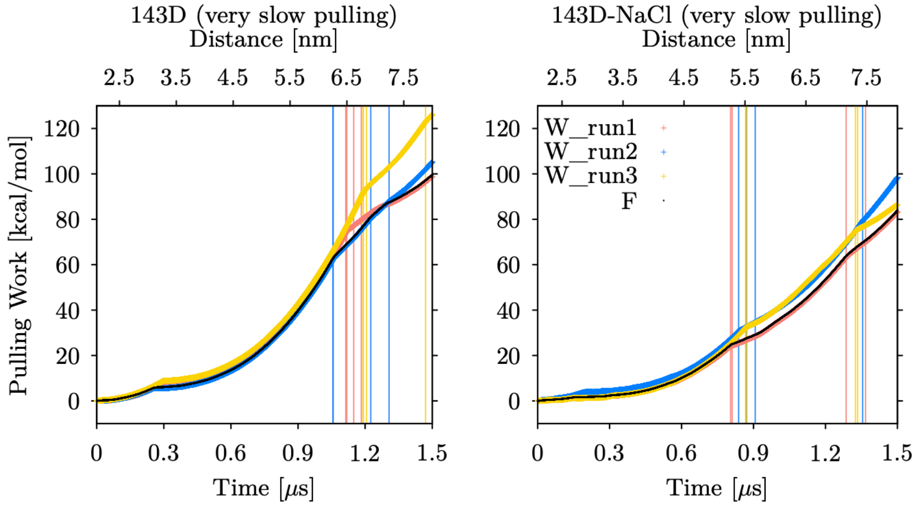

Figure S47: Pulling works required to unfold 143D and $143D_{NaCl}$ GQ systems during *very slow pulling* simulations. See the Figure S46 legend for more details.

REFERENCES

(1) Cang, X. H.; Sponer, J.; Cheatham, T. E. Explaining the Varied Glycosidic Conformational, G-Tract Length and Sequence Preferences for Anti-Parallel G-Quadruplexes. *Nucleic Acids Res.* **2011**, *39* (10), 4499-4512.

(2) Sponer, J.; Mladek, A.; Spackova, N.; Cang, X. H.; Cheatham, T. E.; Grimme, S. Relative Stability of Different DNA Guanine Quadruplex Stem Topologies Derived Using Large-Scale Quantum-Chemical Computations. *J. Am. Chem. Soc.* **2013**, *135* (26), 9785-9796.
